# Isolobal Cationic Iridium Dihydride and Dizinc Complexes: A Dual Role for the ZnR Ligand Enhances H_2_ Activation

**DOI:** 10.1021/acs.inorgchem.4c04058

**Published:** 2024-11-20

**Authors:** Amber M. Walsh, Lia Sotorrios, Rebecca G. Cameron, Anne-Frédérique Pécharman, Barbara Procacci, John P. Lowe, Stuart A. Macgregor, Mary F. Mahon, Neil T. Hunt, Michael K. Whittlesey

**Affiliations:** † Department of Chemistry, University of Bath, Bath BA2 7AY, United Kingdom; ‡ Institute of Chemical Sciences, School of Engineering and Physical Sciences, Heriot-Watt University, Edinburgh EH14 4AS, United Kingdom; § Department of Chemistry and York Biomedical Research Institute, University of York, York YO10 5DD, United Kingdom; ∥ EaStCHEM School of Chemistry, University of St Andrews, St Andrews, North Haugh KY16 9ST, United Kingdom

## Abstract

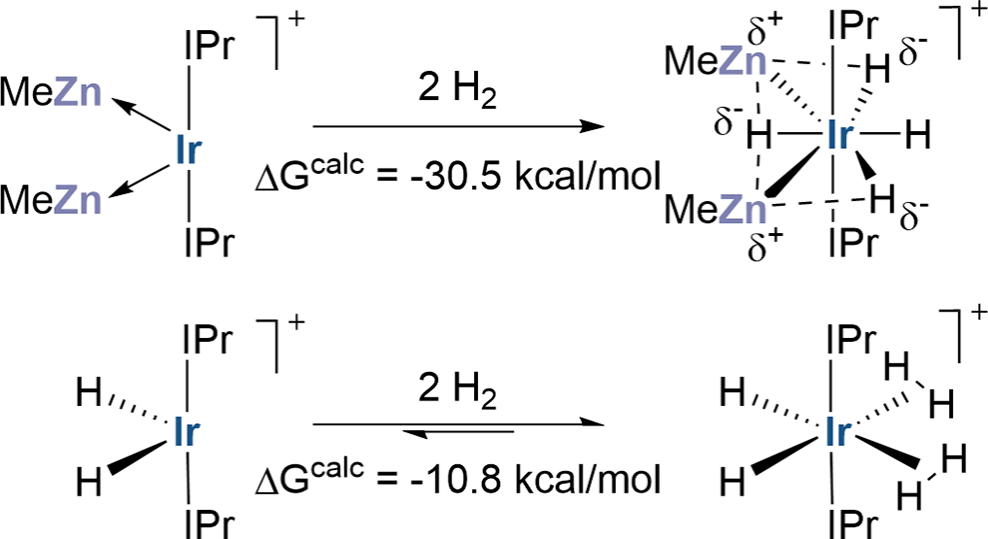

The reaction of [Ir(IPr)_2_H_2_][BAr^F^_4_] (**1**; IPr = 1,3-bis(2,6-diisopropylphenyl)imidazol-2-ylidene; BAr^F^_4_ = B{C_6_H_3_(3,5-CF_3_)_2_}_4_) with ZnMe_2_ proceeds with CH_4_ elimination to give [Ir(IPr)(IPr′)(ZnMe)_2_H][BAr^F^_4_] (**3**, where (IPr′) is a cyclometalated IPr ligand). **3** reacts with H_2_ to form tetrahydride [Ir(IPr)_2_(ZnMe)_2_H_4_][BAr^F^_4_], **4**, that loses H_2_ under forcing conditions to form [Ir(IPr)_2_(ZnMe)_2_H_2_][BAr^F^_4_], **5**. Crystallization of **3** also results in the formation of its noncyclometalated isomer, [Ir(IPr)_2_(ZnMe)_2_][BAr^F^_4_], **2**, in the solid state. Reactions of **1** and CdMe_2_ form [Ir(IPr)_2_(CdMe)_2_][BAr^F^_4_], **6**, and [Ir(IPr)(IPr′)(CdMe)_2_H][BAr^F^_4_], **7**, which reacts with H_2_ to give [Ir(IPr)_2_(CdMe)_2_H_4_][BAr^F^_4_], **8**, and [Ir(IPr)_2_(CdMe)_2_H_2_][BAr^F^_4_], **9**. Structures of **2**–**8** are determined crystallographically. Computational analyses show the various hydrides in **3**–**5** sit on a terminal to bridging continuum, with bridging hydrides exhibiting greater Zn^δ+^···H^δ−^ electrostatic interaction. The isolobal analogy between H and ZnMe ligands holds when both are present as terminal ligands. However, the electrostatic component to the Zn^δ+^···H^δ−^ unit renders it significantly different to a nominally isolobal H···H moiety. Thus, H_2_ addition to **3** is irreversible, whereas H_2_ addition to **1** reversibly forms highly fluxional [Ir(IPr)_2_(η^2^-H_2_)_2_H_2_][BAr^F^_4_], **11**. Computed mechanisms for cyclometalation and H_2_ addition showcase the role of the bridging Zn^δ+^···H^δ−^ moiety in promoting reactivity. In this, the Lewis acidic ZnMe ligand plays a dual role: as a terminal *Z*-type ligand that can stabilize electron-rich Ir centers through direct Ir-ZnMe bonding, or by stabilizing strongly hydridic character via Zn^δ+^···H^δ−^ interactions.

## Abstract 2

The structures and reactivities of [Ir(IPr)_2_H_2_][BAr^F^_4_] and [Ir(IPr)(IPr′)(ZnMe)_2_H][BAr^F^_4_] (IPr = 1,3-bis(2,6-diisopropylphenyl)imidazol-2-ylidene; (IPr′) = cyclometalated IPr) have been probed through a combined experimental and computational investigation to afford insight on the isolobal analogy of H and ZnMe ligands.

## Introduction

Transition metal–main group metal (TM–M′) heterobimetallic complexes are of considerable current interest due to their role in novel catalysis that is founded on cooperative effects to generate novel reactivity distinct to that of the component parts.^1−6^ The rational design of new and more effective TM–M′ catalysts relies on understanding the intrinsic nature of the individual bond activation and forming processes involved. In this context, we have employed an alkane elimination strategy that combines TM–hydrides and M′–alkyls^7−10^ to prepare dual unsaturated TM–M′ complexes as a platform to study well-defined reaction steps of catalytic relevance. These include H_2_ activation,^11−17^ CO addition,^18^ Me migration^18^ and reductive coupling,^19^ where significant heterobimetallic effects have been demonstrated.

Herein, starting with the 14e cationic dihydride [Ir(IPr)_2_H_2_][BAr^F^_4_] (**1**; IPr = 1,3-bis(2,6-diisopropylphenyl)imidazol-2-ylidene; BAr^F^_4_ = B{C_6_H_3_(3,5-CF_3_)_2_}_4_)^20^ and ZnMe_2_, we have employed the same alkane elimination method to target the dual unsaturated heterotrimetallic compound [Ir(IPr)_2_(ZnMe)_2_][BAr^F^_4_] (**2**) and used H_2_ activation as a probe reaction to study TM–M′ cooperativity. The analogous reactions are also explored with CdMe_2_. The direct comparison of **1** and **2** would permit the widely discussed isolobality of the H and ZnR ligands to be interrogated.^21−24^ Our findings demonstrate that H_2_ activation is greatly enhanced by the presence of ZnMe due to its flexible dual role in bonding, namely its ability to stabilize both a low-valent Ir center in the reactant and strongly hydridic character in the products. While this exposes limits in H/ZnR isolobality, it also highlights the potential of TM–M′ heterometallic cooperativity in enhancing small molecule activation.

## Results and Discussion

### Synthesis of [Ir(ZnMe)_2_]^+^ Species and Reactivity with H_2_

**Scheme 1:**
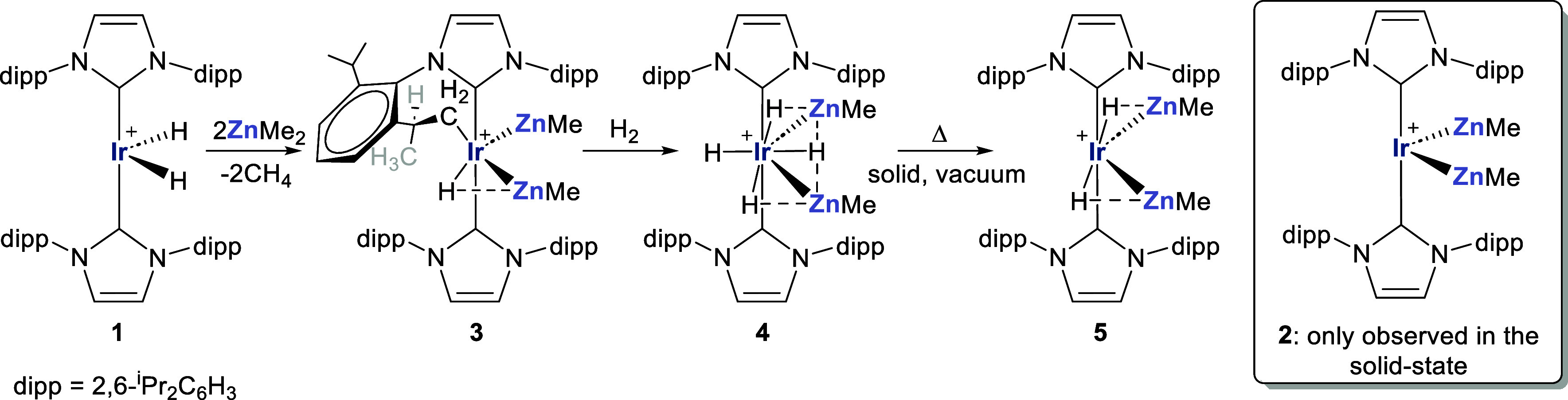
Synthesis of [Ir(IPr)(IPr′)(ZnMe)_2_H][BAr^F^_4_] (**3**) and Reactivity with H_2_ In all cases, [BAr^F^_4_]^−^ counterions are omitted for clarity, as are the agostic interactions in **1**. Solid bonds between Ir and Zn are drawn when their separation ≤ sum of their covalent radii. Solid Ir–H bonds intimate that *r*(Ir–H) < *r*(Zn–H); Zn···H interactions are discussed in computational section.

**Figure 1:**
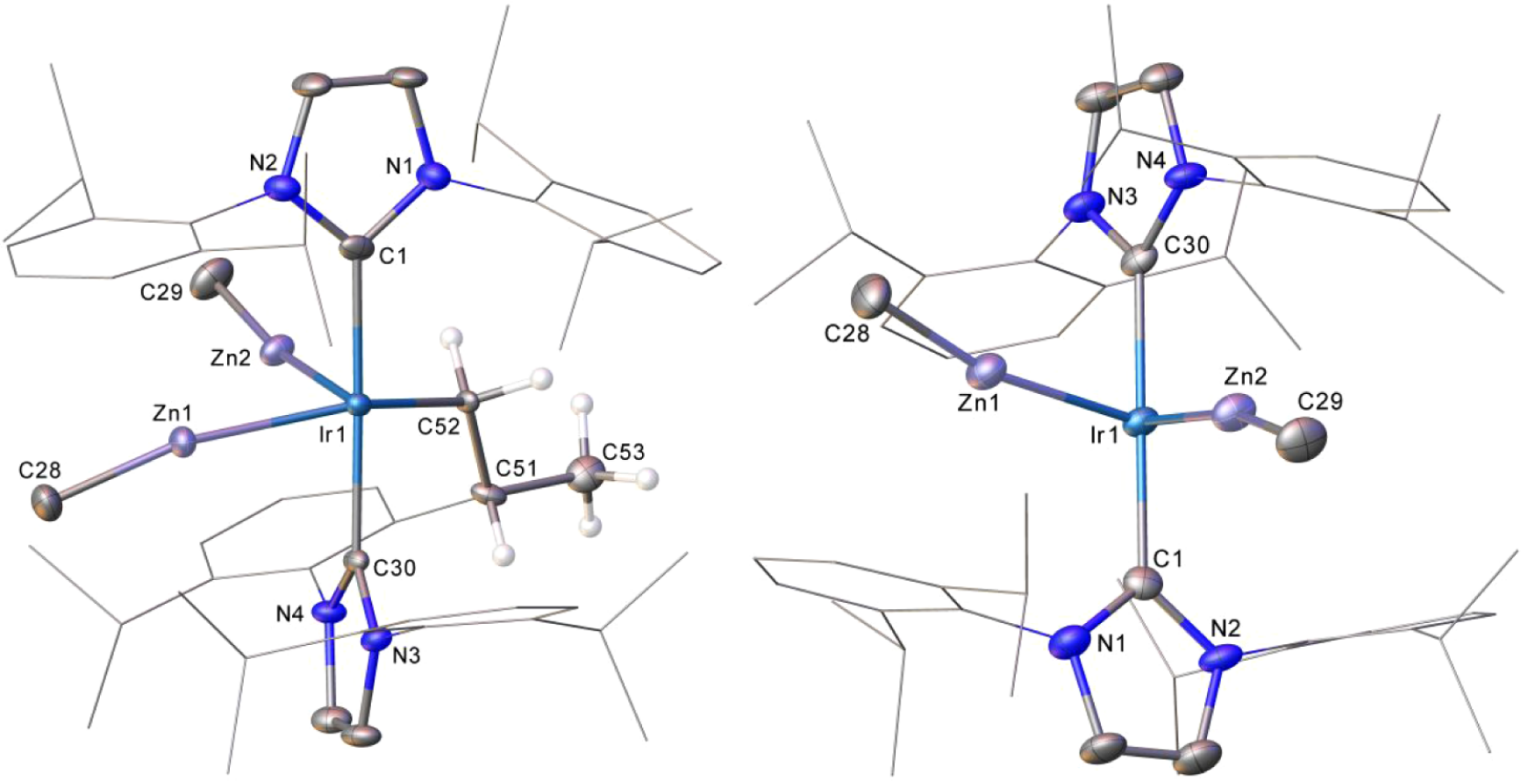
Structures of the cyclometalated cation in (left) (0.5)**3**(0.5)**2** and (right) the noncyclometalated cation in (0.25)**3**(0.75)**2**. Thermal ellipsoids are shown at 30% probability in both cases. For clarity, minor disordered components have been omitted, as have all hydrogen atoms barring those attached to C51, C52 and C53 in (0.5)**3**(0.5)**2** where the hydride ligand could not be reliably located. Dipp substituents are depicted as wireframes, also for visual ease.

Addition of ZnMe_2_ (2 equiv) to a C_6_H_5_F solution of [Ir(IPr)_2_H_2_][BAr^F^_4_] (**1**) generated one major product in the time of mixing which, rather than the anticipated IrZn_2_ product, [Ir(IPr)_2_(ZnMe)_2_][BAr^F^_4_] (**2**, vide infra), was shown by ^1^H NMR spectroscopy (Figures S4–S11) to be the isomer, [Ir(IPr)(IPr′)(ZnMe)_2_H][BAr^F^_4_] (**3**), where IPr′ denotes a cyclometalated IPr ligand (Scheme 1).^25−29^ Especially revealing was the presence of an Ir–H resonance (THF-*d*_8_) at δ −4.14 that integrated in a 1:3:3 ratio with two, sharp ZnMe resonances at δ −0.94 and −0.95. At 298 K, the methine and methyl groups of the dipp substituents appeared as a mixture of sharp signals and broader baseline features; at 219 K, eight methine resonances were apparent between ca. δ 2.6–1.9 which all arose from a single isomer of **3**. While **3** was the only detected product in solution, crystallization efforts (Figure 1) uniformly yielded samples that contained a disordered mixture of **3** and **2** (vide infra).

Exposure of **3** to H_2_ generated [Ir(IPr)_2_(ZnMe)_2_H_4_][BAr^F^_4_] (**4**) in the time of mixing (Scheme 1).^30^ By ^1^H NMR spectroscopy (Figures S15–S21) at room temperature, **4** showed a single, exchange-broadened hydride resonance at δ −10.77 in a 4:6 ratio with a single ZnMe resonance at δ −0.96. At 223 K, the hydride signal had decoalesced into a 1:1:2 set of three separate resonances at δ −9.62, −10.44 and −11.82, which were all still shown to be in exchange through a ROESY measurement. The hydrides afforded *T*_1_ values (400 MHz, 223 K) of 465, 316, and 388 ms respectively, characteristic of classical hydrides, leading to the designation of **4** as a tetrahydride species.^31^ In accord with this, the ^13^C{selective-^1^H} NMR spectrum showed a quintet Ir–*C*_NHC_ resonance (^2^*J*_H–Ir–C(NHC)_ = 4 Hz). The spectrum of the dihydride salt **1** displayed the expected triplet, with a comparable sized splitting (5 Hz).

When **3** was reacted with D_2_ in place of H_2_, three Ir–H resonances were still observed in the low temperature ^1^H NMR spectrum (Figures S24–S27), but these were now all shifted to slightly higher frequencies (Δδ = 65–110 ppb) of the resonances of **4**, consistent with formation of [Ir(IPr)(IPr-*d*)(ZnMe)_2_HD_3_][BAr^F^_4_] (**4-*****d***_**4**_).^32^ The hydride resonances were of low intensity and no longer integrated in a 1:1:2 ratio implying that **4-*****d***_**4**_ exists as a mixture of isomers with residual hydride in all three possible sites. We can therefore exclude H/D exchange proceeding via initial reductive coupling of the IrH/IPr′ ligands in **3** to give **2**, which then adds D_2_ (vide infra).

**Figure 2:**
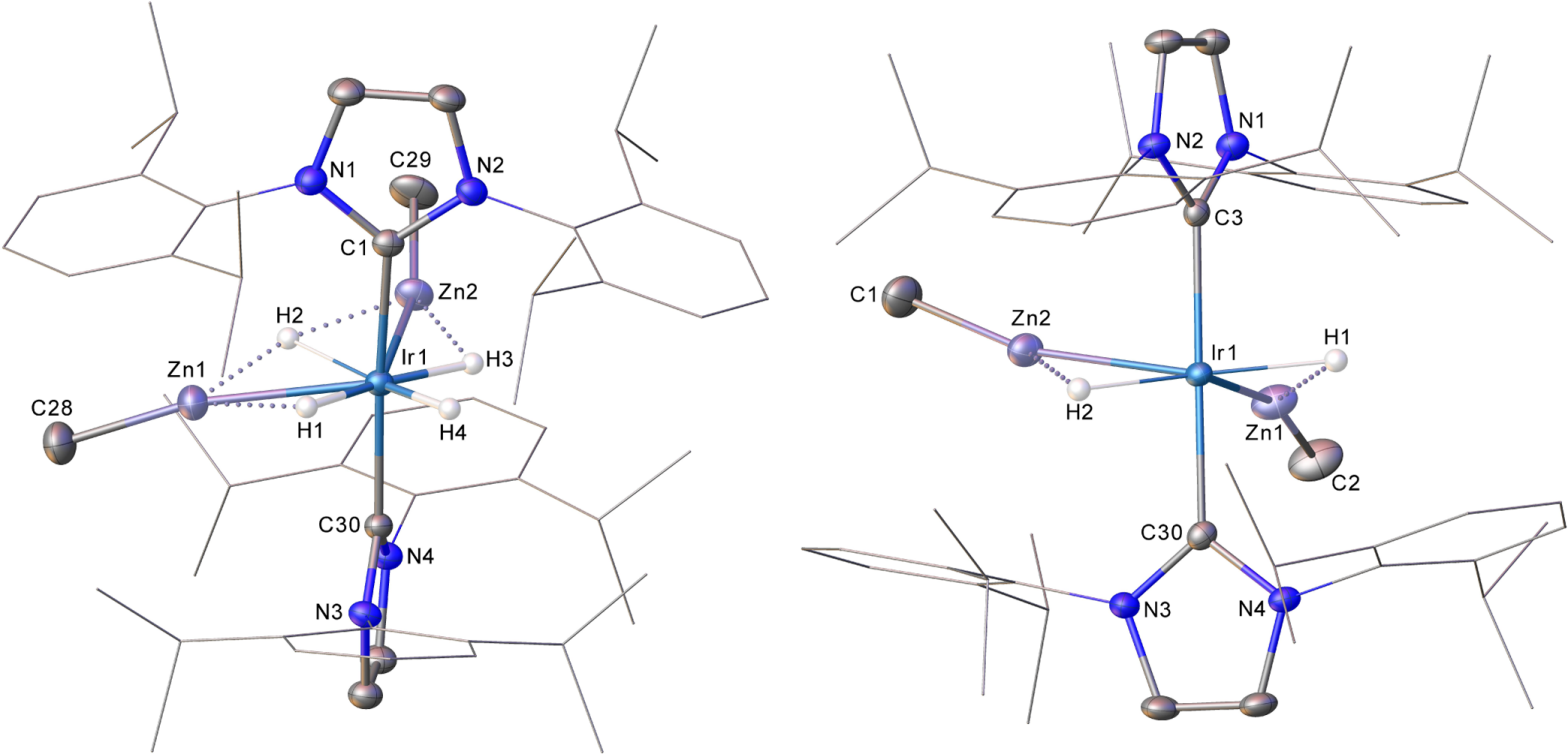
Structures of the cation in (left) [Ir(IPr)_2_(ZnMe)_2_H_4_][BAr^F^_4_] (**4**) and (right) [Ir(IPr)_2_(ZnMe)_2_H_2_][BAr^F^_4_] (**5**). Thermal ellipsoids are shown at 30% probability in both cases. For clarity, minor disordered components have been omitted, as have all hydrogen atoms with the exceptions of hydride ligands. Dipp substituents are depicted as wireframes, also for visual ease. As denoted in Scheme 1, solid Ir–H bonds intimate that *r*(Ir–H) < *r*(Zn–H).

In contrast to the facile intramolecular hydride exchange in **4**, intermolecular processes proved more difficult. Thus, exchange with D_2_ was only observed upon heating at 40 °C (Figures S25–S26), while elimination of H_2_ to generate the dihydride salt [Ir(IPr)_2_(ZnMe)_2_H_2_][BAr^F^_4_] (**5**, Scheme 1) necessitated heating a solid sample of the compound at 60–80 °C under dynamic vacuum for 1–2 weeks. Even then, as conversion to **5** was incomplete, characterization of the product was limited to ^1^H NMR data (Figure S28); an Ir–H resonance (δ −4.15) that was in a 2:6 ratio with a single Ir–ZnMe resonance at δ – 0.98. The fortuitous isolation of a small number of suitable quality single crystals did allow us to confirm the structure of **5** by X-ray crystallography, as shown in Figure 2.^33^

### Reaction of **1** with CdMe_2_

**Scheme 2:**
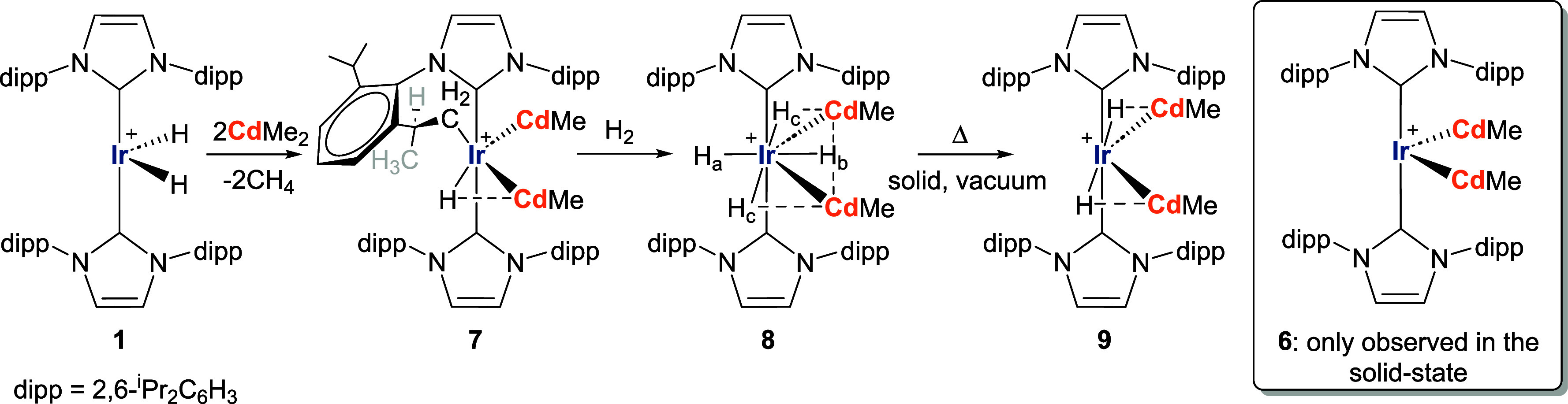
Synthesis and Reactivity of [Ir(IPr)(IPr′)(CdMe)_2_H][BAr^F^_4_] (**7**) In all cases, [BAr^F^_4_]^−^ counterions are omitted for clarity, as are the agostic interactions in **1**. Solid bonds between Ir and Cd bonds are drawn for separations ≤ sum of their covalent radii. Solid Ir–H bonds intimate that *r*(Ir–H) < *r*(Cd–H); Cd···H interactions are discussed in computational section.

**Figure 3:**
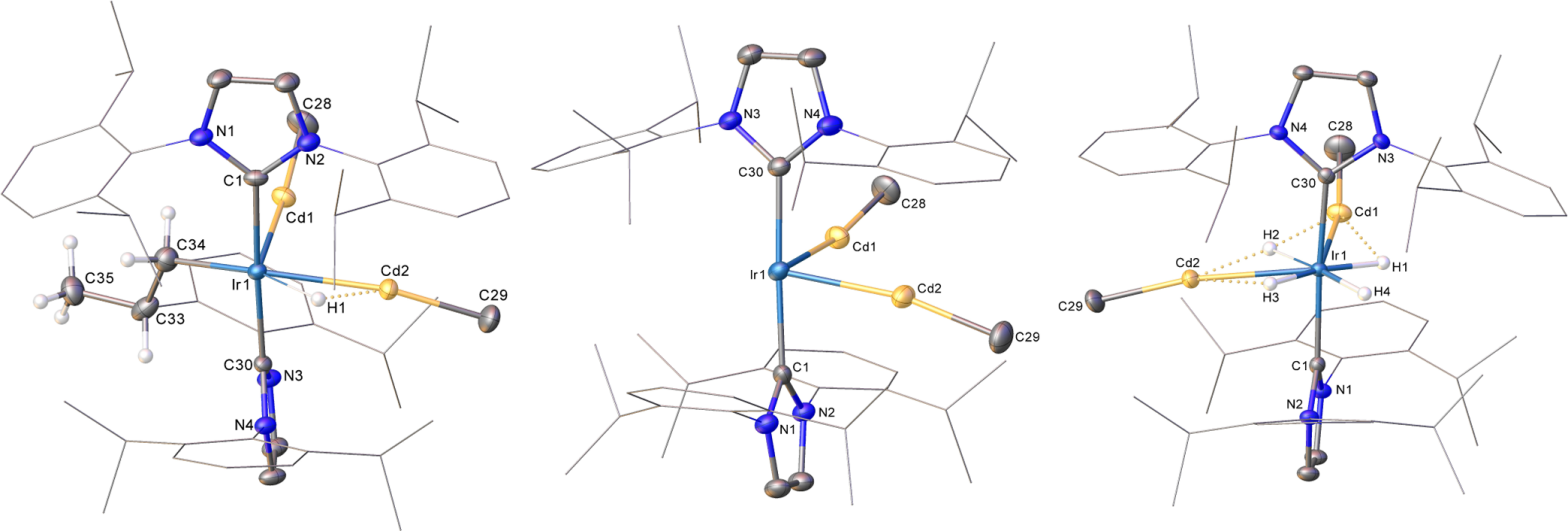
Structures of the cations in (left) [Ir(IPr)(IPr)′(CdMe)_2_H][BAr^F^_4_] (**7**), (center) [Ir(IPr)_2_(CdMe)_2_][BAr^F^_4_] (**6**) and (right) [Ir(IPr)_2_(CdMe)_2_H_4_][BAr^F^_4_] (**8**). Thermal ellipsoids are shown at 30% probability in all cases. For clarity, minor disordered components have been omitted, as have all hydrogen atoms with the exceptions of the C33, C34 and C35 bound hydrogens in **7** and the hydride ligands in both **7** and **8**. Dipp substituents are depicted as wireframes, also for visual ease. As denoted in Scheme 2, solid Ir–H bonds intimate that *r*(Ir–H) < *r*(Cd–H).

Analogous reactivity took place with CdMe_2_ (Scheme 2), to generate [Ir(IPr)(IPr′)(CdMe)_2_H][BAr^F^_4_] (**7**, Figures S29–S36),^34,35^ while at the same time also affording [Ir(IPr)_2_(CdMe)_2_][BAr^F^_4_] (**6**) in the solid-state. Exposure of **7** to H_2_ formed [Ir(IPr)_2_(CdMe)_2_H_4_][BAr^F^_4_] (**8,**Figures S37–S39), which generated [Ir(IPr)_2_(CdMe)_2_H_2_][BAr^F^_4_] (**9**, Figures S33–S36), upon application of heat and vacuum. Compounds **6**–**8** were structurally characterized as shown in Figure 3. The structures are notable in adding to the limited number of TM(CdR)_*x*_ (*x* > 1) compounds^36−39^ and even fewer examples of Cd–H containing species.^40−42^

The tetrahydride **8** showed comparable fluxionality to **4**, exhibiting a single broad hydride resonance at 298 K (Figure S37) and a 1:1:2 set of three separate resonances (δ −7.95 (H_b_), −9.98 (H_a_), −10.44 (H_c_)) at low temperature (Figure S38); ROESY again confirmed low temperature exchange of all the hydrides (Figure S39). The presence of the *I* = 1/2 ^111^/^113^Cd nuclei proved informative about the extent of Ir–H···Cd interactions. Thus, both H_b_ and H_c_ (Scheme 2) showed much larger ^2^*J*_HCd_ splittings (286 and 372 Hz respectively) than H_a_ (41 Hz).^43^ In **7**, the hydride resonance showed a single set of broad satellites,^44^ with a large splitting of 426 Hz resulting from the adjacent H–Ir···Cd coupling; we assume that the splitting to the second CdMe ligand is lost within the line width (ca. 45 Hz) of the main hydride resonance. In the ^1^H–^113^Cd HMBC spectrum (Figure S35), there was a correlation between the hydride and only one of the two ^113^Cd NMR resonances (at δ −178).

### Structural Characterization

The attempted crystallization of **3** afforded two crystal morphologies, yellow needles and yellow-orange blocks. The X-ray structure of the yellow needles (designated (0.5)**3**(0.5)**2**) revealed, unexpectedly, the presence of an equimolar ratio of **3** disordered with noncyclometalated [Ir(IPr)_2_(ZnMe)_2_][BAr^F^_4_] (**2**), while the structure of the blocks, (0.25)**3**(0.75)**2**, contained a 1:3 ratio of disordered **3**:**2** (Figure 1). **2** was only ever evident in the solid-state, as redissolution of crystals for NMR analysis showed only **3**.

The structure of the **2** component in (0.25)**3**(0.75)**2** showed no evidence for any agostic stabilization by the IPr ligands; the closest Ir···H_3_C and Ir···HC distances (2.954 and 3.418 Å respectively) are both significantly longer than the agostic Ir···H_3_C distances of 2.116 and 2.206 Å^45^ measured in **1**.^20^ The readily modeled disorder in both (0.5)**3**(0.5)**2** and (0.25)**3**(0.75)**2** did not extend to the positions of the Ir/Zn cores that are common to both cations in each structure. Notably, the Ir–Zn distances are asymmetric within each of the individual structures of (0.5)**3**(0.5)**2** (2.3778(6) Å, 2.4006(6) Å) and (0.25)**3**(0.75)**2** (2.3874(12) Å, 2.3762(11) Å) and, further, differ between structures. This may suggest a degree of flexibility in the cation cores of **3** and **2** or, indeed, perhaps the influence of some solid-state packing effects. However, all Ir–Zn distances fall in the accepted range for a bonding interaction, based on the sum of the Ir and Zn covalent radii (Ir, 1.41 Å; Zn, 1.22 Å).^46−49^ This criterion precludes a Zn–Zn interaction in either (0.5)**3**(0.5)**2** or (0.25)**3**(0.75)**2**, where the inter-Zn distances are 3.1660(9) and 3.2407(16) Å.

The X-ray structure of **4** (Figure 2) was obtained initially using crystals isolated from a reaction mixture of **3** with H_2_ and was confirmed following a single crystal-to-crystal transformation involving exposure of a single crystal of (0.25)**3**(0.75)**2** to a flow of H_2_ for ca. 1 h. In comparison to the structures (0.5)**3**(0.5)**2** and (0.25)**3**(0.75)**2**, the Ir–Zn distances in **4** are significantly longer and almost symmetrical (2.4930(5), 2.4963(5) Å). The presence of the four hydride ligands leads to a widening of the Zn–Ir–Zn angle to 101.199(17)° relative to the angles in both (0.5)**3**(0.5)**2** (82.99(2)°) and (0.25)**3**(0.75)**2** (84.56(4)°). All of the hydrides in **4** were readily located and freely refined; their character is discussed further in the computational section. The X-ray structure of **5** (Figure 2) revealed a Zn–Ir–Zn angle (90.18(3)°) intermediate between the values for (0.5)**3**(0.5)**2** and (0.25)**3**(0.75)**2** and that of **4**.

The X-ray structures of the cations in **6**–**8**, the cadmium congeners of compounds **2**, **3** and **4**, are shown in Figure 3. The trend in Ir–Cd distances (**6**: 2.5753(4), 2.5953(4) Å; **7**: 2.5770(3), 2.6130(3) Å; **8**: 2.6828(2), 2.6981(2) Å) mirrors that seen for **2**-**4**, notwithstanding that, individually, Ir–Cd bond lengths reflect the increased radius (0.22 Å) of Cd relative to Zn.^46^ The sequential Cd–Ir–Cd angles (83.47(2), 82.31(2) and 100.97(2)°) also broadly reflect the extremes observed in **2**–**4**.

### Computational Studies

#### Computed Geometries and Electronic Structures

**Figure 4:**
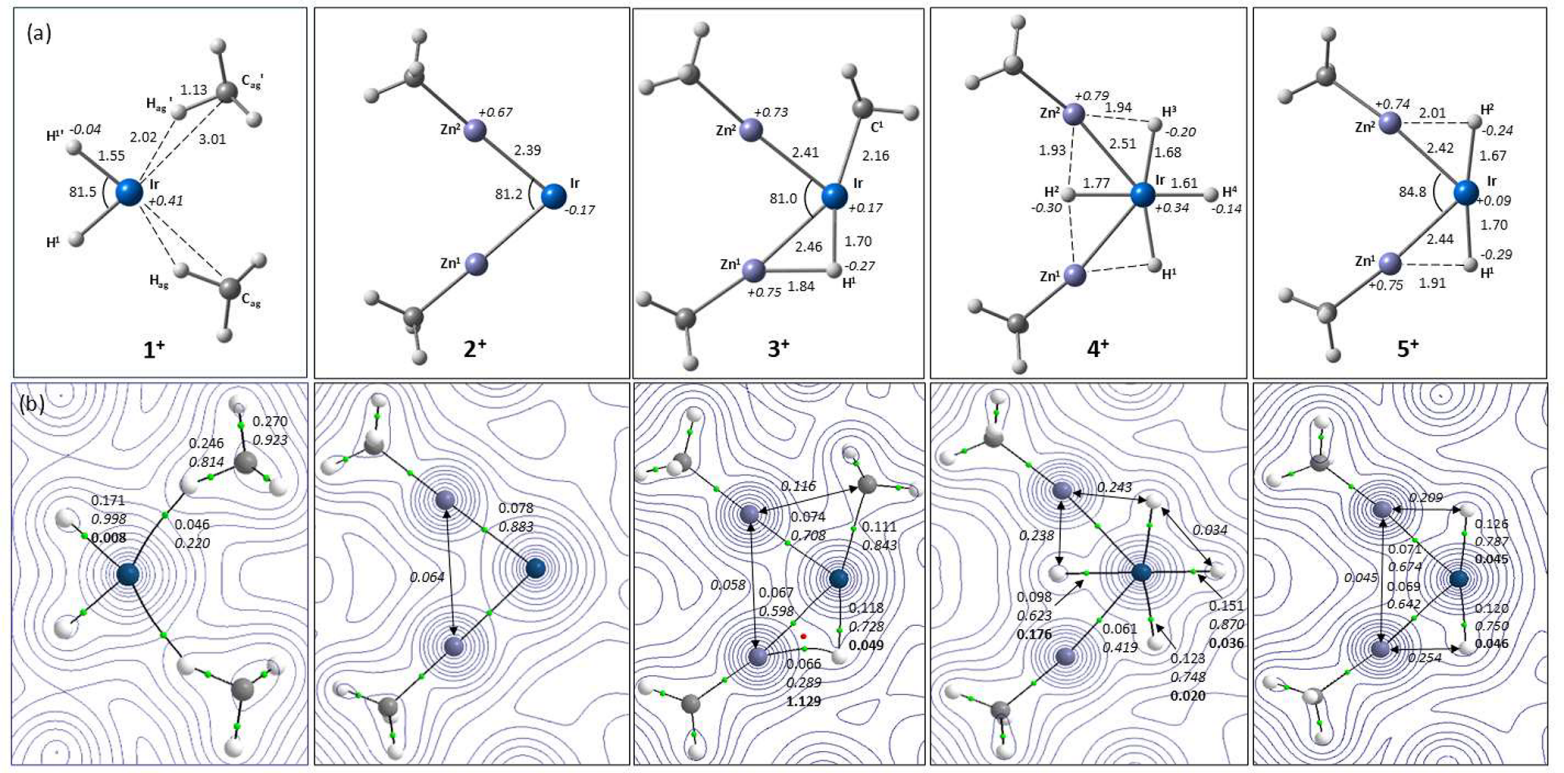
Details of the equatorial Ir–ligand plane in **1**^**+**^–**5**^**+**^ (axial IPr ligands omitted for clarity). (a) Computed geometries with selected distances in Å and QTAIM atomic charges in italics; (b) QTAIM molecular graphs with density contours in the equatorial plane. BCPs and RCPs shown in green and red respectively with the associated BCP ρ(r) (au) in plain text, delocalization indices in italics and, for bond paths to hydrides, ellipticities in bold. Delocalization indices between selected atoms not linked by a bond path are also indicated.

Geometries for the cations **1**^**+**^–**5**^**+**^ were optimized based on the crystallographic structures using the BP86 functional including a correction for dispersion (D3BJ) and Figure 4a shows key distances in the equatorial plane. The calculated Ir–Zn distances follow the trends established crystallographically and lengthen with the number of hydrides present, from 2.39 Å in **2**^**+**^ to 2.51 Å in **4**^**+**^. Outside the crystallographic environment, **1**^**+**^, **2**^**+**^ and **4**^**+**^ all optimize with effective *C*_2_ symmetry. The range of computed Ir–H distances (from 1.55 to1.77 Å) and Zn–H distances (from 1.84 to1.93 Å) suggest significant variations in hydride character and these were explored further with Quantum Theory of Atoms in Molecules (QTAIM).

Computed molecular graphs in the equatorial planes of **1**^**+**^–**5**^**+**^ are shown in Figure 4b. **1**^**+**^ provides a benchmark for a terminal Ir–H bond, with a short Ir–H distance (1.55 Å), a relatively high bond critical point (BCP) electron density (ρ(r) = 0.171 au) and delocalization index (DI(Ir|H) = 0.998). The low BCP ellipticity (ε = 0.008) is also an indicator of terminal Ir–H (i.e., σ-bonding) character.^50^ Bond paths to two IPr Me hydrogens are consistent with the presence of agostic interactions, where the computed Ir···C_agostic_ distance of 3.01 Å compares well with the average of 2.99 Å seen experimentally.

In contrast, cyclometalated **3**^**+**^ exhibits a bridging hydride, with Ir–Zn^1^, Zn^1^–H^1^ and Ir–H^1^ bond paths and an associated ring critical point. The Ir–H^1^ bond is longer and weaker than in **1**^**+**^ (1.70 Å, ρ(r) = 0.118 au; DI(Ir|H^1^) = 0.728) and the increased BCP ellipticity of 0.049 indicates some peripheral interaction with Zn^1^ (Zn^1^–H^1^ = 1.84 Å; ρ(r) = 0.066; DI(Zn^1^|H^1^) = 0.289). In **4**^**+**^, H^4^ shows terminal character (Ir–H^4^: 1.61 Å; ρ(r) = 0.151 au; DI = 0.870; ε = 0.036) and the H^1^/H^3^ pair show more bridging character (Ir–H^2^: 1.68 Å; ρ(r) = 0.123 au; DI = 0.748; ε = 0.020). This is accentuated for H^2^ (Ir–H^2^: 1.77 Å; ρ(r) = 0.098 au; DI = 0.623; ε = 0.176) which suggests interaction with both adjacent Zn centers. While no Zn···H^2^ bond paths are computed, the DI(Zn^1^|H^2^) and DI(Zn^2^|H^2^) values of ca. 0.24 indicate these Zn···H^2^ interactions are only slightly reduced compared to the Zn^1^–H^1^ interaction in **3**^**+**^ where a bond path is present. The trans-H^1^–Ir–H^2^ unit in **5**^**+**^ is similar to the trans-H^1^–Ir–H^3^ unit in **4**^**+**^.

The molecular graph of **2**^**+**^ shows the highest Ir–Zn BCP ρ(r) values (0.078 au) but, unlike **1**^**+**^, no agostic interactions (shortest calculated Ir···H_IPr_ = 3.41 Å). Previously we^16^ and others^51,52^ have identified Zn···Zn interactions in related TM-Zn_2_ species, but these are not present in **2**^**+**^, **3**^**+**^ or **5**^**+**^ (Zn···Zn > 3.1 Å; DI(Zn^1^|Zn^2^) < 0.1). Molecular graphs of the Ir–Cd_2_ species **6**^**+**^–**9**^**+**^ are similar to their dizinc analogues (Figures S62–S65). For **8**^**+**^, Cd···H^1^/H^3^ and Cd···H^2^ interactions are consistent with the large ^1^*J*_HCd_ values seen experimentally (372 and 286 Hz), while the ^1^*J*_HCd_ of 41 Hz associated with H^4^ reflects terminal character.

In general, the hydrides in **3**^**+**^–**5**^**+**^ (and **7**^**+**^–**9**^**+**^) sit on a continuum between terminal and bridging in character. We find computed delocalization indices and BCP ellipticities provide more effective measures of the degree of bridging character, rather than the presence (or otherwise) of a bond path.^53^ Related trends are seen in the computed IR stretches; for example, in **4**^**+**^, ν_Ir–H4_ is at 2215 cm^–1^, ν_sym_ and ν_assym_ (associated with the trans-H^1^–Ir–H^3^ unit) are at 2000 and 1791 cm^–1^, while ν_Ir–H2_ is at 1553 cm^–1^. Of these ν_assym_ has appreciable intensity and likely corresponds to the feature at 1761 cm^–1^ in the experimental spectrum (Figure S53).^54^ Increased bridging character also correlates with larger negative charges on H and increased positive charge at Zn (Figure 4a, atomic charges in italics). This implies a significant electrostatic component to Zn^δ+^···H^δ−^ bonding and an associated stabilization can give key insights when understanding reactivity trends, as discussed below.

#### Formation and Fluxionality of **4**^**+**^

**Figure 5:**
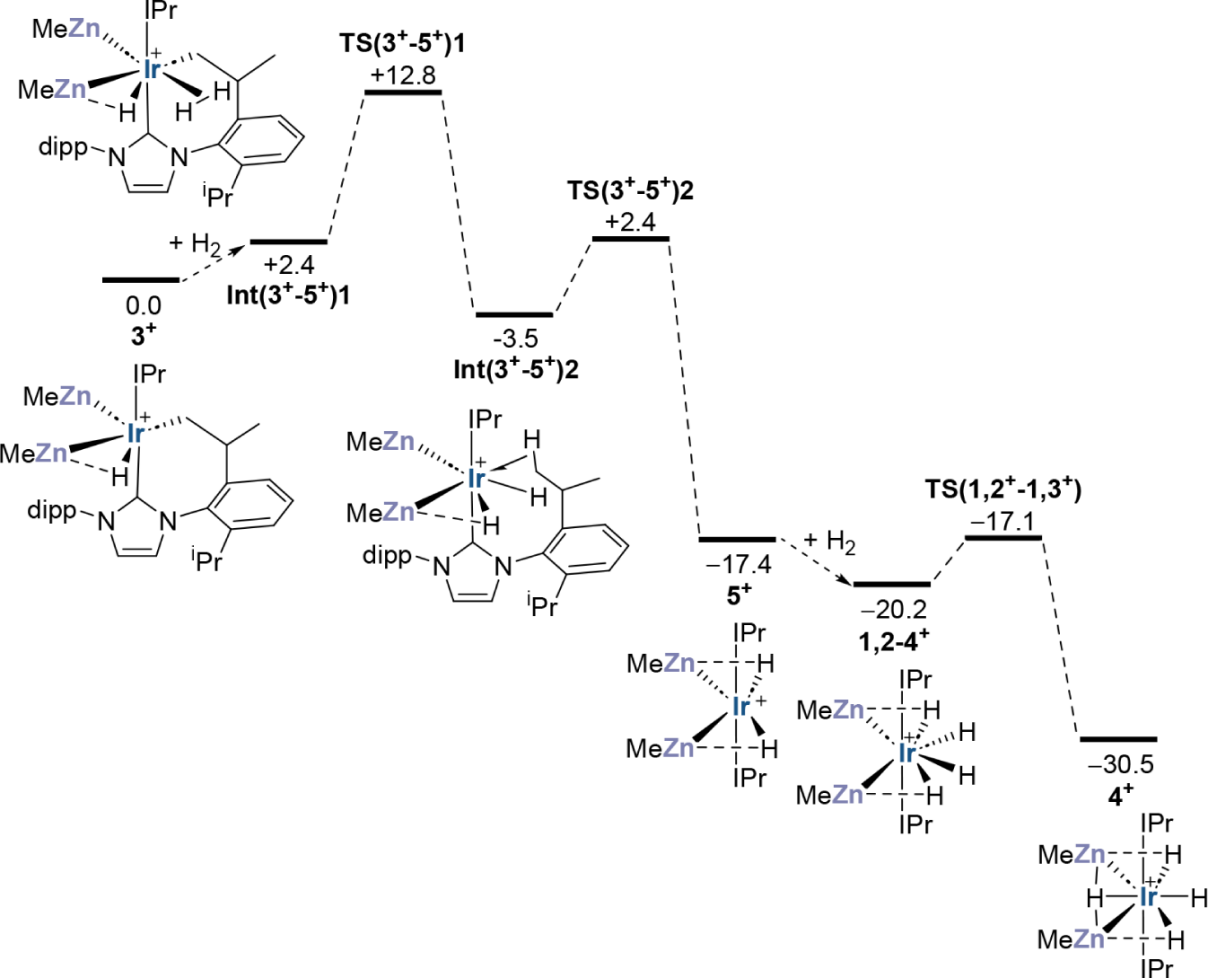
Computed free energy profile (PBE0-D3(PCM = C_6_H_5_F)/Def2-TZVP)//BP86-D3/SDD(Ir,Zn), 6-31G**; kcal/mol) for the reaction of **3**^**+**^ with H_2_ to give **4**^**+**^.

**Figure 6:**
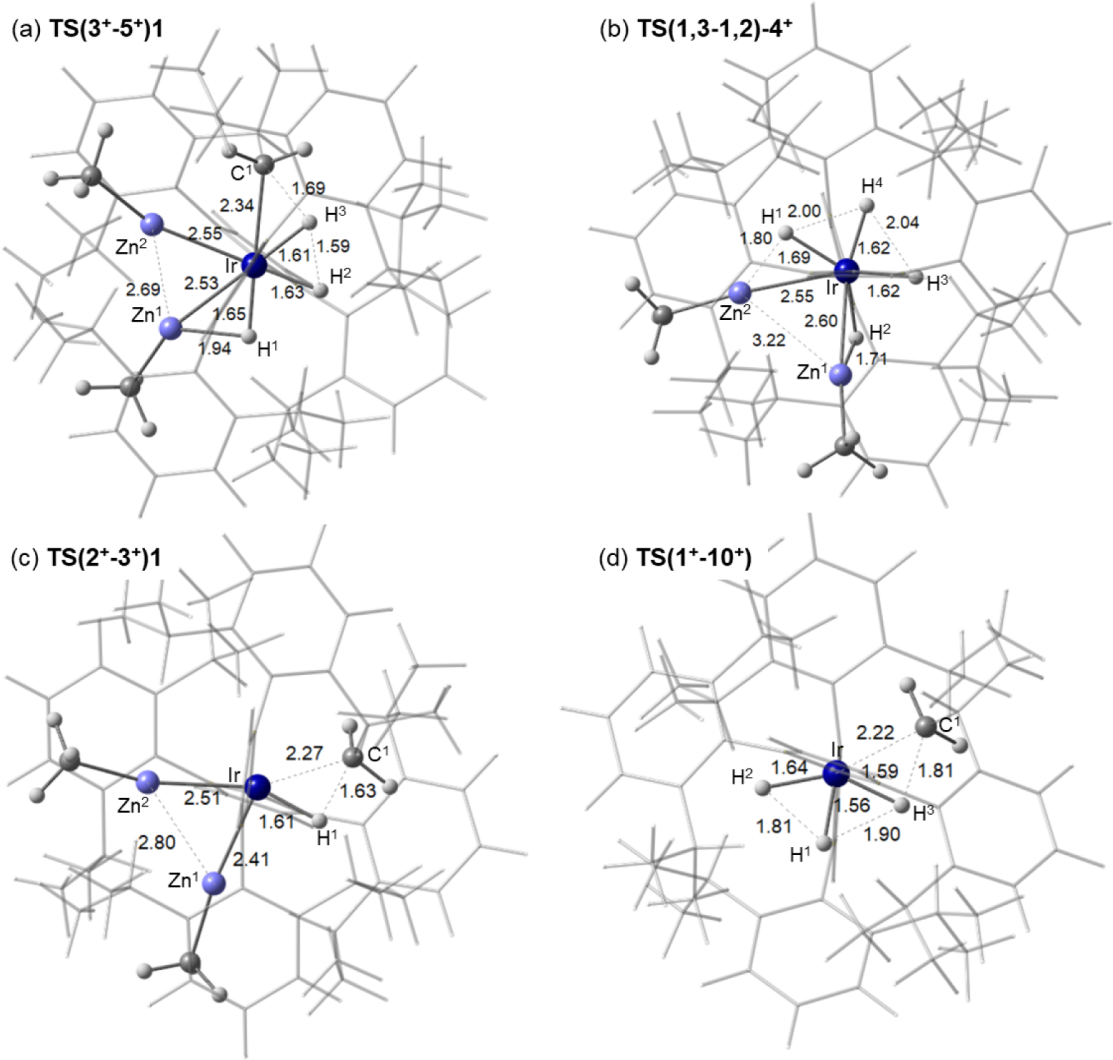
Computed geometries for key transition states from across the reactivity studies shown in Figures 5, 7 and 8. Selected distances (Å) are highlighted between participating atoms shown in ball and stick mode with spectator NHC ligands depicted as wireframe.

For reactivity studies the electronic energies from the BP86-D3 optimizations were recomputed with the PBE0 functional using a larger def2-tzvp basis set with corrections for fluorobenzene solvent and dispersion. This protocol correctly reproduces the greater stability of **3**^**+**^ over **2**^**+**^ seen in solution (see Table S3 for functional testing). The computed profile for the reaction of cyclometalated **3**^**+**^ with H_2_ to form tetrahydride **4**^**+**^ is shown in Figure 5. Initial H_2_ addition gives an η^2^-H_2_ intermediate, **Int(3**^**+**^**–5**^**+**^**)1**, (*G* = +2.4 kcal/mol) from which H_2_ cleavage via σ-CAM^55,56^**TS(3**^**+**^**–5**^**+**^**)1** (*G* = +12.8 kcal/mol, see Figure 6a for structural details) transfers one H onto the cyclometalated arm to form an agostic interaction. This gives **Int(3**^**+**^**–5**^**+**^**)2** (*G* = −3.5 kcal/mol) with cis hydrides that isomerizes to the trans-dihydride isomer **5**^**+**^ at −17.4 kcal/mol. Barrierless H_2_ addition leads to **1,2–4**^**+**^, an isomer of **4**^**+**^ with adjacent ZnMe groups that readily isomerizes to **4**^**+**^ at −30.5 kcal/mol. The initial H_2_ addition step is significant, as an alternative pathway via direct C–H coupling in **3**^**+**^ to form **2**^**+**^, which could then add H_2_, has a higher overall barrier of 20.3 kcal/mol (see also Figure 8 below); it is also consistent with the formation of [Ir(IPr)(IPr-*d*)(ZnMe)_2_HD_3_]^+^ (**4-*****d***_**4**_) seen experimentally with D_2_;^32^ reaction of **2**^**+**^ with D_2_ would instead generate [Ir(IPr)_2_(ZnMe)_2_D_4_]^+^. The lower energy of **5**^**+**^, 13.9 kcal/mol below **Int(3**^**+**^**–5**^**+**^**)2**, reflects the stability gain due to the presence of two versus one adjacent H^δ−^···Zn^δ+^ interaction.

**Figure 7:**
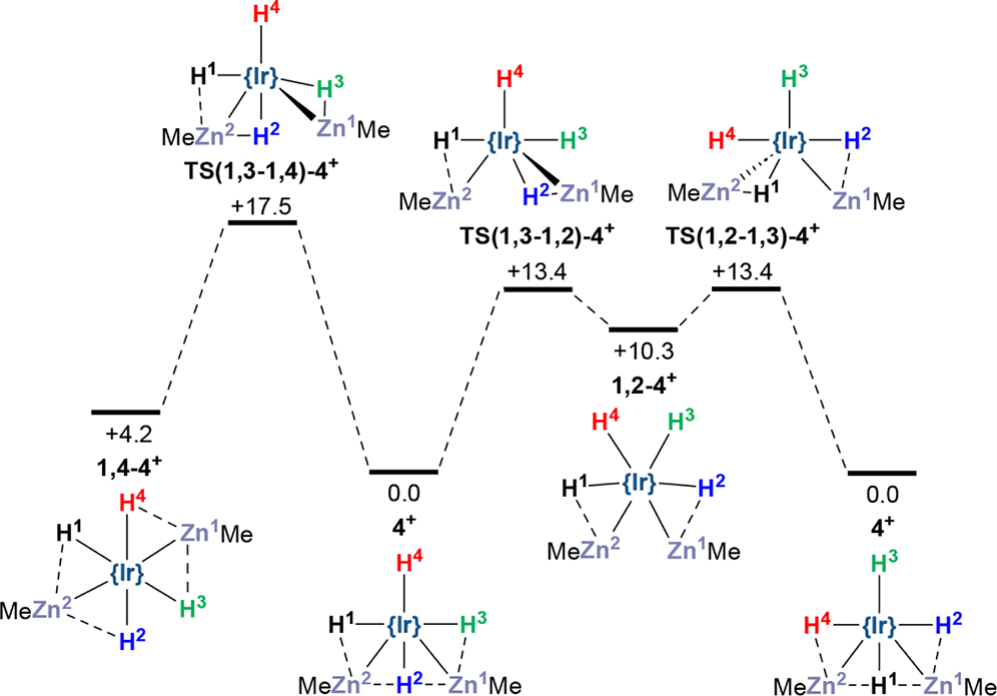
Computed reaction profile (PBE0-D3(C_6_H_5_F, def2-tzvp)//BP86-D3(SDD(Ir,Zn), 6-31g**; kcal/mol) for isomerization and hydride exchange in **4**^**+**^. Axial IPr ligands are omitted for clarity; see Figure S66 for alternative, higher energy H/H exchange processes.

Hydride fluxionality in **4**^**+**^ was investigated by considering possible isomers of this species. The observed structure, with one hydride between the ZnMe ligands, corresponds to the 1,3-isomer and lies 10.3 and 4.2 kcal/mol below the 1,2- and 1,4-isomers respectively (Figure 7). These higher energy isomers can be accessed from **4**^**+**^ via transition states at +13.4 kcal/mol and +17.5 kcal/mol. H/ZnMe exchange proceeds via movement of the ZnMe ligand out of the equatorial plane to allow the adjacent hydride to pass over the Ir–Zn vector (Figure 6b). Exchange of all four hydrides in **4**^**+**^ can be rationalized through the reversible formation of the 1,2-isomer: exchange of H^2^ with Zn^1^ Me, followed by exchange of H^1^ with Zn^2^ Me results in a net rotation of all four hydride ligands. Repeating this process ultimately renders all four hydrides equivalent with a low barrier of 13.4 kcal/mol,^57,58^ consistent with the exchange observed experimentally at 223 K.

### Interrogating ZnMe and H Isolobality

#### Structural Comparison of **1**^**+**^ and **2**^**+**^

**Figure 8:**
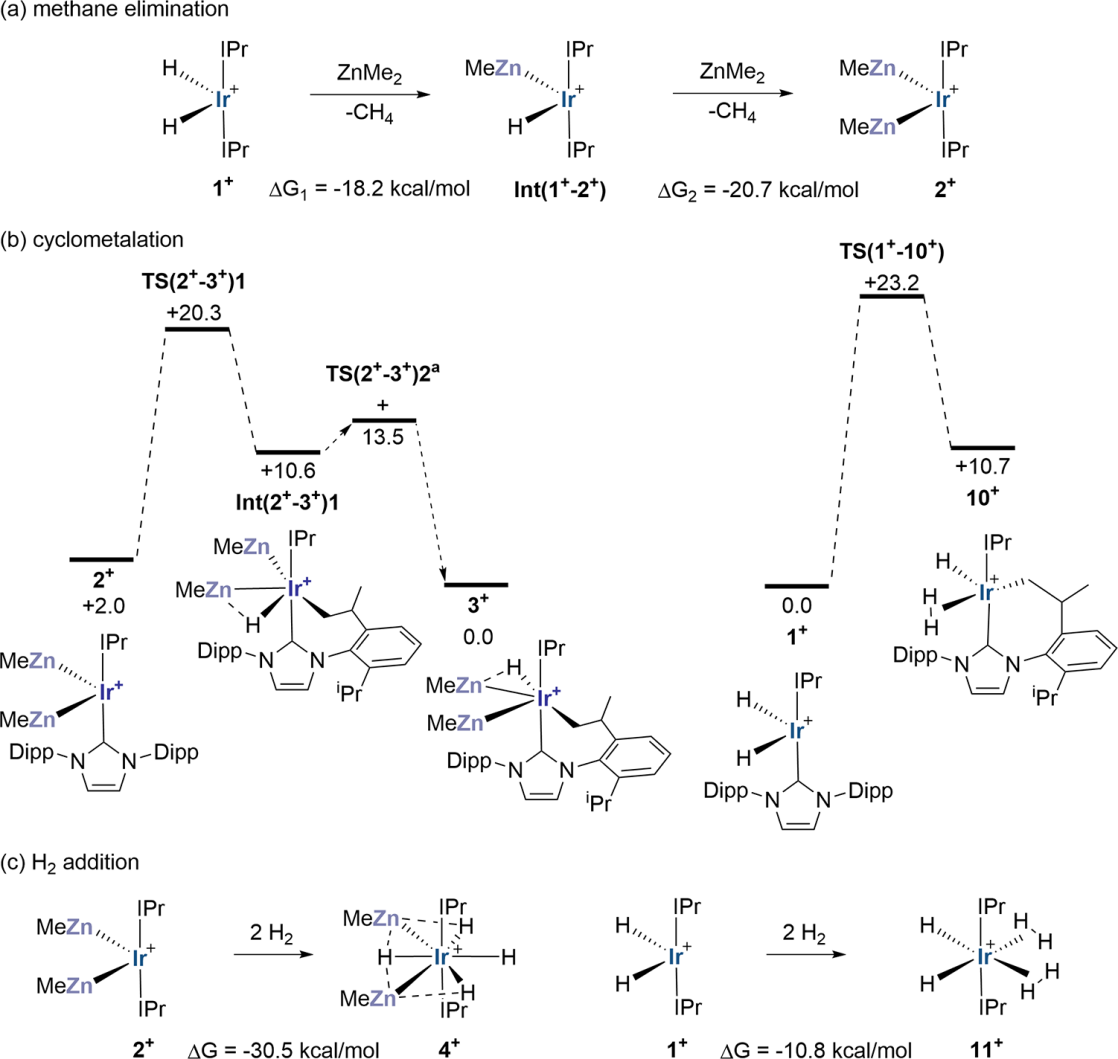
Interrogating H/ZnR isolobality in [Ir(IPr)_2_H_2_]^+^, **1**^**+**^, and [Ir(IPr)_2_(ZnMe)_2_]^+^, **2**^**+**^: (a) thermodynamics of methane elimination, (b) mechanisms of cyclometalation and (c) thermodynamics of H_2_ addition. All free energies in kcal/mol computed at the PBE0-D3 (PCM = C_6_H_5_F/Def2-TZVP//BP86-D3/SDD(Ir,Zn), 6-31g** level. ^a^Isomerization of **Int(2**^**+**^**–3**^**+**^**)1** to **3**^**+**^ involves two H/ZnMe exchange steps that place H trans to the cyclometalated arm followed by rotation of the IPr ligand. Only the highest transition state along this pathway (corresponding to IPr rotation) is indicated (see Figure S68 for full details).

Several observations are consistent with H/ZnR isolobality in these 4-coordinate cations. Both exhibit bent structures with similar calculated H–Ir–H and Zn–Ir–Zn angles around 81° (Figure 4a,b). This reflects enhanced Ir–H and Ir–ZnMe bonding upon distortion from square-planar and is consistent with a formal *d*^6^ electron count.^57,59−62^ The agostic interactions present in **1**^**+**^ are not responsible for this distortion, as the optimized structure of a [Ir(IMe_2_)(H)_2_]^+^ model system (where no equivalent agostic interaction is possible) maintains a small H–Ir–H angle of 91.8°. The lack of agostic interactions in **2**^**+**^ is unusual^57,59−62^ and implies a more electron-rich metal center^63^ and this is reflected in the computed QTAIM charges (*q*_Ir_ = +0.41 in **1**^**+**^ and −0.17 in **2**^**+**^, Figure 4a,b). The similar thermodynamics computed for the sequential methane elimination reactions that take **1**^**+**^ to **2**^**+**^ via the mixed species **Int(1**^**+**^**–2**^**+**^**)** (Figure 8a: Δ*G*_1_ = −18.2 kcal/mol; Δ*G*_2_ = −20.7 kcal/mol) indicate that replacing a terminal H with ZnR has little effect on this process.

#### Reactivity of **1**^**+**^ vs **2**^**+**^

**Scheme 3:**
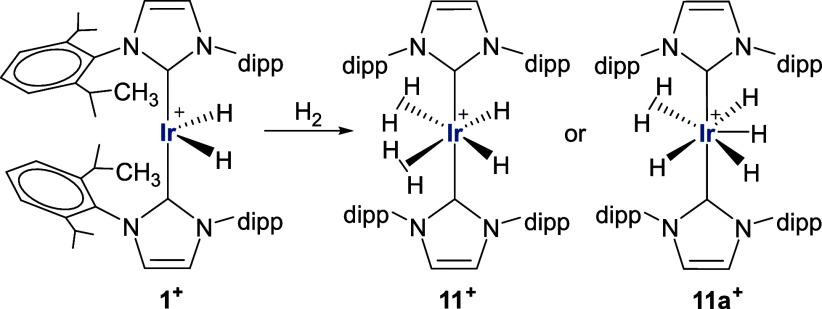
Possible Structures for **11**^**+**^ and **11a**^**+**^ from DFT Calculations, Formed upon H_2_ Addition to **1**^**+**^

Limitations in H/ZnR isolobality begin to be seen when modeling reactivity. Whereas **2**^**+**^ is only 2 kcal/mol higher in energy than its cyclometalated isomer **3**^**+**^, **1**^**+**^ is 10.7 kcal/mol more stable than cyclometalated [Ir(IPr)(IPr′)(η^2^-H_2_)H]^+^, **10**^**+**^ (Figure 8b). This reflects the Zn^δ+^···H^δ−^ interaction present in **3**^**+**^, which confers additional stability compared to the η^2^-H_2_ ligand in **10**^**+**^. This effect is also seen in the cyclometalation mechanism computed for **2**^**+**^, where the lowest energy C–H activation transition state, **TS(2**^**+**^**–3**^**+**^**)1** (*G* = +20.3 kcal/mol) places a hydride cis to Zn in **Int(2**^**+**^**–3**^**+**^**)1** (G = +10.6 kcal/mol, Figure 6c). Isomerization to **3**^**+**^ is easy, but involves a series of low energy processes (H/ZnMe exchange and IPr ligand rotation) the highest of which is via **TS(2**^**+**^**–3**^**+**^**)2** at +13.5 kcal/mol. This is also consistent with the reactivity seen in the solid-state, where both **2** and **3** are present, and both react with H_2_ to form **4**. An alternative pathway with the cyclometalated arm adjacent to Zn has a higher barrier of 28.4 kcal/mol and gives an intermediate at +19.0 kcal/mol (Figure S67). A bridging Zn^δ+^···H^δ+^ unit therefore has a significant impact on stability and, in this context, is very different to an η^2^-H_2_ ligand. This last point should also impact the energetics of H_2_ addition at **1**^**+**^ and **2**^**+**^. H_2_ addition at **2**^**+**^ to give **4**^**+**^ is highly exergonic (Δ*G*^calc^ = −30.5 kcal/mol, Figure 8c), and this is consistent with the difficulty of removing H_2_ from this species experimentally. In contrast H_2_ addition at **1**^**+**^ is computed to be much less favorable and forms [Ir(IPr)_2_(η^2^-H_2_)_2_H_2_]^+^, **11**^**+**^, at −10.8 kcal/mol via **TS(1**^**+**^**–10**^**+**^**)** at +23.2 kcal/mol, see also Figure 6d). This bis-dihydrogen dihydride is 8.5 kcal/mol more stable than the dihydrogen tetrahydride isomer [Ir(IPr)_2_(η^2^-H_2_)H_4_]^+^, **11a**^**+**^, with a minimal barrier (relative to **11a**^**+**^) for their interconversion (Scheme 3 and Figure S70).

To validate this point, we returned to experiment to study this H_2_ addition process. Exposure of **1** to H_2_ did lead to the addition of two molecules of H_2_ to give [Ir(IPr)_2_H_6_][BAr^F^_4_],^65,66^ which was found to be highly fluxional (Figure S40) exhibiting a single ^1^H NMR hydride resonance (δ −7, relative integral of 6) at room temperature which remained invariant down to 188 K,^67^ irrespective of the presence of a H_2_ atmosphere or vacuum.^68^ The temperature-independence of the hydride resonance means that the formulation of [Ir(IPr)_2_H_6_][BAr^F^_4_] cannot be determined on this basis. This contrasts with the phosphine analogues [Ir(PR_3_)_2_H_6_]^+^ (PR_3_ = PCy_3_, P^t^Bu_2_Ph), which were identified as [Ir(PR_3_)_2_(η^2^-H_2_)_2_H_2_]^+^ species based on the 1:2 ratio of hydride/dihydrogen resonances which emerge at low temperature.^69,70^ We could not crystallize [Ir(IPr)_2_H_6_][BAr^F^_4_] as it was found to partially convert back to **1** when evaporated to a solid. H_2_ addition to **1** is therefore reversible, consistent with the low binding energy computed above.

Long *T*_1_ values were measured for the hydride resonance of [Ir(IPr)_2_H_6_][BAr^F^_4_] (500 MHz, THF-*d*_8_, recorded under 1 atm H_2_) across a range of temperatures (328 ms (268 K), 306 ms (248 K), 317 ms (228 K), 360 ms (208 K)), suggesting a *T*_1_ (min) value of ca. 300 ms. There was no effect upon changing from a H_2_ atmosphere to vacuum^72^ or upon changing solvent to CD_2_Cl_2_.^71^ Although such high values might appear surprising given the computational preference for a bis-dihydrogen dihydride formulation, **11**^**+**^, the validity of using *T*_1_ times to assign structures,^73^ especially in very fluxional polyhydride systems, is known to be problematic.^74^

## Conclusions

The isolobal analogy between H and ZnMe (and by extension CdMe) holds well when these are present as terminal ligands. This is reflected in the isostructural bent structures of the **1**^**+**^ and **2**^**+**^ cations and in reactivity where only terminal character is involved (e.g., alkane elimination). However, for bridging hydrides, the electrostatic contribution to the Zn^δ+^···H^δ−^ interaction renders this unit very different to its nominally isolobal H···H analogue. As a result, reversible H_2_ addition to **1** to form **11** contrasts with irreversible H_2_ addition to **3** to form **4**. Mechanisms that feature Zn^δ+^···H^δ−^ interactions in transition states or reactive intermediates are significantly favored kinetically over alternatives where these are lacking.

**Figure 9:**
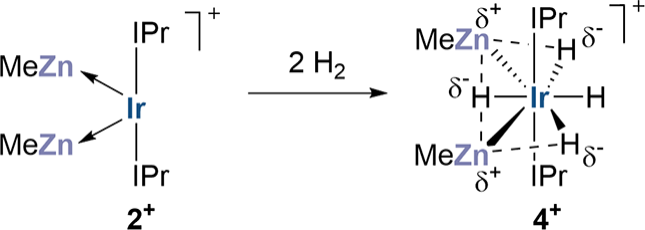
Dual role of {ZnMe}^+^ in stabilizing both **2**^+^, through direct Ir → Zn interaction, and **4**^+^, through Zn^δ+^···H^δ−^ interactions.

Previously we have highlighted the ability of the {ZnMe}^+^ moiety to promote C–H reductive elimination in cyclometalated RuZn species,^14^ and we^11^ and others^75^ have noted the ability of the formally Z-type {ZnMe}^+^ ligand to stabilize low oxidation states. This may appear at odds with the favorable H_2_ activation (“oxidative addition”) at **2**^+^ to form **4**^+^ reported here. However, this can be resolved by the dual role played by ZnMe in these two structures (Figure 9). In **2**^**+**^, the electron-rich Ir center is stabilized by two *Z*-type ZnMe ligands directly interacting with the metal center. In contrast, in **4**^**+**^ the {ZnMe}^+^ moiety interacts primarily with the hydride ligands with a strong electrostatic contribution that confers stability.^76^ In this **4** resembles an “ate” complex tending toward an outer-sphere ion-pair.^77^

In summary, we report here the targeted synthesis of the heterotrimetallic complex [Ir(IPr)_2_(ZnMe)_2_][BAr^F^_4_], **2**, via the alkane elimination reaction of [Ir(IPr)_2_H_2_][BAr^F^_4_], **1**, with ZnMe_2_. Unexpectedly, the cyclometalated isomer **3** is formed in solution, although both **2** and **3** were characterized in the solid state. **3** reacts as a functional equivalent of **2** and readily adds H_2_ to form tetrahydride [Ir(IPr)_2_(ZnMe)_2_H_4_][BAr^F^_4_], **4**, that under forcing conditions can release H_2_ to form dihydride [Ir(IPr)_2_(ZnMe)_2_H_2_][BAr^F^_4_], **5**. Crystallographic and computational studies characterize direct Ir–Zn bonding in **2**–**5** with no evidence for any Zn···Zn interactions. The hydride ligands in **3**–**5** sit on a continuum between terminal and bridging character. Greater bridging character weakens Ir–Zn bonding and induces a higher degree of hydridic character that is stabilized by electrostatic interactions with adjacent Zn^δ+^ centers. The geometries and electronic structures of the Cd analogues (**6**–**9**) mirror those of their Zn congeners (**2**–**5**). Terminal H and ZnMe ligands have similar effects on structure and reactivity and so could be considered isolobal. However, the electrostatic component to bonding in a bridging Zn^δ+^···H^δ−^ unit renders this very different to a H···H moiety. Ultimately this reflects differences in electronegativity that mean the “not identical, but similar”^78,79^ frontier orbital energy criterion for isolobality begins to break down. These insights and how they may promote H–H and other E–H bond activations can be fed into the design of new TM–M′ heterometallics for novel synthesis and catalysis.

## Supporting Information Available

Experimental procedures, characterization data, NMR/IR spectra, computational details, computed geometries (PDF)
