# Supplementary material for: Isolobal Cationic Iridium Dihydride and Dizinc Complexes: A Dual Role for the ZnR Ligand Enhances H2 Activation
Source: Inorg Chem. 2024 Nov 20;63(48):22944–54. doi: 10.1021/acs.inorgchem.4c04058 (PMC11615952; doi:10.1021/acs.inorgchem.4c04058)
Supplement: Supplementary file 1 — ic4c04058_si_001.pdf [file ic4c04058_si_001.pdf]

SUPPORTING INFORMATION

Isolobal Cationic Iridium Dihydride and Dizinc Complexes: A Dual Role for the ZnR Ligand Enhances H_2 Activation

Amber M. Walsh,[a] Lia Sotorrios,[b] Rebecca G. Cameron,[b] Anne-Frédérique Pécharman,[a] Barbara Procacci,[c] John P. Lowe,[a] Stuart A. Macgregor,[d*] Mary F. Mahon,[a*] Neil T. Hunt[c] and Michael K. Whittlesey[a*]

[a] *Department of Chemistry, University of Bath, Bath, BA2 7AY, United Kingdom*

[b] *Institute of Chemical Sciences, School of Engineering and Physical Sciences, Heriot-Watt University, Edinburgh EH14 4AS, United Kingdom*

[c] *Department of Chemistry and York Biomedical Research Institute, University of York, York YO10 5DD, United Kingdom*

[d] *EaStCHEM School of Chemistry, University of St Andrews, North Haugh, St Andrews KY16 9ST, United Kingdom*

Table of Contents

S1. Experimental.......... S2

S1.1. Experimental Details and Characterization Data.......... S2

S1.2. NMR and IR Spectra.......... S15

S2. Computational Studies.......... S74

S2.1. Computational Details.......... S74

S2.2. Functional Testing.......... S75

S2.3. QTAIM Studies.......... S77

S2.3. Additional Computed Reaction Profiles.......... S82

S2.4. Computed Structures and Energies.......... S87

S3. References.......... S142

S1. Experimental

S1.1 Experimental Details and Characterization Data

General Comments. All manipulations were carried out under argon using standard Schlenk, high vacuum and glovebox techniques using dry and degassed solvents. CD_2Cl_2 and C_6D_5F were vacuum transferred from CaH_2 and THF-d_8 from potassium or NaK. NMR spectra were recorded on Bruker Avance 400 and 500 MHz NMR spectrometers and referenced as follows: CD_2Cl_2 (^{1}H, δ 5.32; ^{13}C, δ 54.0), C_6D_5F (^{1}H, δ 7.11; ^{13}C, δ 163.0) and THF-d_8 (^{1}H, δ 3.58; ^{13}C, δ 67.6). ^{1}H NMR spectra recorded unlocked in C_6H_5F were referenced to the most upfield shifted multiplet set to δ 7.11. ^{2}H NMR spectra were recorded in THF-h_8 and referenced to THF-d_1 set as δ 3.62 (i.e. value of THF-h_8 in THF-d_8).[1] ^{113}Cd{^{1}H} NMR spectra (referenced to the proton resonance of TMS according to the unified scale) were recorded with inverse-gated decoupling. IR spectra were recorded on a Bruker ALPHA ATR-IR spectrometer inside a glovebox. Electrospray ionization (ESI) mass spectrometry was performed on a Bruker MaXis HD ESI-QTOF mass spectrometer. Elemental analyses were performed by Elemental Microanalysis Ltd, Okehampton, Devon, U.K. $ZnMe_2$ (1.2 M solution in toluene), H_2, D_2 (99%) and CO were used as received. $NaBAr^F_4$ was prepared according to the literature.[2] $CdMe_2$ (2.4 M in toluene) was kindly provided by Dr Andrew Johnson (C*AUTION: Both dimethyl zinc*[3] *and dimethyl cadmium*[4] *are extremely hazardous and should be handled with utmost care employing appropriate safety measures*).

[Ir(IPr)$_2$H$_2$][BAr$^F_4$] (1). The dihydride salt **1** was prepared according to the method reported by Aldridge[5] by Na[BAr$^F_4$] abstraction of the chloride ligand from [Ir(IPr)$_2$H$_2$Cl].[5] We found **1** to be only partially soluble in C_6D_6,[6] but far more soluble in THF-d_8, CD_2Cl_2 and C_6H_5F, in which it was characterized by ^{1}H NMR spectroscopy. Figures S1 and S2 show

spectra in THF-d_8 and CD_2Cl_2. Some degradation to [IPrH][$BAr^F{}_4$] was apparent in THF-d_8 over days, while new, unknown resonances appeared more rapidly (ca. 12 h) in CD_2Cl_2.

[Ir(IPr)(IPr')(ZnMe)$_2$H][BAr$^F{}_4$] (3). A solution of [Ir(IPr)$_2$H$_2$][BAr$^F{}_4$] (**1**; 200 mg, 0.11 mmol) in C_6H_5F (2 mL) was treated with $ZnMe_2$ (0.18 mL of 1.2 M toluene solution, 0.22 mmol) and stirred at room temperature for 10 min. The resulting solution was reduced to dryness, the residue redissolved in C_6H_5F and layered with hexane to yield orange crystals of **3**. Yield: 147 mg (68%). ^{1}H NMR (THF-d_8, 400 MHz, 219 K): δ 7.90-7.84 (br m, 9H, BAr$^F{}_4$ + NC*H*=CHN), 7.83 (d, $^3J_{HH}$ = 1.7 Hz, 1H, NC*H*=CHN), 7.82 (d, $^3J_{HH}$ = 1.7 Hz, 1H, NC*H*=CHN), 7.71-7.66 (br m, 5H, BAr$^F{}_4$ + NC*H*=CHN), 7.64-7.40 (m, 10H, Ar), 7.39-7.28 (m, 3H, Ar), 2.78 (m, 2H, C*H*Me$_2$), 2.64 (sept, $^3J_{HH}$ = 6.8 Hz, 1H, C*H*Me$_2$), 2.52 (br t, J_{HH} = 13.9 Hz, 1H, CH(Me)C*H*HIr), 2.44 (sept, $^3J_{HH}$ = 6.6 Hz, 1H, C*H*Me$_2$), 2.25 (br m, 1H, C*H*-CH$_2$), 2.12 (sept, $^3J_{HH}$ = 6.7 Hz, 1H, C*H*Me$_2$), 2.00 (br m, 1H, C*H*Me$_2$), 1.89 (sept, $^3J_{HH}$ = 6.3 Hz, 1H, C*H*Me$_2$), 1.58 (d, $^3J_{HH}$ = 6.7 Hz, 3H, CH*Me*$_2$), 1.34 (d, $^3J_{HH}$ = 6.6 Hz, 3H, CH*Me*$_2$), 1.27 (d, $^3J_{HH}$ = 6.9 Hz, 3H, CH*Me*$_2$), 1.25 (d, $^3J_{HH}$ = 6.7 Hz, 3H, CH*Me*$_2$), 1.19-0.87 (m, 31H, CH*Me*$_2$ + CH(Me)CH*H*Ir), 0.53 (d, $^3J_{HH}$ = 6.7 Hz, 3H, CH*Me*$_2$), -0.94 (s, 3H, Zn*Me*), -0.95 (s, 3H, Zn*Me*), -4.14 (s, 1H, Ir*H*). ^{1}H NMR (CD_2Cl_2, 400 MHz, 222 K): δ 7.71 (s, 8H, BAr$^F{}_4$), 7.54 (s, 4H, BAr$^F{}_4$), 7.52-7.48 (m, 2H, Ar), 7.41-7.12 (m, 10H, Ar), 7.08 (d, $^3J_{HH}$ = 1.7 Hz, 2H, NC*H*=CHN), 7.06 (d, $^3J_{HH}$ = 1.9 Hz, 1H, NC*H*=CHN), 7.01 (d, $^3J_{HH}$ = 1.8 Hz, 1H, NC*H*=CHN), 2.68 (m, 2H, C*H*Me$_2$), 2.52 (sept, $^3J_{HH}$ = 6.9 Hz, 1H, C*H*Me$_2$), 2.34 (br t, J_{HH} = 14.2 Hz, 1H, CH(Me)C*H*HIr), 2.31 (sept, $^3J_{HH}$ = 6.1 Hz, 1H, C*H*Me$_2$), 2.12 (br m, 1H, C*H*-CH$_2$), 1.97 (sept, $^3J_{HH}$ = 6.5 Hz, 1H, C*H*Me$_2$), 1.86 (sept, $^3J_{HH}$ = 6.5 Hz, 1H, C*H*Me$_2$), 1.76 (sept, $^3J_{HH}$ = 6.9 Hz, 1H, C*H*Me$_2$), 1.46 (d, $^3J_{HH}$ = 6.8 Hz, 3H, CH*Me*$_2$), 1.19-1.14 (m, 9H, CH*Me*$_2$), 1.06-0.82 (m, 28H, CH*Me*$_2$ + CH(Me)CH*H*Ir), 0.37 (d, $^3J_{HH}$ = 6.3 Hz, 3H, CH*Me*$_2$), -1.04 (s, 3H, Zn*Me*), -1.05 (s, 3H, Zn*Me*), -4.24 (s, 1H, Ir*H*). ^{13}C{^{1}H} DEPTQ (THF-d_8, 126 MHz, 219 K): δ 173.1 (s, IrC_{NHC}), 173.0 (s, IrC_{NHC}), 163.1 (1:1:1:1 quart, $^1J_{CB}$

= 50 Hz, BAr^F_4), 147.7 (s, C_{ipso}), 147.5 (s, C_{ipso}), 146.7 (s, C_{ipso}), 146.6 (s, C_{ipso}), 146.4 (s, C_{ipso}), 146.3 (s, C_{ipso}), 144.9 (s, C_{ipso}), 144.5 (s, C_{ipso}), 139.0 (s, C_{ipso}), 137.2 (s, C_{ipso}), 135.6 (br s, BAr^F_4), 135.0 (s, C_{ipso}), 132.6 (s, *C*H), 132.0 (s, *C*H), 131.3 (s, *C*H), 131.1 (s, *C*H), 130.1 (br quart, $^2J_{CF}$ = 31 Hz, BAr^F_4), 128.7 (s, *C*H), 128.0 (s, *C*H=CHN), 127.4 (s, *C*H), 127.0 (s, C_{ipso}), 126.7 (2 x s, N*C*H=CHN), 126.0 (s, *C*H), 125.9 (s, *C*H), 125.7 (s, *C*H), 125.6 (br quart, $^1J_{CF}$ = 273 Hz, BAr_4^F), 125.5 (s, *C*H), 124.8 (s, *C*H), 124.6 (s, *C*H), 118.0 (br m, BAr^F_4), 40.5 (s, *C*HCH_2Ir), 39.5 (s, CH*C*H_2Ir), 30.5 (s, *C*HMe_2), 30.3 (s, *C*HMe_2), 30.2 (s, *C*HMe_2), 30.1 (s, *C*HMe_2), 30.0 (s, *C*HMe_2), 29.9 (s, *C*HMe_2), 29.7 (s, *C*HMe_2), 29.3 (s, CHMe_2), 27.3 (s, CHMe_2), 27.1 (s, CHMe_2), 27.0 (s, CHMe_2), 26.2 (s, CHMe_2), 26.1 (s, CHMe_2), 26.0 (s, CHMe_2), 24.8 (s, CHMe_2), 24.3 (s, CHMe_2), 23.6 (s, CHMe_2), 23.2 (s, CHMe_2), 22.9 (s, CHMe_2), 22.8 (s, CHMe_2), 22.5 (s, CHMe_2), -1.6 (s, Zn*Me*), -8.9 (s, Zn*Me*). ATR-IR (ν, cm^{-1}): 1792 (w, Ir-H). Anal. Calcd for $C_{88}H_{90}BF_{24}IrN_4Zn_2$ (1993.4): C, 53.02; H, 4.55; N 2.81. Found: C, 53.07; H, 4.39; N 2.79. ESI-TOF MS: $[M]^+$ m/z = 1127.3949 (theoretical m/z = 1127.4433).

[Ir(IPr)$_2$(ZnMe)$_2$H$_4$][BAr$^F_4$] (4). A J. Young's resealable ampule was charged with a C_6H_5F (1 mL) solution of **3** (80 mg, 0.040 mmol), degassed via three freeze-pump-thaw cycles and placed under 1 atm H_2, leading to a rapid color change from red-orange to colorless. After 5 min shaking, the solution was concentrated and layered with hexane to yield colorless crystals of **4**. Yield: 48 mg (60 %). ^{1}H NMR (CD_2Cl_2, 400 MHz, 201 K): δ 7.71 (br s, 8H, BAr^F_4), 7.59 (t, $^3J_{HH}$ = 7.8 Hz, 2H, Ar), 7.53 (br s, 4H, BAr^F_4), 7.44-7.31 (m, 8H, Ar), 7.18 (d, $^3J_{HH}$ = 7.9 Hz, 1H, Ar), 7.13 (d, $^3J_{HH}$ = 7.8 Hz, 1H, Ar), 7.05 (t, $^3J_{HH}$ = 8.7 Hz, 1H, Ar), 7.01 (s, 4H, NC*H*=CHN), 2.28 (sept, $^3J_{HH}$ = 6.6 Hz, 2H, C*H*Me_2) 2.23 (sept, $^3J_{HH}$ = 6.6 Hz, 2H, C*H*Me_2), 1.73 (sept, $^3J_{HH}$ = 6.3 Hz, 2H, C*H*Me_2), 1.61 (sept, $^3J_{HH}$ = 6.3 Hz, 2H, C*H*Me_2), 1.24-1.04 (m 18H, C*H*Me_2), 0.97 (d, $^3J_{HH}$ = 6.6 Hz, 12H, CHMe_2), 0.83 (d, $^3J_{HH}$ = 6.7 Hz, 6H, CHMe_2), 0.77 (d, $^3J_{HH}$ = 6.7 Hz, 6H, CHMe_2), 0.58 (d, $^3J_{HH}$ = 6.4 Hz, 6H,

CHMe_2), -1.08 (s, 6H, ZnMe), -9.62 (s, 1H, IrH; T_1 (400 MHz, 223 K) 465 ms), -10.44 (s, 1H, IrH; T_1 (400 MHz, 223 K) 316 ms), -11.82 (s, 2H, IrH; T_1 (400 MHz, 223 K) 388 ms). $^{13}C\{^1H\}$ DEPTQ NMR (CD_2Cl_2, 101 MHz, 201 K): δ 161.5 (1:1:1:1 quart, $^1J_{CB}$ = 49 Hz, BAr^F_4), 155.4 (s, IrC_{NHC}),# 145.3 (s, C_{ipso}), 145.2 (s, C_{ipso}), 144.6 (s, C_{ipso}), 142.6 (s, C_{ipso}), 137.1 (s, C_{ipso}), 136.6 (s, C_{ipso}), 134.5 (br s, BAr^F_4), 132.5 (s, CH), 130.0 (s, CH), 128.4 (br quart, $^2J_{CF}$ = 30 Hz, BAr^F_4), 127.0 (s, CH), 126.2 (s, CH), 125.2 (s, CH), 125.1 (s, CH), 124.3 (quart, $^1J_{CF}$ = 270 Hz, BAr_4^F), 123.8 (s, CH), 123.7 (s, CH), 117.4 (br s, BAr^F_4), 115.2 (s, CH), 115.1 (s, CH), 29.2 (s, CHMe$_2$), 29.1 (s, CHMe$_2$), 28.6 (s, CHMe$_2$), 28.5 (s, CHMe$_2$), 26.3 (s, CHMe_2), 25.6 (s, CHMe_2), 25.4 (s, CHMe_2), 25.3 (s, CHMe_2), 22.1 (s, CHMe_2), 21.9 (s, CHMe_2), 21.8 (s, CHMe_2), 2.7 (s, ZnMe) (# split into quintet ($^2J_{CH}$ = 4 Hz) in $^{13}C\{selective\text{-}^1H\}$ NMR spectrum (THF-d_8, 126 MHz, 278 K)). ATR-IR (ν, cm^{-1}): 1761 (w, Ir-H). Anal. Calcd for $C_{88}H_{94}BF_{24}IrN_4Zn_2$ (1997.5): C, 52.91; H, 4.74; N 2.80. Found: C, 52.82; H, 4.56; N 2.76. ESI-TOF MS: $[M]^+$ m/z = 1131.3983 (theoretical m/z = 1131.4746).

Reaction of 3 with D_2. A J. Young's resealable NMR tube was charged with a C_6H_5F (0.5 mL) solution of **3** (10 mg, 0.005 mmol), degassed via three freeze-pump-thaw cycles and placed under 1 atm D_2. The solution, which immediately changed color from red-orange to colorless, was then monitored periodically by NMR spectroscopy.

H/D Exchange in 4. A J. Young's resealable NMR tube was charged with a $[D_2]$-dichlomethane (0.5 mL) solution of **4** (10 mg, 0.005 mmol), degassed via three freeze-pump-thaw cycles, placed under 1 atm D_2 and heated at 40 ^{0}C. The extent of H/D exchange was monitored by recording ^{1}H NMR spectra of the sample in a pre-cooled NMR probe at 226 K. After a total of 3 h at 40 ^{0}C, the tube was freeze-pump-thaw degassed (3 cycles), placed under H_2, heated at 40 ^{0}C for another 3 h and then cooled again to 226 K for NMR measurements.

[Ir(IPr)$_2$(ZnMe)$_2$H$_2$][BAr$^F_4$] (5). A solid sample of **4** (40 mg, 0.020 mmol) was placed in a J Young's resealable ampule and heated at 60 ^{0}C for 14 days under dynamic vacuum, leading to a color change from colorless to orange. The residue was dissolved in C_6H_5F (0.5 mL) and layered with hexane to yield 30 mg of a mixture of orange crystals of **5** and colorless crystals of **4**. Redissolution in CD_2Cl_2 and analysis of the IrH/ZnMe integrals by ^{1}H NMR spectroscopy at 201 K showed the sample comprized ca. 78% of **5**. Selected ^{1}H NMR (CD_2Cl_2, 400 MHz, 201 K): δ 2.53 (m, 2H, C*H*Me$_2$), 2.23 (br m, 2H, C*H*Me$_2$), 2.08 (m, 2H, C*H*Me$_2$), 1.56 (br m, 2H, C*H*Me$_2$), 1.16 (m, 12H, CH*Me*$_2$), 1.02 (m, 12H, CH*Me*$_2$), 0.90 (m, 12H, CH(C*H*$_3$)$_2$), 0.77 (br d, $^3J_{HH}$ ~ 5.8 Hz, 6H, CH*Me*$_2$), 0.36 (br d, $^3J_{HH}$ ~ 5.3 Hz, 6H, CH*Me*$_2$), -0.98 (s, 6H, Zn*Me*), -4.15 (s, 2H, Ir*H*). ATR-IR (ν, cm^{-1}): 1697 (w, Ir-H).

[Ir(IPr)(IPr')(CdMe)$_2$H][BAr$^F_4$] (7). CdMe$_2$ (9.2 μL of 2.4 M toluene solution, 0.023 mmol) was added to a C_6H_5F (0.5 mL) solution of **1** (20 mg, 0.011 mmol) in a J. Youngs resealable NMR tube, resulting in a rapid color change from pale orange to deep red. After ca. 10 min, the solution was reduced to dryness, the residue redissolved in C_6H_5F and layered with hexane to dark red crystals of **7** that were spectroscopically and structurally characterized. ^{1}H NMR (THF-*d*$_8$, 400 MHz, 228 K): δ 7.86 (br m, 8H, BAr$^F_4$), 7.80 (d, $^3J_{HH}$ = 1.9 Hz, 1H, NC*H*=CHN), 7.70-7.34 (m, 16H, BAr$^F_4$ + Ar + NC*H*=CHN), 7.29 (m, 2H, Ar), 7.21-7.10 (m, 1H, Ar), 3.03 (br t, $^2J_{HH}$ = 13.3 Hz, 1H, CH(Me)C*H*HIr), 2.85-2.61 (m, 3H, C*H*Me$_2$), 2.37 (sept, $^3J_{HH}$ = 6.5 Hz, 1H, C*H*Me$_2$), 2.28 (br m, 1H, C*H*Me$_2$), 2.19 (sept, $^3J_{HH}$ = 6.7 Hz, 1H, C*H*Me$_2$), 2.03 (sept, $^3J_{HH}$ = 6.6 Hz, 1H, C*H*Me$_2$), 1.91 (br sept, $^3J_{HH}$ = 6.6 Hz, 1H, C*H*Me$_2$), 1.59 (d, $^3J_{HH}$ = 6.8 Hz, 3H, CH*Me*$_2$), 1.38-0.97 (m, 36H, CH*Me*$_2$ + CH(Me)CH*H*Ir), 0.90 (d, $^3J_{HH}$ = 6.9 Hz, 6H, CH*Me*$_2$), 0.53 (d, $^3J_{HH}$ = 6.7 Hz, 3H, CH*Me*$_2$), -0.54 (s ($^2J_{HCd}$ = 66 Hz), 3H, Cd*Me*), -0.62 (s ($^2J_{HCd}$ = 64 Hz), 3H, Cd*Me*), -3.70 (s ($^2J_{HCd}$ = 426 Hz), 1H, Ir*H*). ^{13}C{^{1}H} DEPTQ (THF-*d*$_8$, 126 MHz, 228 K): δ 172.4 (s, IrC_{NHC}), 171.9 (s, IrC_{NHC}), 163.1 (1:1:1:1 quart, $^1J_{CB}$ = 50 Hz, BAr$^F_4$), 148.4 (s, C_{ipso}), 147.8 (s, C_{ipso}), 147.6

(s, C_{ipso}), 146.5 (s, C_{ipso}), 146.3 (s, C_{ipso}), 146.0 (s, C_{ipso}), 144.7 (s, C_{ipso}), 143.9 (s, C_{ipso}), 139.6 (s, C_{ipso}), 137.5 (s, C_{ipso}), 135.9 (s, C_{ipso}), 135.6 (br s, BAr^F_4), 132.3 (s, *C*H), 131.6 (s, *C*H), 131.2 (s, *C*H), 131.0 (s, *C*H), 130.1 (br quart, $^2J_{CF}$ = 31 Hz, BAr^F_4), 128.7 (s, *C*H), 128.0 (s, *C*H), 127.7 (s, *C*H), 127.5 (s, *C*H), 126.8 (C*H*), 126.7 (s, *C*H), 125.8 (s, *C*H), 125.7 (s, *C*H), 125.6 (br quart, $^1J_{CF}$ = 273 Hz, BAr_4^F), 125.4 (s, *C*H), 124.7 (s, *C*H), 123.8 (s, *C*H), 118.5 (br m, BAr^F_4), 42.7 (s, $CH\mathit{C}H_2Ir$), 40.7 (s, $\mathit{C}HCH_2Ir$), 30.6 (s, $\mathit{C}HMe_2$), 30.4 (s, $\mathit{C}HMe_2$), 30.3 (s, $\mathit{C}HMe_2$), 30.2 (s, $\mathit{C}HMe_2$), 30.1 (s, $\mathit{C}HMe_2$), 29.9 (s, $\mathit{C}HMe_2$), 29.8 (s, $\mathit{C}HMe_2$), 29.4 (s, $\mathit{C}HMe_2$), 27.3 (s, $CH\mathit{Me}_2$), 27.2 (s, $CH\mathit{Me}_2$), 27.0 (s, $CH\mathit{Me}_2$), 26.4 (s, $CH\mathit{Me}_2$), 26.1 (s, $CH\mathit{Me}_2$), 26.0 (s, $CH\mathit{Me}_2$), 24.7 (s, $CH\mathit{Me}_2$), 24.4 (s, $CH\mathit{Me}_2$), 23.7 (s, $CH\mathit{Me}_2$), 23.2 (s, $CH\mathit{Me}_2$), 23.1 (s, $CH\mathit{Me}_2$), 22.9 (s, $CH\mathit{Me}_2$), 22.7 (s, $CH\mathit{Me}_2$), 22.0 (s, $CH\mathit{Me}_2$), 0.7 (s, Cd*Me*), -7.2 (s, Cd*Me*). $^{113}Cd\{^1H\}$ NMR (THF-d_8, 111 MHz, 228 K): δ -178.3 (s), -435.7 (s).

$[Ir(IPr)_2(CdMe)_2H_4][BAr^F_4]$ (8). A sample of **7** (ca. 5-10 mg) was dissolved in C_6H_5F (0.5 mL) in a J. Youngs resealable NMR tube, freeze-pump-thaw degassed (3 cycles) and placed under 1 atm of H_2, leading to a near instantaneous color change from red to colorless. The solution was concentrated and layered with hexane to afford a few colorless crystals of **8** that allowed structural and partial solution characterization. ^{1}H NMR (THF-d_8, 400 MHz, 228 K): δ 7.86 (br m, 8H, BAr^F_4), 7.77-7.60 (m, 12H, BAr^F_4 + Ar + NC*H*=CHN), 7.56 (m, 2H, Ar), 7.45 (t, $^3J_{HH}$ = 7.7 Hz, 2H, Ar), 7.35 (d, $^3J_{HH}$ = 7.7 Hz, 2H, Ar), 7.23 (d, $^3J_{HH}$ = 7.7 Hz, 2H, Ar), 2.37 (br m, 4H, $C\mathit{H}Me_2$), 2.00-1.81 (br m, 4H, $C\mathit{H}Me_2$), 1.40-1.28 (br m, 18H, $CH\mathit{Me}_2$), 1.07 (br d, $^3J_{HH}$ = 6.5 Hz, 6H, $CH\mathit{Me}_2$), 1.05 (br d, $^3J_{HH}$ = 6.8 Hz, 6H, $CH\mathit{Me}_2$), 0.95 br d, $^3J_{HH}$ = 6.5 Hz, 6H, $CH\mathit{Me}_2$), 0.85 (br d, $^3J_{HH}$ = 6.4 Hz, 6H, $CH\mathit{Me}_2$), 0.61 (br d, $^3J_{HH}$ = 6.3 Hz, 6H, $CH\mathit{Me}_2$), -0.65 (s ($^2J_{HCd}$ = 63 Hz), 6H, Cd*Me*), -7.95 (s ($^2J_{HCd}$ = 286 Hz), 1H, Ir*H*; T_1 (400 MHz, 228 K) 383 ms), -9.97 (s ($^2J_{HCd}$ = 41 Hz), 1H, Ir*H*; T_1 (400 MHz,

228 K) 294 ms), -10.44 (s ($^{2}J_{HCd}$ = 372 Hz), 2H, Ir*H*; T_1 (400 MHz, 228 K) 371 ms). ATR-IR (ν, cm^{-1}): 1752 (w, Ir-H).

[Ir(IPr)$_2$(CdMe)$_2$H$_2$][BAr^F$_4$] (9). A solid sample of **8** (20 mg, 0.01 mmol) was placed in a J. Young's resealable NMR tube and heated at 80 ^{0}C for 7 days under dynamic vacuum. Over this time, the color of the sample changed from colorless to red. The sample was dissolved in CD_2Cl_2 and **9** characterized by a combination of ^{1}H and ^{113}Cd NMR spectroscopy at low temperature. Selected ^{1}H NMR (CD_2Cl_2, 400 MHz, 201 K): δ 2.53 (m, 2H, C*H*Me$_2$), 2.23 (br m, 2H, C*H*Me$_2$), 2.08 (m, 2H, C*H*Me$_2$), 1.56 (br m, 2H, C*H*Me$_2$), 1.16 (m, 12H, CH*Me*$_2$), 1.02 (m, 12H, CH*Me*$_2$), 0.90 (m, 12H, CH(C*H*$_3$)$_2$), 0.77 (br d, $^{3}J_{HH}$ ~ 5.8 Hz, 6H, CH*Me*$_2$), 0.36 (br d, $^{3}J_{HH}$ ~ 5.3 Hz, 6H, CH*Me*$_2$), -0.98 (s, 6H, Zn*Me*), -4.15 (s, 2H, Ir*H*). ^{113}Cd{^{1}H} NMR (THF-*d*$_8$, 111 MHz, 228 K): δ -275.0 (s).

[Ir(IPr)$_2$H$_6$][BAr^F$_4$] (11). A J Youngs NMR tube containing a C_6H_5F (0.5 mL) solution of **1** (20 mg, 0.01 mmol) was freeze-pump-thaw degassed three times and placed under 1 atm H_2, leading to an immediate color change from yellow-orange to colorless. ^{1}H NMR spectroscopy showed full conversion to [Ir(IPr)$_2$H$_6$][BAr^F$_4$] (**11**), which was characterized spectroscopically. ^{1}H NMR (THF-*d*$_8$, 400 MHz, 298 K): δ 7.79 (br s, 8H, BAr^F$_4$), 7.57 (br s, 4H, BAr^F$_4$), 7.50 (t, $^{3}J_{HH}$ = 7.8 Hz, 4H, Ar), 7.47 (s, 4H, NC*H*=CHN), 7.26 (d, $^{3}J_{HH}$ = 7.9 Hz, 8H, Ar), 2.15 (sept, $^{3}J_{HH}$ = 6.9 Hz, 8H, C*H*Me$_2$), 1.02 (d, $^{3}J_{HH}$ = 7.1 Hz, 24H, CH*Me*$_2$), 1.00 (d, $^{3}J_{HH}$ = 7.3 Hz, 24H, CH*Me*$_2$), -7.30 (br s, 6H, Ir*H*). Selected ^{13}C{selective-^{1}H} (THF-*d*$_8$, 126 MHz, 278 K): δ 156.9 (br s, IrC_{NHC}).

[Ir(IPr)$_2$(CO)$_2$H$_2$][BAr^F$_4$] (12). A J. Young's resealable ampule was charged with a C_6H_5F (0.5 mL) solution of **1** (80 mg, 0.040 mmol), freeze-pump-thaw degassed three times and exposed to 1 atm CO, leading to a near instantaneous color change from orange-red to colorless. After 5 min shaking, the solution was layered with hexane to yield colorless crystals of **12**. Yield: 43 mg (53 %). ^{1}H NMR (C_6D_5F, 400 MHz, 298 K): δ 8.57 (s, 8H,

BAr$^F{}_4$), 7.87 (s, 4H, BAr$^F{}_4$), 7.58 (t, $^3J_{HH}$ = 7.7 Hz, 4H, Ar), 7.33 (d, $^3J_{HH}$ = 7.7 Hz, 8H, Ar), 7.07 (s, 4H, NC*H*=CHN), 2.38 (sept, $^3J_{HH}$ = 6.9 Hz, 8H, C*H*Me$_2$), 1.18 (d, $^3J_{HH}$ = 6.9 Hz, 24H, CH*Me*$_2$), 1.14 (d, $^3J_{HH}$ = 6.9 Hz, 24H, CH*Me*$_2$), -9.88 (s, 2H, Ir*H*). ATR-IR (ν, cm^{-1}): 2026 (s, CO), 2065 (s, CO). ESI-TOF MS: [M+]$^+$ *m*/*z* = 1027.5369 (theoretical *m*/*z* = 1027.5436).

[Ir(IPr)$_2$(CO)$_2$(ZnMe)$_2$][BAr$^F{}_4$] (13). A J. Young's resealable ampule was charged with a C_6H_5F (0.5 mL) solution of **3** (38 mg, 0.019 mmol), subjected to three freeze-pump-thaw cycles of degassing and placed under 1 atm CO. A near instantaneous color change from red-orange to colorless was observed. The solution was layered with hexane to yield colorless crystals of **13**. Yield: 27 mg (69 %). ^{1}H NMR (C_6D_5F, 400 MHz, 298 K): δ 8.57 (s, 8H, BAr$^F{}_4$), 7.86 (s, 4H, BAr$^F{}_4$), 7.66 (t, $^3J_{HH}$ = 7.5 Hz, 2H, Ar), 7.62 (t, $^3J_{HH}$ = 7.5 Hz, 2H, Ar), 7.42 (m, 8H, Ar),* 7.06 (s, 2H, NC*H*=CHN),# 2.87 (sept, $^3J_{HH}$ = 6.7 Hz, 2H, C*H*Me$_2$), 2.71 (sept, $^3J_{HH}$ = 6.7 Hz, 2H, C*H*Me$_2$), 2.39 (sept, $^3J_{HH}$ = 6.7 Hz, 2H, C*H*Me$_2$), 2.37 (sept, $^3J_{HH}$ = 6.7 Hz, 2H, C*H*Me$_2$), 1.49 (d, $^3J_{HH}$ = 6.7 Hz, 6H, CH*Me*$_2$), 1.42 (d, $^3J_{HH}$ = 6.7 Hz, 6H, CH*Me*$_2$), 1.37 (d, $^3J_{HH}$ = 6.7 Hz, 12H, CH*Me*$_2$), 1.20 (d, $^3J_{HH}$ = 6.7 Hz, 6H, CH*Me*$_2$), 1.14 (d, $^3J_{HH}$ = 6.8 Hz, 6H, CH*Me*$_2$), 1.08 (d, $^3J_{HH}$ = 6.9 Hz, 6H, CH*Me*$_2$), 1.05 (d, $^3J_{HH}$ = 6.7 Hz, 6H, CH*Me*$_2$), -0.82 (s, 6H, Zn*Me*) (*obscured by benzene impurity in solvent; #second NCH=C*H*N resonance obscured by C_6H_5F). ATR-IR (ν, cm^{-1}): 2001 (s, CO), 2035 (s, CO). ESI-TOF MS: [M]$^+$ *m*/*z* = 1183.4216 (theoretical *m*/*z* = 1183.4331).

X-ray crystallography. Data for all compounds (Table S1) were collected using an Agilent Supernova diffractometer at 150 K. All structures were solved using SHELXT[7] and refined using SHELXL[8] *via* the Olex2[9] interface. The motif in all structures corresponded to one cation plus one anion and disorder modelling was accompanied with the inclusion of relevant distance and ADP restraints, to assist convergence. Additional, noteworthy points follow.

The cation in (0.5)**2**(0.5)**3** contains a 50:50 mixture of cyclometalated and non-cyclometalated moieties. As such, C51, C52 and C53 were treated for disorder and, once this modelling took place, the two hydrogen atoms pertaining to the activated methyl (C52) were readily located and refined subject to being equidistant from the parent atom. The hydride associated with the cyclometalated species (which would bear half site-occupancy) eluded credible location and, hence, was omitted from the refinement. In the anion, the fluorines attached to C60, C61, C68 and C77 were addressed by taking account of 55:45, 55:45, 50:50 and 60:40, respective, disorder ratios. Additionally, the whole of the CF_3 groups containing C69 and C85, plus phenyl carbons C81, C82 and C83 were modeled to take account of an even split over two locations.

In (0.25)**2**(0.75)**3**, the cation also equates to an average of non-cyclometalated and cyclometalated species, this time in a 75:25 ratio. Thus, C54, C55 and C56 were treated for disorder. The hydrogen atoms associated with the minor disordered portion of C55 in the activated species, each of which would have one-quarter site-occupancy, could not be reliably located and were, therefore, omitted from the refinement. CF_3 disorder in the anion was modelled for the fluorines attached to C63, C68, C71, C76 and C79 in 85:15, 70:30, 50:50, 70:30 and 75:25 ratios (respectively) while the whole of the trifluoromethyl group containing C87 was treated for 60:40 disorder.

All four hydrides in **4** were located based on credible electron density maxima accompanied by sensible U_{iso} values. H1, H2 and H3 were refined without restraints, as was H4 albeit its location at a shorter distance than one might have anticipated [1.37(4) Å] from Ir1. The esd values pertaining to the M-H distances are all typically large. However, the stability of the hydride positions through refinement affords these assignments with ample plausibility despite their locations at a metal core with significant electron density. CF_3 disorder was modelled for the fluorines attached to C63, C68, C71, C76 and C79 in 85:15,

70:30, 50:50, 70:30 and 85:15 ratios (respectably) while the whole of the trifluoromethyl group containing C87 was treated for 60:40 disorder. There are two hydrides in the cation in **5** and, of these, H2, was readily located and refined freely. H1 was somewhat more elusive, but a tentative positional suggestion has been made in the model, based on the associated electron density being similarly distant from Ir1 and Zn1 as H2 is from the iridium center and Zn2, respectively. The U_{iso} for H1 was refined without restraints, thereby offering more than a soupçon of credibility as to its assignment. The question begs as to *why* H1 is less well defined than H2 and the answer may be related to the fact that H1 lies close to the highest residual electron density peak (Q1) in the difference Fourier map. In particular, Q1, is located 0.707 Å from H1 and 2.235 Å from Ir1. As such, this peak may be indicative of some very minor disorder pertaining to Zn1. Alternatively, it may arise from a very minor reaction by-product or (indeed) may just be an artifact in the data. The $[BAr^F{}_4]^-$ anion did not fail to delight with disorder, and this structure was no exception with fluorines in the CF_3 groups based on C60, C63 and C71 being treated for respective disorders of 65:35, 50:50 and 85:15. Additionally, the entire CF_3 moieties which included C68, C76 and C84 were modelled to take account of 70:30, 75:25 and 60:40 disorder ratios.

In the structure of **6**, the asymmetric unit comprizes one cation and one anion. In the former, the methyl groups attached to C40 were treated for 75:25 disorder. Additionally, C12, C13, C25 and C26 were each modeled to take account of a 55:45 split. This renders, overall, a 55:45 ratio of non-metalated to metalated cations in the overall sample. In the anion, the fluorines attached to C63, C76 and C87 were modelled to take account of respective 75:25, 75:25 and 68:32 disorders while the entire CF3 groups containing C71 and C84 were treated for sequential 75:25 and 60:60 splits. The hydrogens attached to C26A were not readily evident in the electron density map and, hence, were included at calculated positions. One cation and one anion constitute the asymmetric unit in the structure of **7**. The hydride in the

cation was located and refined without restraints. In the anion, the fluorines attached to C60, C63, C68 and C79 were modelled to take account of respective 60:40, 50:50, 55:45 and 55:55 disorders while the entire CF_3 moiety based on C87 was treated for a 75:25 split. The hydrogens attached to C34 were not readily evident in the electron density map and, hence, were included at calculated positions.

One cation and one anion constitute the asymmetric unit in the structure of **8**. The hydrides in the cation were located and were refined without distance restraints. CF_3 disorder was modelled for the fluorines attached to C63, C76 and C87 in 85:15, 55:45, 50:50 and 60:40 ratios (respectively) while the whole of the trifluoromethyl group containing C84 was treated for 55:45 disorder.

Table S1. Crystal data and structural refinement details.

Identification code	**(0.5)2(0.5)3**	**(0.25)2(0.75)3**	**4**	**5**	**6**
Empirical formula	$C_{88}H_{90}BF_{24}IrN_4Zn_2$	$C_{88}H_{90}BF_{24}IrN_4Zn_2$	$C_{88}H_{94}BF_{24}IrN_4Zn_2$	$C_{88}H_{92}BF_{24}IrN_4Zn_2$	$C_{88}H_{89.55}BCd_2F_{24}IrN_4$
Formula weight	1993.38	1993.38	1997.42	1995.40	2086.99
Crystal system	monoclinic	monoclinic	monoclinic	monoclinic	monoclinic
Space group	$P2_1/c$	$P2_1/n$	$P2_1/n$	$P2_1/n$	$P2_1/n$
a/ Å	20.1389(5)	13.3423(3)	13.38005(16)	13.3304(2)	13.4901(2)
b/ Å	14.9841(4)	16.5165(4)	16.5854(2)	16.4852(2)	16.6109(2)
c/ Å	28.4595(8)	40.3445(12)	40.3637(5)	40.3109(6)	40.0168(5)
α/ °	90	90	90	90	90
β/ °	92.778(2)	98.329(3)	98.2853(12)	98.1711(15)	98.994(1)
γ/ °	90	90	90	90	90
U/ Å^3	8578.0(4)	8796.9(4)	8863.78(19)	8768.5(2)	8856.8(2)
Z	4	4	4	4	4
ρ_{calc}/ g cm^{-3}	1.544	1.505	1.497	1.512	1.565
μ/ mm^{-1}	2.201	4.349	4.316	2.154	7.500
F(000)	4016.0	4016.0	4032.0	4024.0	4158.0
Crystal size/ mm^3	0.185 × 0.08 × 0.062	0.075 × 0.065 × 0.047	0.082 × 0.07 × 0.054	0.133 × 0.117 × 0.039	0.131 × 0.108 × 0.073
Radiation	Mo Kα (λ = 0.71073)	Cu Kα (λ = 1.54184)	Cu Kα (λ = 1.54184)	Mo Kα (λ = 0.71073)	Cu Kα (λ = 1.54184)
2θ range for data collection/°	6.576 to 54.97	7.352 to 140.126	6.928 to 140.148	6.596 to 55.028	6.952 to 146.902
Index ranges	$-26 \le h \le 26$, $-19 \le k \le 17$, $-36 \le l \le 31$	$-15 \le h \le 16$, $-20 \le k \le 17$, $-49 \le l \le 45$	$-14 \le h \le 16$, $-18 \le k \le 20$, $-49 \le l \le 37$	$-17 \le h \le 17$, $-21 \le k \le 21$, $-51 \le l \le 52$	$-16 \le h \le 16$, $-20 \le k \le 19$, $-45 \le l \le 49$
Reflections collected	78331	44735	38966	75598	118164
Independent reflections, R_{int}	19639, 0.0693	16640, 0.0595	16762, 0.0318	20117, 0.0463	17719, 0.0537
Data/restraints/parameters	19639/613/1312	16640/446/1292	16762/361/1280	20117/384/1284	17719/489/1298
Goodness–of–fit on F^2	1.171	1.138	1.053	1.039	1.067
Final R1, wR2 [I>=2σ(I)]	0.0546, 0.0927	0.0749, 0.1787	0.0363, 0.0885	0.0404, 0.0794	0.0509, 0.1244
Final R1, wR2 [all data]	0.0841, 0.1006	0.0911, 0.1877	0.0454, 0.0931	0.0587, 0.0861	0.0568, 0.1283
Largest diff. peak/hole/ e Å^{-3}	1.56/−0.80	1.84/−1.34	0.84/−0.61	0.77/−0.73	1.49/−1.73

Identification code	**7**	**8**
Empirical formula	$C_{88}H_{90}BCd_2F_{24}IrN_4$	$C_{88}H_{94}BCd_2F_{24}IrN_4$
Formula weight	2087.44	2091.48
Crystal system	monoclinic	monoclinic
Space group	$P2_1/c$	$P2_1/n$
a/ Å	20.1631(2)	13.5206(1)
b/ Å	15.0085(10)	16.5650(1)
c/ Å	28.6737(3)	40.2731(3)
α/ °	90	90
β/ °	92.5050(1)	98.636(1)
γ/ °	90	90
U/ Å^3	8668.88(14)	8917.65(11)
Z	4	4
ρ_{calc}/ g cm^{-3}	1.599	1.558
μ/ mm^{-1}	7.662	7.449
F(000)	4160.0	4176.0
Crystal size/ mm^3	0.145 × 0.133 × 0.114	0.173 × 0.066 × 0.057
Radiation	Cu Kα (λ = 1.54184)	Cu Kα (λ = 1.54184)
2θ range for data collection/°	6.648 to 146.768	6.652 to 145.998
Index ranges	−25 ≤ h ≤ 24,<br>−17 ≤ k ≤ 18,<br>−35 ≤ l ≤ 35	−16 ≤ h ≤ 16,<br>−19 ≤ k ≤ 20,<br>−49 ≤ l ≤ 49
Reflections collected	121785	93338
Independent reflections, R_{int}	17345, 0.0578	17760, 0.0390
Data/restraints/parameters	17345/535/1246	17760/361/1231
Goodness–of–fit on F^2	1.080	1.032
Final R1, wR2 [I>=2σ(I)]	0.0344, 0.0821	0.0320, 0.0790
Final R1, wR2 [all data]	0.0396, 0.0851	0.0350, 0.0810
Largest diff. peak/hole/ e Å^{-3}	1.55/−0.65	1.54/−1.38

S1.2 NMR and IR Spectra

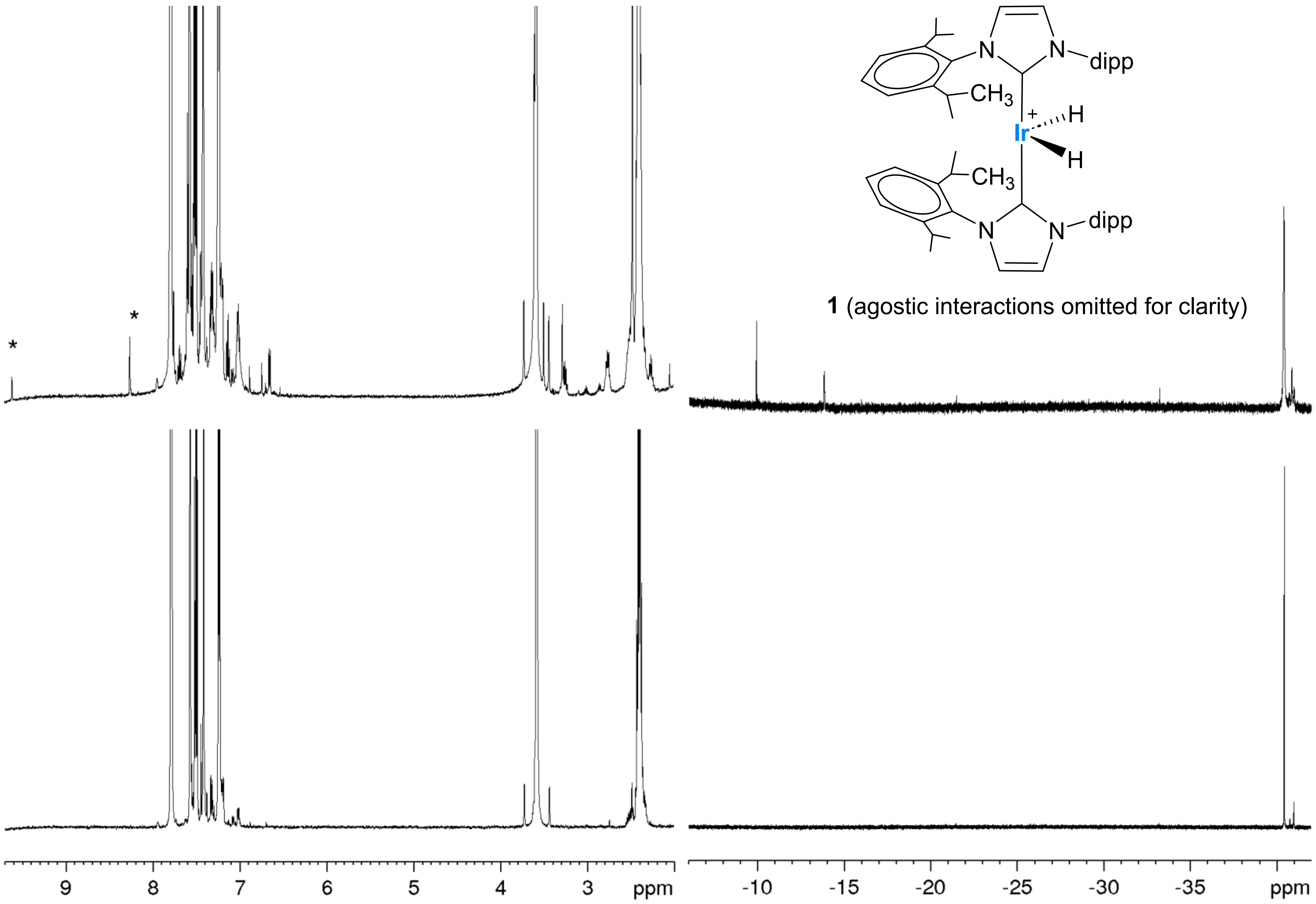

Figure S1. Room temperature ^{1}H NMR spectrum (THF-d_8, 500 MHz, 298 K) of (bottom) redissolved crystalline $[Ir(IPr)_2H_2][BAr^F{}_4]$ (**1**) then (top) after standing for 1 week in solution (* = $[IPrH][BAr^F{}_4]$).

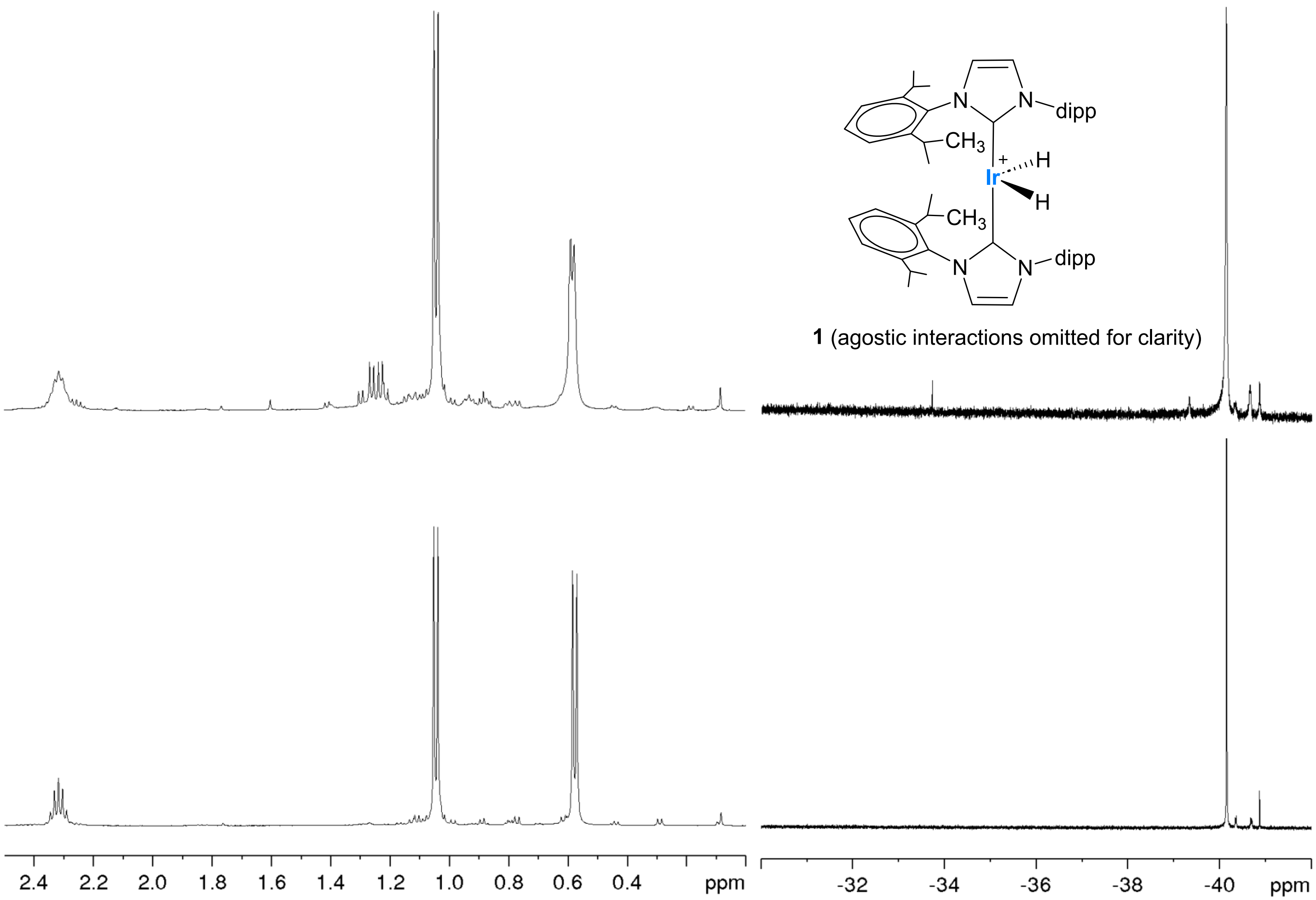

Figure S2. Room temperature ^{1}H NMR spectrum (CD_2Cl_2, 500 MHz, 298 K) of (bottom) redissolved crystalline $[Ir(IPr)_2H_2][BAr^F_4]$ (**1**) then (top) after standing overnight in solution.

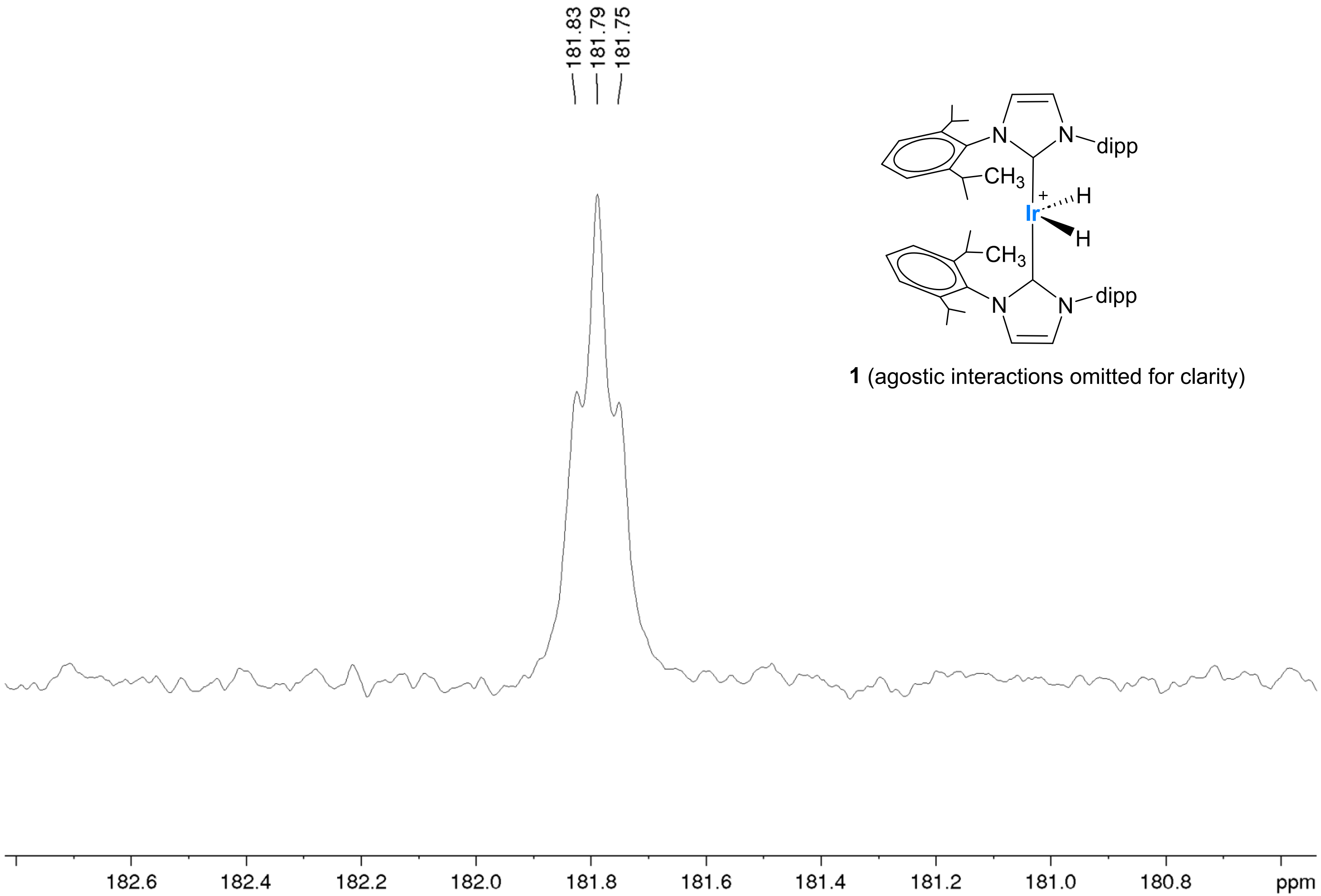

1 (agostic interactions omitted for clarity)

Figure S3. Triplet hydride splitting of the IrC_{IPr} resonance in the ^{13}C{selective ^{1}H}* NMR spectrum (THF-d_8, 126 MHz, 278 K) of [Ir(IPr)$_2$H$_2$][BAr$^F_4$] (**1**) (* ^{1}H decoupler set to decouple aromatic protons only (δ 6-9)).

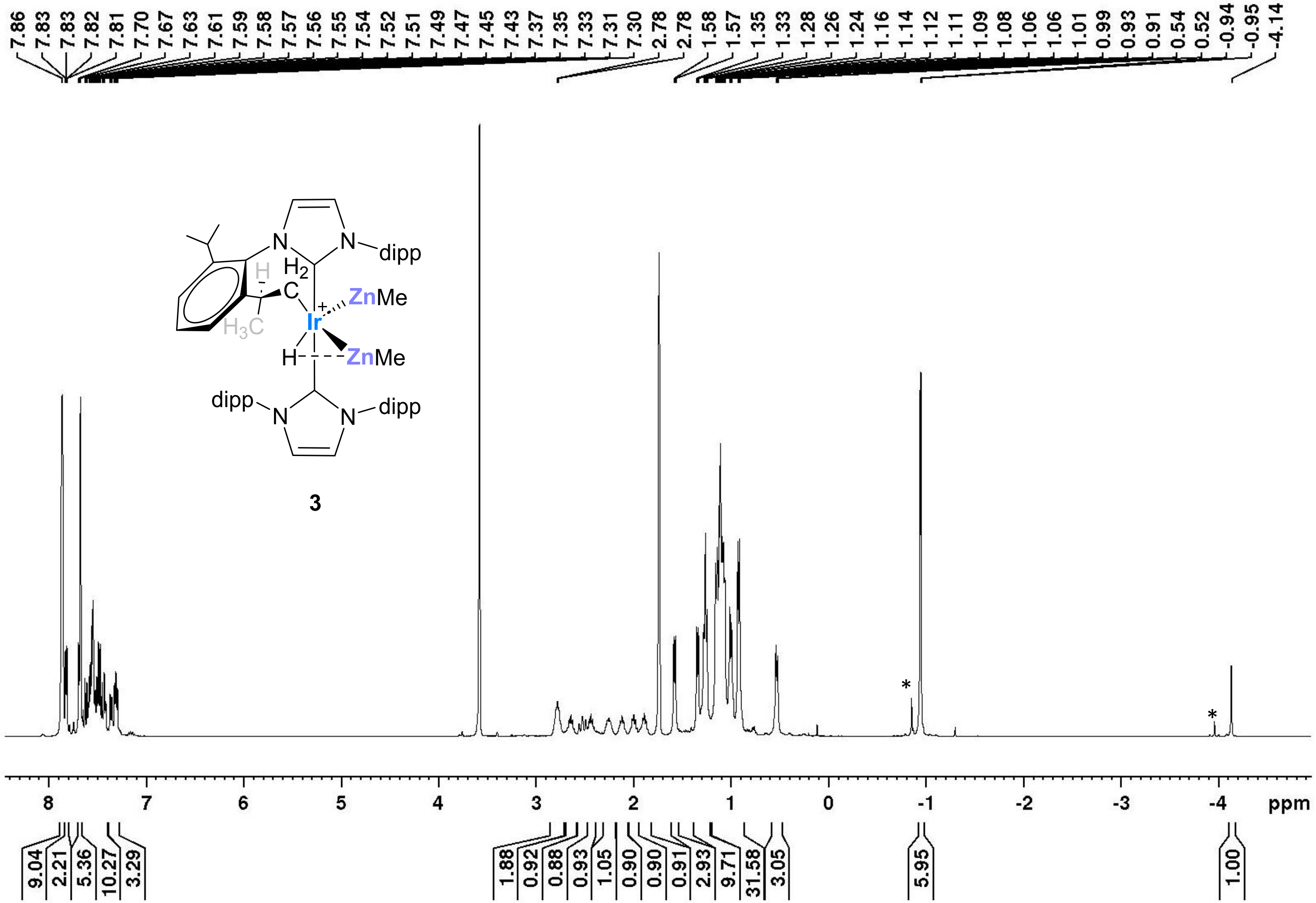

Figure S4. ^{1}H NMR spectrum (THF-d_8, 400 MHz, 219 K) of [Ir(IPr)(IPr')$(ZnMe)_2H$][BAr$^F_4$] (**3**) (* = [Ir$(IPr)_2(ZnMe)_2H_2$][BAr$^F_4$] (**5**)).

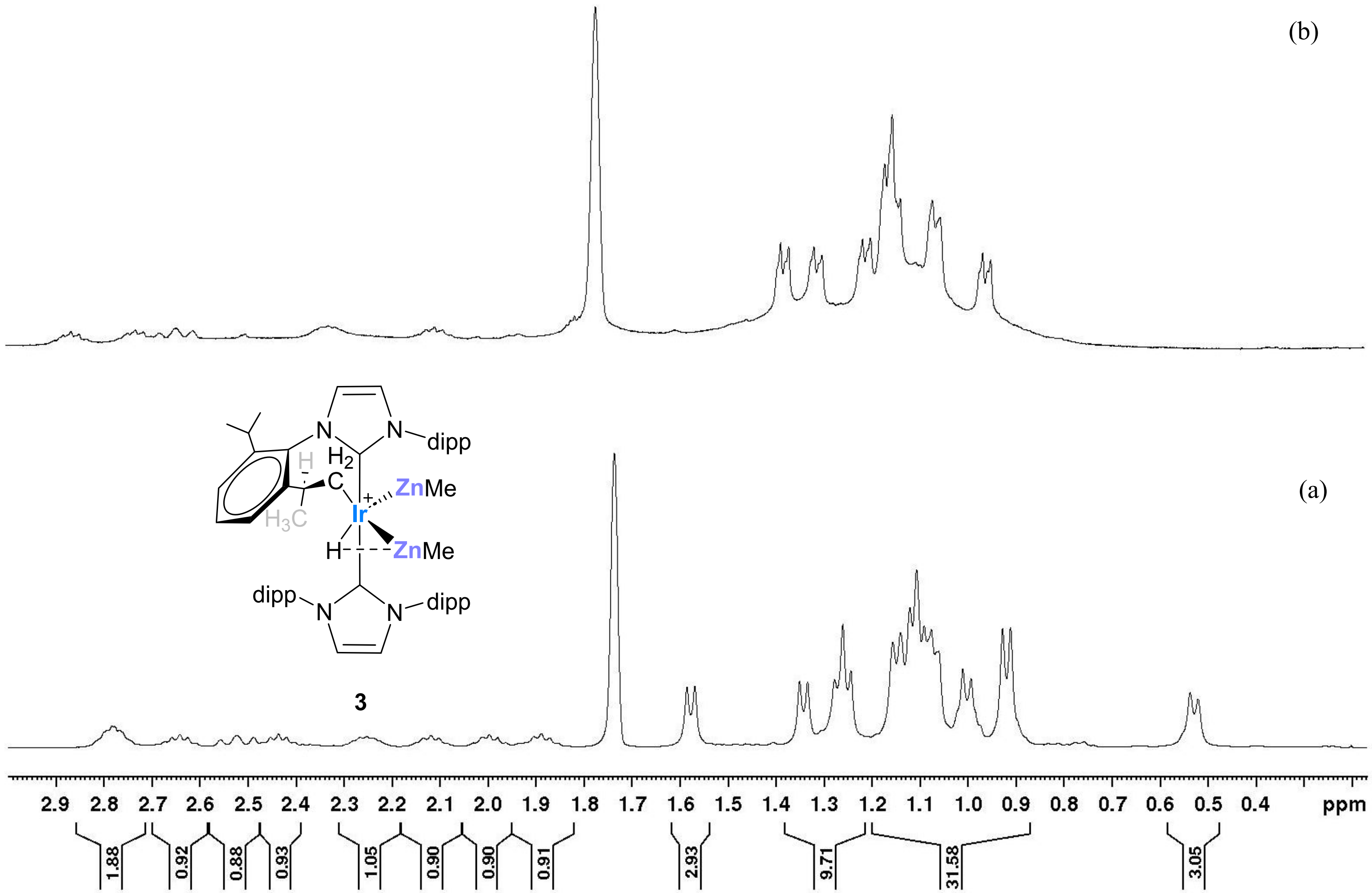

Figure S5. (a) 219 K and (b) 298 K ^{1}H NMR spectra (THF-d_8, 400 MHz) of [Ir(IPr)(IPr')(ZnMe)$_2$H][BAr$^F{}_4$] (**3**) showing the sharpening of the alkyl region at low temperature.

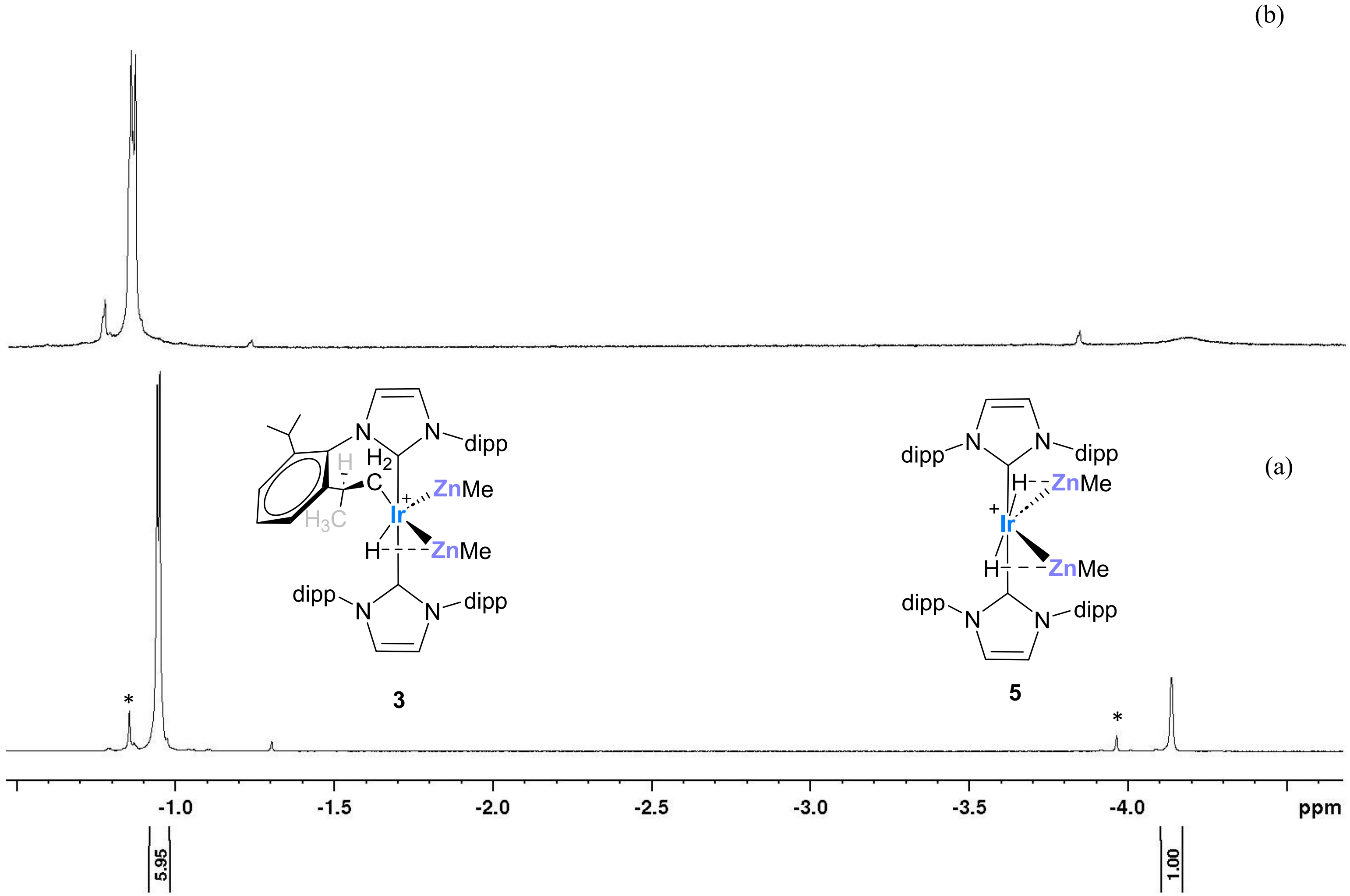

Figure S6. Zn*Me* and Ir*H* resonances of [Ir(IPr)(IPr')$(ZnMe)_2$H][BAr$^F{}_4$] (**3**) in the (a) 219 K and (b) 298 K ^{1}H NMR spectra (THF-d_8, 400 MHz) (* = [Ir$(IPr)_2(ZnMe)_2H_2$][BAr$^F{}_4$] **5**).

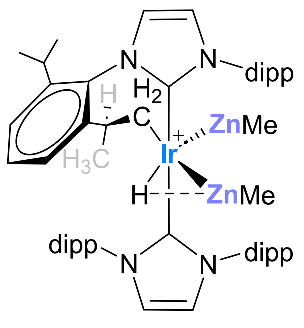

3

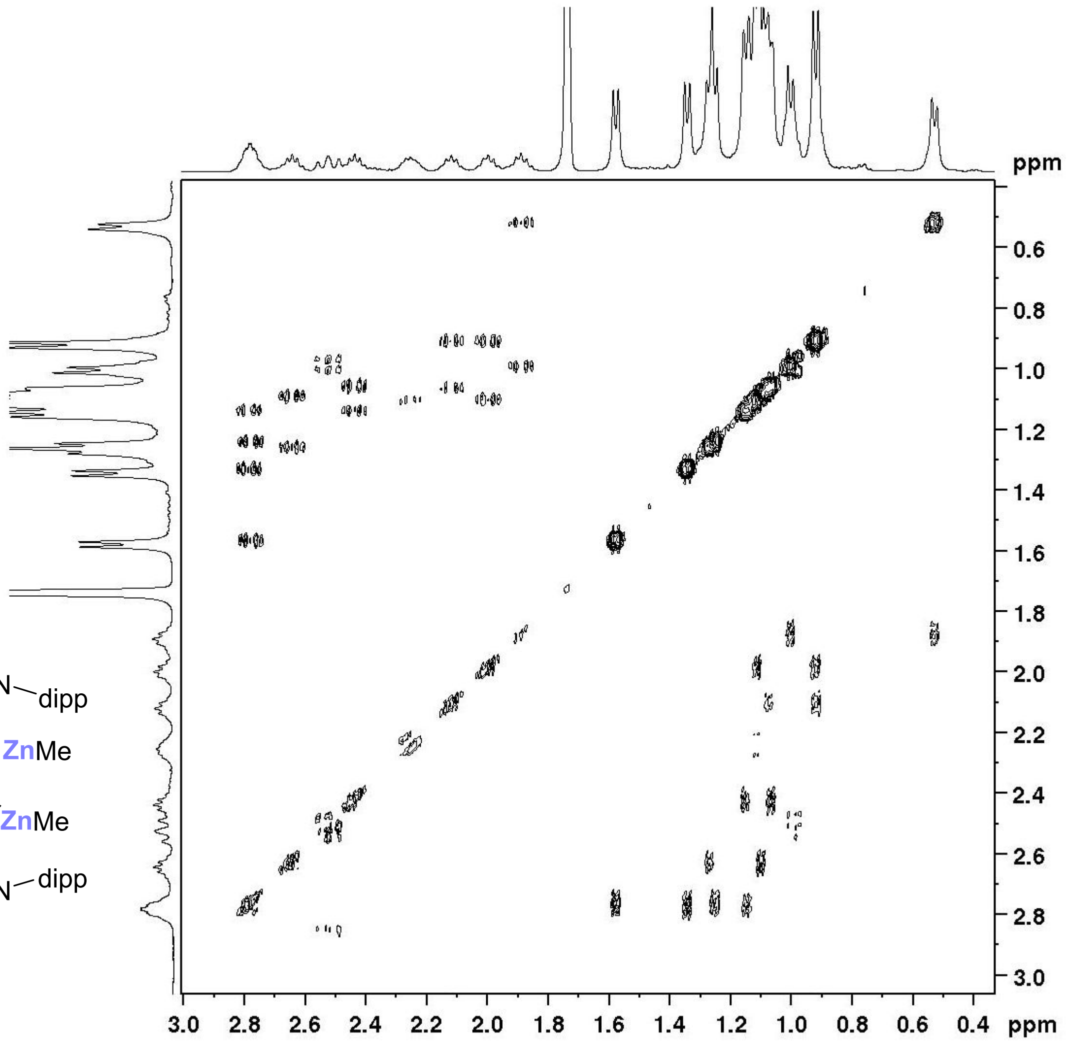

Figure S7. Low temperature (219 K) ^{1}H COSY spectrum (THF-d_8, 400 MHz) of [Ir(IPr)(IPr')(ZnMe)$_2$H][BAr$^F{}_4$] (**3**).

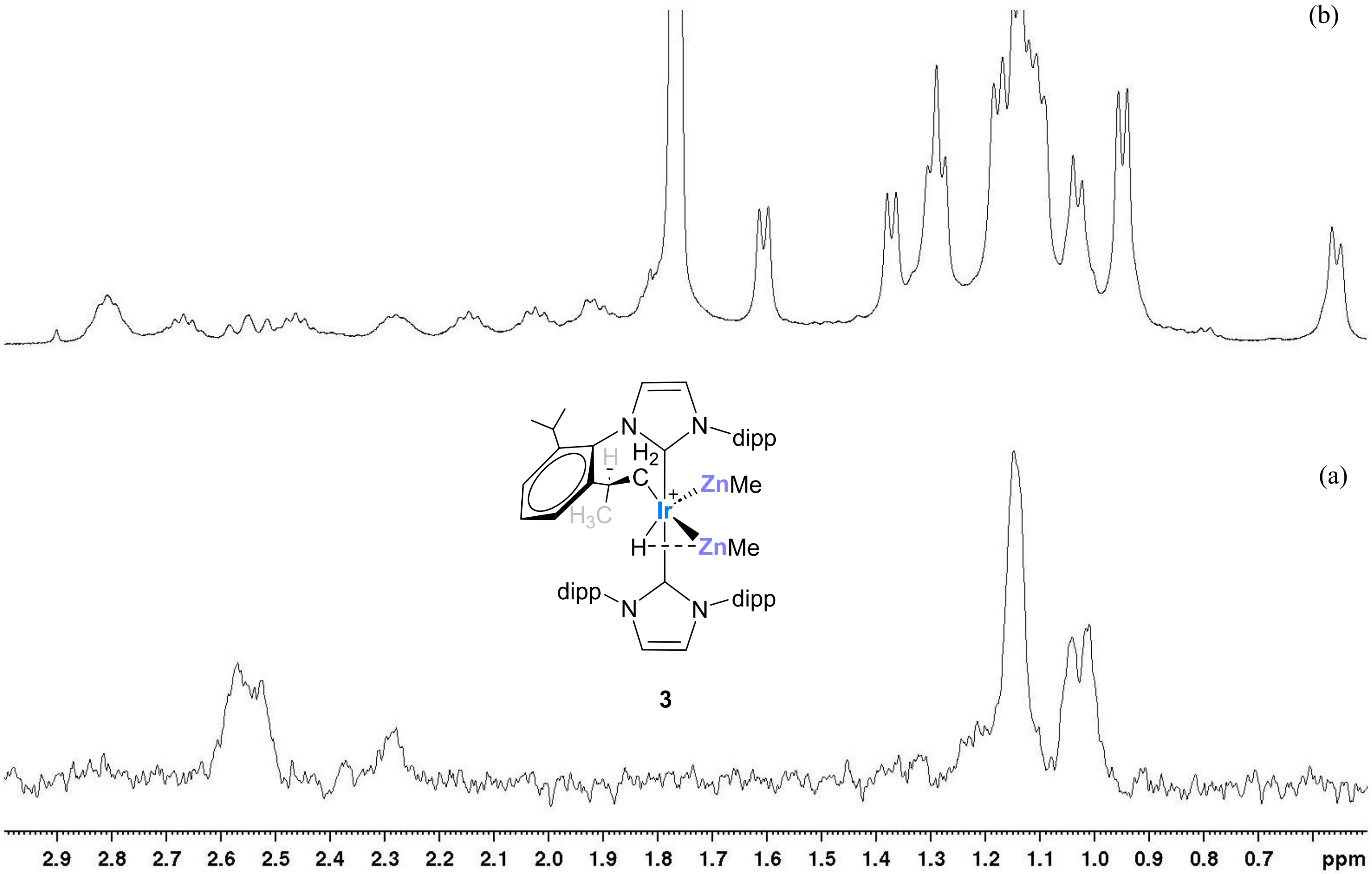

Figure S8. Alkyl region of the low temperature (a) ^{1}H selective TOCSY and (b) standard ^{1}H NMR spectra (THF-d_8, 400 MHz, 219 K) of $[Ir(IPr)(IPr')(ZnMe)_2H][BAr^F{}_4]$ (**3**). The former connects the triplet CH(Me)C*H*HIr resonance (δ 2.55) to the C*H*(Me)CH_2Ir resonance (δ 2.28), the CH(*Me*)CH_2Ir resonance (δ 1.15) and the diastereotopic CH(Me)CH*H*Ir resonance at δ 1.02.

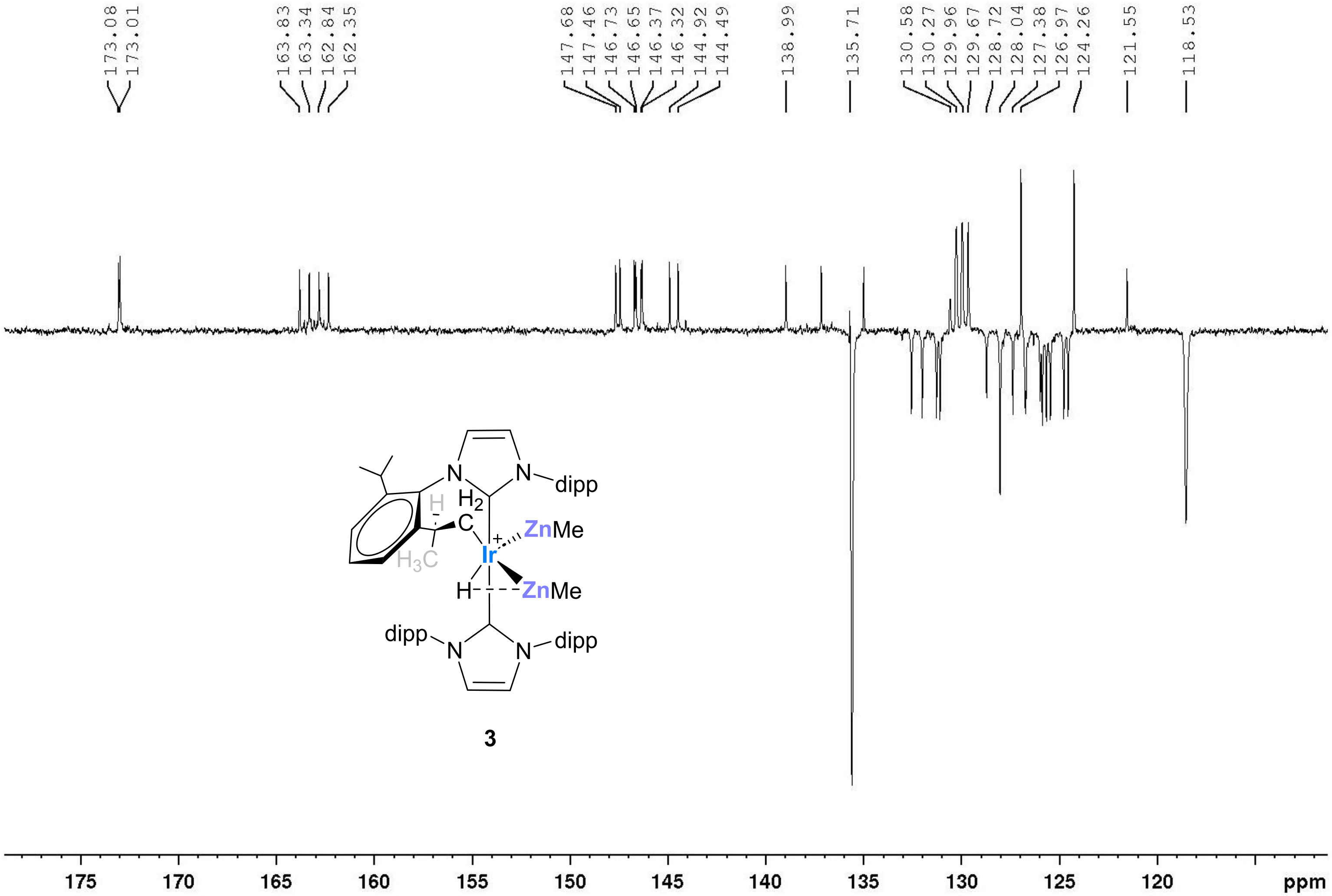

Figure S9. High frequency region of the low temperature (219 K) $^{13}C\{^1H\}$ PENDANT NMR spectrum (THF-d_8, 126 MHz) of $[Ir(IPr)(IPr')(ZnMe)_2H][BAr^F{}_4]$ (**3**).

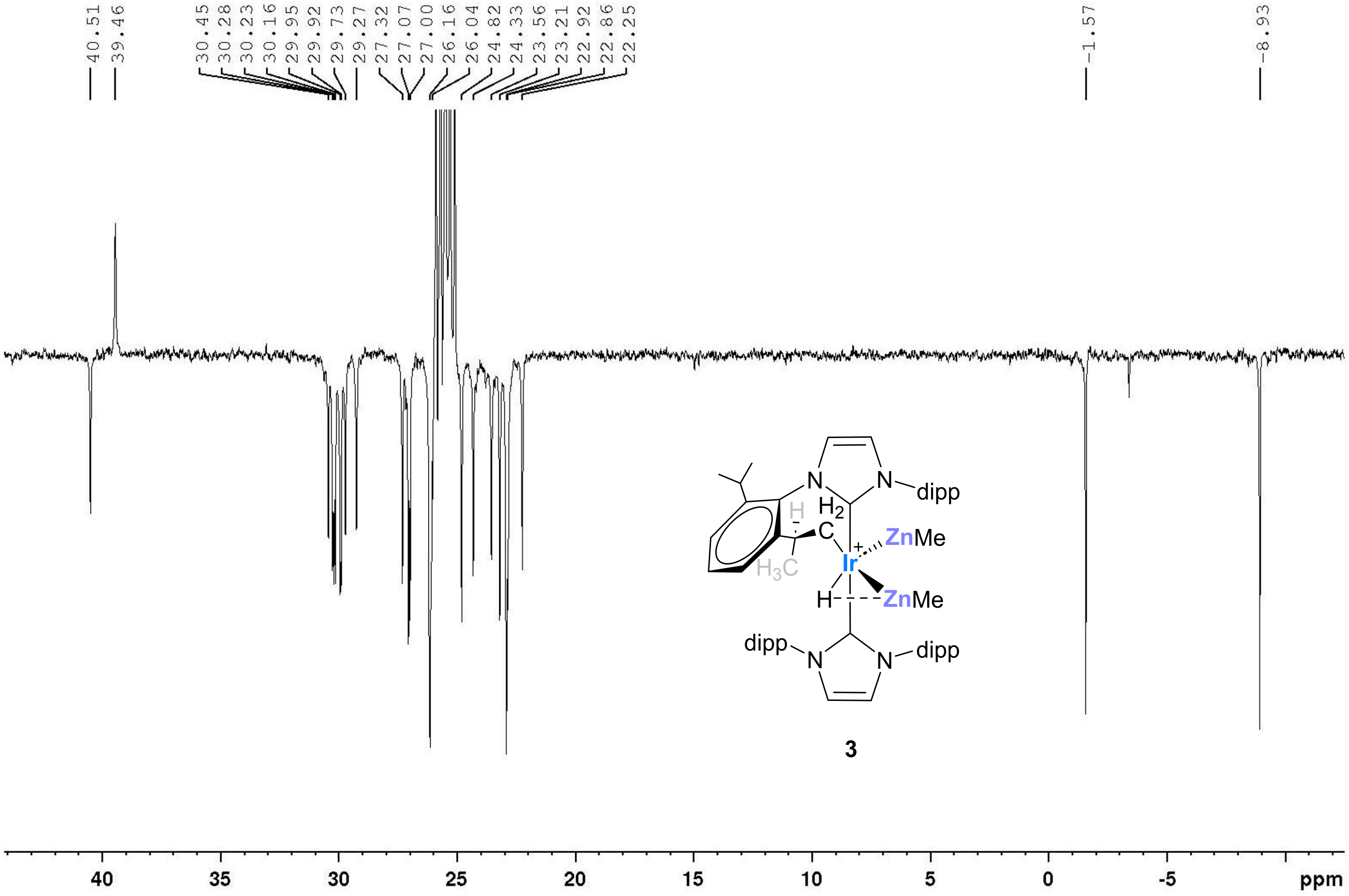

Figure S10. Alkyl region of low temperature (219 K) $^{13}C\{^{1}H\}$ PENDANT NMR spectrum (THF-d_8, 126 MHz) of [Ir(IPr)(IPr')(ZnMe)$_2$H][BAr$^F{}_4$] (**3**).

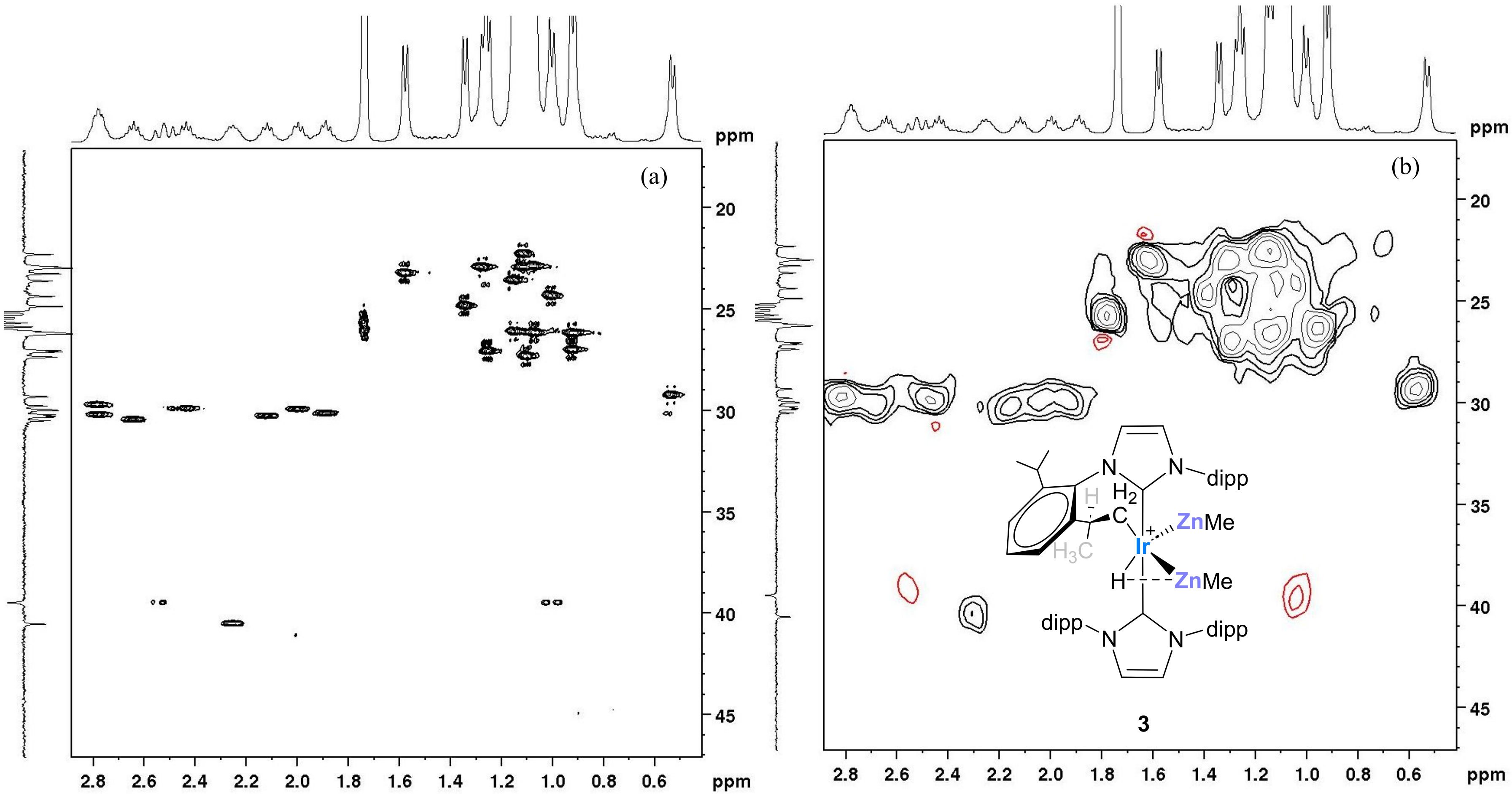

Figure S11. Low temperature (a) ^{1}H-^{13}C HSQC spectrum (THF-d_8, 219 K) of [Ir(IPr)(IPr')(ZnMe)$_2$H][BAr$^{F}{}_4$] (**3**). (b) DEPT-Edited HSQC measurement used to establish CH_2 resonances (shown as red cross peaks).

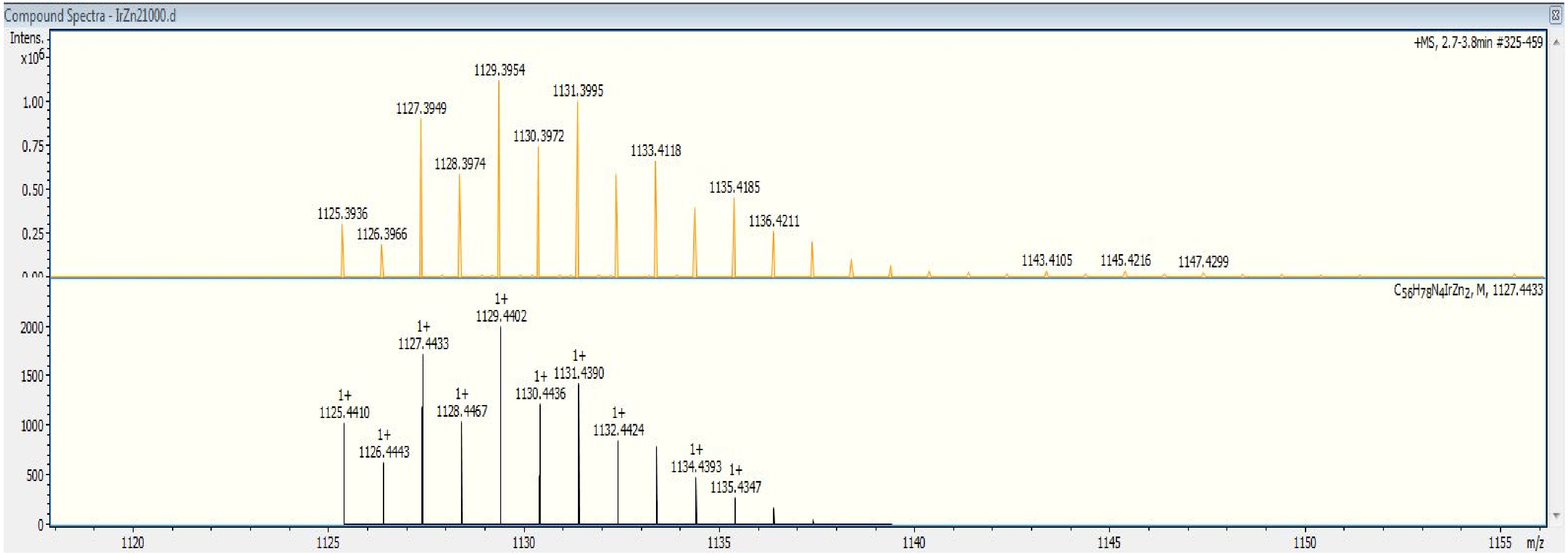

Figure S12. ESI mass spectrum of [Ir(IPr)(IPr')(ZnMe)$_2$H]$^+$ (**3**$^+$).

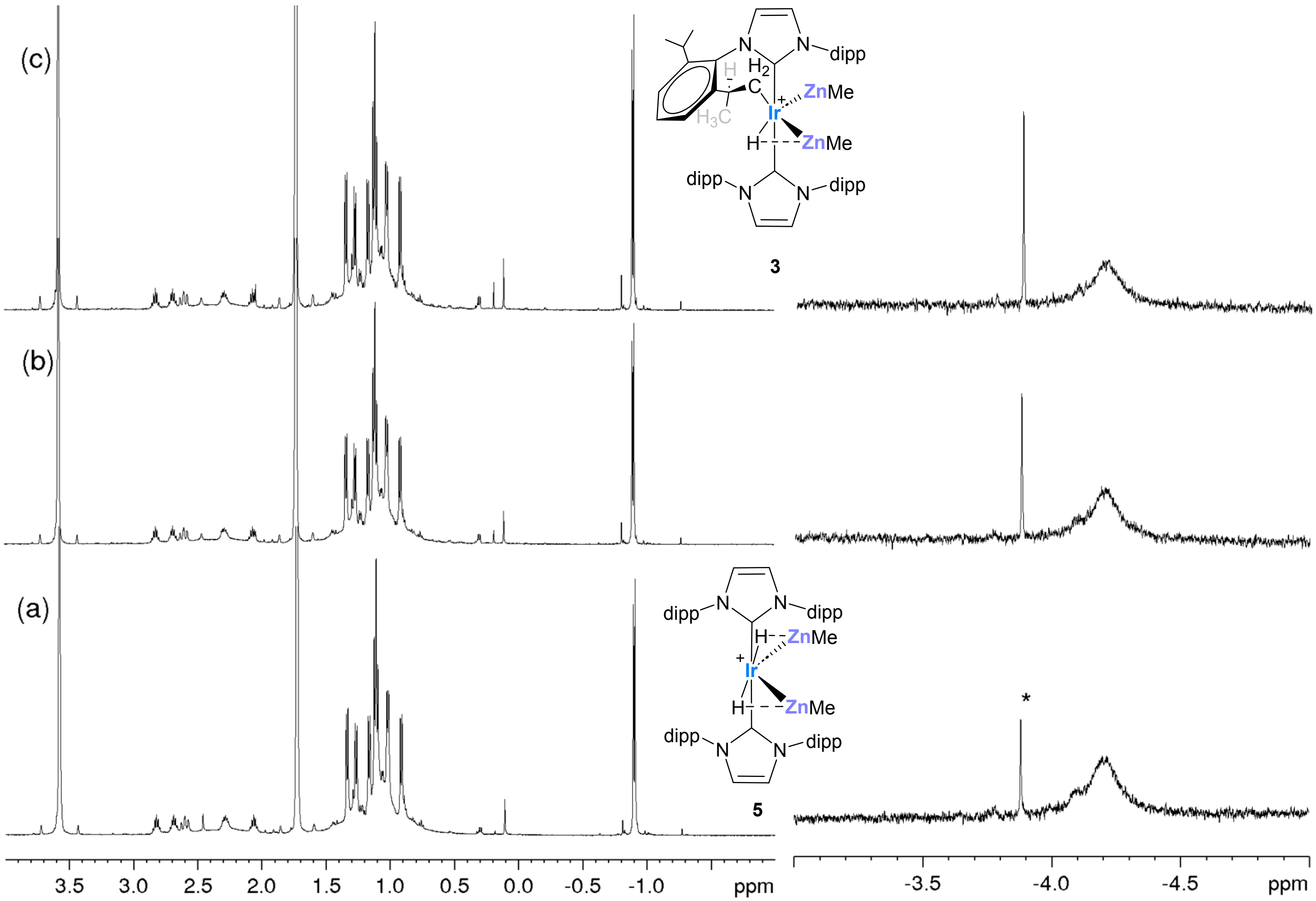

Figure S13. (Left) Alkyl and (right) Ir*H* regions of the ^{1}H NMR spectrum (400 MHz, 298 K) of crystalline [Ir(IPr)(IPr')$(ZnMe)_2$H][BAr^F_4] (**3**) (a) immediately after redissolution in THF-d_8 and then after (b) 5 days and (b) 10 days in solution (* = [Ir$(IPr)_2(ZnMe)_2H_2$][BAr^F_4] **5**).

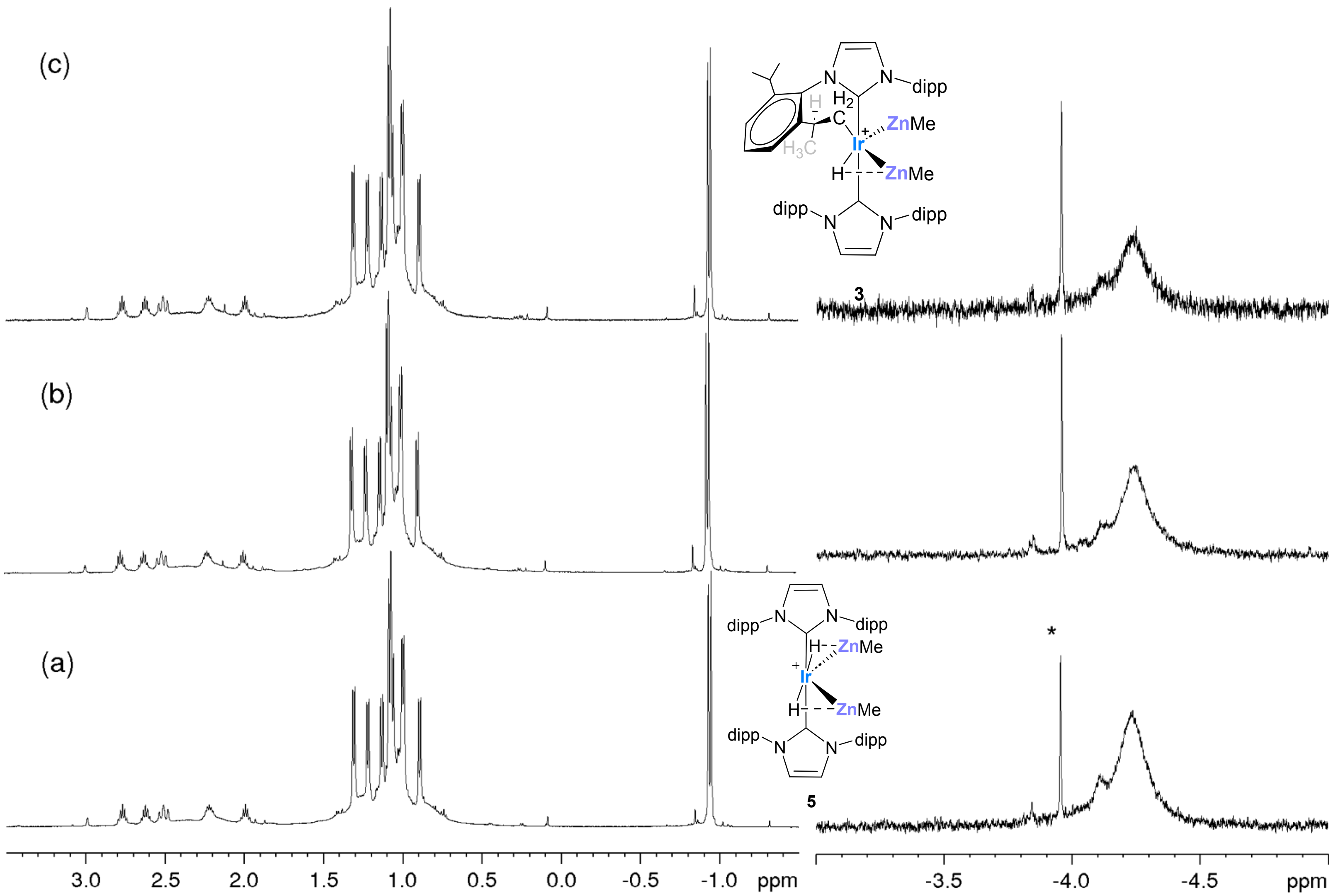

Figure S14. (Left) Alkyl and (right) Ir*H* regions of the ^{1}H NMR spectrum (400 MHz, 298 K) of crystalline [Ir(IPr)(IPr')$(ZnMe)_2$H][BAr^F_4] (**3**) (a) immediately after redissolution in CD_2Cl_2 and then after (b) 5 days and (b) 10 days in solution (* = [Ir$(IPr)_2$$(ZnMe)_2$$H_2$][$BAr^F_4$] **5**).

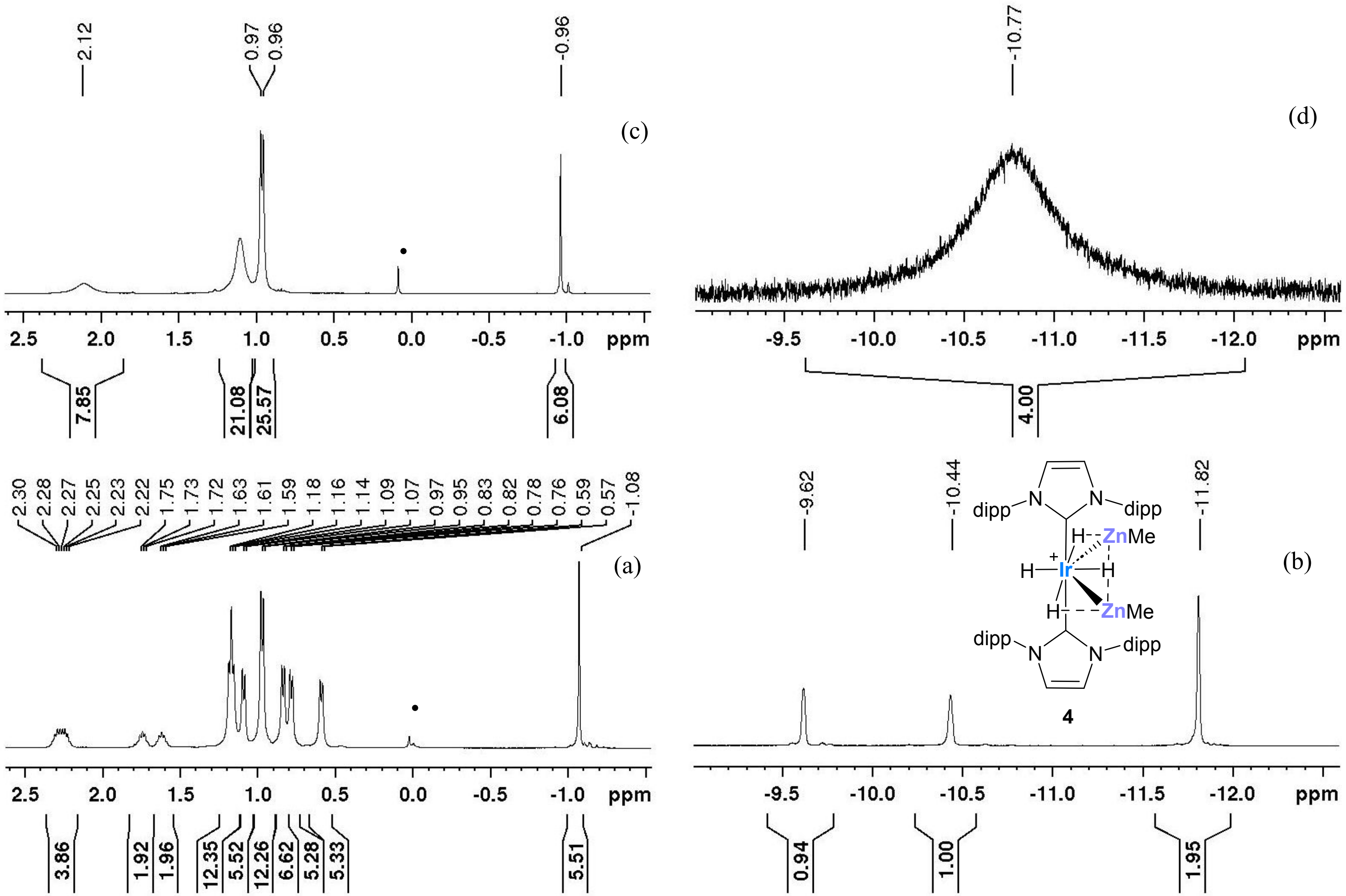

Figure S15. (a) Alkyl and (b) hydride regions of the low temperature (201 K) ^{1}H NMR spectrum (CD_2Cl_2, 400 MHz) of $[Ir(IPr)_2(ZnMe)_2H_4][BAr^F{}_4]$ (**4**). The spectra in (c) and (d) show these same regions in the 298 K spectrum (• = silicone grease)

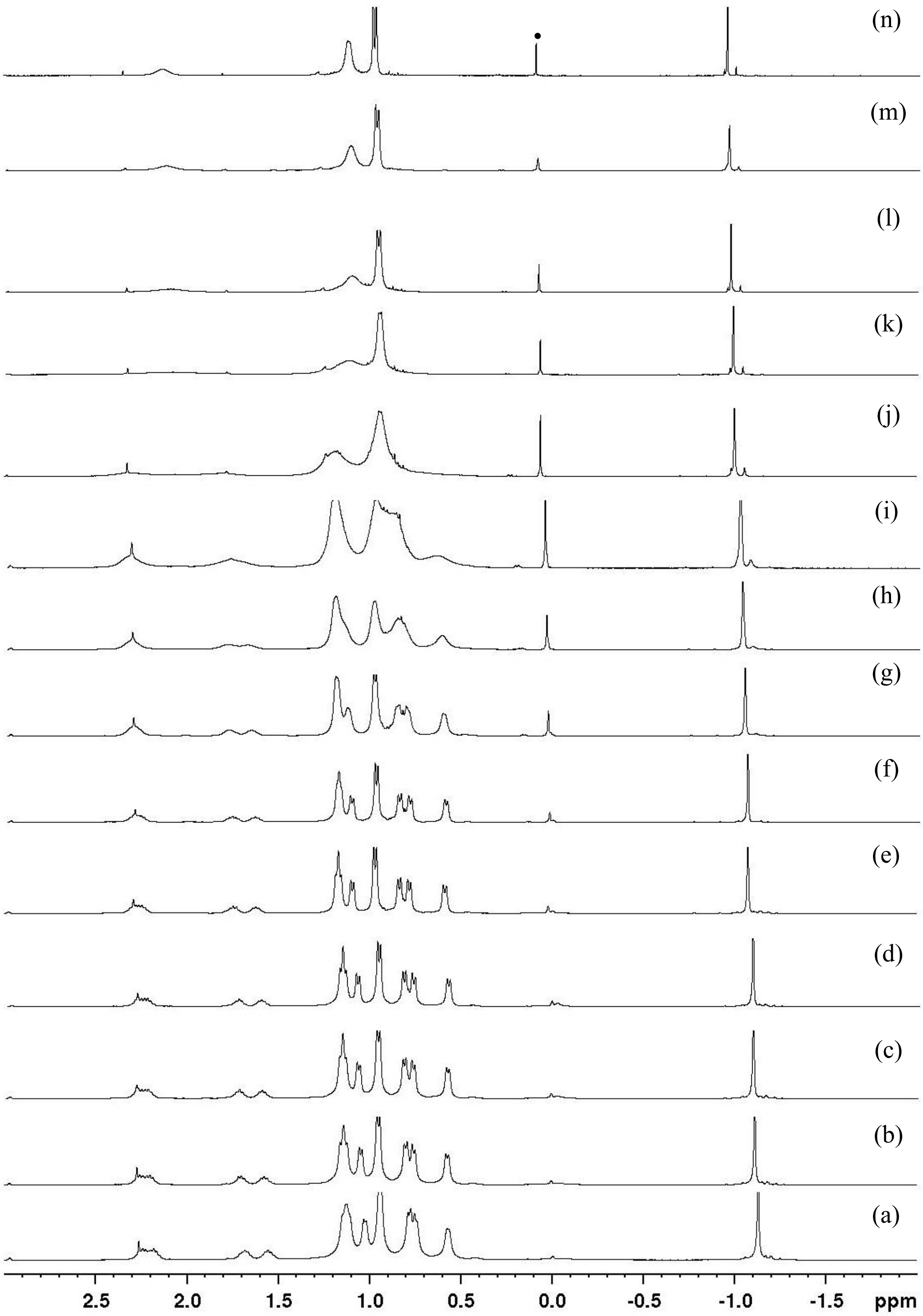

Figure S16. Alkyl region of the VT ^{1}H NMR spectrum (CD_2Cl_2, 400 MHz) of $[Ir(IPr)_2(ZnMe)_2H_4][BAr^F_4]$ (**4**); (a) T = 168, (b) 179, (c) 190, (d) 201, (e) 212, (f) 222, (g) 234, (h) 245, (i) 256, (j) 267, (k) 278, (l) 289, (m) 300 and (n) 310 K (• = silicone grease).

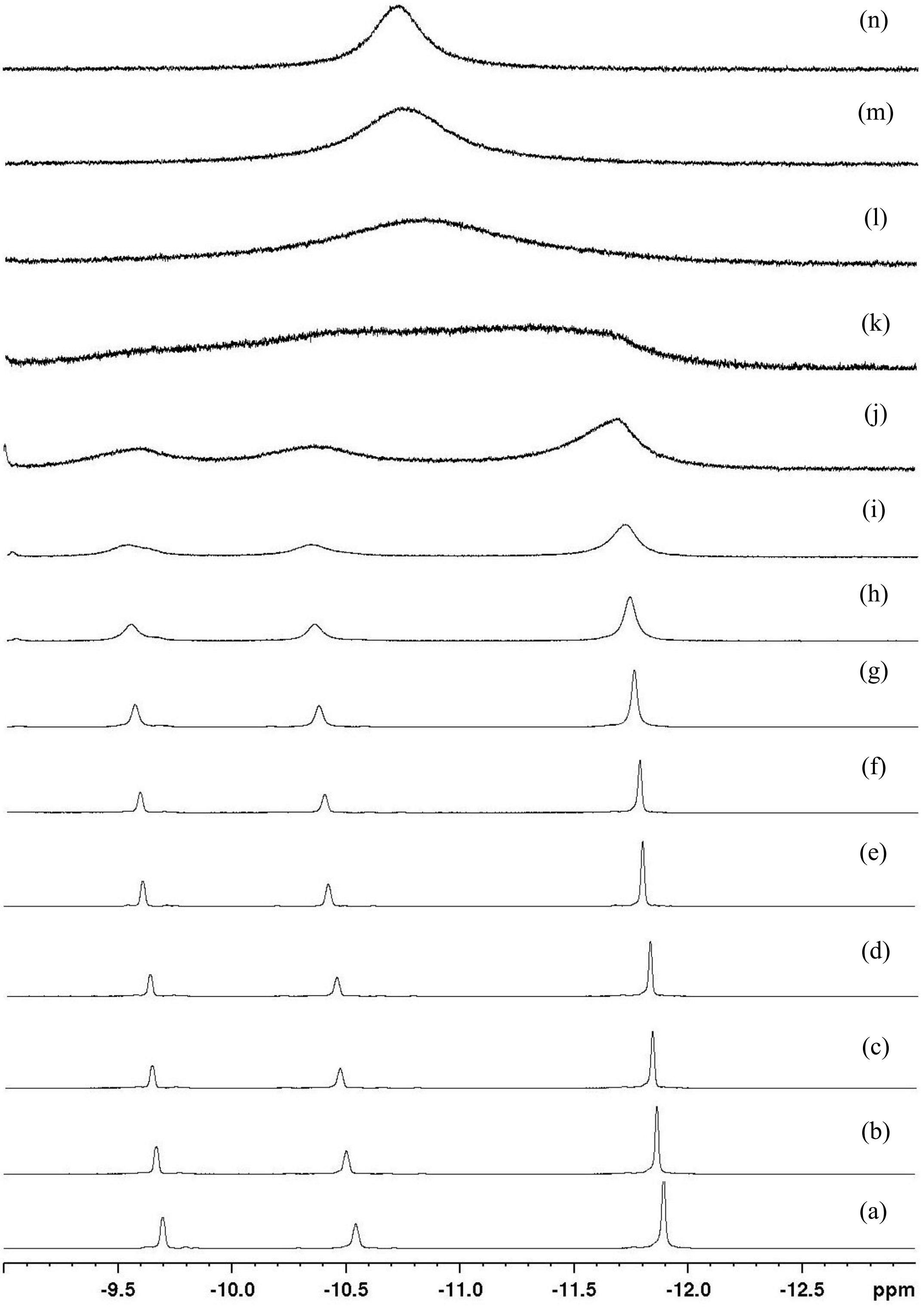

Figure S17. Hydride region of the VT ^{1}H NMR spectrum (CD_2Cl_2, 400 MHz) of $[Ir(IPr)_2(ZnMe)_2H_4][BAr^F{}_4]$ (**4**); (a) T = 168, (b) 179, (c) 190, (d) 201, (e) 212, (f) 222, (g) 234, (h) 245, (i) 256, (j) 267, (k) 278, (l) 289, (m) 300 and (n) 310 K.

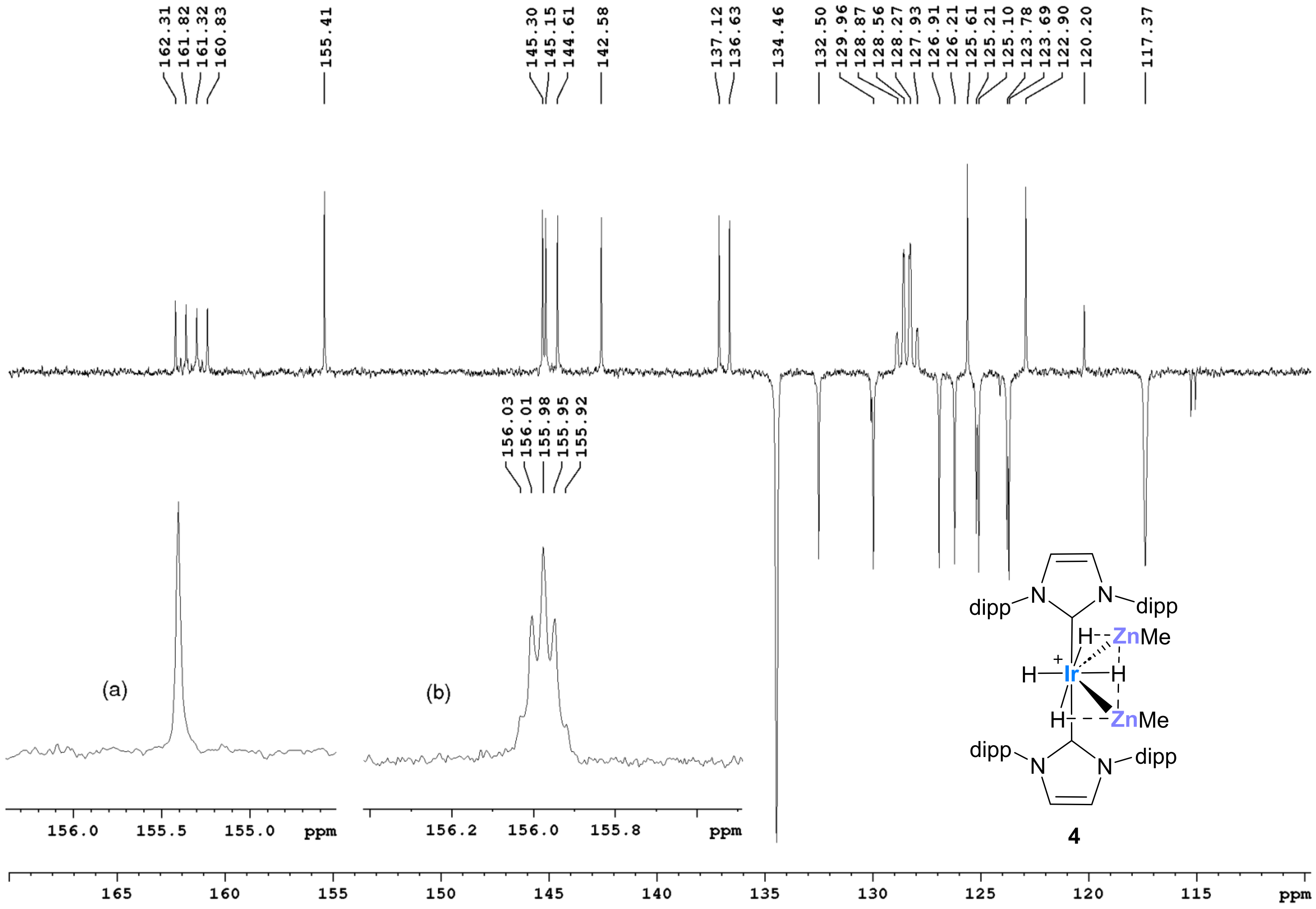

Figure S18. Partial $^{13}C\{^{1}H\}$ DEPTQ NMR spectrum (CD_2Cl_2, 101 MHz, 201 K) of $[Ir(IPr)_2(ZnMe)_2H_4][BAr^F{}_4]$ (**4**). Inset (a) shows expansion of the IrC_{NHC} resonance and (b) the same signal in the $^{13}C\{$selective-$^{1}H\}$ spectrum (THF-d_8, 126 MHz, 278 K).

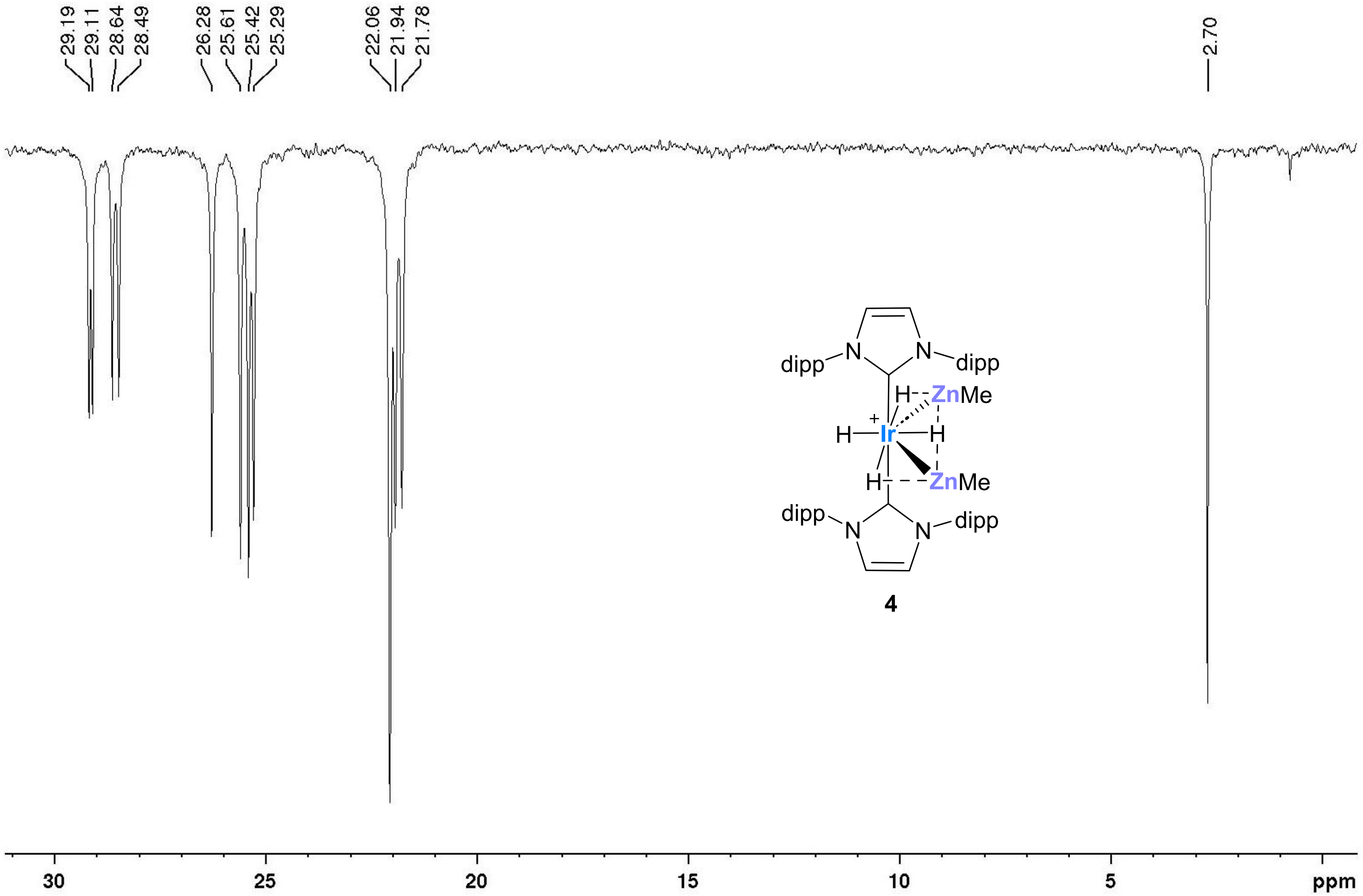

Figure S19. Alkyl region of the low temperature $^{13}C\{^1H\}$ DEPTQ NMR spectrum (CD_2Cl_2, 126 MHz, 201 K) of $[Ir(IPr)_2(ZnMe)_2H_4][BAr^F{}_4]$ (**4**).

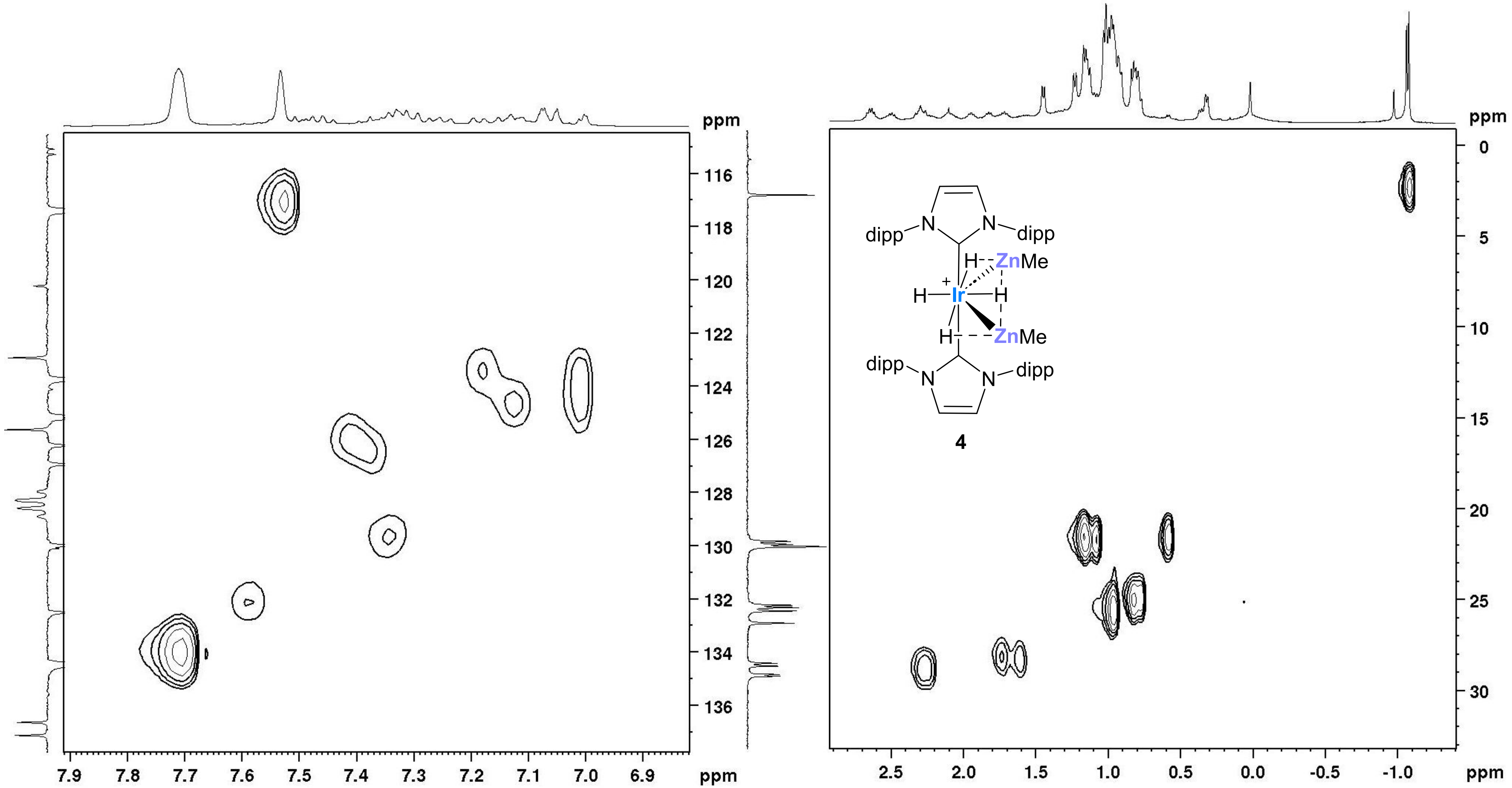

Figure S20. (Left) Aromatic and (right) alkyl regions of the low temperature (201 K) ^{1}H-^{13}C HSQC spectrum (CD_2Cl_2) of $[Ir(IPr)_2(ZnMe)_2H_4][BAr^F{}_4]$ (**4**).

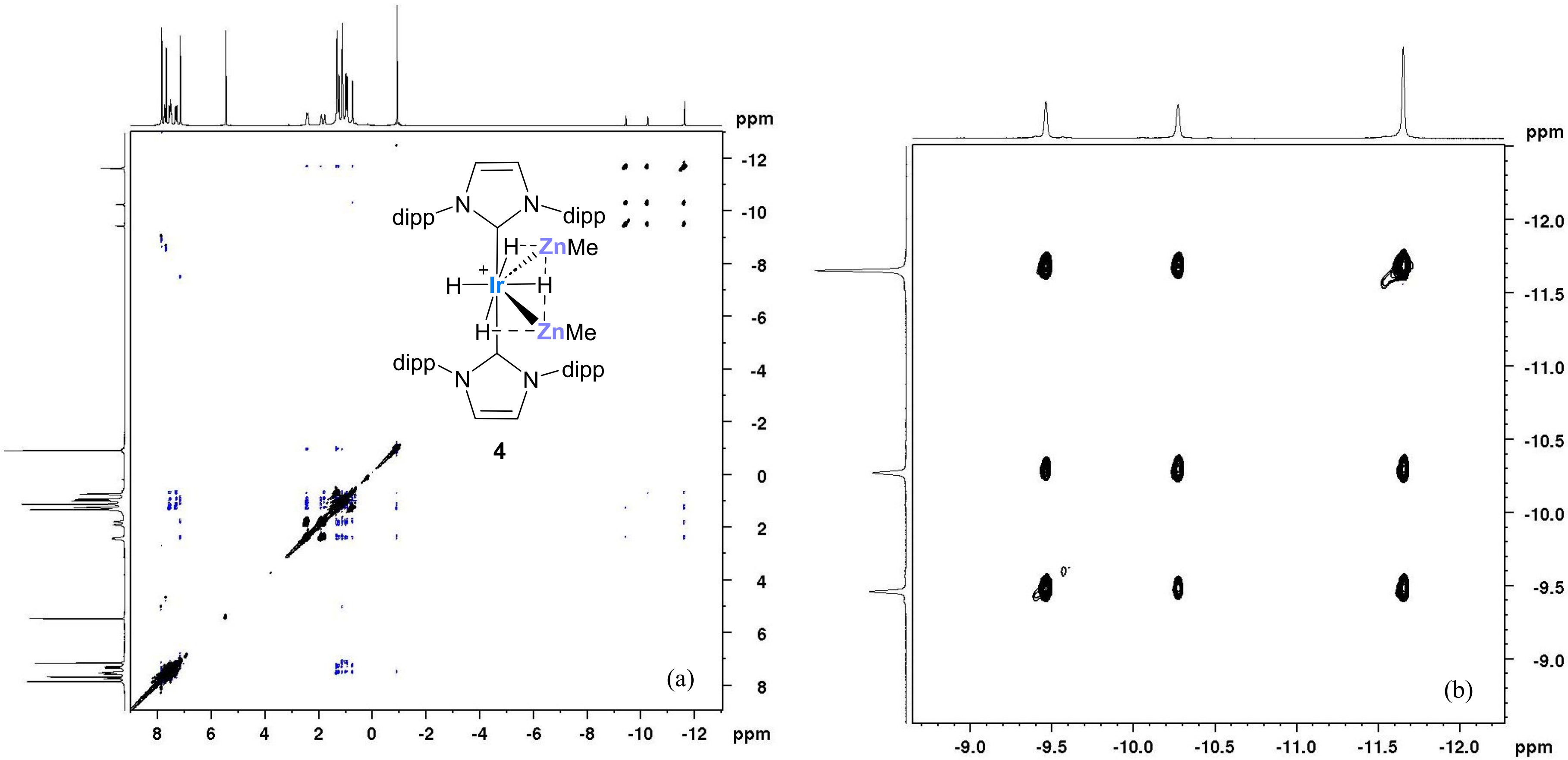

Figure S21. (a) Low temperature (223 K) ^{1}H ROESY spectrum (CD_2Cl_2, 500 MHz) of $[Ir(IPr)_2(ZnMe)_2H_4][BAr^F{}_4]$ (**4**) with an expansion of just the hydride region shown in (b).

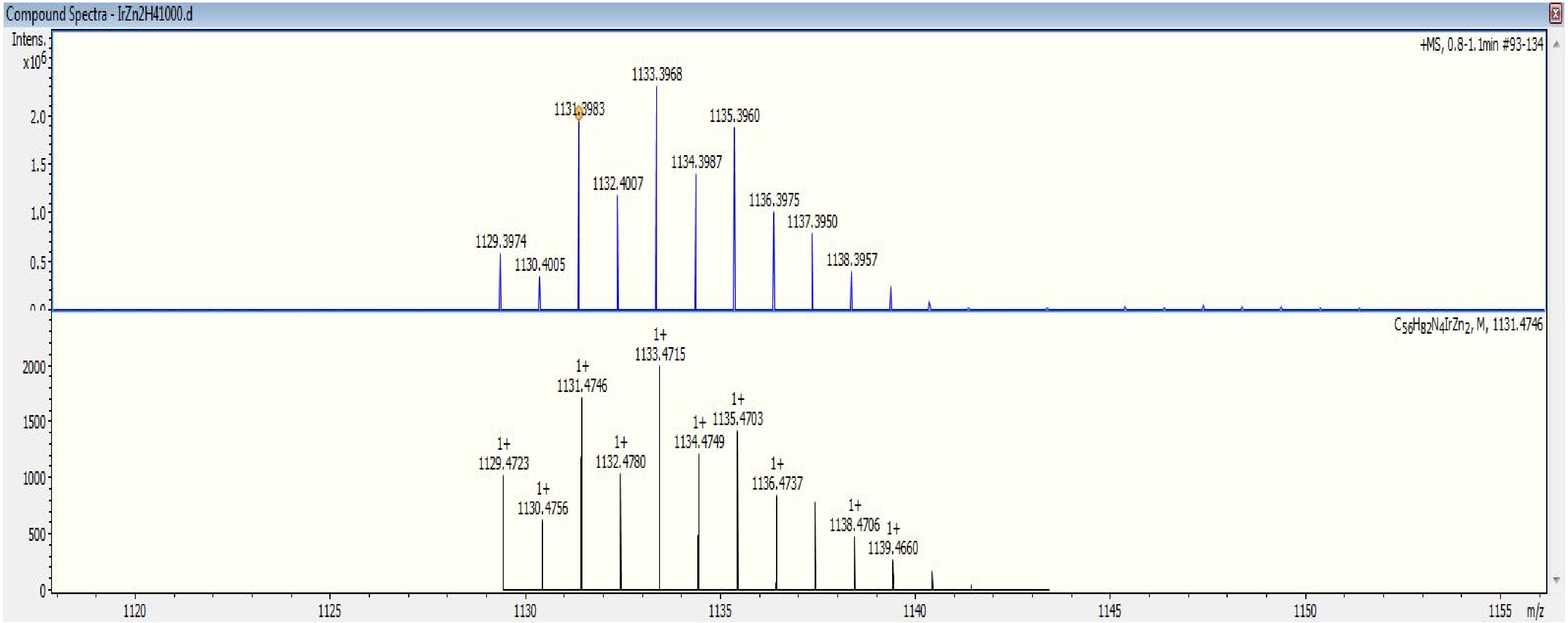

Figure S22. ESI mass spectrum of $[Ir(IPr)_2(ZnMe)_2H_4]^+$ (**4**$^+$).

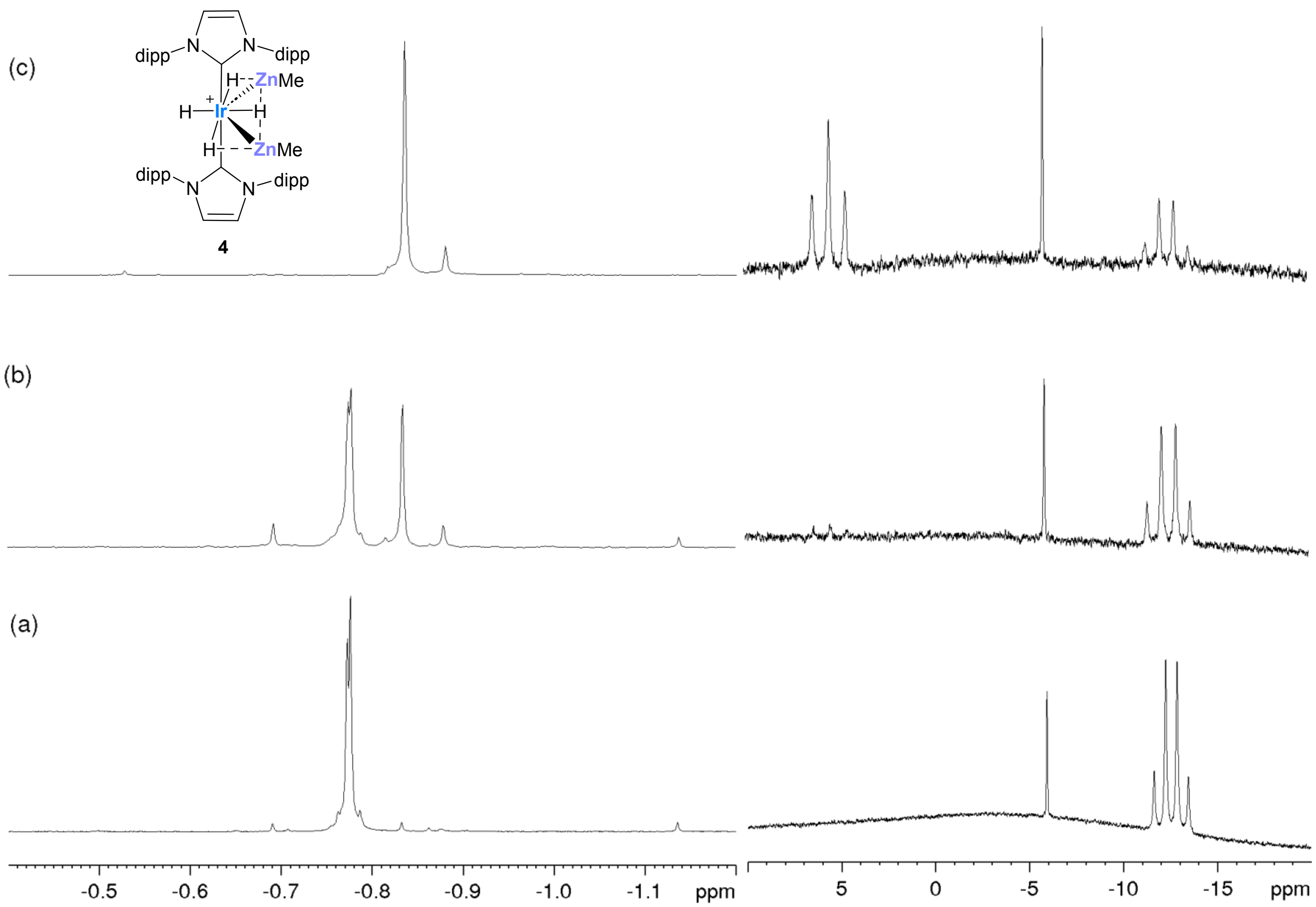

Figure S23. (Left) Zn*Me* region of the room temperature ^{1}H NMR spectrum (C_6H_5F, 500 MHz, 298 K) of the reaction of [Ir(IPr)(IPr)′(ZnMe)H][BAr$^F_4$] (**3**) with $Me_2NH{\cdot}BH_3$ after (a) 5 min, (b) 12 h and (c) 1 week showing formation of [Ir(IPr)$_2$(ZnMe)$_2$H$_4$][BAr$^F_4$] (**4**). The corresponding ^{11}B (161 MHz) spectrum (right) shows loss of $Me_2NH{\cdot}BH_3$ (δ -12) and growth of $[Me_2N{\cdot}BH_2]_2$ (δ 6). Signal at δ -6 from [BAr$^F_4$]$^-$.

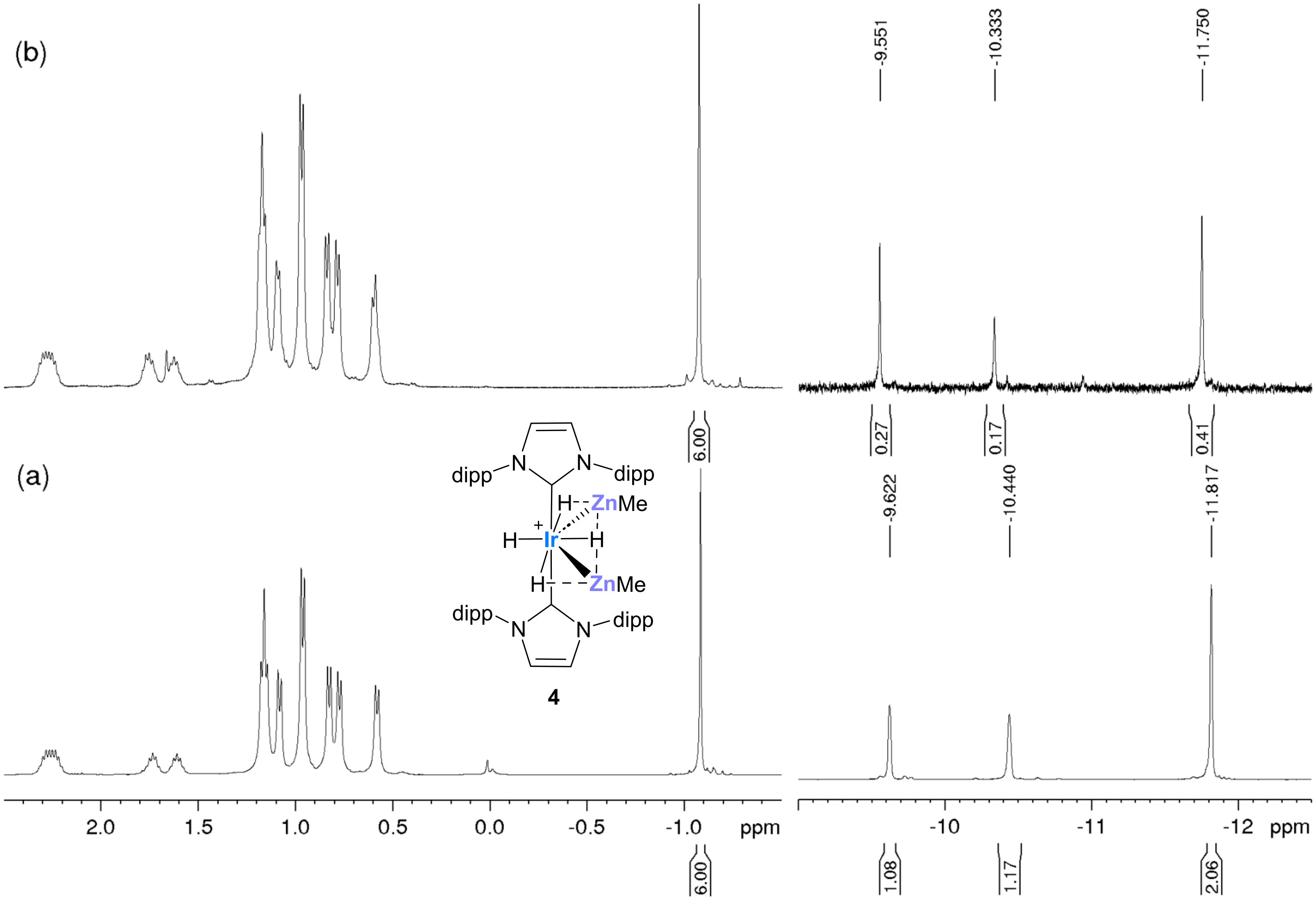

Figure S24. Alkyl (left) and Ir*H* (right) regions of the low temperature ^{1}H NMR spectra (CD_2Cl_2, 400 MHz, 210 K) of (a) $[Ir(IPr)_2(ZnMe)_2H_4][BAr^F{}_4]$ (**4**) and (b) a reaction of $[Ir(IPr)(IPr')(ZnMe)_2H][BAr^F{}_4]$ (**3**) with D_2 (1 atm).

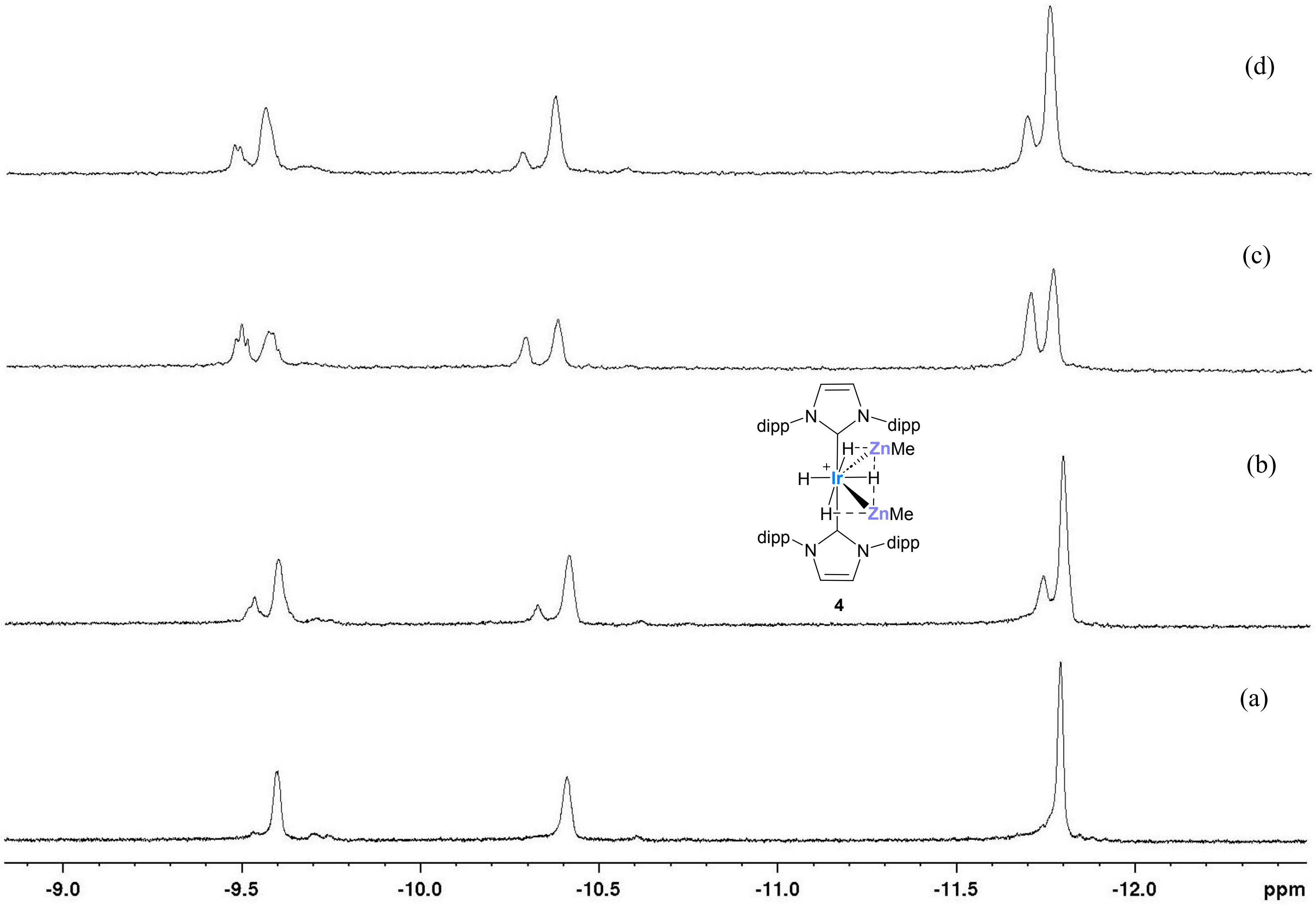
(d)
(c)
(b)
(a)
dipp
N
N
dipp
H
Zn
Me
+
H
Ir
H
H
Zn
Me
dipp
N
N
dipp
4
-9.0
-9.5
-10.0
-10.5
-11.0
-11.5
-12.0
ppm

Figure S25. Hydride region of the 210 K ^1H NMR spectrum (CD_2Cl_2, 400 MHz) recorded (a) immediately after addition of 1 atm D_2 to $[Ir(IPr)_2(ZnMe)_2H_4][BAr^F_4]$ (**4**), then after (b) 30 min at 40 ^0C and (c) 3 h at 40 ^0C. Spectrum (d) shows that degassing the sample, adding H_2 (1 atm) and heating at 40 ^0C for 30 min leads to the partial reversal of the reaction.

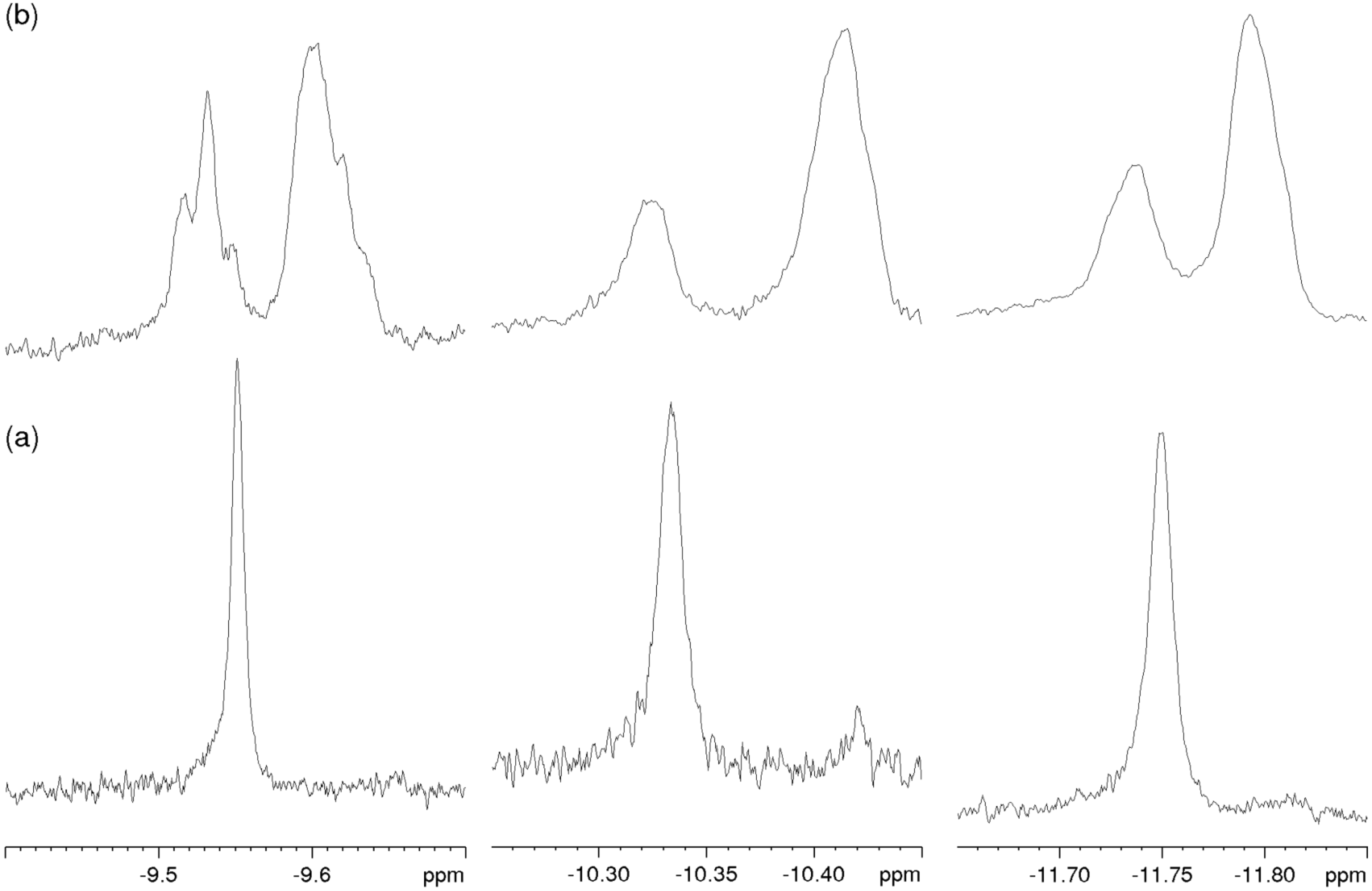

Figure S26. Overlay of hydride regions of the ^{1}H NMR spectra (CD_2Cl_2, 400 MHz, 210 K) from adding 1 atm D_2 to (a) $[Ir(IPr)(IPr')(ZnMe)_2H][BAr^F{}_4]$ (**3**) and (b) $[Ir(IPr)_2(ZnMe)_2H_4][BAr^F{}_4]$ (**4)** to emphasize the formation of what we assign as [Ir(IPr)(IPr-*d*)$(ZnMe)_2HD_3][BAr^F{}_4]$ (**4-*d*$_4$**) in the former and $[Ir(IPr)_2(ZnMe)_2H_{4-n}D_n][BAr^F{}_4]$ (n = 1-3) in the latter.

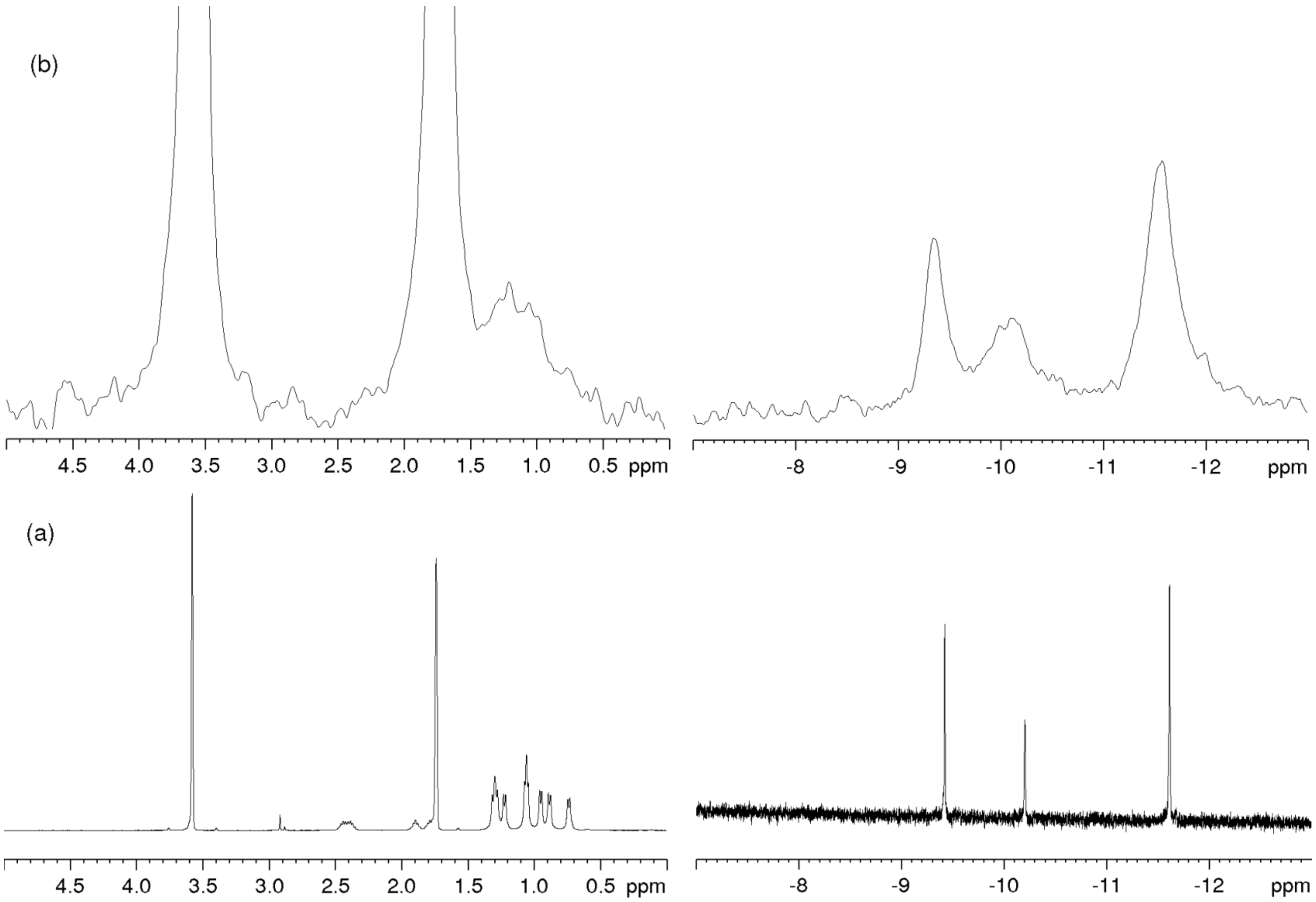

Figure S27. (a) Alkyl and hydride regions of the ^{1}H NMR spectrum (400 MHz, 210 K) of [Ir(IPr)(IPr')(ZnMe)$_2$H][BAr^F$_4$] (**3**) + D_2 in THF-d_8. (b) ^{2}H NMR spectrum (61 MHz, 210 K) of the same experiment repeated in THF-h_8. Deuterium is clearly incorporated at Ir in (b), while there is also evidence for deuteration at the dipp Me resonances at ca. δ 1-1.5.

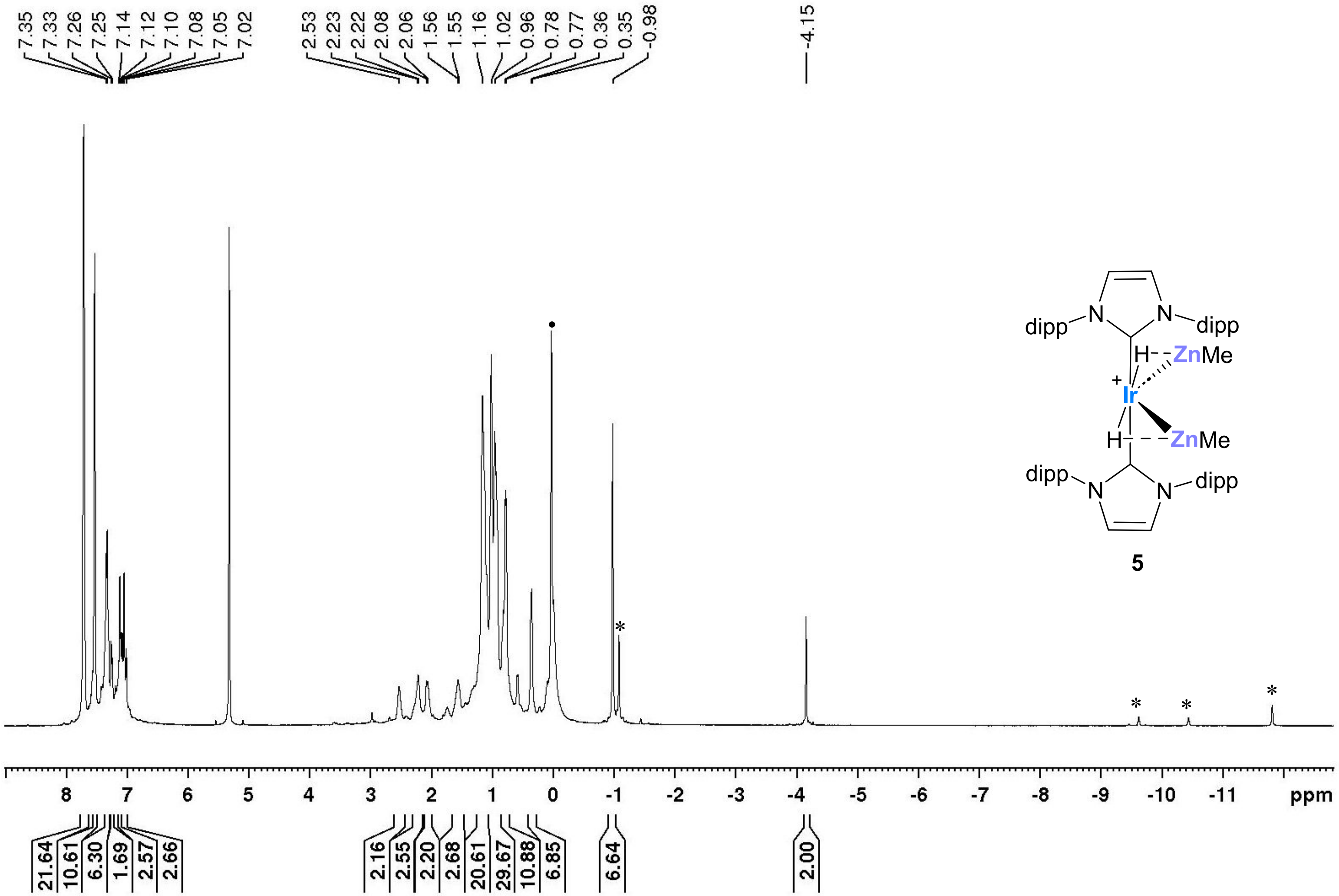

Figure S28. Low temperature ¹H NMR spectrum (CD_2Cl_2, 400 MHz, 201 K) of $[Ir(IPr)_2(ZnMe)_2H_2][BAr^F_4]$ (**5**) generated upon heating **4** at 60 ^{0}C for 2 weeks under vacuum (• = silicone grease, * = residual **4**).

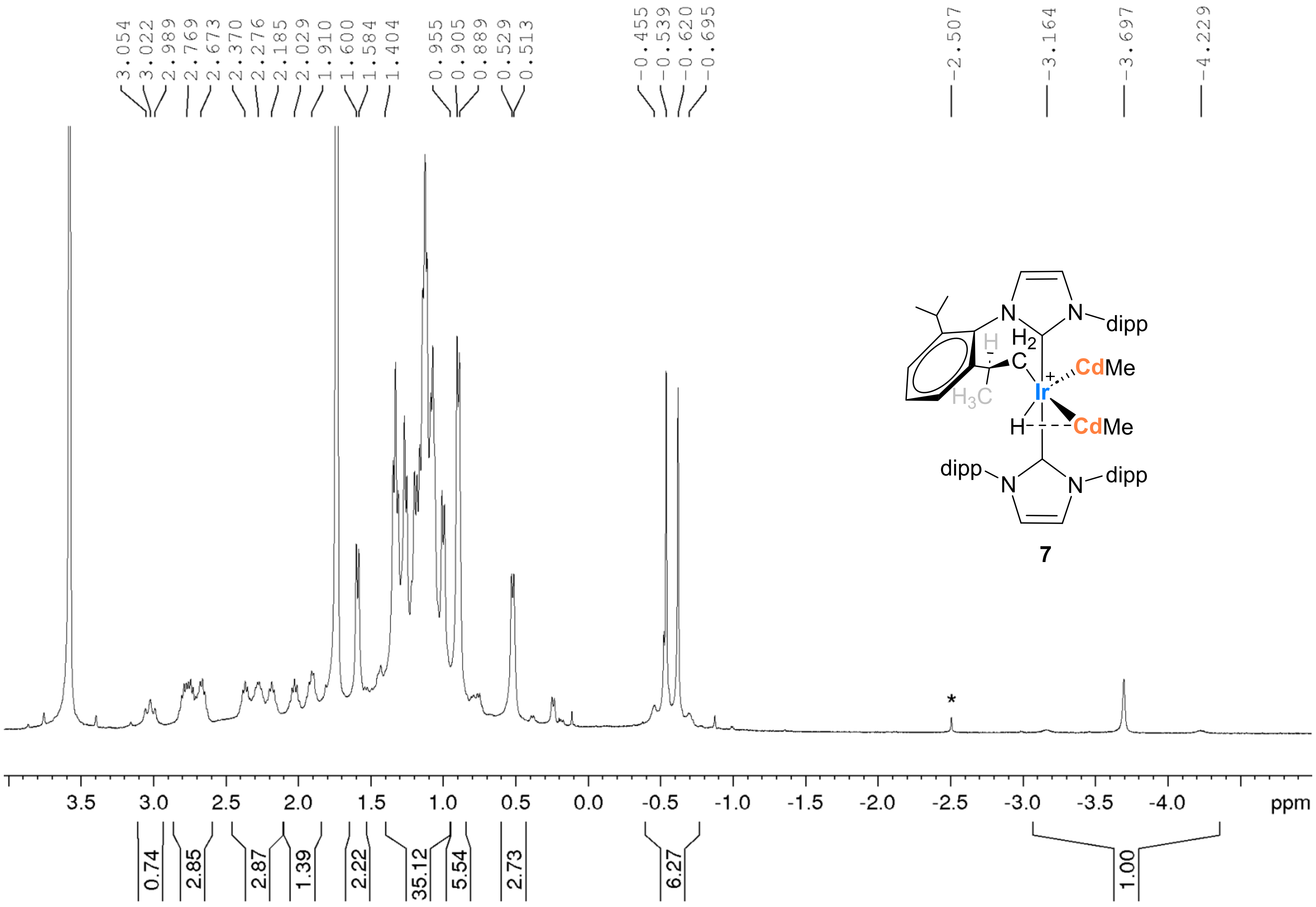

Figure S29. Low frequency region of the low temperature ^{1}H NMR spectrum (THF-d_8, 500 MHz, 228 K) of recrystallized [Ir(IPr)(IPr')(CdMe)$_2$H][BAr$^F{}_4$] (**7**) (* = [Ir(IPr)$_2$(CdMe)$_2$H$_2$][BAr$^F{}_4$] (**9**)).

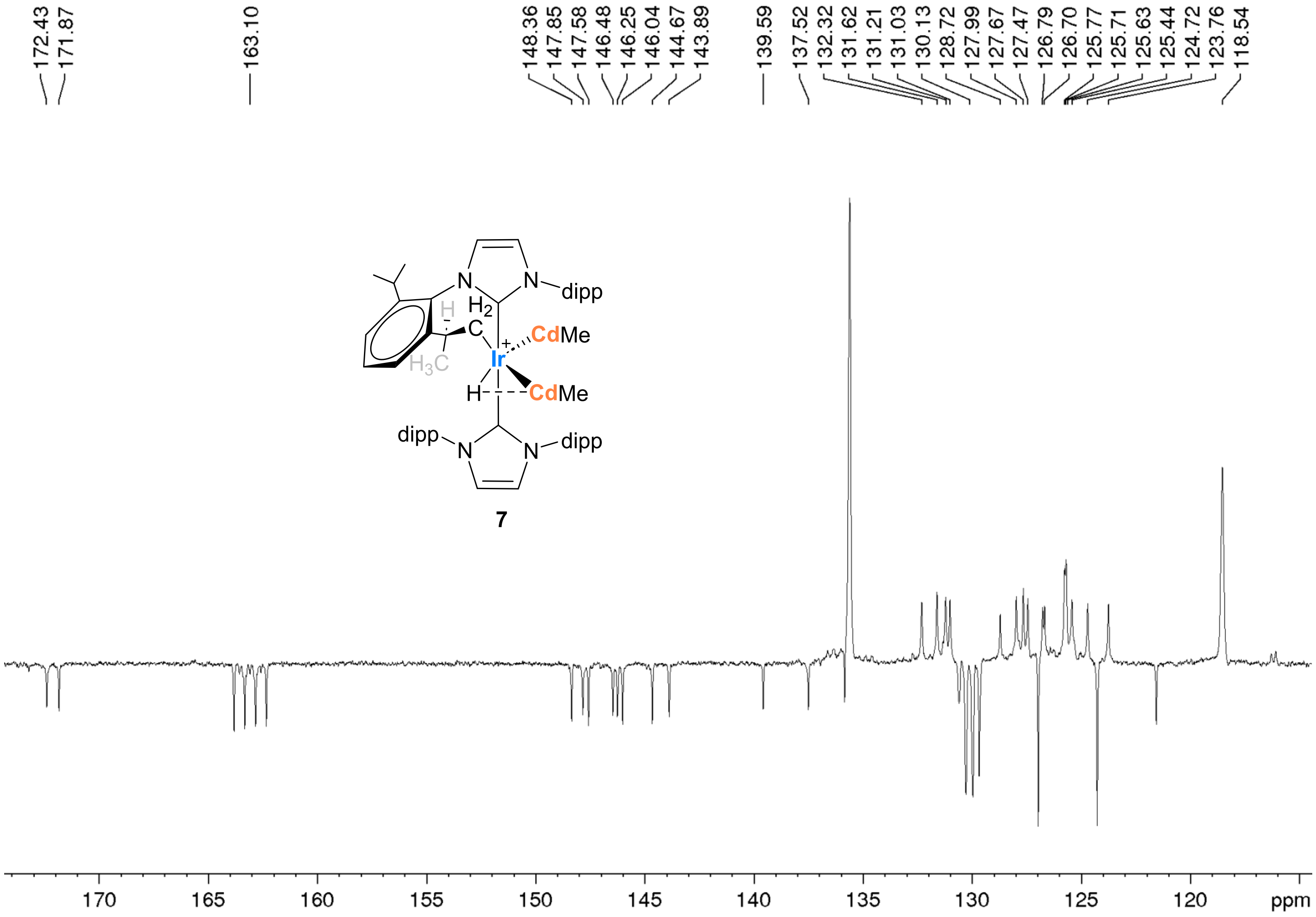

Figure S30. High frequency region of the room temperature $^{13}C\{^1H\}$ DEPTQ NMR spectrum (THF-d_8, 101 MHz, 298 K) of $[Ir(IPr)(IPr')(CdMe)_2H][BAr^F{}_4]$ (**7**).

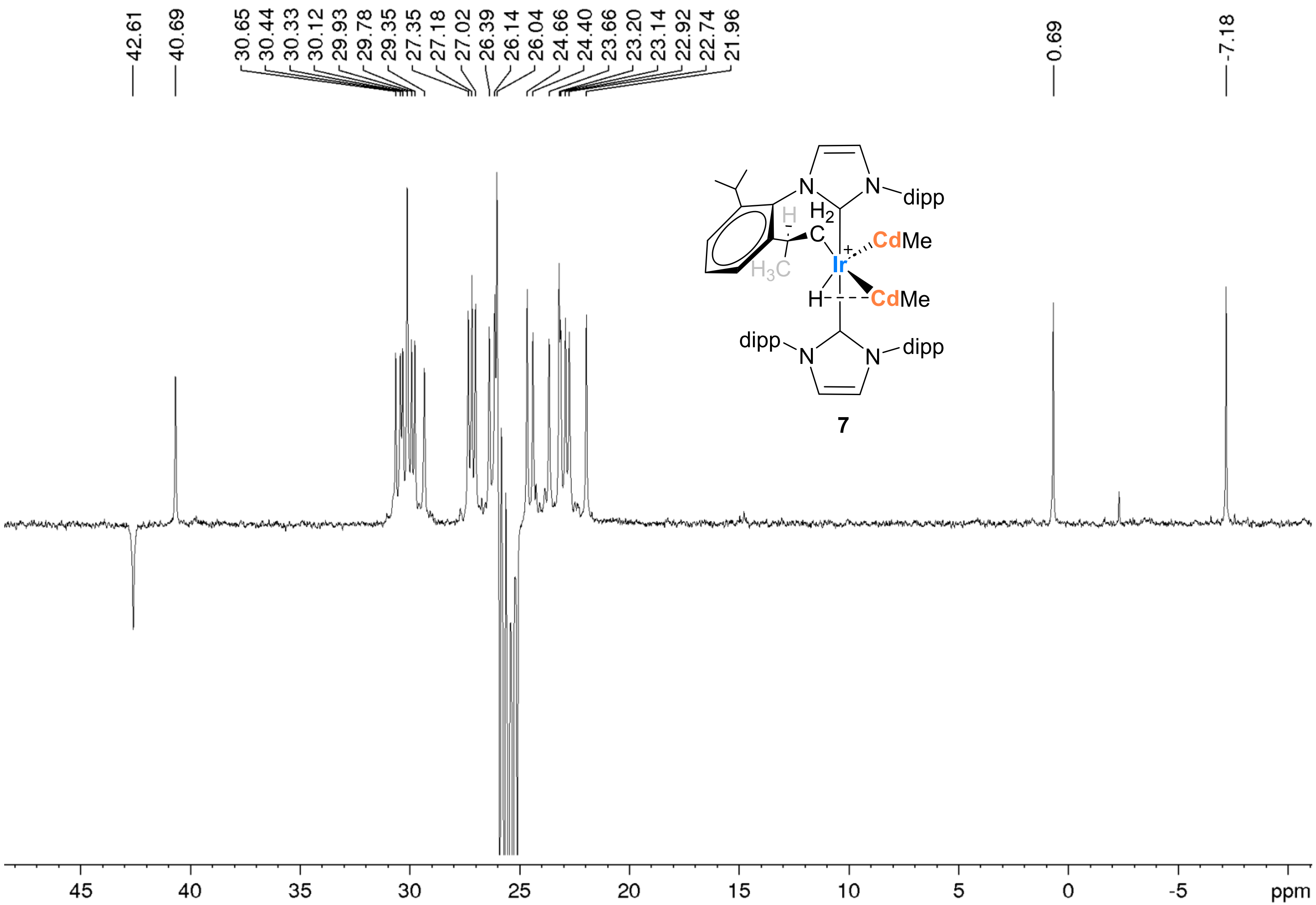

Figure S31. Low frequency region of the room temperature $^{13}C\{^1H\}$ DEPTQ NMR spectrum (THF-d_8, 101 MHz, 298 K) of $[Ir(IPr)(IPr')(CdMe)_2H][BAr^F{}_4]$ (**7**).

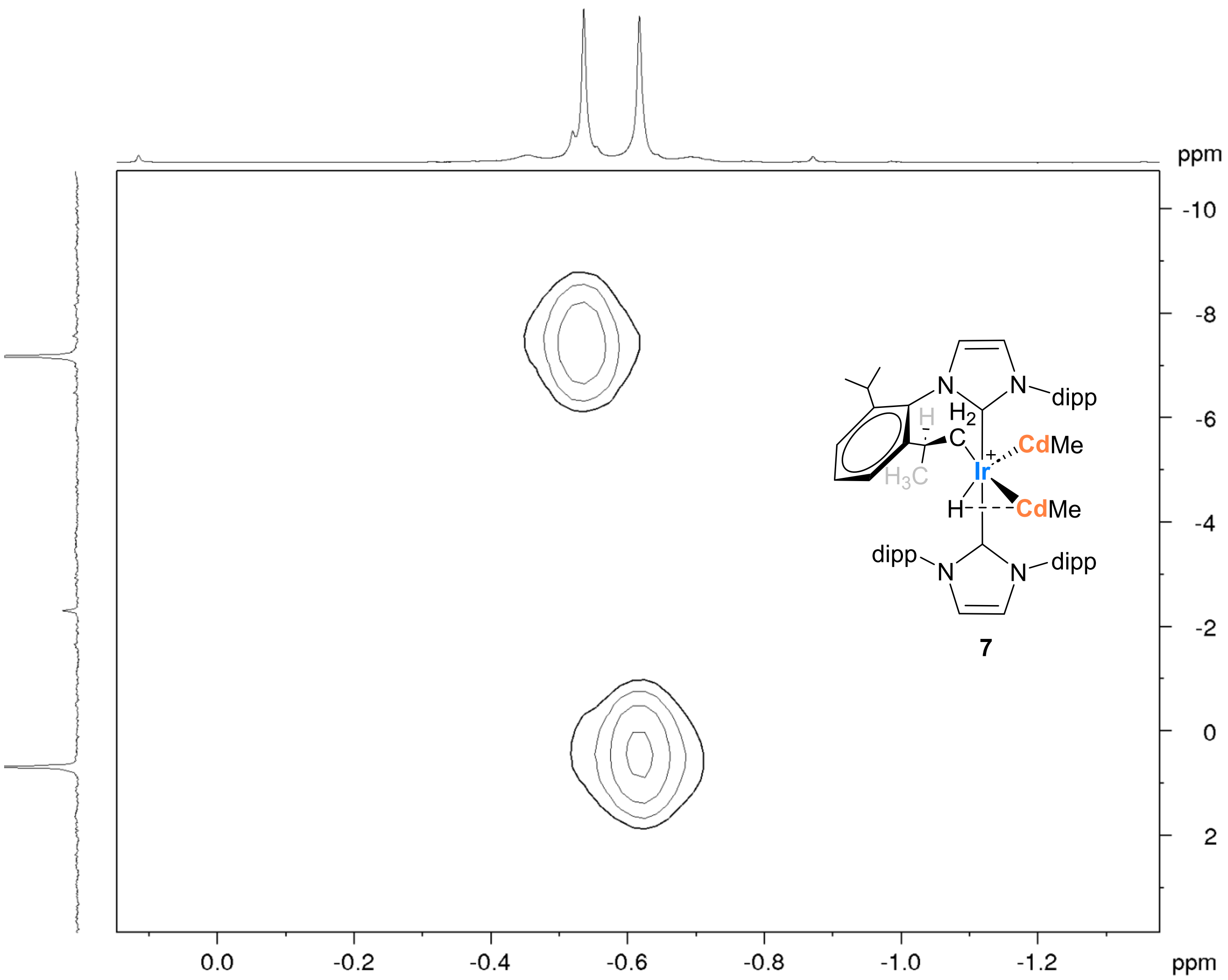

Figure S32. Partial low temperature ^{1}H-^{13}C HSQC spectrum (THF-d_8, 228 K) of [Ir(IPr)(IPr')(CdMe)$_2$H][BAr^F$_4$] (**7)** highlighting the Cd*Me* correlations.

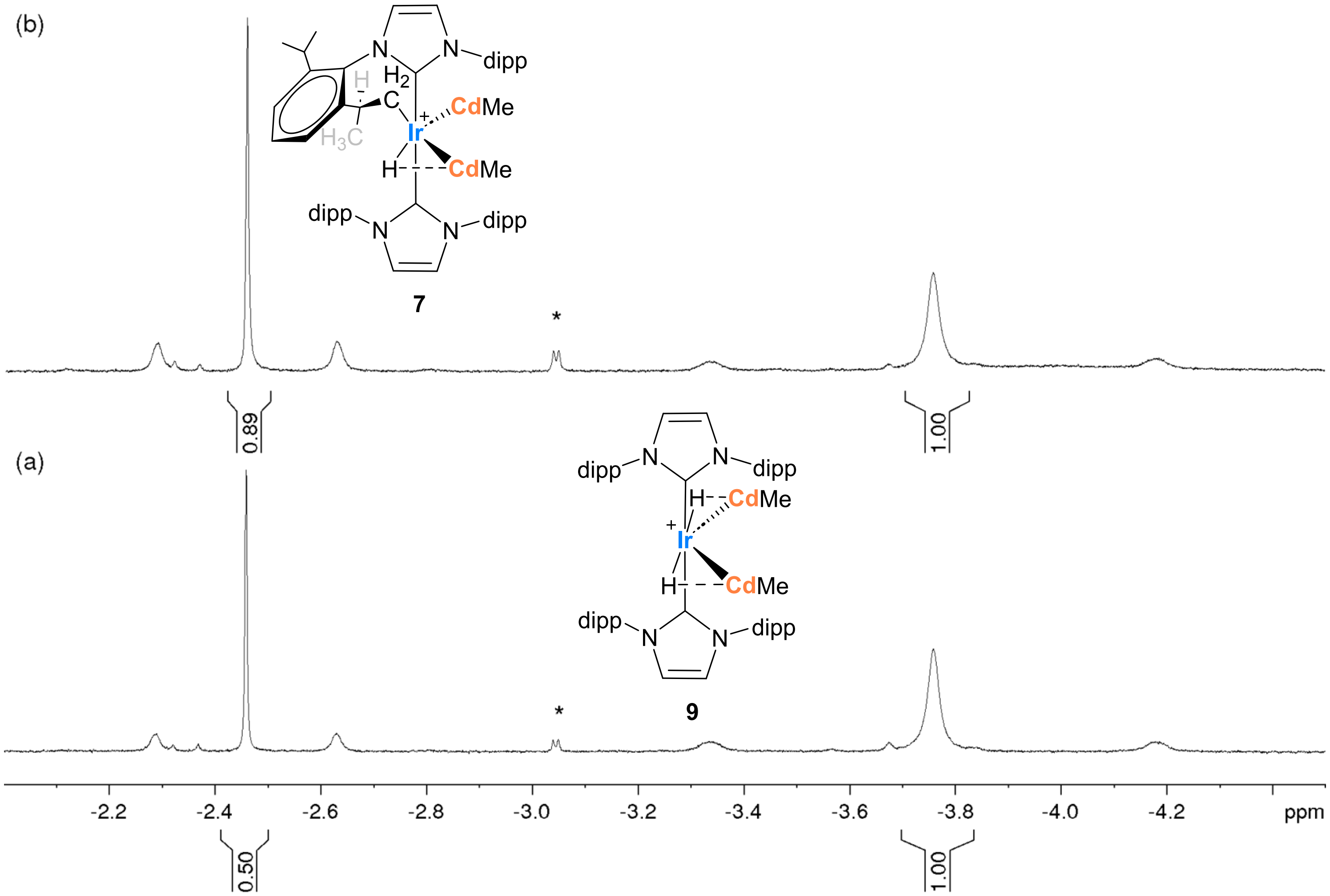

Figure S33. Hydride region of room temperature ^{1}H NMR spectra (THF-d_8, 500 MHz, 298 K) of separate samples (a and b) of recrystallized [Ir(IPr)(IPr')(CdMe)$_2$H][BAr^F$_4$] (**7**, ca. δ -3.7) showing the variation in amounts of [Ir(IPr)$_2$(CdMe)$_2$H$_2$][BAr^F$_4$] (**9**, ca. δ -2.4) (* = unknown impurity).

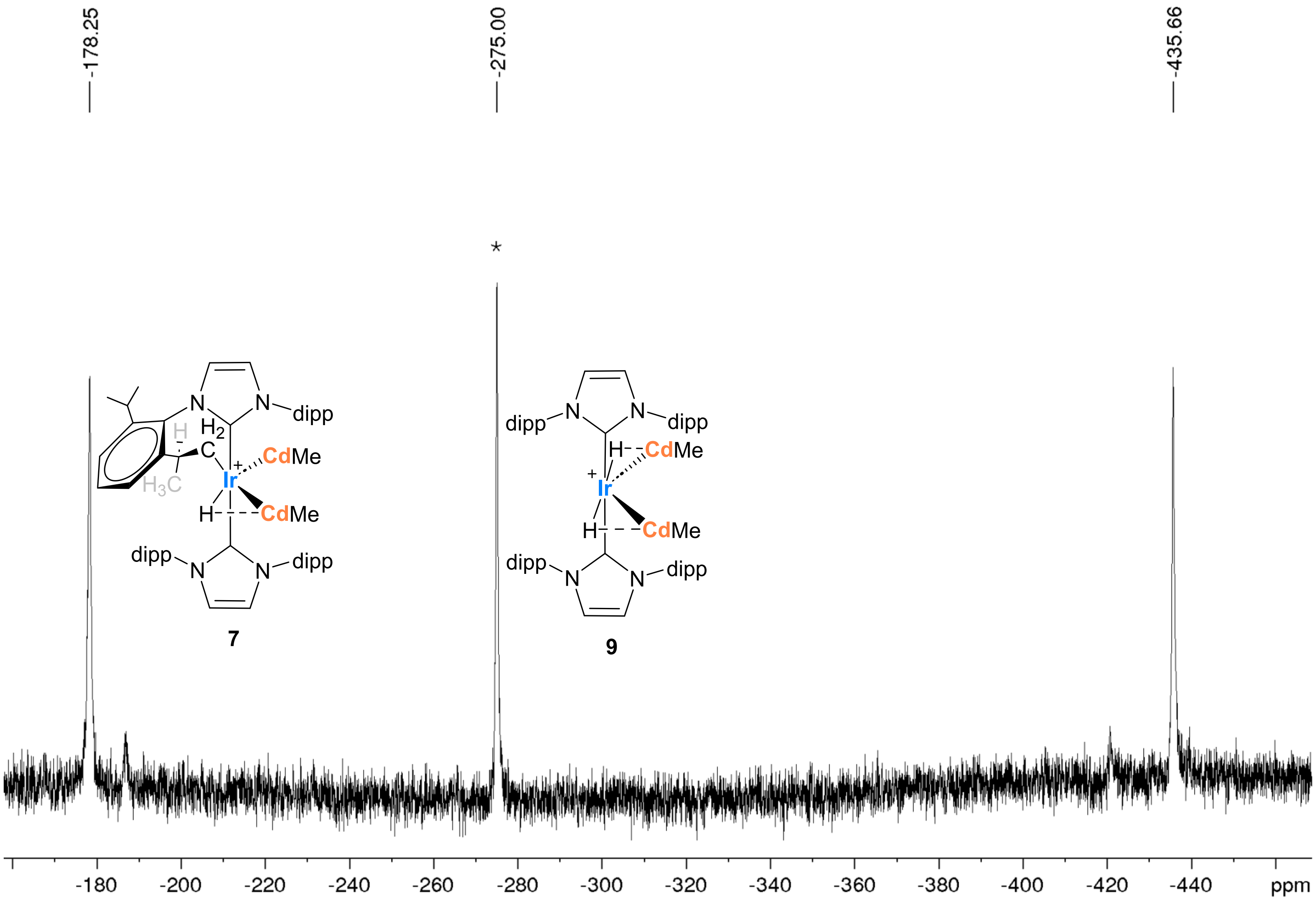

Figure S34. Low temperature $^{113}Cd\{^1H\}$ NMR spectrum (THF-d_8, 111 MHz, 228 K) of $[Ir(IPr)(IPr')(CdMe)_2H][BAr^F{}_4]$ (**7**) (* = $[Ir(IPr)_2(CdMe)_2H_2][BAr^F{}_4]$ (**9**)).

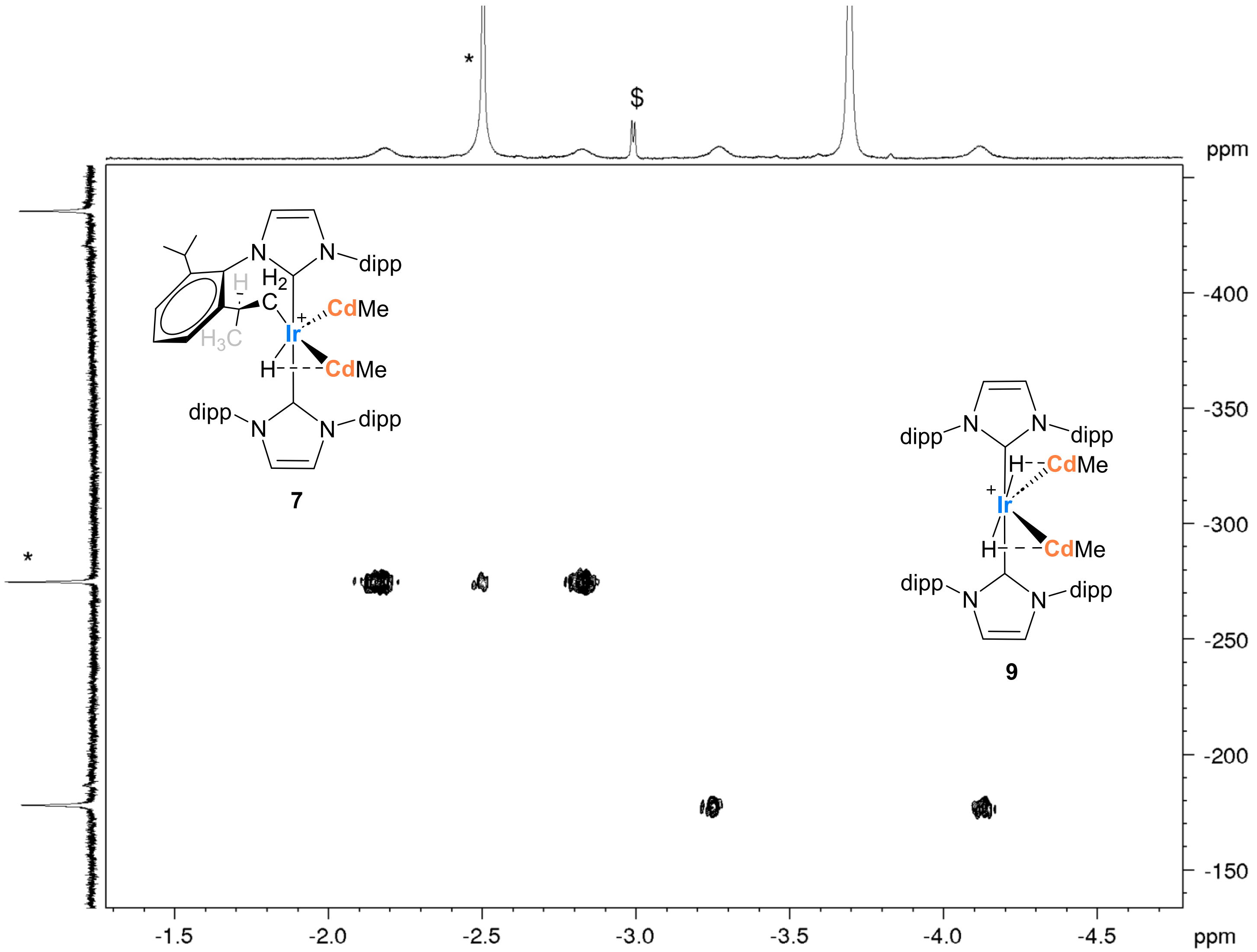

Figure S35. Partial low temperature ^{1}H-^{113}Cd HMBC spectrum (THF-d_8, 228 K) of $[Ir(IPr)(IPr')(CdMe)_2H][BAr^F{}_4]$ (**7**) (* = $[Ir(IPr)_2(CdMe)_2H_2][BAr^F{}_4]$ (**9**), $ = unknown impurity) showing *H*Ir*Cd* correlations (correlation optimized for a 200 Hz $^{2}J_{HCd}$ coupling).

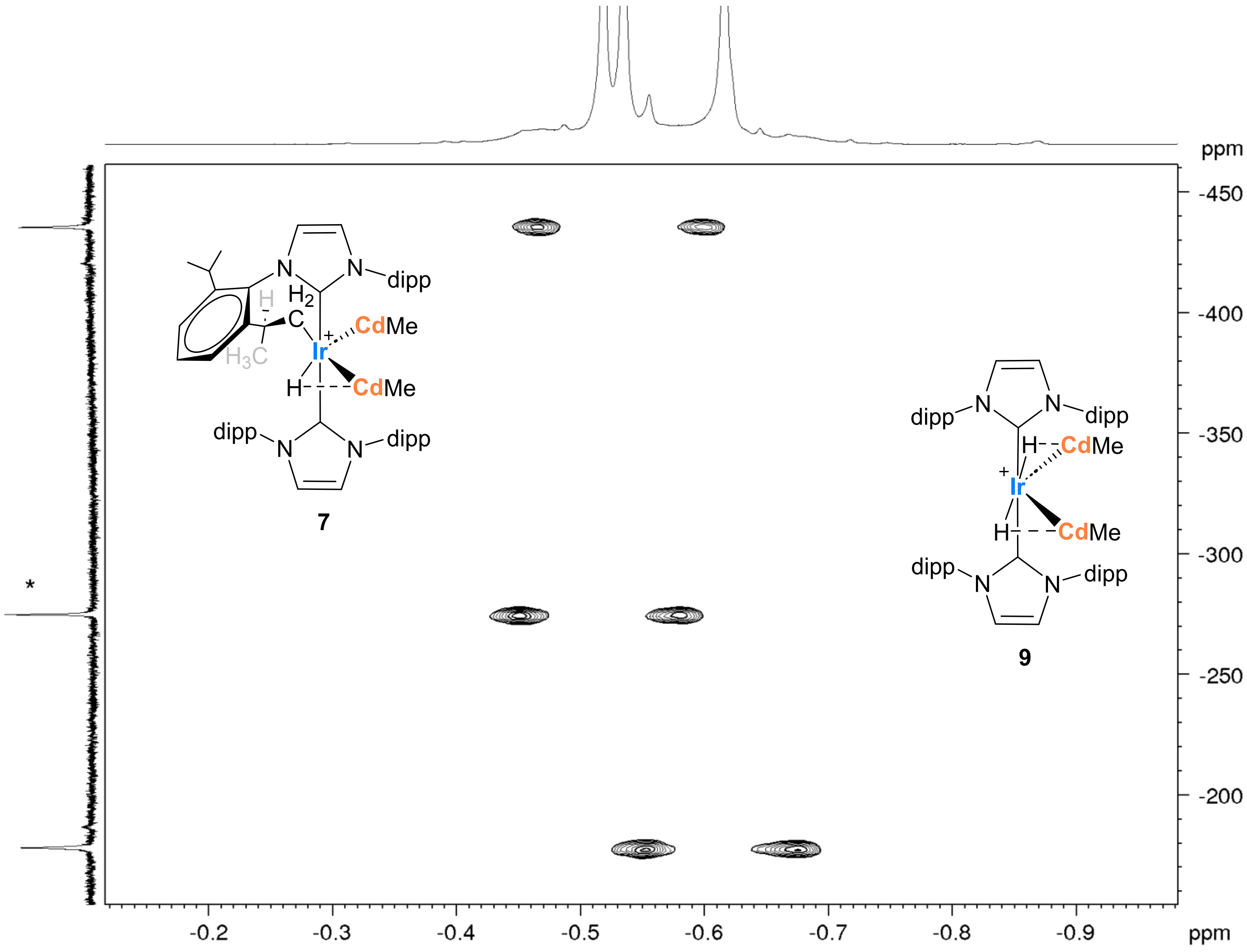

Figure S36. Low temperature ^{1}H-^{113}Cd HMBC spectrum (THF-d_8, 228 K) of $[Ir(IPr)(IPr')(CdMe)_2H][BAr^F{}_4]$ (**7**) (* = $[Ir(IPr)_2(CdMe)_2H_2][BAr^F{}_4]$ (**9**)) showing correlations of Cd*Me* resonances (correlation optimized for a 200 Hz $^{2}J_{HCd}$ coupling).

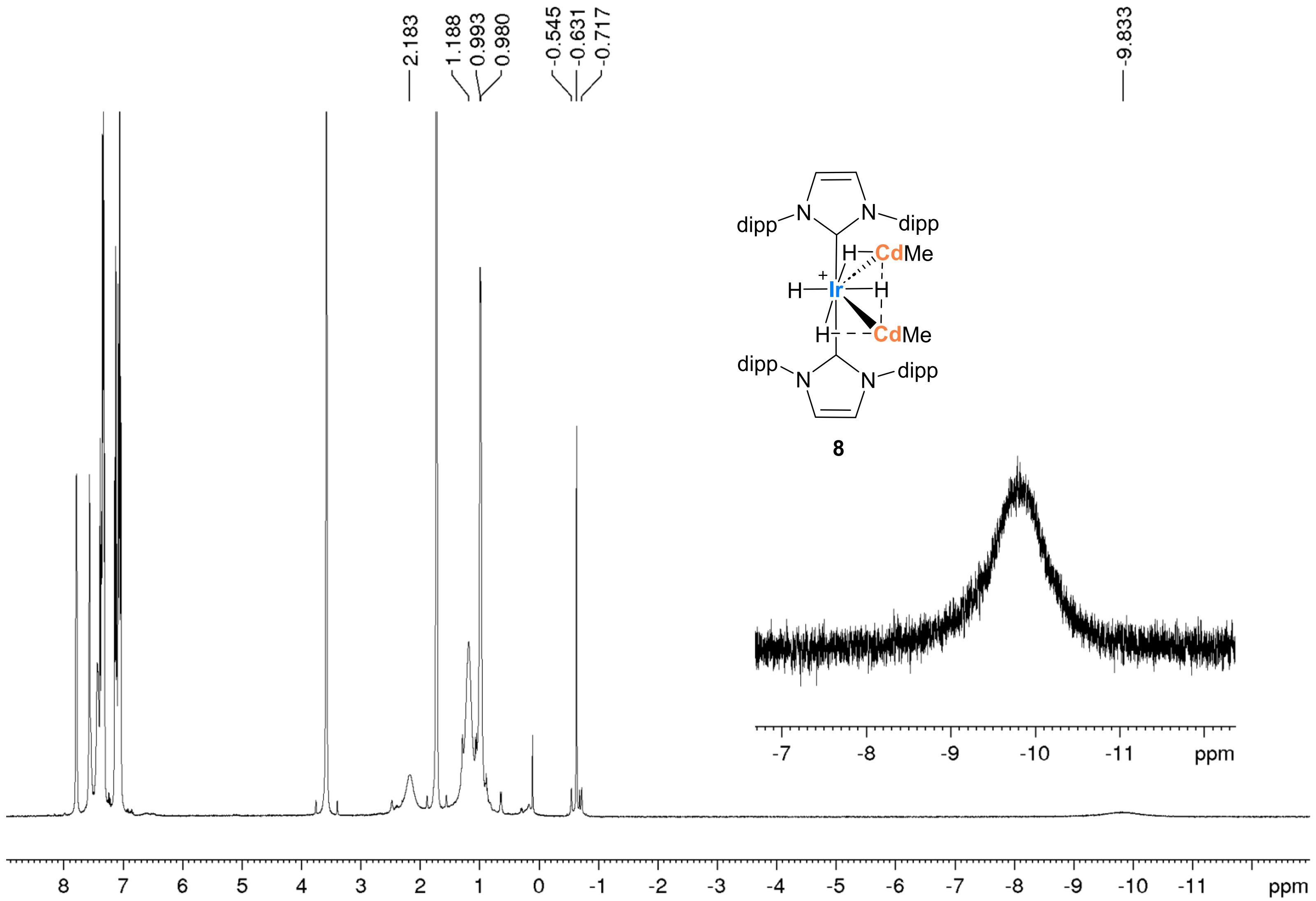

Figure S37. Room temperature ^{1}H NMR spectrum (THF-d_8, 400 MHz, 298 K) of $[Ir(IPr)_2(CdMe)_2H_4][BAr^F{}_4]$ (**8**). Expansion of the hydride signal shown in inset.

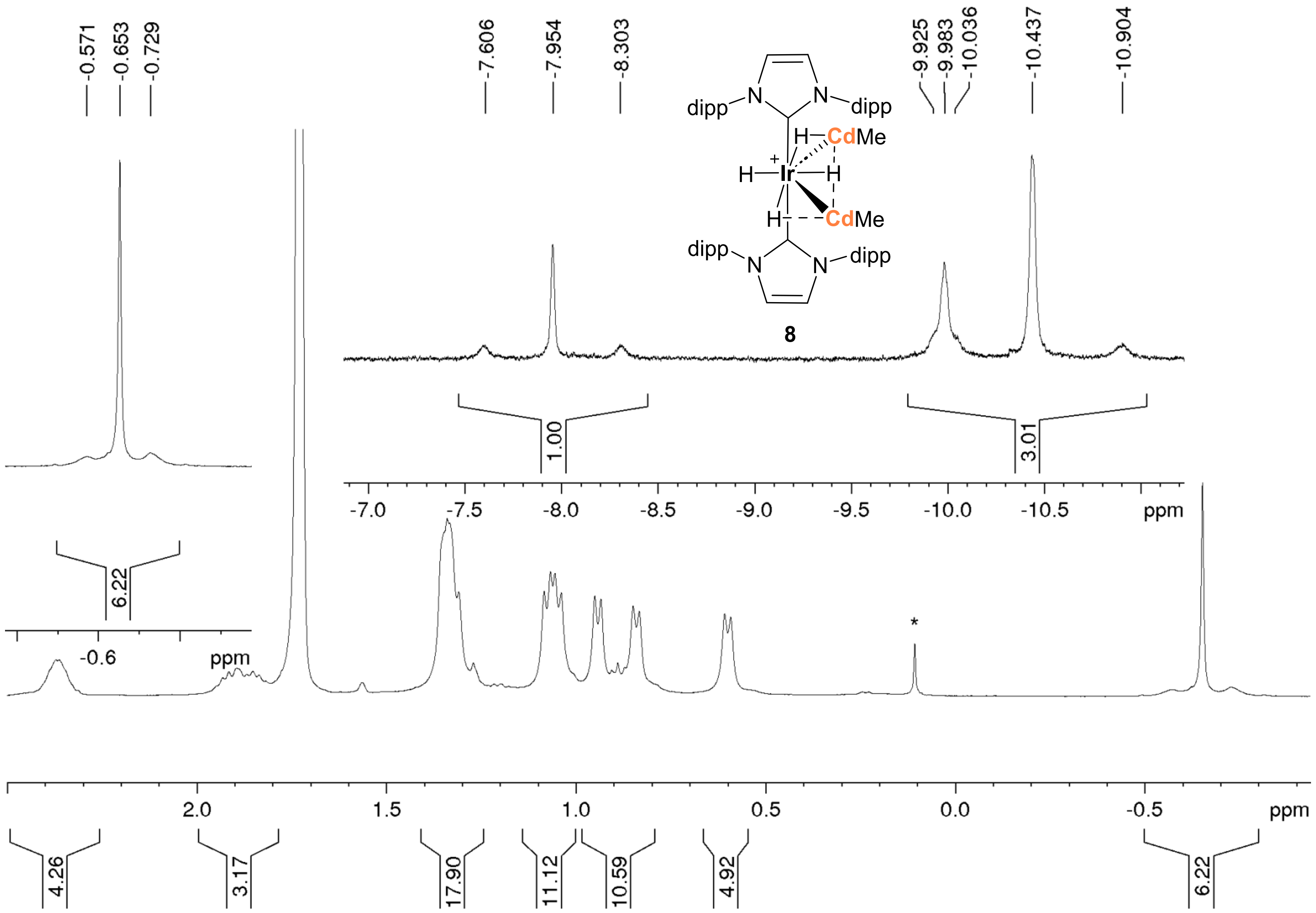

Figure S38. Low frequency region of the low temperature ^{1}H NMR spectrum (THF-d_8, 400 MHz, 228 K) of $[Ir(IPr)_2(CdMe)_2H_4][BAr^F_4]$ (**8**). Expansions of the Cd*Me* resonance and Ir*H* signals shown in insets (* = silicone grease).

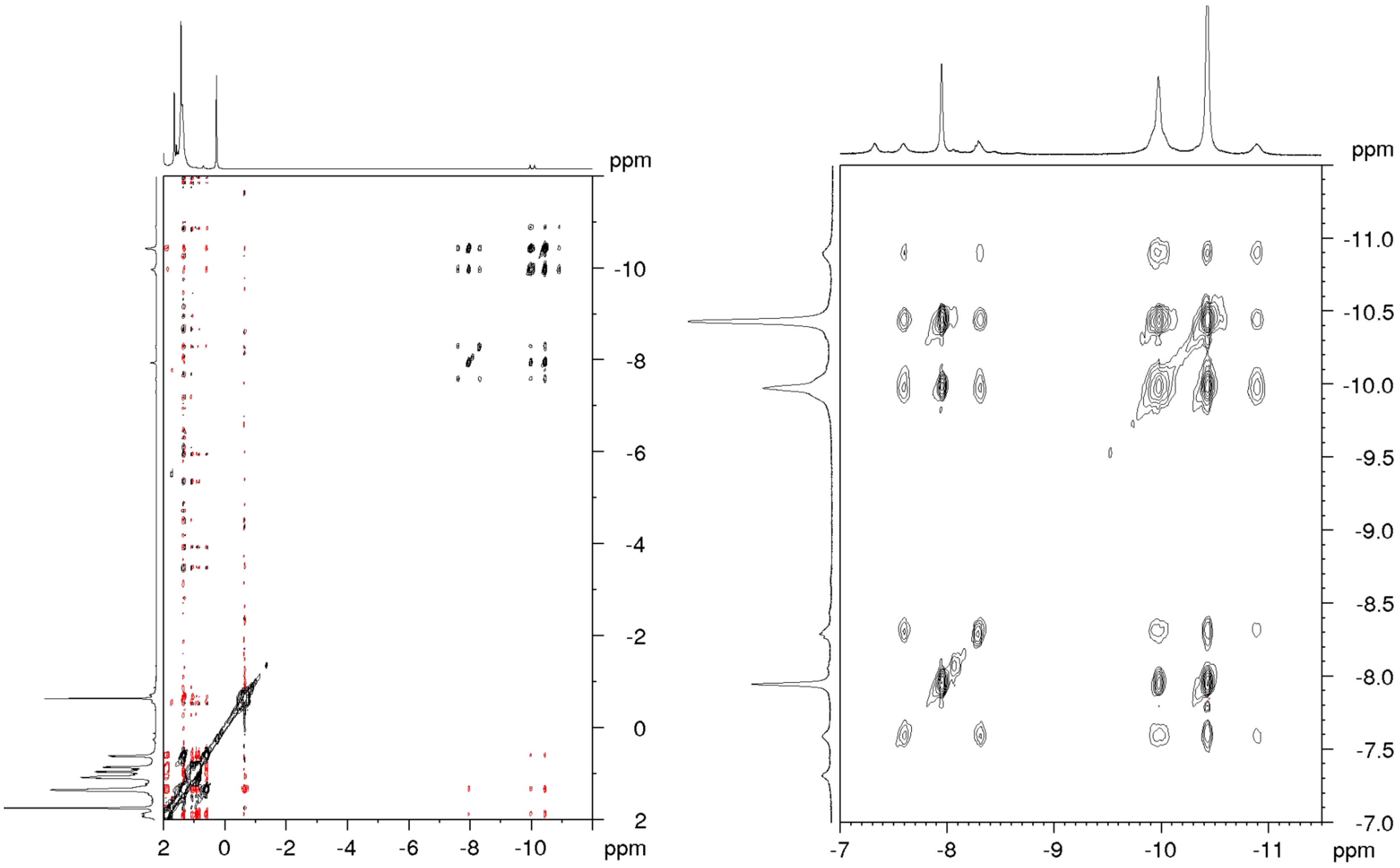

Figure S39. Low temperature (228 K) ^{1}H ROESY spectrum (THF-d_8, 400 MHz) of [Ir(IPr)$_2$(CdMe)$_2$H$_4$][BAr$^F{}_4$] (**8**) with an expansion of just the hydride region shown on the right.

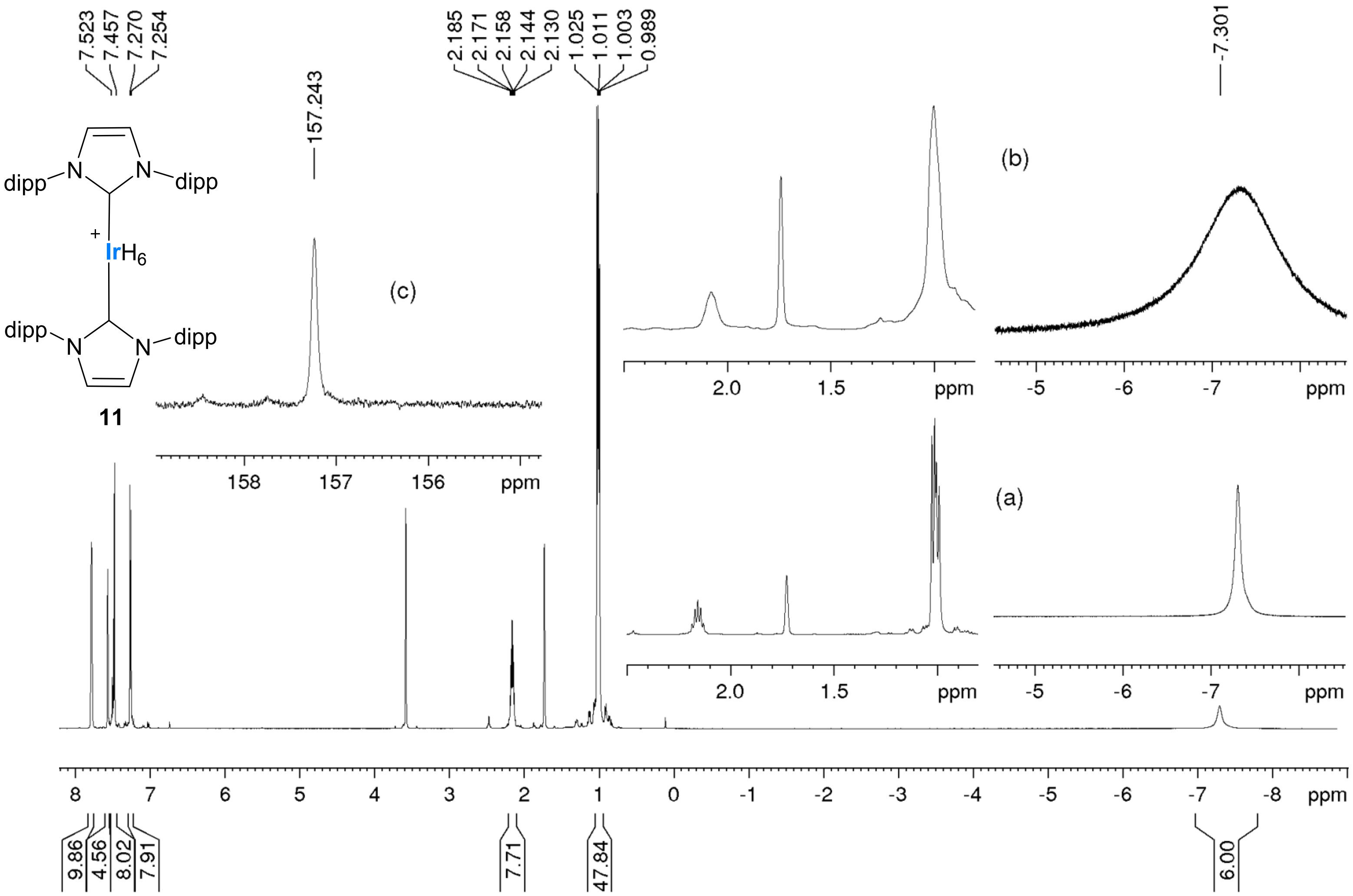

Figure S40. ^{1}H NMR spectrum (THF-d_8, 400 MHz, 298 K) of $[Ir(IPr)_2H_6][BAr^F{}_4]$ (**11**). Expansions of dipp and Ir*H* regions at 298 and 188 K are shown in insets (a) and (b) respectively. Inset (c) shows the Ir-C_{NHC} resonance in the $^{13}C\{selective\text{-}^1H\}$ NMR spectrum (THF-d_8, 126 MHz, 278 K); notable is the absence of splitting (c.f. Figures S3 and S18).

Fluxionality of 11. The fluxionality of **11** arises from both inter- and intramolecular exchange processes. Thus, ^{1}H NMR spectra measured under H_2 showed a (broad) signal for free H_2 only at <248 K, consistent with intermolecular exchange with H_2. This resonance sharpened upon further cooling; full width at half-height (FWHH) in CD_2Cl_2: 46 Hz at 248 K, 2 Hz at 208 K. Exposure of a THF-d_8 solution of **11** to D_2 resulted in H/D exchange, with a broad, shifted hydride resonance ca. 30% of the intensity of the starting material observed by ^{1}H NMR spectroscopy within ca. 1 h. Upon cooling to 248 K, separate hydride resonances for the $[IrH_5D]^+$ (**11-*d*1**), $[IrH_4D_2]^+$ (**11-*d*2**), $[IrH_3D_3]^+$ (**11-*d*3**) and $[IrH_2D_4]^+$ (**11-*d*4**) isotopomers appeared, all of which were considerably high frequency shifted relative to **11** ($\Delta\delta$ (= δ_{dn} - δ_{d0}) of +130 ppb, +230 ppb, +310 ppb and +330 ppb respectively; Figure S41).[10-16] At 218 K, a single broad and somewhat asymmetric resonance, assigned to $[IrHD_5]^+$ (**11-*d*5**), was detected, which was shifted even further downfield ($\Delta\delta$ = ca. +380 ppb). Both a sharp(ish) singlet for H_2 (δ 4.56) and a 1:1:1 triplet (J = 42.8 Hz) for HD (δ 4.52) were now also apparent (Figure S42). After degassing and replenishing with D_2, only **11-*d*5** remained (δ -6.97; $\Delta\delta$ = +340 ppb). There was no J_{HD} splitting on any of the resonances at any temperature, although peak linewidths did increase with higher deuterium content (Figure S42),[17] consistent with the presence of unresolved coupling.

The magnitude of the isotopomer shifts is too great to be attributable to intrinsic isotope effects,[18] and by comparison to the literature, suggests either (a) a rapid equilibrium involving two distinct species[19] or (b) a non-statistical distribution of deuterium across dihydrogen and hydride ligands.[14] Support for the latter comes from the approximate alignment of the ^{2}H NMR resonance of the $[IrD_6]^+$ isotopomer with the ^{1}H resonance of $[IrH_6]^+$ (both spectra measured in THF-h_8) as well as the shift to lower frequency of $[IrHD_5]^+$, $[IrH_2D_4]^+$ etc isotopomers in the ^{2}H NMR spectrum (Figure S43).

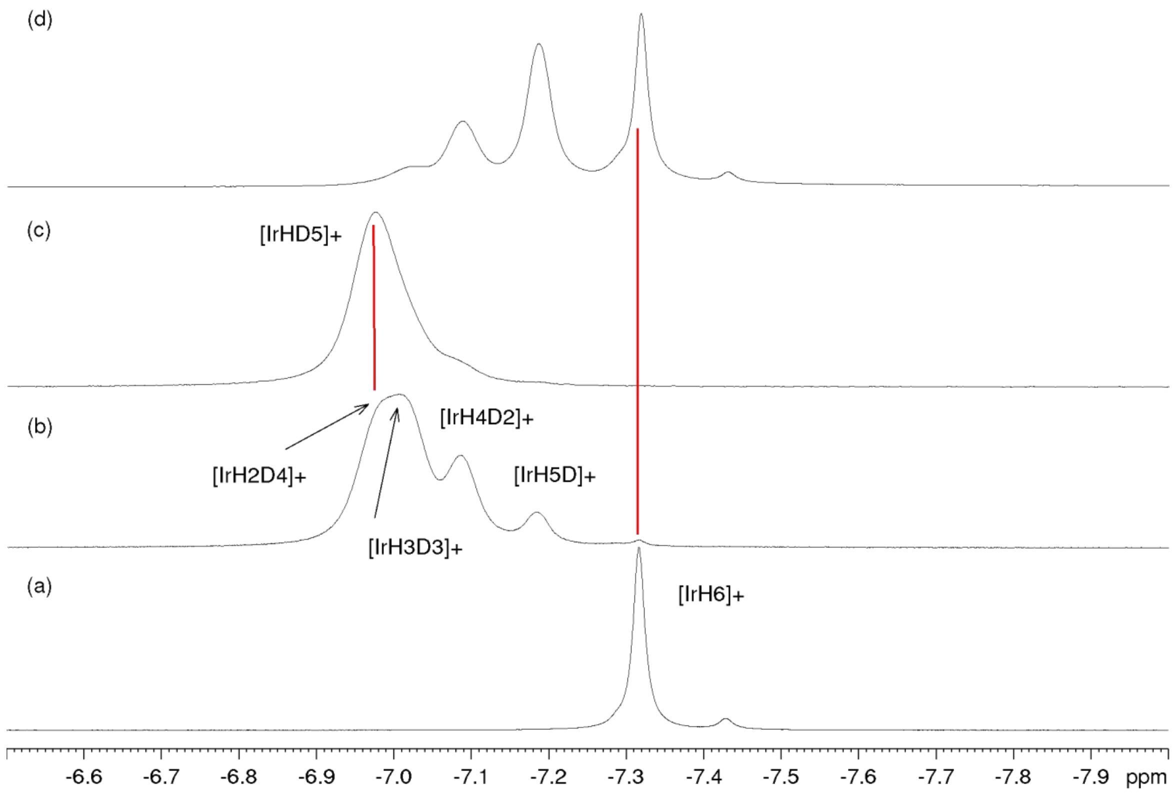

Figure S41. Hydride region of the low temperature ^1H NMR spectrum (THF-d_8, 500 MHz, 248 K) of (a) $[Ir(IPr)_2H_6][BAr^F{}_4]$ (**11**), (b) 2 h after addition of 1 atm D_2, (c) after then standing overnight, degassing and replenishing with additional D_2 and (d) 2 h after degassing and addition of 1 atm H_2. The red lines highlight (i) the slight shift to higher frequency between (b) to (c) that suggests formation of $[Ir(IPr)_2HD_5][BAr^F{}_4]$ and (ii) reformation of **11** in (d) through comparison to (a).

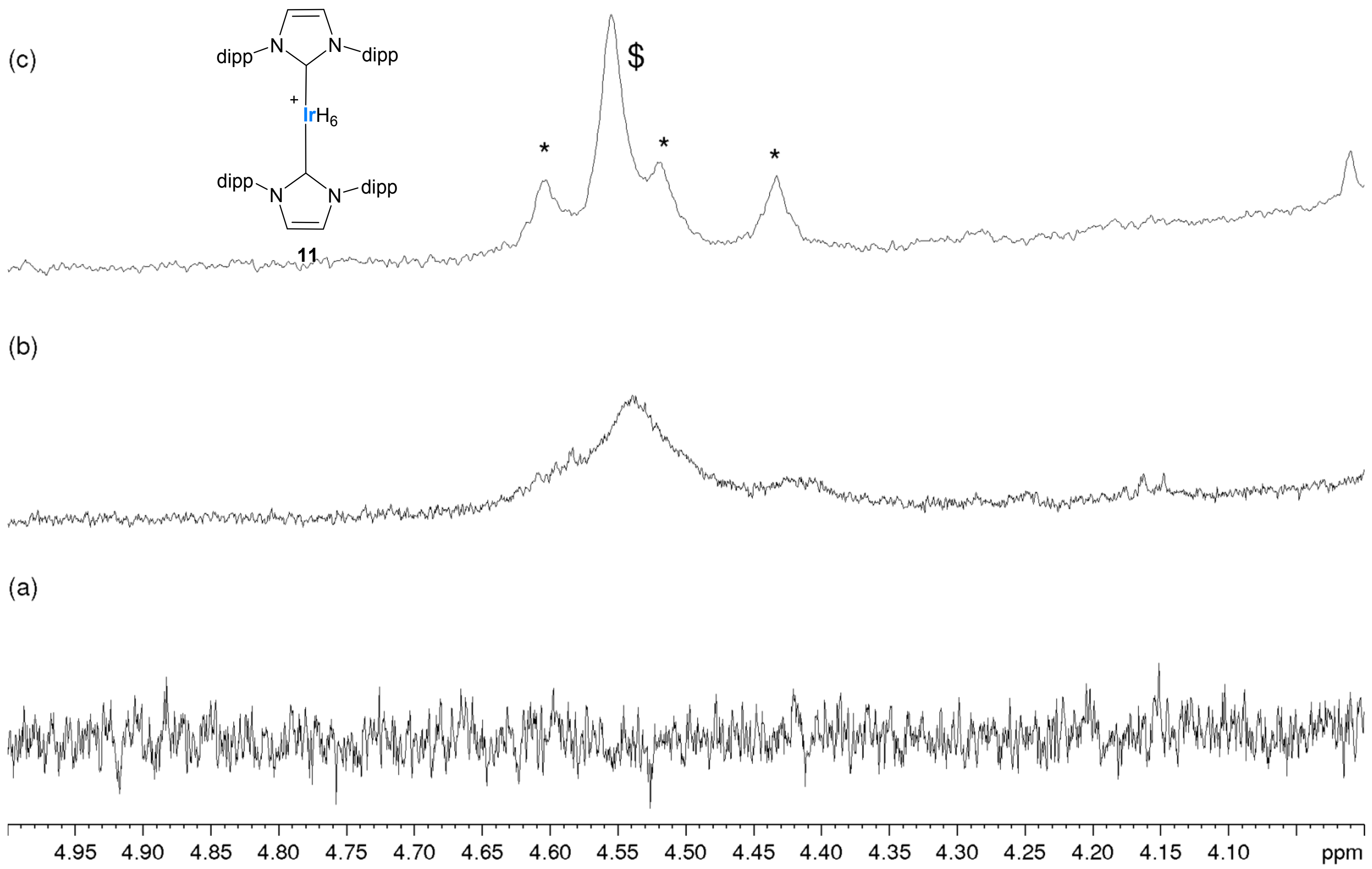

Figure S42. ^{1}H NMR spectrum (THF-d_8, 500 MHz) of the reaction of $[Ir(IPr)_2H_6][BAr^F_4]$ (**11**) with D_2. (a) 298 K spectrum 1 h after addition, (b) 248 K spectrum 2 h after addition and (c) 218 K spectrum 4h after addition. * denotes free HD, $ denotes free H_2.

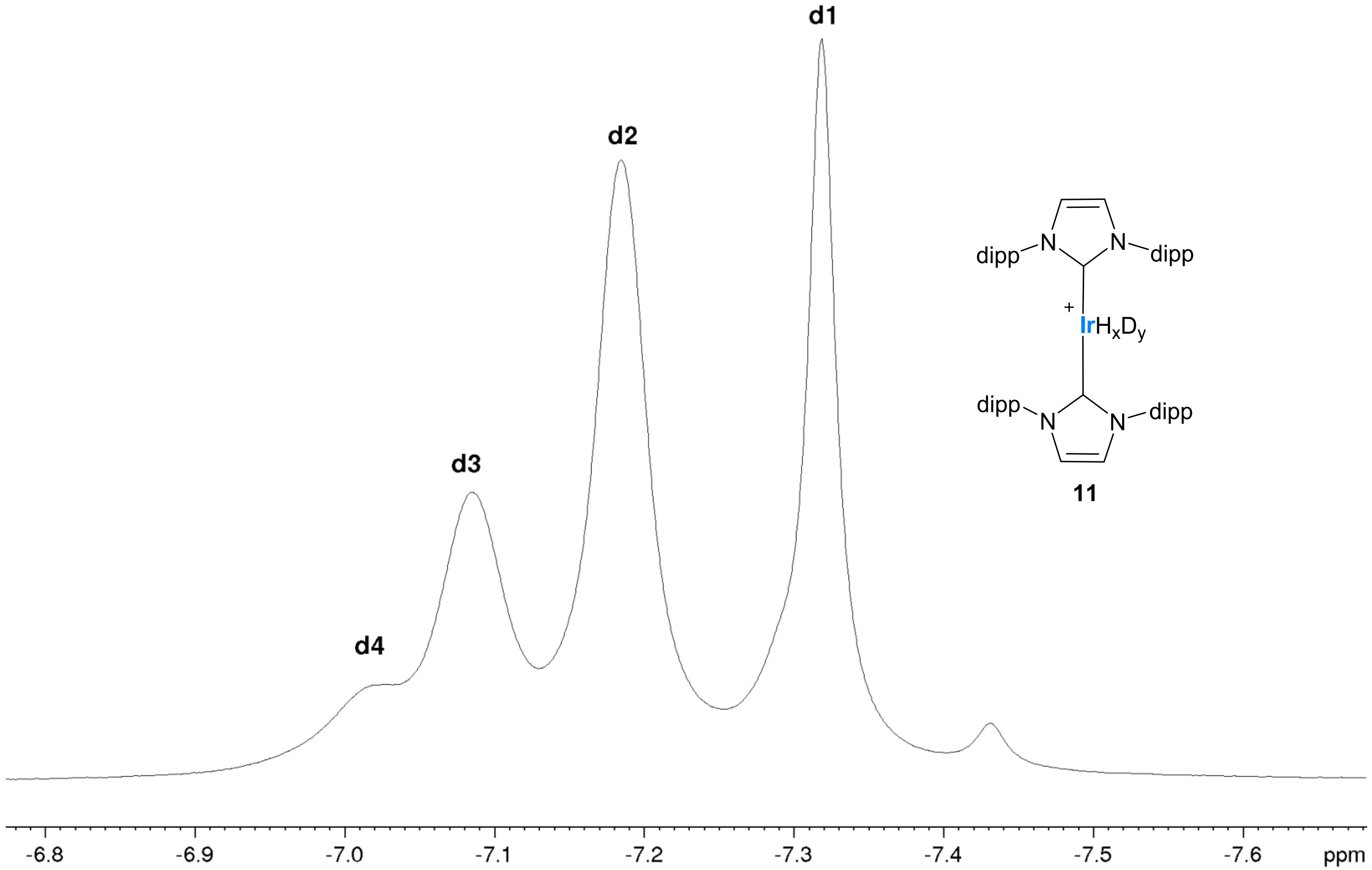

Figure S43. Hydride region of the low temperature ^{1}H NMR spectrum (THF-d_8, 500 MHz, 248 K) of a sample of $[Ir(IPr)_2H_6][BAr^F{}_4]$ (**11**) exposed to D_2, degassing and then H_2 to afford a mixture of $[Ir(IPr)_2H_2D_4][BAr^F{}_4]$ (**11-*d*$_4$**), $[Ir(IPr)_2H_3D_3][BAr^F{}_4]$ (**11-*d*$_3$**), $[Ir(IPr)_2H_4D_2][BAr^F{}_4]$ (**11-*d*$_2$**) and $[Ir(IPr)_2H_5D_1][BAr^F{}_4]$ (**11-*d*$_1$**). The spectrum is shown to highlight the increase in linewidth with higher deuterium content.

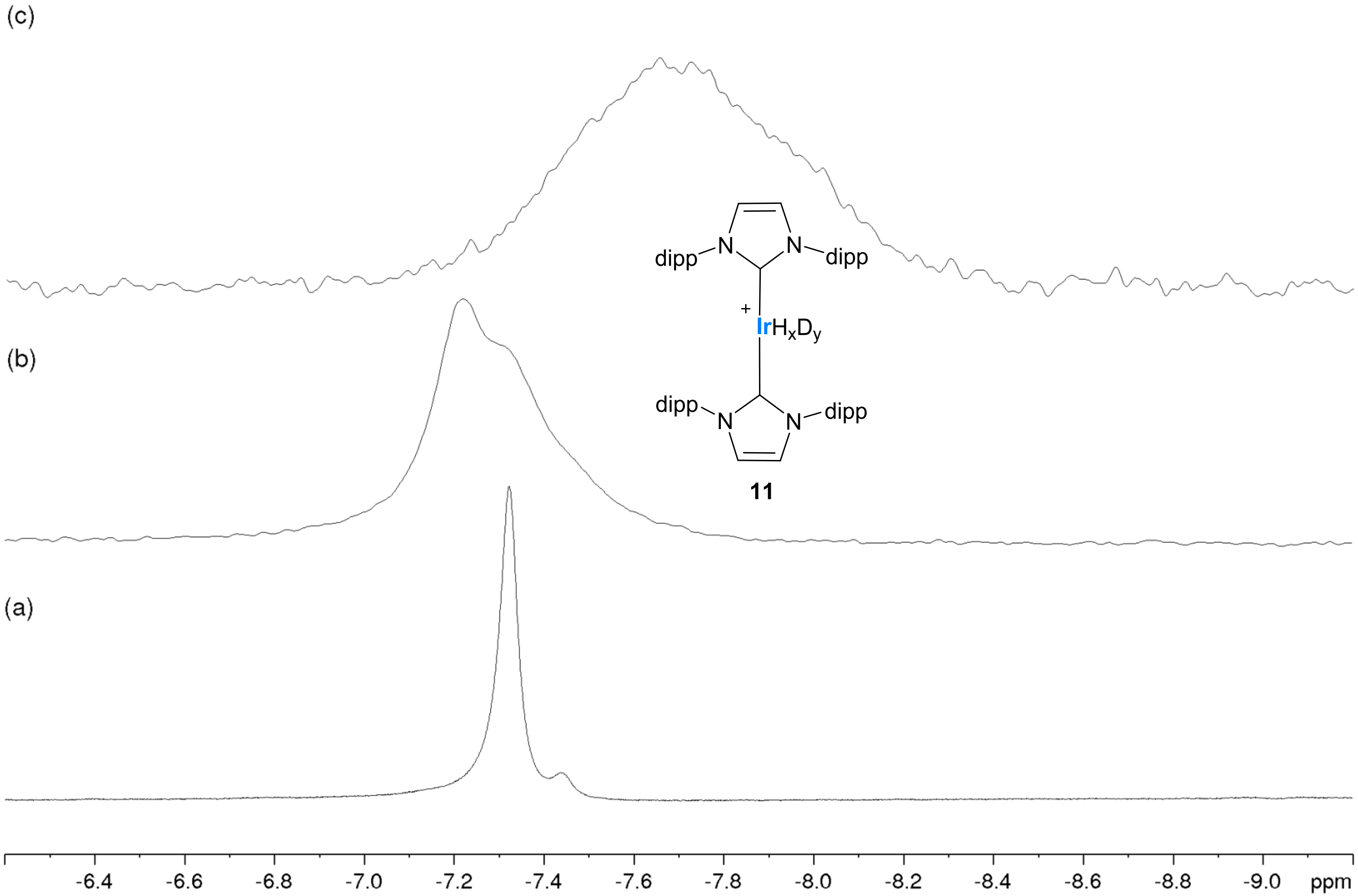

Figure S44. Low frequency region of (a) the ^{1}H NMR spectrum (THF-h_8, 400 MHz, 228 K) of $[Ir(IPr)_2H_6][BAr^F{}_4]$ (**11**) together with the ^{2}H NMR spectra (THF-h_8, 61 MHz, 248 K) of (b) **11** after 24 h under 1 atm D_2 and (c) after degassing and addition of 1 atm H_2.

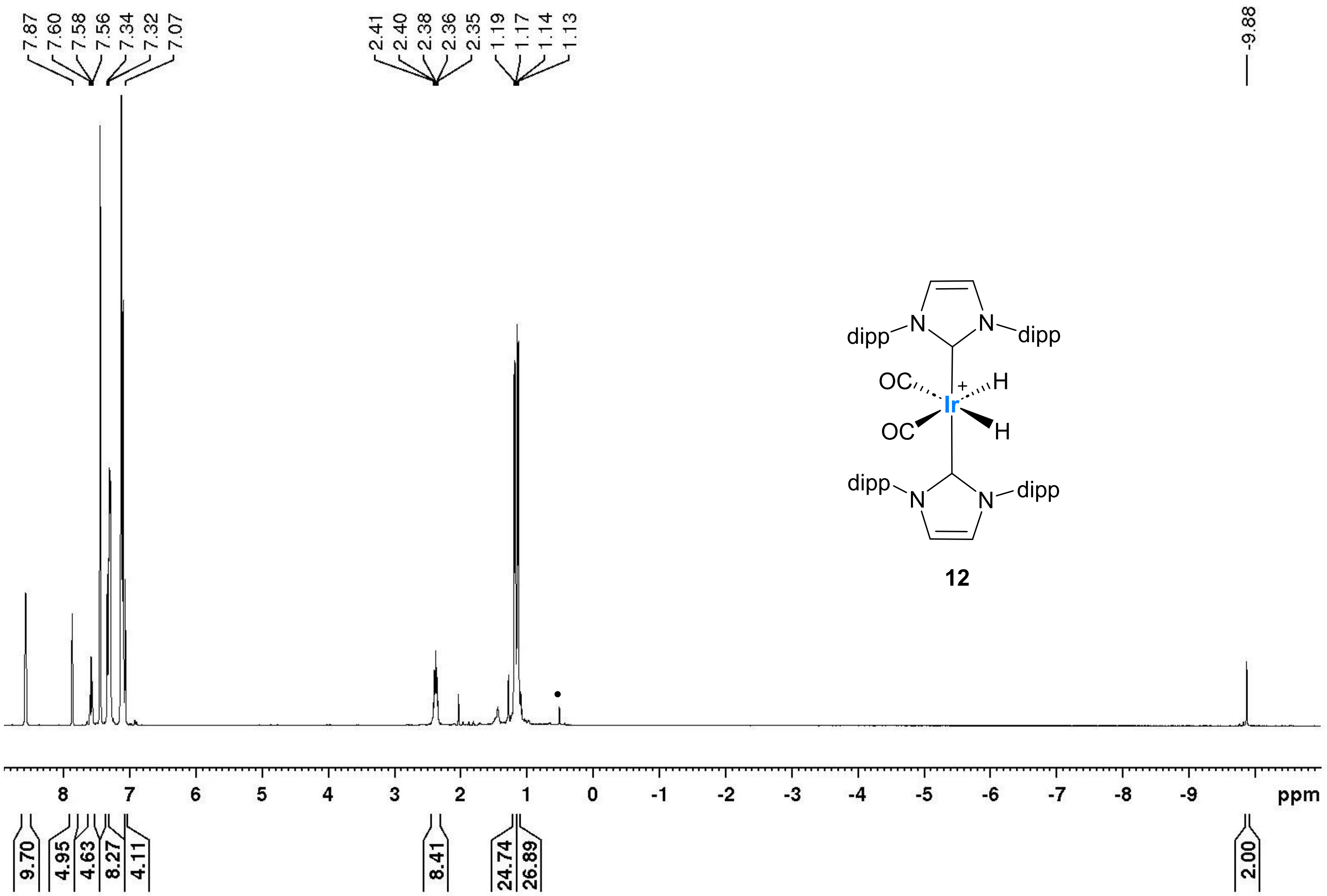

Figure S45. Room temperature ^{1}H NMR spectrum (C_6D_5F, 400 MHz, 298 K) of $[Ir(IPr)_2(CO)_2H_2][BAr^F_4]$ (**12**) (• = silicone grease).

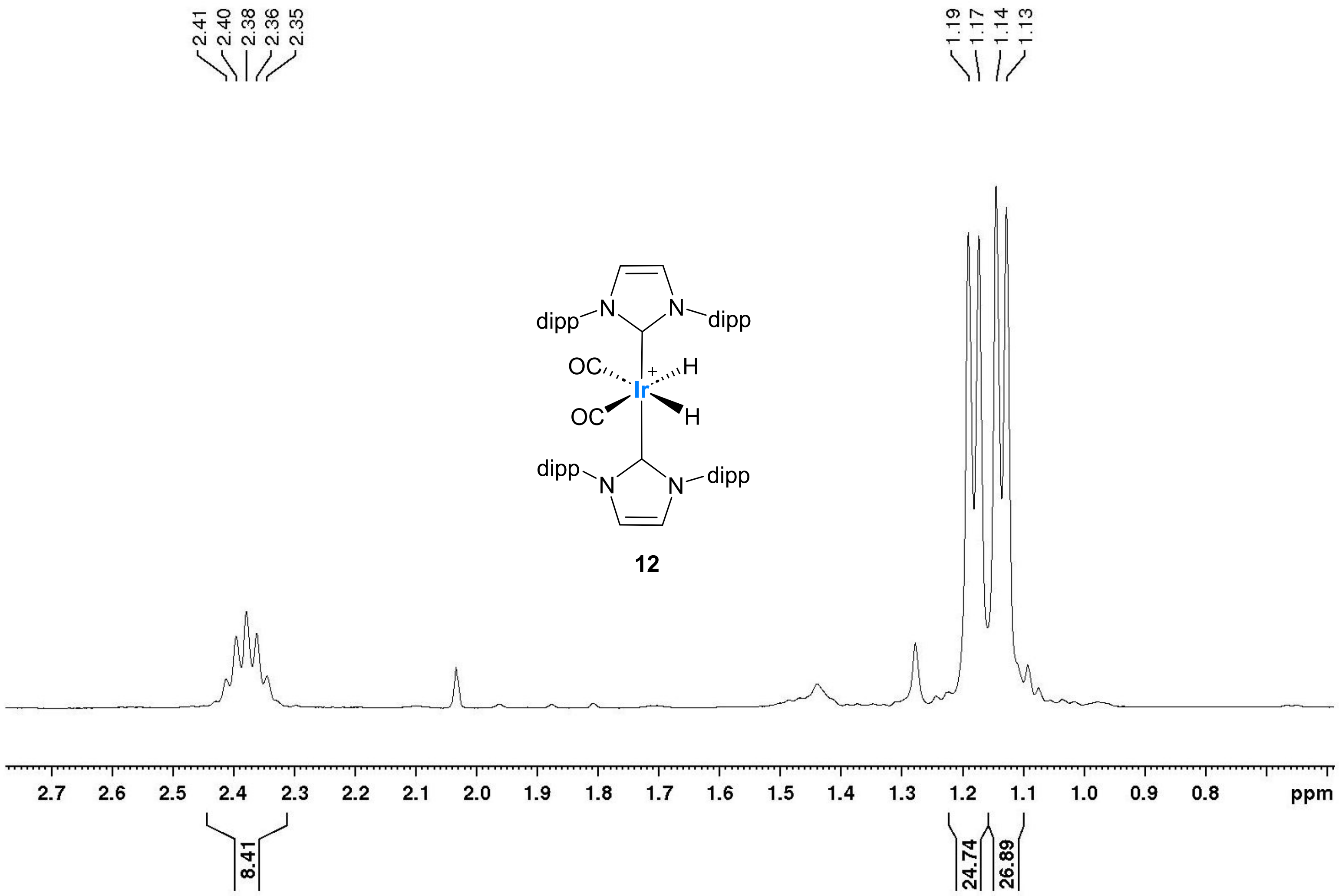

Figure S46. Alkyl region of the room temperature ^{1}H NMR spectrum (C_6D_5F, 400 MHz, 298 K) of $[Ir(IPr)_2(CO)_2H_2][BAr^F{}_4]$ (**12**).

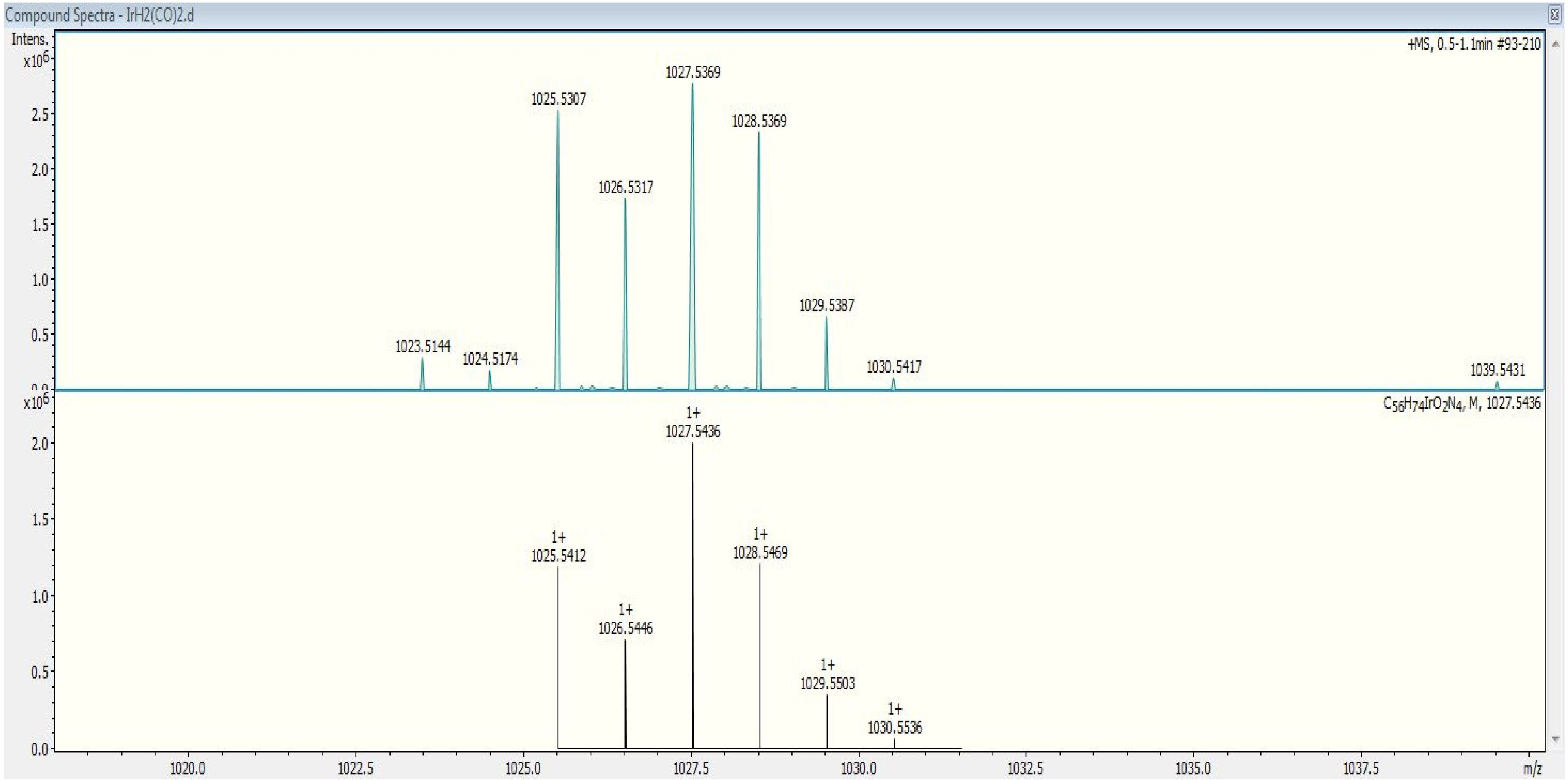

Figure S47. ESI mass spectrum of $[Ir(IPr)_2(CO)_2H_2]^+$ (**12**$^+$).

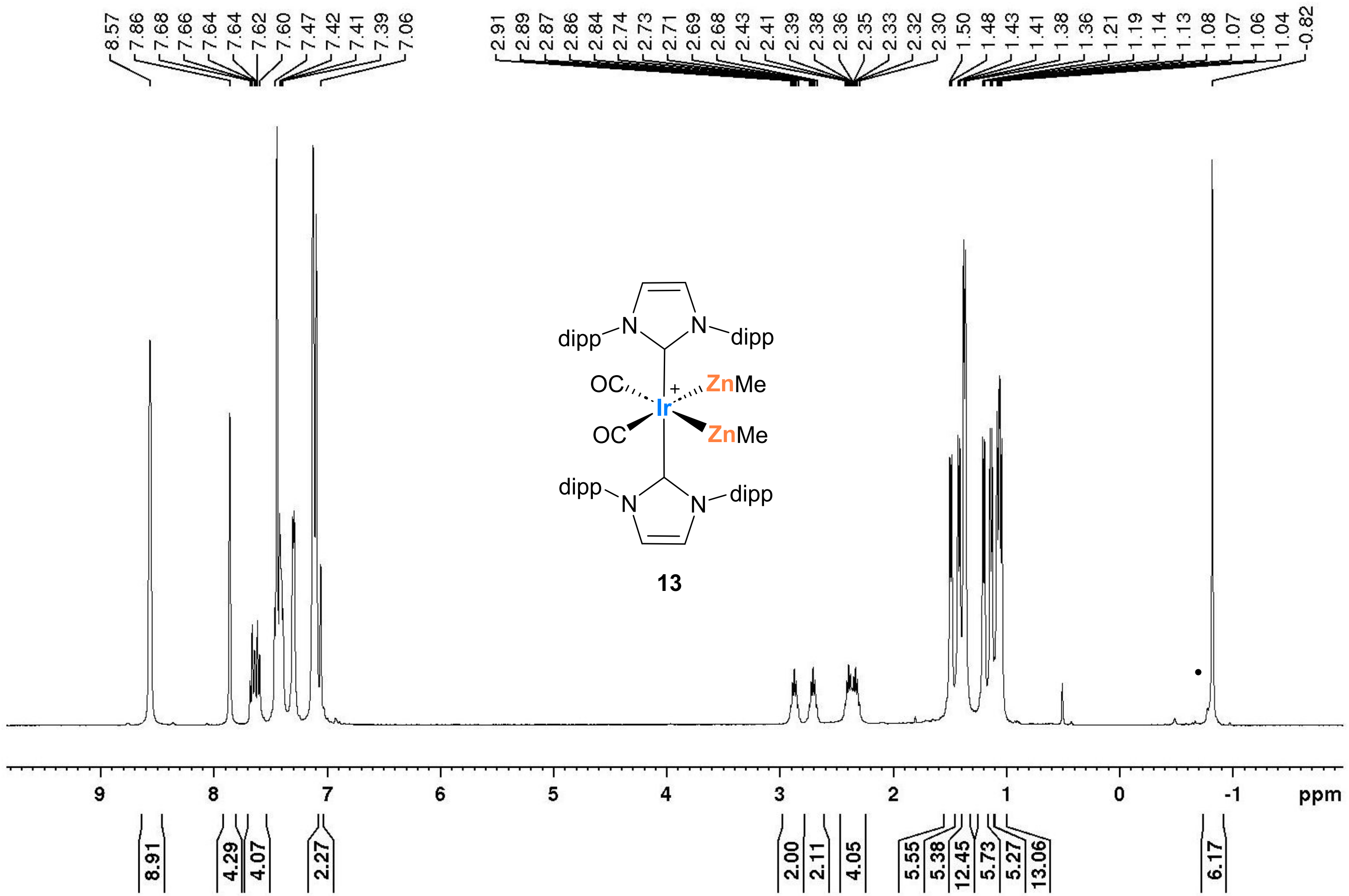

Figure S48. Room temperature ^{1}H NMR spectrum (C_6D_5F, 400 MHz, 298 K) of $[Ir(IPr)_2(CO)_2(ZnMe)_2][BAr^F_4]$ (**13**) (• = silicone grease).

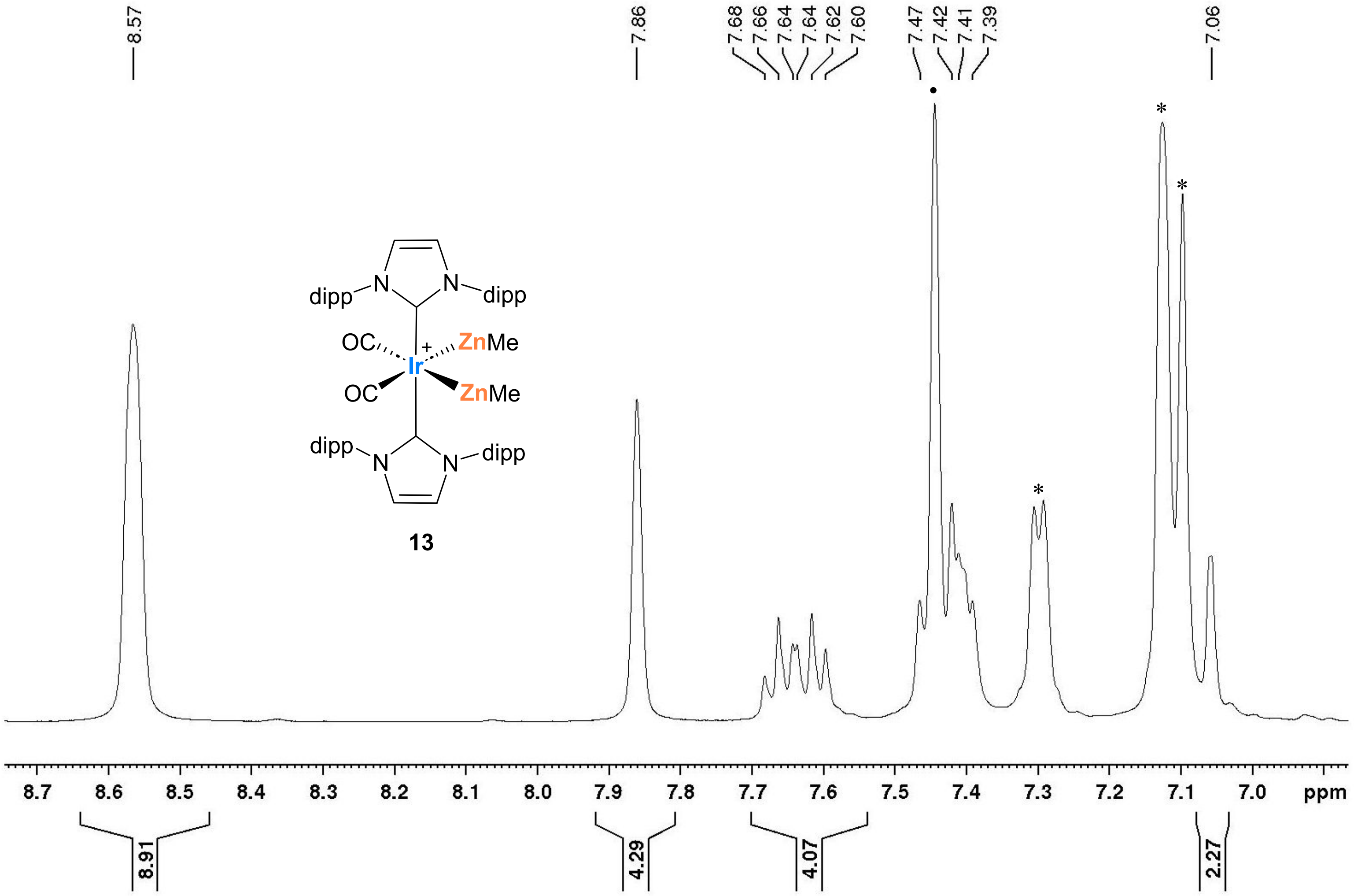

Figure S49. High frequency region of the room temperature ^{1}H NMR spectrum (C_6D_5F, 400 MHz, 298 K) of $[Ir(IPr)_2(CO)_2(ZnMe)_2][BAr^F{}_4]$ (**13**) (• = C_6H_6, * = C_6H_5F).

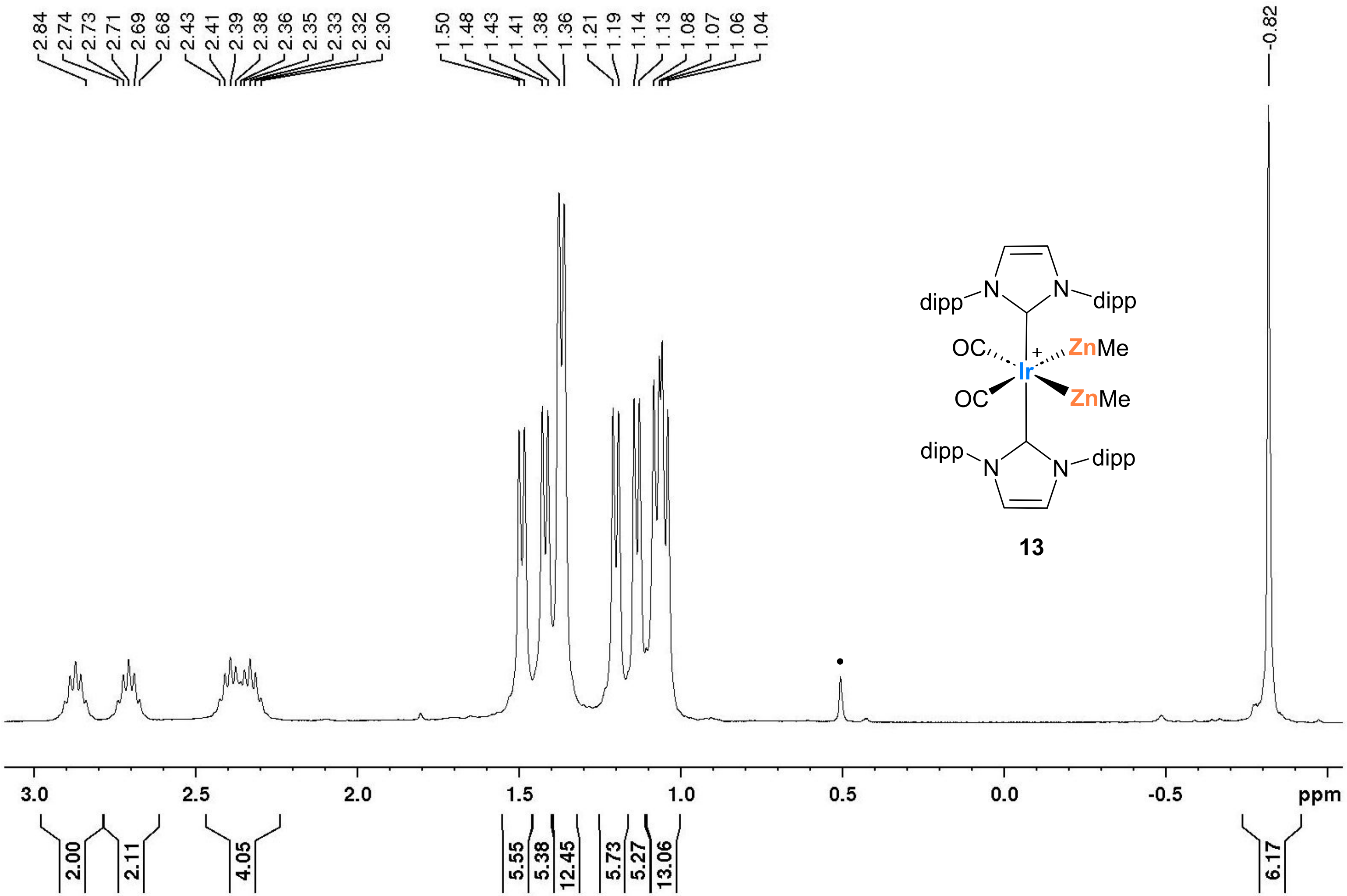

Figure S50. Alkyl region of the room temperature ^{1}H NMR spectrum (C_6D_5F, 400 MHz, 298 K) of $[Ir(IPr)_2(CO)_2(ZnMe)_2][BAr^F{}_4]$ (**13**). (• = silicone grease).

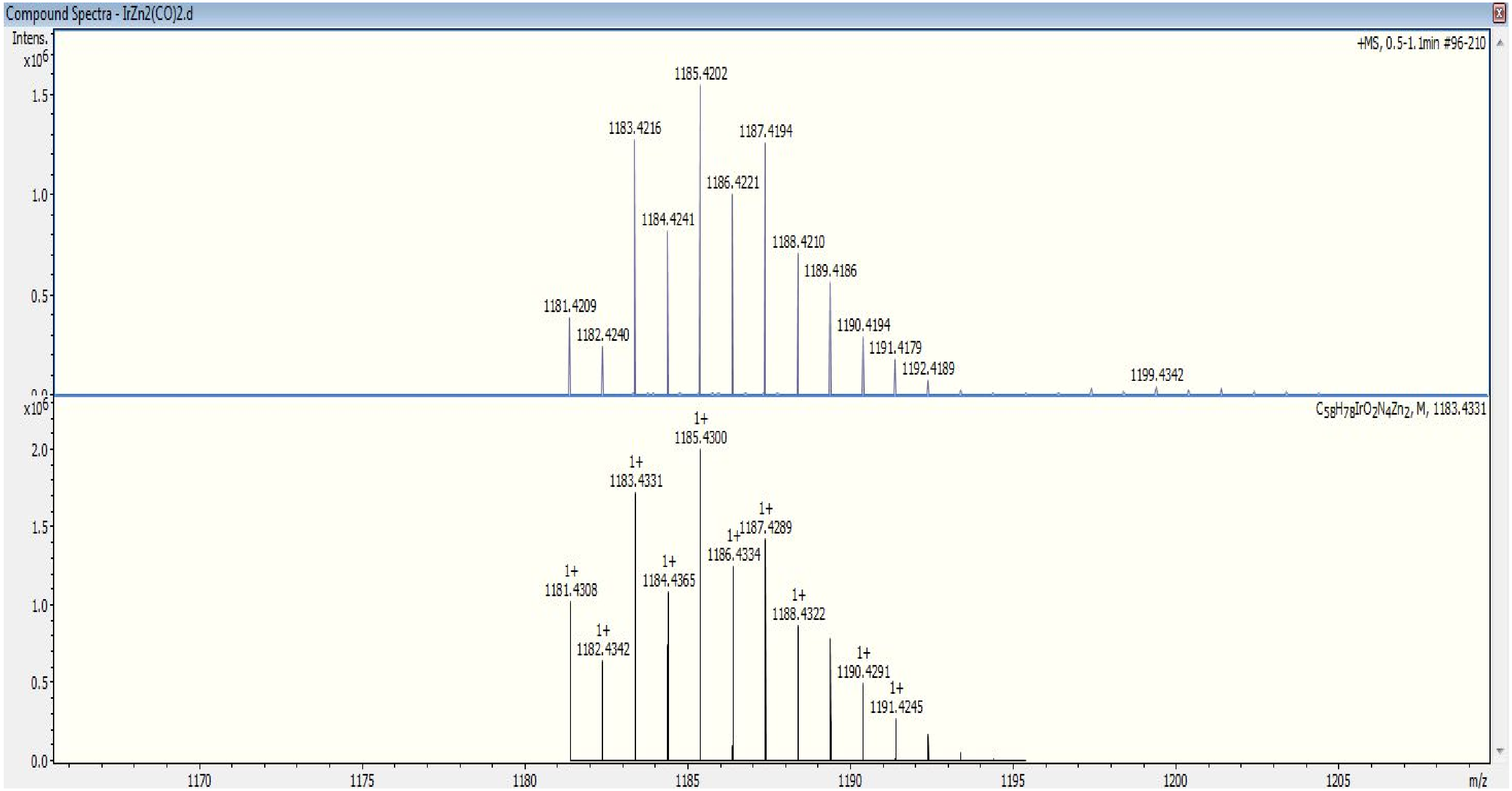

Figure S51. ESI mass spectrum of $[Ir(IPr)_2(CO)_2(ZnMe)_2]^+$ (**13**$^+$).

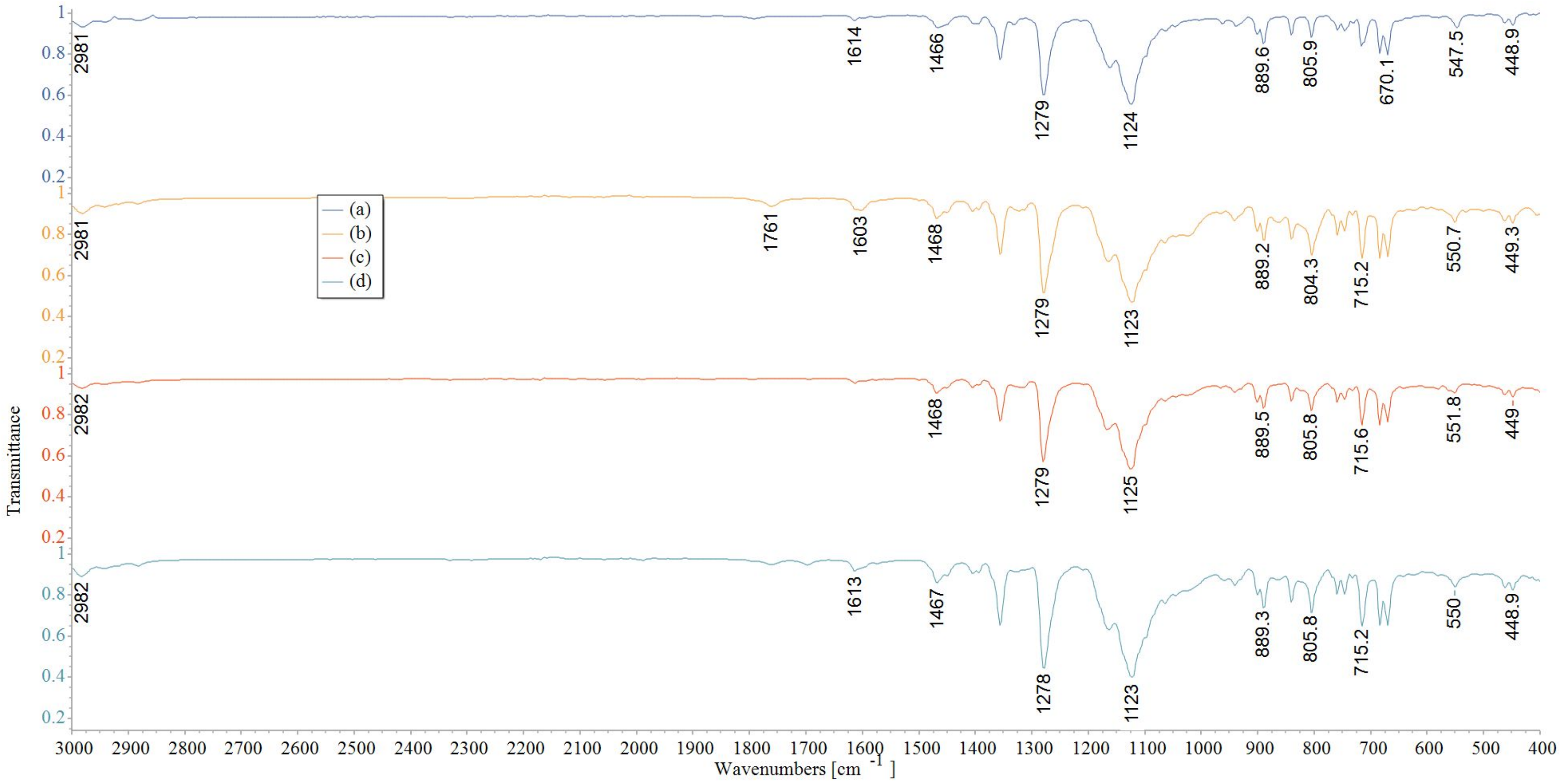

Figure S52. ATR-IR spectra of (a) [Ir(IPr)(IPr')(ZnMe)$_2$H][BAr$^F{}_4$] (**3**), (b) [Ir(IPr)$_2$(ZnMe)$_2$H$_4$][BAr$^F{}_4$] (**4**), (c) **3** + D$_2$ and (d) [Ir(IPr)$_2$(ZnMe)$_2$H$_2$][BAr$^F{}_4$] (**5**).

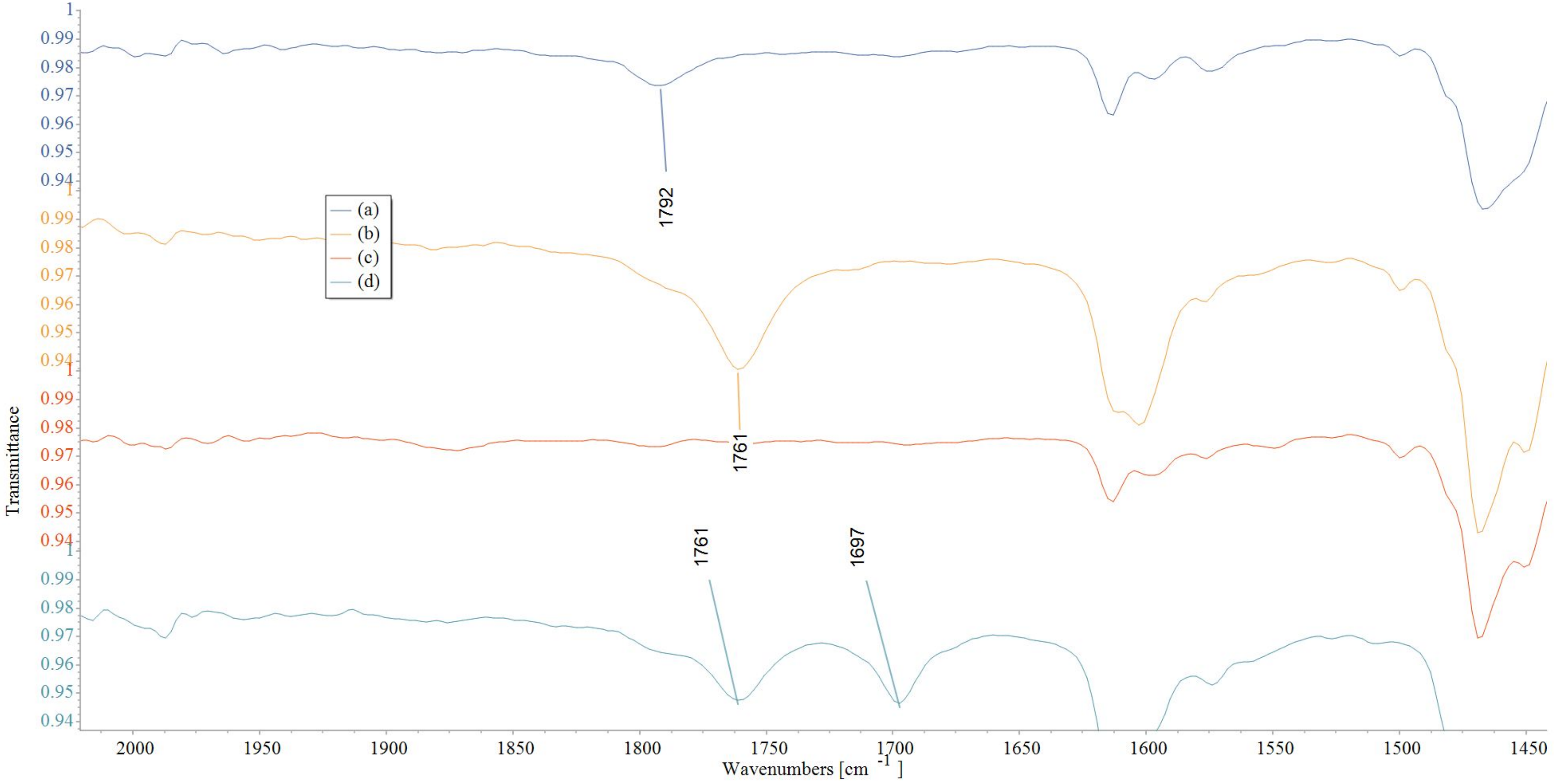

Figure S53. Ir-H stretching region of the ATR-IR spectra of (a) [Ir(IPr)(IPr')$(ZnMe)_2$H][BAr^F_4] (**3**), (b) [$Ir(IPr)_2(ZnMe)_2H_4$][BAr^F_4] (**4**), (c) **3** + D_2 and (d) [$Ir(IPr)_2(ZnMe)_2H_2$][BAr^F_4] (**5**).

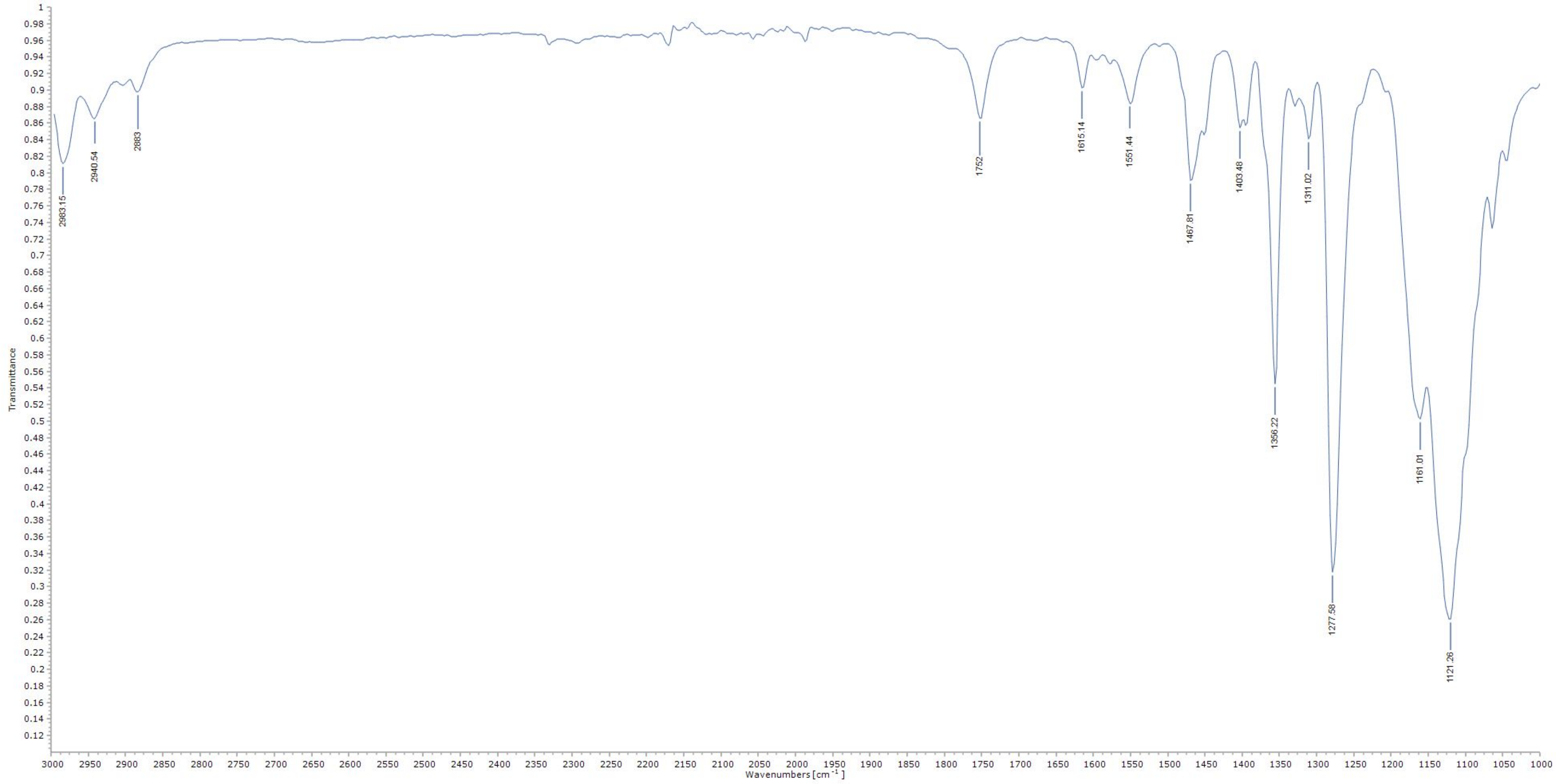

Figure S54. ATR-IR spectrum of $[Ir(IPr)_2(CdMe)_2H_4][BAr^F{}_4]$ (**8**).

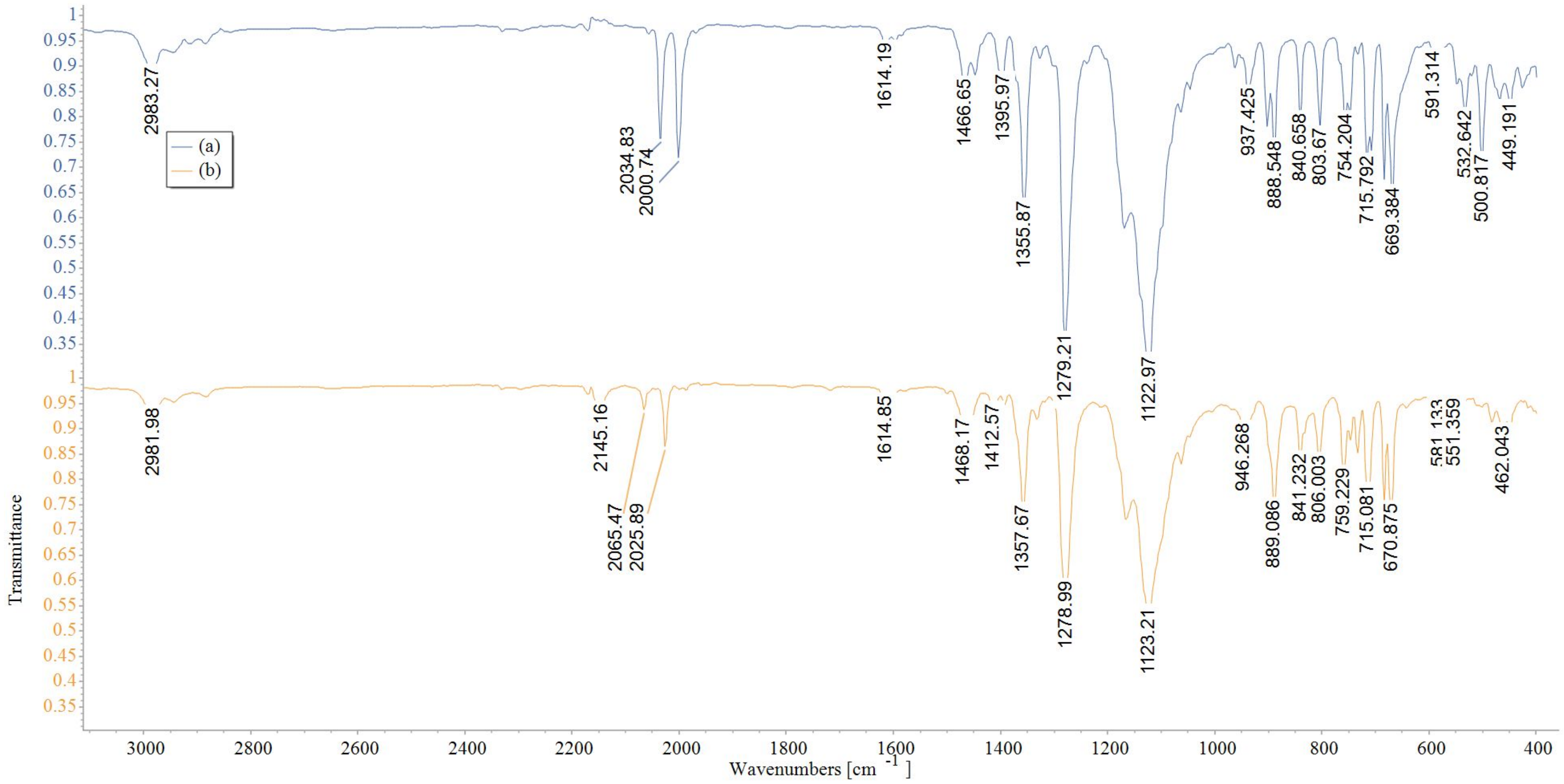

Figure S55. ATR-IR spectra of (a) $[Ir(IPr)_2(CO)_2(ZnMe)_2][BAr^F{}_4]$ (**13**) and (b) $[Ir(IPr)_2(CO)_2H_2][BAr^F{}_4]$ (**12**).

2D-IR spectroscopy. The two-dimensional infrared (2D-IR) spectrometer consisted of two Yb-based amplified lasers (Pharos 20W and Pharos 10W, Light Conversion) synchronized by a single, common oscillator.[20] The amplifiers were each used to pump an optical parametric amplifier (OPA, Orpheus Mid-IR, Light Conversion) equipped with difference frequency generation (Lyra, Light Conversion) to produce independently tuneable sources for one or two-color 2D-IR spectroscopy. In the mid-IR, the two OPAs produce usable bandwidths of >200 cm^{-1} with energies of 2.5 and 1.5 μJ/pulse respectively at a pulse repetition rate of 50 kHz. 2D-IR data collection was achieved using a 2DQuick spectrometer (Phasetech). The spectrometer employs the pump-probe beam geometry for 2D-IR data collection and uses a mid-IR pulse shaper to generate and control the time delay (τ) between the pair of 'pump' pulses.[21-23] The waiting time (T_w) between the second pump and probe pulses was determined by an optical delay line. Signal measurement was achieved via twin 64-element HgCdTe array detectors configured to allow simultaneous collection of either signal and reference or ZZZZ (parallel) and ZZYY (perpendicular) polarization-resolved data. In the experiments that follow, the output of the OPA pumped by the Pharos 20W amplifier was used to create all three infrared pulses (two pump, one probe). Excitation pulses were centred either at 2000 cm^{-1}, or at 2140 cm^{-1} to focus on the ν_{CO} or $\nu_{Ir\text{-}H}$ modes respectively. For a given value of T_w, τ was scanned in steps of 20 fs to a maximum delay time of 4 ps applying a rotating frame frequency of 1585 cm^{-1}. Each 2D-IR plot represents the average of 2000 spectra, repeated 3 times.

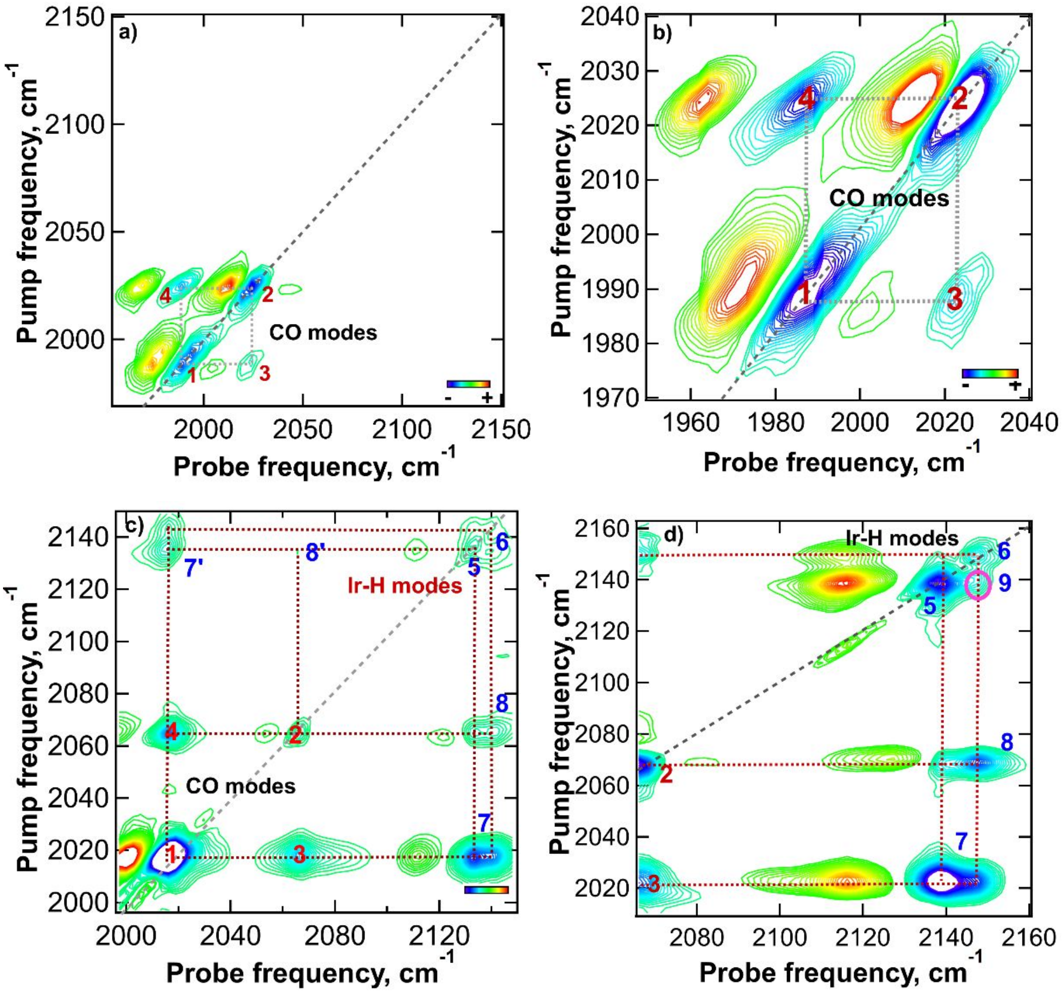

Figure S56. 2D-IR spectra of (a,b) $[Ir(IPr)_2(CO)_2(ZnMe)_2][BAr^F{}_4]$ (**13**) and (c,d) $[Ir(IPr)_2(CO)_2H_2][BAr^F{}_4]$ (**12**) in fluorobenzene solution measured at T_w = 250 fs using ZZYY (perpendicular) polarization. Spectra a, b and c were acquired with the excitation frequency set to 2000 cm^{-1} in resonance with the ν_{CO} modes, whereas the spectrum in d was recorded with the excitation frequency set on resonance with the Ir-H modes at 2140 cm^{-1}.

2D-IR spectra interpretation. The 2D-IR spectra in Figure S56 strengthen the characterization of the compounds $[Ir(IPr)_2(CO)_2H_2][BAr^F{}_4]$ (**12**) and $[Ir(IPr)_2(CO)_2(ZnMe)_2][BAr^F{}_4]$ (**13**). The spectra recorded for **13** (Figure S56a and b) display two peaks (indicated by red labels 1, 2) along the diagonal (pump frequency = probe frequency) corresponding to the fundamental v=0-1 transitions of the ν_{CO} symmetric and antisymmetric stretching modes, also observed in the IR absorption spectrum (Figure S56,

peaks indicated by the orange rectangles). It is evident from this spectrum that no other peaks are detectable along the diagonal at higher frequencies consistent with the absence of hydride ligands in **13**. Figure 56b shows an expansion of the same spectrum focussing on the ν_{CO} region; off-diagonal peaks (labelled 3 & 4 and connected by dotted grey lines) are visible in addition to the on-diagonal peaks 1 and 2. Peaks 3 and 4 are cross peaks indicating vibrational coupling and delocalization between the two ν_{CO} modes.

The 2D-IR spectrum of **12** (Figure 56c) in the ν_{CO} region is very similar to that of **13** displaying a square pattern of four peaks (1-4). In addition to the ν_{CO} modes, the spectrum for **12** displays extra peaks along the diagonal at a frequency of ca. 2140 cm^{-1} (peaks 5 & 6 in Figure S56c). These extra features are vibrationally coupled to the two ν_{CO} modes as indicated by the presence of the cross peaks identified by the dotted red lines on the spectrum. Shifting the excitation frequency from 2000 cm^{-1} to 2140 cm^{-1} gave access to more detail relating to these peaks, which are weaker than those due to the ν_{CO} modes.

The expanded region (Figure S56d) shows two peaks 5 and 6, which are coupled to each other (via peak 9, indicated by the pink circle) and also show coupling to the ν_{CO} modes indicated by cross peaks 7, (7'), & 8 (8') as mentioned above. From this and comparison to literature on metal-hydride IR data,[24] we conclude that peaks 5 and 6 are assignable to the symmetric and antisymmetric $\nu_{Ir\text{-}H}$ modes. The coupling patterns indicate that there is a degree of delocalization both between the $\nu_{Ir\text{-}H}$ modes and also involving the ν_{CO} modes, as noted in reference 64 in the manuscript.

S2. Computational Studies.

S2.1. Computational Details. DFT calculations were run with Gaussian 16 (Revision C.01).[25] Ir and Zn centers were described with Stuttgart RECPs and associated basis sets[26] with 6-31G** basis sets used for all other atoms.[27,28] Optimizations employed the BP86[29,30] functional and included a correction for dispersion (D3[31] with Becke-Johnson damping[32]). All stationary points were fully characterised via analytical frequency calculations as either minima (all positive eigenvalues) or transition states (one negative eigenvalue). In the cases of **8**$^+$ and **9**$^+$ a very small imaginary mode was computed (< 10 cm^{-1}) that despite repeated attempts could not be removed. Transition states were also characterised via IRC calculations and subsequent geometry optimizations confirmed they linked to the minima as reported in the text or in the Supporting Information. The electronic energies were then recomputed with the PBE0 functional using the def2TZVP[33,34] basis set and corrected for the effects of fluorobenzene solvent using the PCM approach and dispersion (D3BJ).[35] The corrected electronic energies were then combined with the thermochemical corrections from the BP86-optimized geometries to give the free energies quoted in the text.

Quantum theory of atoms in molecules (QTAIM)[36] analyses used the AIMALL program[37] and were performed on optimised structures with starting geometries taken from the crystallographic studies. All BCP metrics are provided in atomic units and Figure S57-S65 include values for the Laplacian ($\nabla^2\rho(r)$) and total energy density (H(r)). Computed geometries are displayed with ChemCraft[38] with all geometries supplied as a separate XYZ file.

S2.2 Functional testing

Geometries of **1**$^+$-**5**$^+$ were optimized with the BP86 functional both with and without a dispersion correction. In general, improved geometries were obtained when optimized with dispersion (Table S2), in particular, the Zn-Ir-Zn angle in **2**$^+$ and the description of the agostic interaction in **1**$^+$ and their absence in **2**$^+$.

Table S2. Key parameters optimised with and without a D3BJ correction for **1**$^+$ and **2**$^+$.

**Complex**		**X-Ir-X/°**	**Shortest Ir…C contact/Å**[a]
**1**$^+$ (X = H)	Experiment	-	3.049(5)/2.943(5)
	BP86	83.0	3.23
	BP86-D3BJ	81.5	3.01
**2**$^+$ (X = ZnMe)	Experiment	84.5(3)	3.35
	BP86	96.7	4.54
	BP86-D3BJ	81.2	3.41

[a]Shortest contact to an IPr Me group to reflect the extent of any agostic interaction.

The relative energies of isomers **2**$^+$ and **3**$^+$ and **11**$^+$ and **11a**$^+$ were subjected to a functional screening using the BP86-D3 optimized structures and a range of functionals, including GGA functionals (PBE,[39] BP86,[29,30] BLYP,[31] B97D,[41] TPSS[42]), hybrid GGA functionals (PBE0,[43] B3LYP,[44] ωB97x-D,[45] B3PW91[44,46]) and Minnesota functionals (M06, M06L and M06-2X).[47] Relative free energies are reported in Table S3 (kcal/mol) and include corrections for C_6H_5F solvent and dispersion (except for B97D, ωB97x-D and the Minnesota functionals). For the **2**$^+$/**3**$^+$ pair no systematic preference was found; however, all functionals found the bis-dihydrogen dihydride tautomer of **11**$^+$ to be favored. PBE0 was selected in the reactivity studies as one of the functionals that predict **3**$^+$ to dominate over **2**$^+$ in solution, as seen experimentally.

Table S3. Functional screening for the **2**$^+$/**3**$^+$ and **11**$^+$/**11a**$^+$ pairs. For **2**$^+$ vs. **3**$^+$, a negative value (kcal/mol) indicates non-cyclometalated **2**$^+$ is favored. For **11**$^+$ vs. **11a**$^+$, negative values indicate the bis-dihydrogen dihydride tautomer of **11**$^+$ is favored.

**XC**	**BP86**	**BLYP**	**B3LYP**	**PBE**	**PBE0**	**B97D3**	**B97D**	**M06**	**ωB97x-D**	**TPSS**	**B3PW91**	**M06L**	**M06-2X**
**2**$^+$ vs. **3**$^+$	-0.3	-6.8	-4.2	-1.0	+2.0	-6.7	-7.8	-8.1	-1.3	+2.6	+2.1	-4.6	-3.9
**11**$^+$ vs. **11a**$^+$	-6.7	-8.9	-10.6	-6.4	-8.5	-8.7	-10.2	-13.1	-0.5	-8.2	-8.7	-13.6	-12.4

S2.3 QTAIM Studies

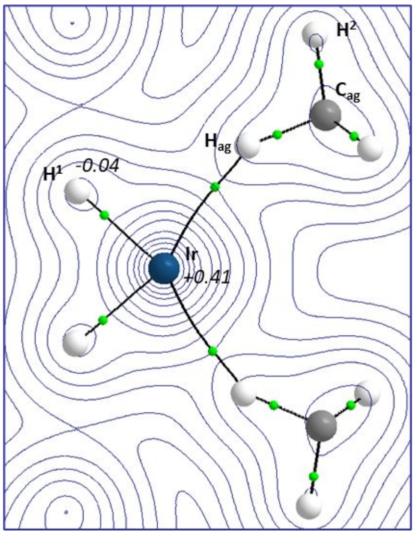

Bond	ρ(r)	$\nabla^2\rho$(r)	ε	H (r)	δ (A,B)
**Ir–H¹**	0.171	0.021	0.008	-0.105	0.988
**Ir-H_{ag}**	0.046	0.111	0.195	-0.011	0.220
**C_{ag}-H_{ag}**	0.246	-0.710	0.017	-0.226	0.814
**C_{ag}-H^2**	0.270	-0.894	0.012	-0.268	0.923

Figure S57. QTAIM molecular graphs with selected BCP metrics (au) tabulated for **1**$^+$. Density contours are shown in the equatorial plane with axial ligands omitted for clarity. BCPs and RCPs shown in green and red respectively, with selected atomic charges in italics.

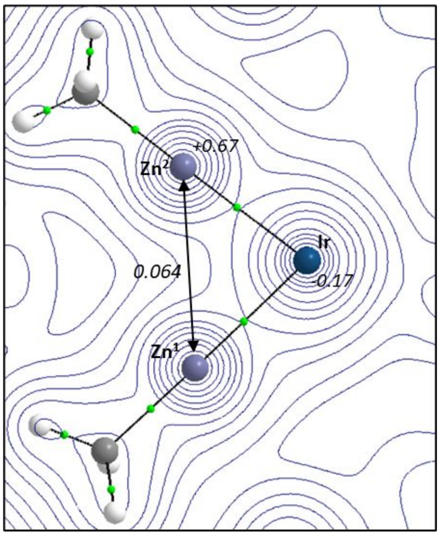

Bond	ρ(r)	$\nabla^2\rho$(r)	ε	H (r)	δ (A,B)
**Ir–Zn**	0.078	0.147	0.032	-0.025	0.888

Figure S58. QTAIM molecular graphs with selected BCP metrics (au) tabulated for **2**$^+$. Density contours are shown in the equatorial plane with axial ligands omitted for clarity. BCPs and RCPs shown in green and red respectively, with selected atomic charges shown in italics. Delocalization indices between selected atoms are also indicated.

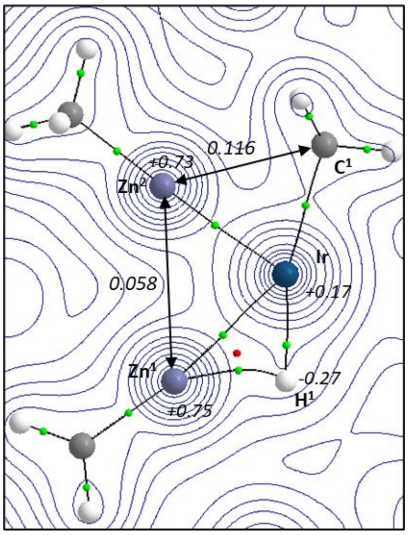

Bond	ρ(r)	$\nabla^2\rho$(r)	ε	H (r)	δ (A,B)
**Ir–H¹**	0.118	0.152	0.048	-0.053	0.728
**Ir-Zn¹**	0.067	0.115	0.395	-0.021	0.598
**Ir-Zn²**	0.074	0.139	0.032	-0.023	0.708
**Ir-C**	0.110	0.124	0.086	-0.045	0.843
**Zn¹-H¹**	0.066	0.110	1.129	-0.017	0.289

Figure S59. QTAIM molecular graphs with selected BCP metrics (au) tabulated for **3**$^+$. Density contours are shown in the equatorial plane with axial ligands omitted for clarity. BCPs and RCPs shown in green and red respectively, with selected atomic charges shown in italics. Delocalization indices between selected atoms are also indicated.

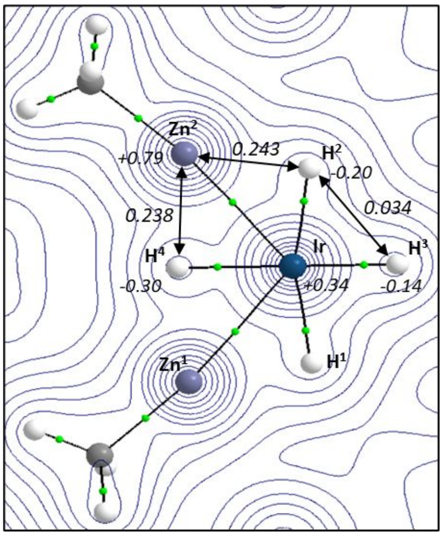

Bond	ρ(r)	$\nabla^2\rho$(r)	ε	H (r)	δ (A,B)
**Ir–H¹**	0./123	0.141	0.020	-0.057	0.748
**Ir-H³**	0.151	0.063	0.036	-0.083	0.872
**Ir-H⁴**	0.098	0.188	0.176	-0.038	0.623
**Ir-Zn¹**	0.061	0.099	0.637	-0.018	0.419

Figure S60. QTAIM molecular graphs with selected BCP metrics (au) tabulated for **4**$^+$. Density contours are shown in the equatorial plane with axial ligands omitted for clarity. BCPs and RCPs shown in green and red respectively, with selected atomic charges shown in italics. Delocalization indices between selected atoms are also indicated.

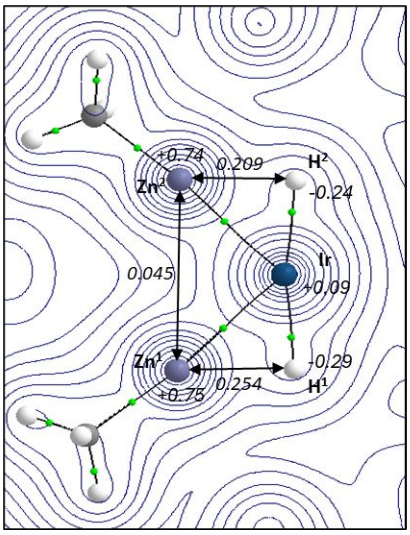

Bond	ρ(r)	$\nabla^2\rho$(r)	ε	H (r)	δ (A,B)
**Ir–H¹**	0.126	0.139	0.045	-0.059	0.787
**Ir-H²**	0.120	0.152	0.046	-0.054	0.750
**Ir-Zn¹**	0.069	0.128	0.167	-0.021	0.642
**Ir-Zn²**	0.071	0.135	0.063	-0.022	0.674

Figure S61. QTAIM molecular graphs with selected BCP metrics (au) tabulated for **5**$^{+}$. Density contours are shown in the equatorial plane with axial ligands omitted for clarity. BCPs and RCPs shown in green and red respectively, with selected atomic charges shown in italics. Delocalization indices between selected atoms are also indicated.

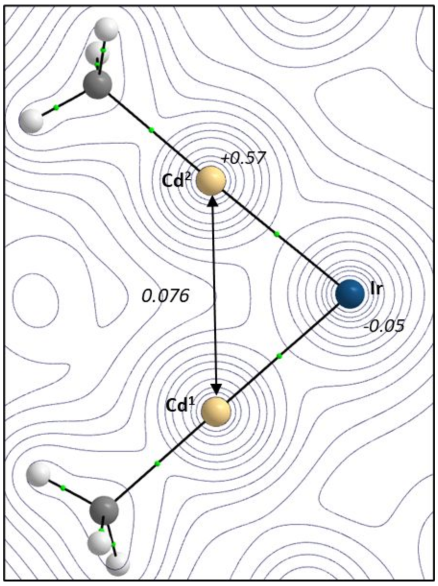

Bond	ρ(r)	$\nabla^2\rho$(r)	ε	H (r)	δ (A,B)
**Ir–Cd**	0.069	0.118	0.033	-0.021	0.871

Figure S62. QTAIM molecular graphs with selected BCP metrics (au) tabulated for **6**$^+$. Density contours are shown in the equatorial plane with axial ligands omitted for clarity. BCPs and RCPs shown in green and red respectively, with selected atomic charges shown in italics. Delocalization indices between selected atoms are also indicated.

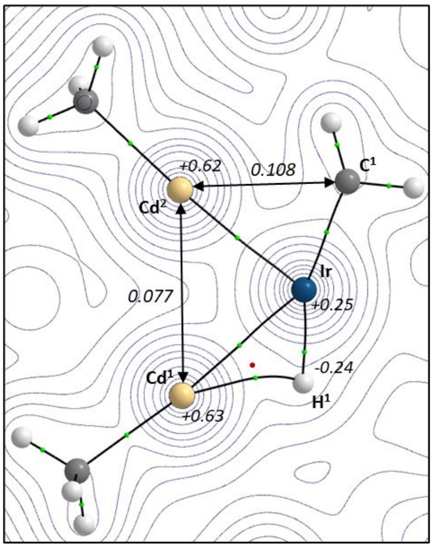

Bond	ρ(r)	$\nabla^2\rho$(r)	ε	H (r)	δ (A,B)
**Ir–H^1**	0.120	0.133	0.064	-0.055	0.732
**Ir-Cd^1**	0.059	0.103	0.316	-0.015	0.592
**Ir-Cd^2**	0.065	0.116	0.036	-0.018	0.697
**Ir-C^1**	0.112	0.124	0.096	-0.045	0.843
**Cd^1-H^1**	0.057	0.103	1.145	-0.012	0.278

Figure S63. QTAIM molecular graphs with selected BCP metrics (au) tabulated for **7**$^+$. Density contours are shown in the equatorial plane with axial ligands omitted for clarity. BCPs and RCPs shown in green and red respectively, with selected atomic charges shown in italics. Delocalization indices between selected atoms are also indicated.

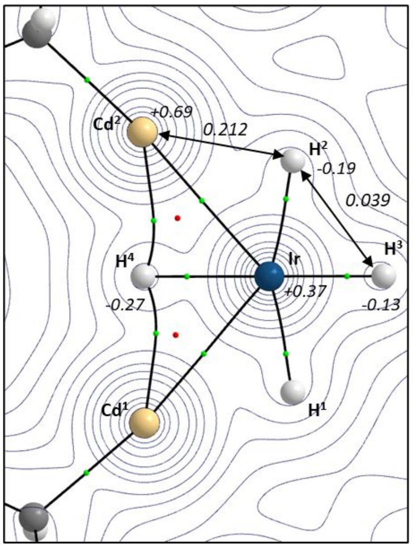

Bond	ρ(r)	$\nabla^2\rho$(r)	ε	H (r)	δ (A,B)
**Ir–H¹**	0.125	0.121	0.049	-0.060	0.750
**Ir-H³**	0.151	0.064	0.038	-0.083	0.869
**Ir-H⁴**	0.096	0.174	0.095	-0.037	0.592
**Ir-Cd¹**	0.054	0.095	0.548	-0.013	0.418

Figure S64. QTAIM molecular graphs with selected BCP metrics (au) tabulated for **8**$^+$. Density contours are shown in the equatorial plane with axial ligands omitted for clarity. BCPs and RCPs shown in green and red respectively, with selected atomic charges shown in italics. Delocalization indices between selected atoms are also indicated.

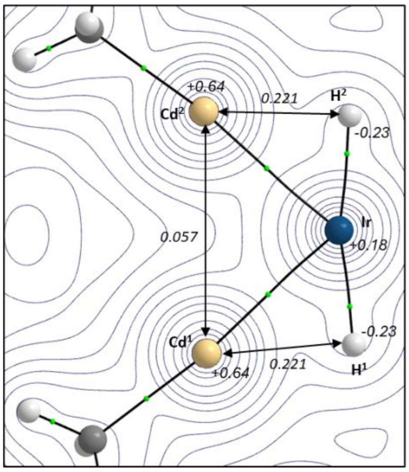

Bond	ρ(r)	$\nabla^2\rho$(r)	ε	H (r)	δ (A,B)
**Ir–H¹**	0.125	0.129	0.073	-0.059	0.769
**Ir-H²**	0.125	0.129	0.073	-0.059	0.769
**Ir-Cd¹**	0.062	0.114	0.072	-0.017	0.656
**Ir-Cd²**	0.062	0.113	0.072	-0.017	0.656

Figure S65. QTAIM molecular graphs with selected BCP metrics (au) tabulated for **9**$^+$. Density contours are shown in the equatorial plane with axial ligands omitted for clarity. BCPs and RCPs shown in green and red respectively, with selected atomic charges shown in italics. Delocalization indices between selected atoms are also indicated.

S2.4 Additional Computed Reaction Profiles

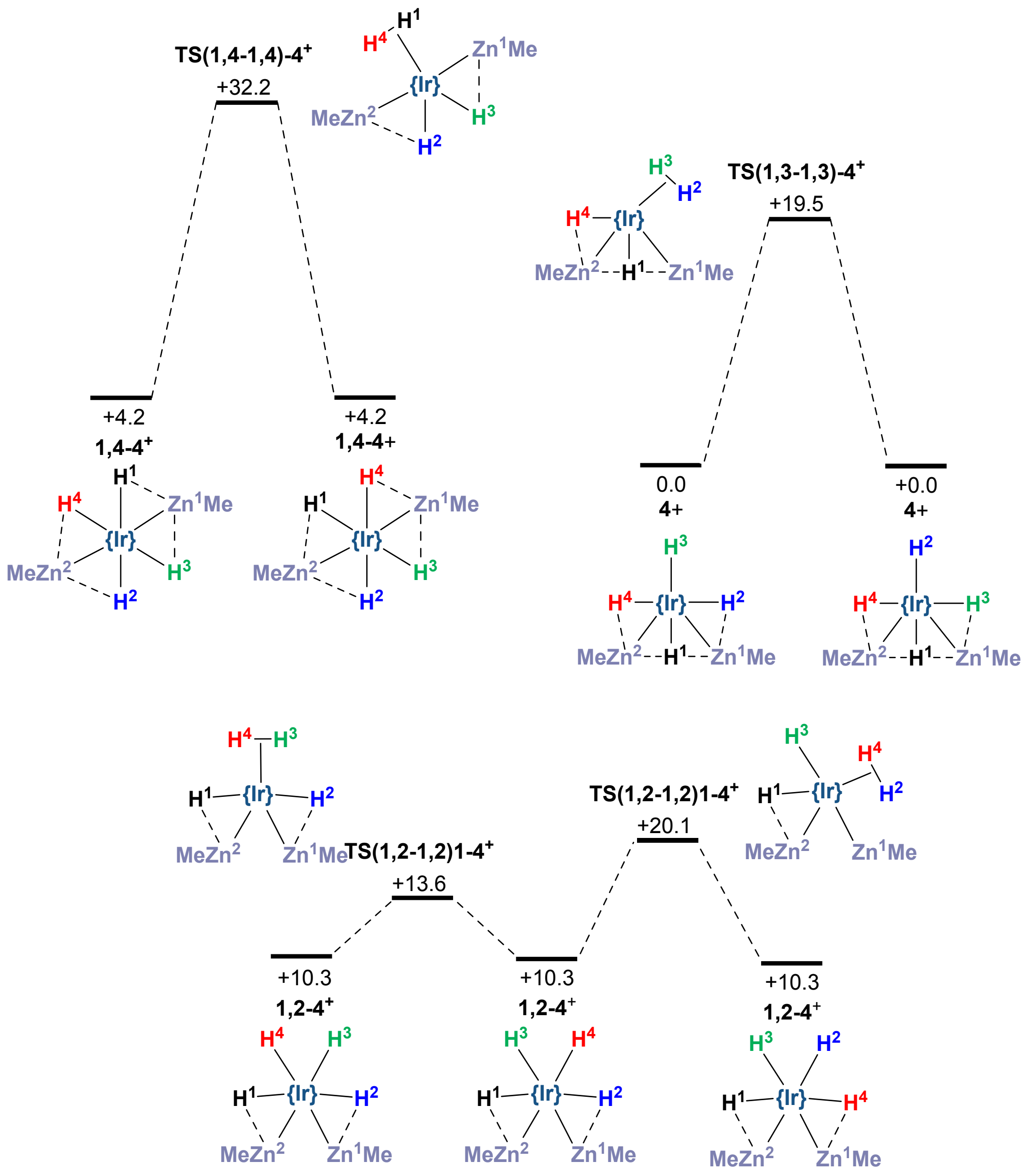

Figure S66. H/H exchange processes computed in **4**+ and its isomers **1**, **2-4**+ and **1**, **4-4**+ (PBE0-D3(C_6H_5F, def2-tzvp)//BP86-D3(SDD(Ir,Zn), 6-31g**; kcal/mol). Axial IPr ligands are omitted for clarity.

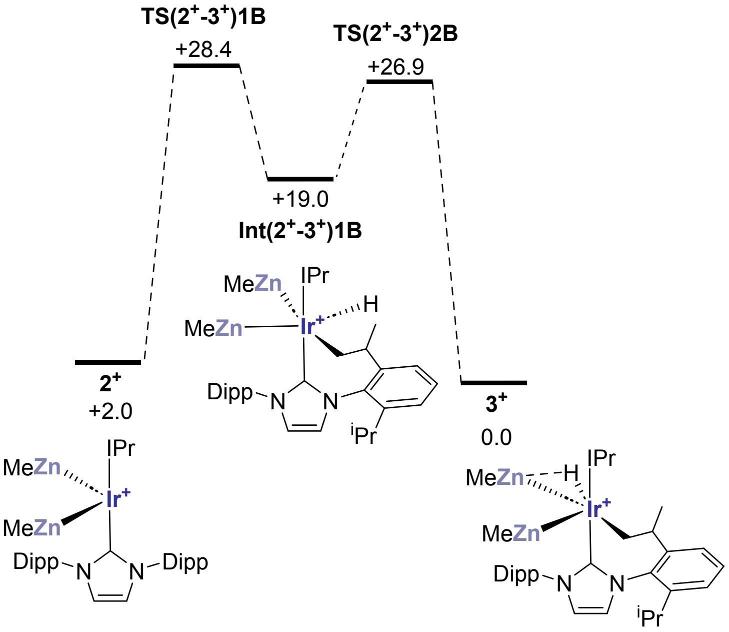

Figure S67. Alternative pathway for cyclometalation of **2**$^+$ (PBE0-D3(C_6H_5F, def2-tzvp)//BP86-D3(SDD(Ir,Zn), 6-31g**; kcal/mol).

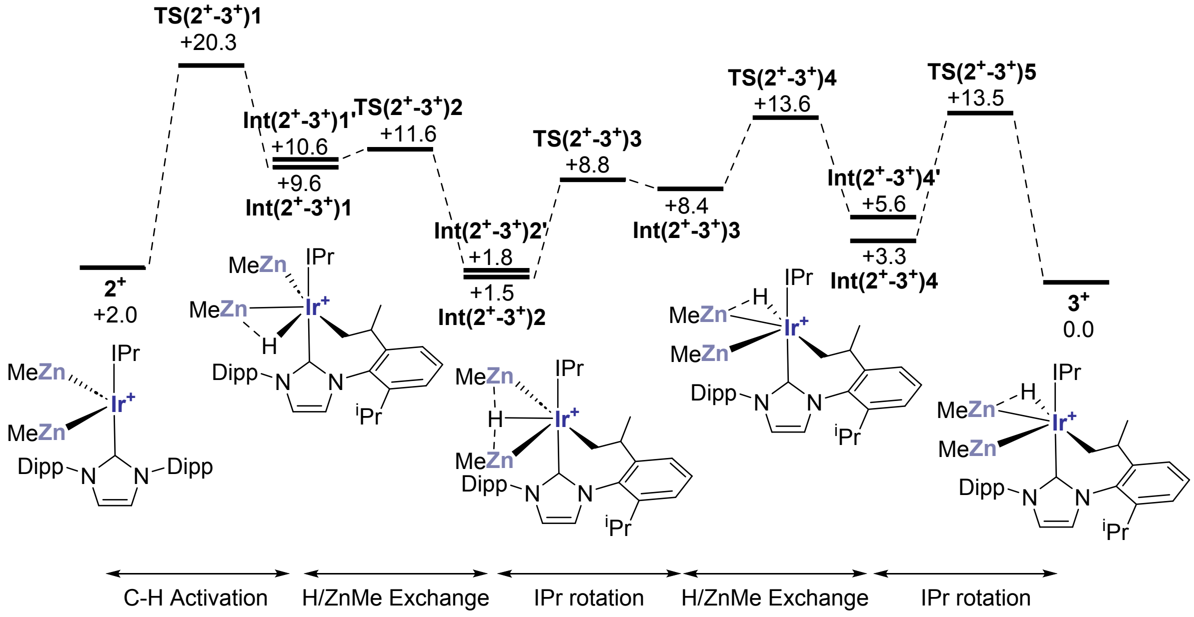

Figure S68. Full pathway for cyclometalation of **2**$^+$ to give **3**$^+$ (PBE0-D3(C_6H_5F, def2-tzvp)//BP86-D3(SDD(Ir,Zn), 6-31g**; kcal/mol). IRC calculations on sequential transition states located two slightly different conformers for intermediates **Int(2$^+$-3$^+$)1**, **Int(2$^+$-3$^+$)2** and **Int(2$^+$-3$^+$)4** and the higher energy form is indicated with a prime.

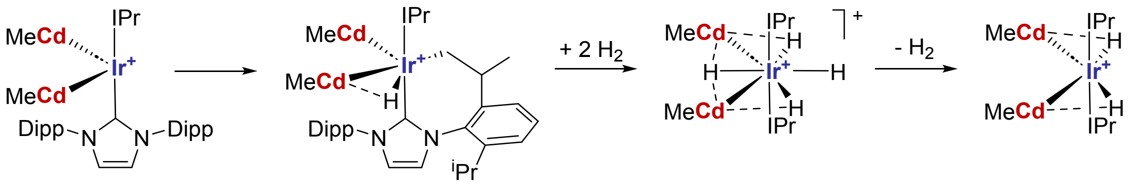

6^+	7^+	8^+	9^+
+2.1	0.0	-28.5	-16.6

Figure S69. Relative free energies of $IrCd_2$ species, **6^+** to **9^+** (PBE0-D3(C_6H_5F, def2-tzvp)//BP86-D3(SDD(Ir,Zn), 6-31g**; kcal/mol).

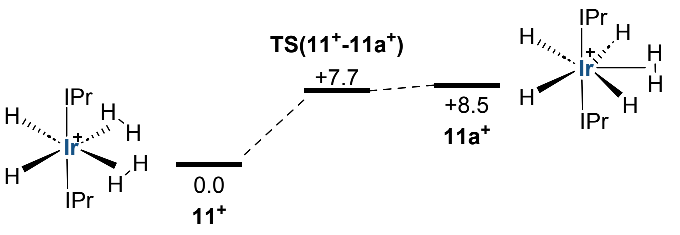

Figure S70. Isomers of **11**$^+$ and their interconversion (PBE0-D3(C_6H_5F, def2-tzvp)//BP86-D3(SDD(Ir,Zn), 6-31g**; kcal/mol). **TS(11**$^+$**-11a**$^+$**)** is a genuine transition state on the electronic surface but falls below **11a**$^+$ once zero-point energy corrections are applied. **11a**$^+$ therefore represents an approximation to the transition state for H-H exchange in **11**$^+$.

S2.5 Computed Structures (A) and Energies (atomic units).

1^+

SCF = -2426.08139691
H(0 K)= -2424.947932
G(298 K)= -2425.044768
SCF (C6H5F) = -2426.12129311
Lowest Frequencies = 18.7452cm-1, 22.0325cm-1
SCF [PBE0(C6H5F, D3)] = -2423.82475176

133

Ir	0.10569	-0.00001	0.00002
C	0.04590	1.90529	0.75992
N	1.07171	2.81522	0.92744
C	2.39302	2.66076	0.37892
C	3.30104	1.81733	1.05465
C	4.57742	1.65875	0.48204
C	4.92839	2.34605	-0.68800
C	4.01429	3.20768	-1.31166
C	2.71757	3.38293	-0.79441
C	1.68169	4.26729	-1.48497
C	2.30308	5.43888	-2.26443
C	0.76957	3.42020	-2.40239
C	2.91003	1.12722	2.35773
C	4.10899	0.92758	3.30614
C	2.24370	-0.23730	2.10135
C	0.66353	3.93586	1.65438
C	-0.65687	3.74384	1.94701
N	-1.01761	2.51854	1.38648
C	-2.34237	1.96392	1.51159
C	-2.64835	1.23993	2.68230
C	-1.60265	0.96491	3.75724
C	-1.42142	-0.54796	3.99394
C	-1.94705	1.70354	5.06712
C	-3.97241	0.78187	2.82428
C	-4.92140	1.00995	1.82049
C	-4.57143	1.70409	0.65359
C	-3.27252	2.21115	0.47579
C	-2.88013	3.02743	-0.75218
C	-3.70113	2.67257	-2.00111
C	-2.98404	4.54352	-0.46609
C	0.04655	-1.90532	-0.75985
N	1.07258	-2.81502	-0.92724
C	0.66478	-3.93571	-1.65430
C	-0.65566	-3.74403	-1.94703
N	-1.01676	-2.51883	-1.38653
C	-2.34172	-1.96469	-1.51153
C	-2.64807	-1.24089	-2.68225
C	-1.60254	-0.96553	-3.75727
C	-1.42178	0.54740	-3.99389
C	-1.94687	-1.70421	-5.06715
C	-3.97229	-0.78329	-2.82414
C	-4.92111	-1.01162	-1.82025
C	-4.57078	-1.70557	-0.65333
C	-3.27168	-2.21218	-0.47561
C	-2.87888	-3.02821	0.75240
C	-2.98203	-4.54438	0.46647
C	-3.70005	-2.67369	2.00132
C	2.39393	-2.66002	-0.37899
C	2.71903	-3.38206	0.79427
C	4.01578	-3.20627	1.31124
C	4.92938	-2.34422	0.68741
C	4.57786	-1.65705	-0.48254
C	3.30144	-1.81618	-1.05489
C	2.90990	-1.12622	-2.35789
C	4.10854	-0.92640	-3.30666
C	2.24338	0.23818	-2.10136
C	1.68364	-4.26683	1.48503
C	0.77122	-3.42003	2.40243
C	2.30565	-5.43802	2.26459
H	5.30896	1.01010	0.96934
H	5.93066	2.22227	-1.11015
H	4.31111	3.74686	-2.21484
H	1.03554	4.69924	-0.69946
H	1.50794	6.10890	-2.62982
H	2.86365	5.09107	-3.14921
H	2.98806	6.03069	-1.63534
H	-0.04427	4.04047	-2.81428
H	0.31957	2.57438	-1.85709
H	1.34674	3.01195	-3.24972
H	2.17772	1.77772	2.87063
H	3.75499	0.58493	4.29202
H	4.80194	0.15892	2.92333
H	4.67370	1.86298	3.44826
H	2.08849	-0.78868	3.04306
H	1.19699	-0.12444	1.69622
H	2.84807	-0.85662	1.43046
H	1.34918	4.74466	1.88981
H	-1.36831	4.34554	2.50429
H	-0.63511	1.35430	3.39525
H	-0.61570	-0.72640	4.72628
H	-2.34101	-1.01062	4.38976
H	-1.15774	-1.06513	3.05645
H	-1.16731	1.52983	5.82768
H	-2.90684	1.35098	5.48178
H	-2.03279	2.79073	4.90411
H	-4.25820	0.23852	3.73051
H	-5.94372	0.63979	1.94559
H	-5.32022	1.85874	-0.12696
H	-1.82209	2.79528	-0.97209
H	-3.27452	3.17640	-2.88413
H	-4.74854	3.00946	-1.91034
H	-3.70186	1.58813	-2.18464
H	-2.69451	5.12088	-1.36059
H	-4.02098	4.81609	-0.20530
H	-2.33002	4.85687	0.36224
H	1.35064	-4.74437	-1.88962
H	-1.36689	-4.34591	-2.50438
H	-0.63487	-1.35466	-3.39541
H	-0.61623	0.72613	-4.72635
H	-2.34158	1.00983	-4.38952
H	-1.15811	1.06458	-3.05641
H	-1.16723	-1.53028	-5.82777
H	-2.90677	-1.35187	-5.48173
H	-2.03233	-2.79143	-4.90416
H	-4.25835	-0.24007	-3.73037
H	-5.94357	-0.64181	-1.94525

H -5.31945 -1.86044 0.12729
H -1.82096 -2.79551 0.97229
H -2.69210 -5.12148 1.36101
H -4.01886 -4.81753 0.20585
H -2.32796 -4.85750 -0.36189
H -3.27320 -3.17731 2.88434
H -4.74730 -3.01105 1.91056
H -3.70125 -1.58924 2.18485
H 4.31302 -3.74533 2.21436
H 5.93169 -2.22001 1.10935
H 5.30903 -1.00807 -0.96998
H 2.17756 -1.77689 -2.87054
H 3.75419 -0.58387 -4.29245
H 4.67338 -1.86171 -3.44888
H 4.80147 -0.15760 -2.92409
H 2.08800 0.78961 -3.04303
H 1.19671 0.12518 -1.69616
H 2.84770 0.85757 -1.43050
H 1.03762 -4.69917 0.69964
H -0.04230 -4.04061 2.81449
H 0.32077 -2.57449 1.85705
H 1.34830 -3.01140 3.24963
H 1.51086 -6.10837 2.63014
H 2.99084 -6.02960 1.63552
H 2.86613 -5.08982 3.14927
H -1.07007 0.31892 -0.96129
H -1.06987 -0.31936 0.96143

2^{+}

SCF = -2959.28973476
H(0 K)= -2958.101808
G(298 K)= -2958.208429
SCF (C6H5F) = -2959.33674528
Lowest Frequencies = 25.9393cm-1, 27.5036cm-1
SCF [PBE0(C6H5F, D3)] = -6060.75499825

141
Ir 0.03610 -0.11182 0.12205
Zn -1.49203 0.34685 -1.64553
Zn 0.79928 2.06900 -0.46565
N -2.18822 1.54649 1.58359
N -0.44076 1.25497 2.82608
N 0.81936 -2.48249 -1.70579
N 1.94975 -0.72567 -2.26797
C -0.93802 0.94322 1.57042
C -1.34921 1.99149 3.58741
H -1.12956 2.29069 4.60765
C -2.45157 2.17534 2.80358
H -3.39722 2.67230 2.99545
C -3.20007 1.39686 0.56931
C -3.40329 2.44925 -0.35572
C -2.54207 3.70603 -0.32731
H -1.58473 3.44004 0.15778
C -2.22330 4.23508 -1.73555
H -1.79728 3.44423 -2.37243
H -1.49565 5.05968 -1.67334
H -3.12230 4.62776 -2.23987
C -3.21139 4.80990 0.52168
H -4.18883 5.08930 0.09258
H -2.57722 5.71195 0.54609
H -3.37839 4.48515 1.56071
C -4.46457 2.30731 -1.27012
H -4.65401 3.09841 -2.00027
C -5.27187 1.16337 -1.26543
H -6.08812 1.07192 -1.98854
C -5.04465 0.13740 -0.33822
H -5.69519 -0.74205 -0.33436
C -4.01223 0.23612 0.61295
C -3.85831 -0.79833 1.72132
H -2.86273 -0.65898 2.17296
C -4.91631 -0.53932 2.81786
H -4.84450 0.48643 3.21527
H -4.77932 -1.24171 3.65733
H -5.93644 -0.67592 2.42002
C -3.92227 -2.24779 1.21732
H -4.87075 -2.47102 0.69992
H -3.84763 -2.94563 2.06839
H -3.09138 -2.46465 0.52967
C 0.77406 0.70129 3.36870
C 1.93071 1.50786 3.43242
C 1.92853 2.94447 2.92352
H 1.06626 3.05383 2.24014
C 3.19687 3.30208 2.12738
H 4.10484 3.23720 2.75065
H 3.12647 4.33766 1.75738
H 3.32848 2.64183 1.25509
C 1.72987 3.92923 4.09758
H 0.79709 3.72536 4.64807
H 1.68876 4.96782 3.72866
H 2.56488 3.85291 4.81496
C 3.07131 0.94410 4.03436
H 3.98528 1.53950 4.11699
C 3.05686 -0.36952 4.51968
H 3.95868 -0.79015 4.97524
C 1.89139 -1.14450 4.44012
H 1.88802 -2.16052 4.84497
C 0.71346 -0.61644 3.88193
C -0.58364 -1.41738 3.83893
H -1.41538 -0.69708 3.74324
C -0.61789 -2.34490 2.61286
H -0.43899 -1.78109 1.66404
H -1.58954 -2.85173 2.51195
H 0.16108 -3.11603 2.68297
C -0.83129 -2.22468 5.12784
H -0.09544 -3.03751 5.25030
H -1.82933 -2.69205 5.09210
H -0.78274 -1.58275 6.02227
C -2.44921 0.56757 -3.32975
H -3.48876 0.23466 -3.19774
H -1.96539 -0.03371 -4.11735
H -2.45195 1.62585 -3.63116
C 1.17354 3.97523 -0.62520
H 0.67199 4.52668 0.18724
H 0.81388 4.34933 -1.59558
H 2.25940 4.13534 -0.56458
C 1.00539 -1.15656 -1.34457
C 1.61962 -2.85378 -2.78648
H 1.60818 -3.86316 -3.18552

```
C   2.33547 -1.74717 -3.14003
H   3.09267 -1.59048 -3.90176
C   0.05151 -3.43275 -0.94056
C  -1.29381 -3.67275 -1.30092
C  -1.90728 -3.01690 -2.53342
H  -1.34055 -2.08977 -2.73778
C  -3.38540 -2.62918 -2.36778
H  -4.02091 -3.50509 -2.15406
H  -3.75645 -2.17544 -3.30062
H  -3.52655 -1.89626 -1.55856
C  -1.72781 -3.94877 -3.75440
H  -0.66800 -4.19752 -3.92554
H  -2.11789 -3.46762 -4.66705
H  -2.27442 -4.89536 -3.60329
C  -1.99422 -4.63808 -0.55382
H  -3.03693 -4.85696 -0.80014
C  -1.36787 -5.33863  0.48577
H  -1.92972 -6.09001  1.04931
C  -0.02089 -5.10430  0.79030
H   0.46276 -5.68210  1.58387
C   0.72362 -4.14244  0.08323
C   2.22003 -3.96334  0.32929
H   2.52935 -3.01825 -0.14801
C   2.99225 -5.11477 -0.35536
H   2.72976 -6.08532  0.09926
H   4.08029 -4.96894 -0.24755
H   2.75998 -5.17622 -1.43088
C   2.61019 -3.86236  1.81490
H   2.17791 -2.96801  2.28881
H   3.70745 -3.79194  1.90516
H   2.29537 -4.75015  2.38991
C   2.64911  0.53227 -2.22128
C   2.29052  1.55518 -3.13124
C   1.14849  1.36949 -4.12297
H   0.48187  0.58666 -3.71607
C   1.68013  0.87813 -5.48784
H   2.37628  1.61647 -5.92124
H   0.84620  0.73684 -6.19561
H   2.21575 -0.08022 -5.40001
C   0.31385  2.64862 -4.30346
H  -0.02798  3.04223 -3.33342
H  -0.57206  2.43757 -4.92317
H   0.88636  3.44488 -4.80844
C   3.06015  2.73462 -3.10842
H   2.81668  3.54808 -3.79688
C   4.12039  2.88620 -2.20678
H   4.69785  3.81571 -2.19952
C   4.45152  1.85403 -1.31736
H   5.29344  1.98043 -0.63098
C   3.73929  0.64082 -1.32193
C   4.12209 -0.51672 -0.40763
H   3.71807 -1.44051 -0.85833
C   3.48210 -0.35097  0.98167
H   3.88011  0.54118  1.48764
H   3.68609 -1.22283  1.62245
H   2.37881 -0.22501  0.90586
C   5.64663 -0.70127 -0.28558
H   6.12627 -0.78757 -1.27419
H   5.86700 -1.61793  0.28610
H   6.11974  0.13797  0.25217
```

3⁺

```
SCF  =           -2959.29008475
H(0 K)=          -2958.104732
G(298 K)=        -2958.208884
SCF (C6H5F) =    -2959.33475062
Lowest Frequencies = 24.1929cm-1,
29.6683cm-1
SCF [PBE0(C6H5F, D3)] =
-6060.75814652

141
Ir  0.02420 -0.13404 -0.20267
Zn -1.56249  0.88506  1.38319
Zn  1.31736  1.79804  0.43734
N   2.55029 -1.61815  0.99302
N   1.13794 -1.11366  2.55141
N  -2.47109  0.29298 -1.99507
N  -1.25035  2.08166 -1.93954
C   1.31773 -1.00848  1.17437
C   3.08952 -2.09380  2.18849
H   4.03263 -2.63040  2.21189
C   2.20224 -1.77076  3.17131
H   2.21237 -1.94989  4.24171
C   3.13481 -1.95038 -0.28279
C   4.20375 -1.16814 -0.77228
C   4.71364 -1.50534 -2.03887
H   5.53660 -0.92219 -2.46124
C   4.16418 -2.56142 -2.77879
H   4.56363 -2.79394 -3.77077
C   3.12153 -3.33449 -2.25014
H   2.72026 -4.17211 -2.82810
C   2.59663 -3.05844 -0.97379
C   4.77787 -0.01069  0.04036
H   4.00013  0.30596  0.76103
C   6.01199 -0.47670  0.84600
H   5.77204 -1.31530  1.51885
H   6.40846  0.34973  1.45940
H   6.81213 -0.81343  0.16500
C   5.14030  1.21720 -0.81616
H   5.97007  1.00291 -1.51056
H   5.46478  2.04396 -0.16341
H   4.28003  1.57089 -1.40748
C   1.49218 -3.91613 -0.36410
H   1.47375 -3.71195  0.72099
C   1.76338 -5.42404 -0.53381
H   1.71435 -5.73225 -1.59194
H   1.00287 -6.00702  0.01200
H   2.75649 -5.70149 -0.14472
C   0.11092 -3.54800 -0.93360
H  -0.12043 -2.46790 -0.79188
H  -0.69148 -4.11599 -0.43742
H   0.06069 -3.75956 -2.01177
C  -0.05608 -0.80489  3.29961
C  -1.06573 -1.79818  3.36305
C  -2.19120 -1.52051  4.15902
H  -2.99077 -2.26326  4.23323
C  -2.29184 -0.31976  4.87694
H  -3.17381 -0.12806  5.49550
C  -1.26617  0.62954  4.81528
```

```
H  -1.35177  1.55644  5.38854
C  -0.12197  0.41056  4.02090
C  -0.89561 -3.14829  2.67709
H  -0.10455 -3.03823  1.91766
C  -0.42359 -4.19862  3.70784
H  -1.18785 -4.35384  4.48851
H   0.50919 -3.88810  4.20639
H  -0.23977 -5.16663  3.21196
C  -2.15677 -3.62237  1.93884
H  -1.97468 -4.61160  1.48609
H  -2.42739 -2.92721  1.13054
H  -3.02277 -3.73021  2.61390
C   1.03243  1.40422  4.01248
H   1.65264  1.18702  3.12397
C   1.91822  1.20654  5.26283
H   2.77232  1.90402  5.24322
H   2.31717  0.18108  5.32260
H   1.34053  1.39753  6.18331
C   0.56021  2.86366  3.90705
H  -0.06808  3.01429  3.01456
H   1.42668  3.53910  3.83258
H  -0.02654  3.17195  4.78840
C  -2.85903  2.08119  2.19959
H  -3.47040  2.57671  1.42700
H  -2.36708  2.85016  2.81279
H  -3.51632  1.48337  2.85022
C   2.57708  3.10536  1.13408
H   3.10697  2.70120  2.01144
H   2.04537  4.02550  1.41846
H   3.30436  3.34404  0.34435
C  -1.29811  0.78461 -1.45958
C  -3.11810  1.24186 -2.79003
H  -4.04328  1.01463 -3.31030
C  -2.35012  2.36958 -2.75626
H  -2.46418  3.33569 -3.23750
C  -2.85683 -1.09446 -1.92585
C  -2.31420 -1.97279 -2.89210
C  -2.69155 -3.32592 -2.81014
H  -2.29982 -4.03796 -3.54322
C  -3.57511 -3.76687 -1.81688
H  -3.85734 -4.82328 -1.77033
C  -4.12451 -2.86014 -0.89951
H  -4.83814 -3.21545 -0.15076
C  -3.77865 -1.49705 -0.93473
C  -1.47056 -1.45945 -4.05575
H  -1.18459 -0.41796 -3.83303
C  -0.16495 -2.24176 -4.28350
H   0.50751 -2.16855 -3.41530
H   0.36662 -1.83172 -5.15857
H  -0.35329 -3.31031 -4.48637
C  -2.33306 -1.43826 -5.33825
H  -2.64011 -2.45917 -5.62296
H  -1.76611 -1.00438 -6.17896
H  -3.24947 -0.84176 -5.19615
C  -4.45003 -0.47594 -0.02361
H  -3.81259  0.42764 -0.00501
C  -4.61566 -0.95227  1.42824
H  -5.10197 -0.16454  2.02620
H  -3.64353 -1.17703  1.89341
H  -5.24630 -1.85462  1.49786
C  -5.81501 -0.06500 -0.62183
H  -6.49635 -0.93187 -0.66589
H  -5.70885  0.32803 -1.64618
H  -6.28928  0.71486 -0.00266
C  -0.12503  2.96937 -1.81764
C   1.09661  2.56177 -2.42192
C   2.15452  3.48625 -2.42565
H   3.10441  3.21152 -2.89133
C   2.00119  4.75994 -1.85506
H   2.83292  5.47044 -1.88063
C   0.80038  5.11635 -1.23436
H   0.70321  6.10101 -0.76760
C  -0.29251  4.22558 -1.19419
C  -1.28825  5.03237  0.97678
H  -0.72455  4.24685  1.50497
H  -2.22972  5.20635  1.52272
H  -0.69537  5.96139  1.02230
C  -1.57960  4.61444 -0.47822
H  -2.22957  3.72221 -0.44111
C  -2.33260  5.73157 -1.23086
H  -1.72278  6.64956 -1.28421
H  -3.27390  5.97968 -0.71297
H  -2.57902  5.43881 -2.26503
C   1.24614  1.14146 -2.93155
H   0.28711  0.84992 -3.39770
C   1.51489  0.19793 -1.72953
H   2.53311  0.43039 -1.37758
H   1.59334 -0.83762 -2.11958
C   2.34126  0.96947 -3.99799
H   2.20086  1.65430 -4.85145
H   2.32398 -0.06568 -4.37539
H   3.34870  1.13591 -3.57958
H  -1.23819 -0.80325  0.71947
```

4⁺

```
SCF  =           -2961.70934369
H(0 K)=          -2960.489385
G(298 K)=        -2960.599346
SCF (C6H5F) =    -2961.75236747
Lowest Frequencies = 10.9915cm-1,
14.0886cm-1
SCF [PBE0(C6H5F, D3)] =
-6063.17548647

145
Ir  0.00422  0.00264  0.00092
H  -1.48435  0.21565  0.74740
H  -0.00997 -1.75496 -0.18026
H   1.49908  0.33496 -0.68545
Zn -1.56074 -1.71320  0.96175
Zn  1.53223 -1.50558 -1.30424
N  -2.02543 -0.31762 -2.34038
N  -0.52536  1.15738 -2.84864
N   0.53534  0.54383  3.02422
N   2.03641 -0.79499  2.22518
C  -0.90022  0.30123 -1.83159
C  -1.39977  1.08314 -3.93612
H  -1.27058  1.71239 -4.81097
C  -2.34644  0.15521 -3.61717
H  -3.21671 -0.20637 -4.15551
```

C	-2.92939	-1.16094	-1.60069
C	-2.80995	-2.56569	-1.72182
C	-1.68454	-3.18751	-2.53922
H	-0.83390	-2.48094	-2.51179
C	-1.20242	-4.52214	-1.94877
H	-1.00015	-4.43147	-0.87035
H	-0.27562	-4.84356	-2.45096
H	-1.94565	-5.32535	-2.08941
C	-2.09942	-3.36529	-4.01648
H	-2.97576	-4.03102	-4.09572
H	-1.27376	-3.81554	-4.59256
H	-2.35624	-2.40425	-4.48907
C	-3.78829	-3.35072	-1.08028
H	-3.73602	-4.43993	-1.15298
C	-4.81729	-2.75527	-0.33895
H	-5.56494	-3.38501	0.15265
C	-4.88788	-1.36135	-0.21004
H	-5.69428	-0.91052	0.37591
C	-3.95036	-0.52680	-0.84854
C	-4.08002	0.99105	-0.80488
H	-3.12880	1.41796	-1.16170
C	-5.19752	1.45218	-1.76642
H	-5.01717	1.10139	-2.79580
H	-5.25429	2.55347	-1.78573
H	-6.17945	1.06486	-1.44481
C	-4.29910	1.53948	0.61326
H	-5.21795	1.14496	1.07976
H	-4.39200	2.63738	0.58008
H	-3.44404	1.30316	1.26367
C	0.45700	2.21028	-2.73474
C	1.77805	1.97452	-3.17508
C	2.15803	0.66580	-3.85704
H	1.47520	-0.11883	-3.47801
C	3.59860	0.20891	-3.57450
H	4.33926	0.92914	-3.96078
H	3.78773	-0.75418	-4.07395
H	3.78320	0.07410	-2.49706
C	1.92117	0.77847	-5.38081
H	0.87569	1.03638	-5.61367
H	2.15600	-0.17688	-5.87965
H	2.56595	1.56181	-5.81444
C	2.69428	3.03428	-3.04788
H	3.72800	2.89424	-3.37497
C	2.29962	4.26996	-2.51648
H	3.03019	5.07939	-2.42349
C	0.97174	4.48129	-2.12848
H	0.66724	5.45796	-1.73978
C	0.01391	3.45538	-2.23578
C	-1.44703	3.72262	-1.88771
H	-2.01858	2.80055	-2.08465
C	-1.64628	4.06631	-0.40100
H	-1.31580	3.24038	0.24687
H	-2.71288	4.25812	-0.19463
H	-1.08397	4.97179	-0.11683
C	-2.02760	4.81987	-2.80493
H	-1.52475	5.78820	-2.64144
H	-3.10212	4.96115	-2.59962
H	-1.91126	4.55803	-3.86981
C	-2.26969	-3.22574	1.94840
H	-3.36006	-3.14023	2.06123

H	-1.80715	-3.29160	2.94726
H	-2.04854	-4.15191	1.39351
C	2.21874	-2.77912	-2.59686
H	1.75947	-2.61850	-3.58646
H	1.98240	-3.80078	-2.25799
H	3.31075	-2.68717	-2.68744
C	0.90878	-0.08910	1.85457
C	1.41679	0.25868	4.07041
H	1.29149	0.70107	5.05349
C	2.36329	-0.58491	3.56890
H	3.23765	-1.04323	4.01994
C	-0.45470	1.59064	3.12830
C	-1.76450	1.26551	3.54475
C	-2.12904	-0.15435	3.96232
H	-1.45891	-0.84520	3.41504
C	-3.57870	-0.54865	3.63356
H	-4.30533	0.07114	4.18527
H	-3.75411	-1.59591	3.92564
H	-3.79799	-0.45612	2.55810
C	-1.85385	-0.34921	5.47129
H	-0.80173	-0.14447	5.72526
H	-2.08210	-1.38486	5.77463
H	-2.48354	0.33188	6.06878
C	-2.68564	2.32417	3.64448
H	-3.71092	2.11714	3.96245
C	-2.30752	3.64101	3.34789
H	-3.04204	4.44814	3.42914
C	-0.99104	3.93142	2.97212
H	-0.69845	4.96618	2.76865
C	-0.02877	2.91052	2.86015
C	1.42465	3.24682	2.54452
H	1.98412	2.29987	2.47163
C	2.04591	4.05855	3.70138
H	1.54528	5.03493	3.81759
H	3.11497	4.24839	3.50671
H	1.96010	3.52264	4.66142
C	1.59191	3.96606	1.19569
H	1.22916	3.34163	0.36595
H	2.65684	4.18876	1.01371
H	1.04401	4.92345	1.17288
C	2.93400	-1.47553	1.32737
C	2.78316	-2.86947	1.13414
C	1.64678	-3.63230	1.80400
H	0.80331	-2.92586	1.91748
C	2.05260	-4.10715	3.21691
H	2.92182	-4.78465	3.16370
H	1.21947	-4.65476	3.68849
H	2.31628	-3.26286	3.87316
C	1.15424	-4.81563	0.95644
H	0.95805	-4.50854	-0.08256
H	0.22157	-5.22089	1.38113
H	1.88847	-5.63895	0.93525
C	3.74469	-3.51314	0.33051
H	3.66801	-4.58964	0.15860
C	4.78834	-2.79029	-0.26332
H	5.52128	-3.31106	-0.88687
C	4.89386	-1.40591	-0.07345
H	5.71358	-0.85361	-0.54285
C	3.97462	-0.71476	0.73964
C	4.14908	0.76853	1.04254

```
H   3.23851  1.11518  1.55710
C   4.29352  1.63101 -0.22135
H   5.16761  1.33882 -0.82816
H   4.42548  2.68896  0.05964
H   3.39231  1.56662 -0.84907
C   5.33973  0.97192  2.00498
H   5.21507  0.38829  2.93207
H   5.43219  2.03600  2.27963
H   6.28719  0.65764  1.53470
H   0.02090  1.60211  0.16453
```

5⁺

```
SCF  =          -2960.50555393
H(0 K)=         -2959.301413
G(298 K)=       -2959.408870
SCF (C6H5F) =   -2960.55052775
Lowest Frequencies = 14.5916cm-1,
24.0539cm-1
SCF [PBE0(C6H5F, D3)] =
-6061.97131132

143
Ir -0.02251  0.09479  0.12859
H   1.46510 -0.38410  0.72959
H  -1.53324  0.75353 -0.25123
Zn  1.19414 -1.87422 -0.58611
Zn -1.38934 -0.25974 -1.85955
N   1.99208  1.24567 -1.96952
N   0.54569  2.74282 -1.38020
N  -2.10831 -1.97409  1.22160
N  -0.47713 -1.67592  2.61296
C  -2.09123 -0.39664 -3.66710
H  -3.16259 -0.14586 -3.63858
H  -1.98061 -1.41776 -4.06001
H  -1.56981  0.30687 -4.33725
C   1.75345 -3.68409 -1.02650
H   1.46995 -3.93293 -2.05982
H   1.28334 -4.40540 -0.33778
H   2.84730 -3.75035 -0.93506
C   0.91260  1.43441 -1.12035
C   2.85356  0.09124 -2.00676
C   2.73022 -0.81703 -3.08452
C   1.66373 -0.64290 -4.15859
H   0.89645  0.04600 -3.76075
C   0.96475 -1.97008 -4.49934
H   1.65492 -2.68914 -4.97159
H   0.13686 -1.79356 -5.20421
H   0.55333 -2.44277 -3.59403
C   2.26620 -0.00398 -5.42915
H   2.72260  0.97669 -5.21937
H   1.48503  0.14073 -6.19401
H   3.04853 -0.65346 -5.85775
C   3.66655 -1.86830 -3.15372
H   3.60893 -2.58507 -3.97740
C   4.65806 -2.01257 -2.17796
H   5.37136 -2.83969 -2.24594
C   4.74375 -1.10581 -1.11053
H   5.52730 -1.23058 -0.35853
C   3.85676 -0.01935 -1.00772
C   4.00661  1.04130  0.07627
H   3.00396  1.45937  0.26360
C   4.92027  2.18588 -0.41862
H   5.93943  1.81360 -0.61941
H   4.99015  2.97457  0.34987
H   4.53728  2.64771 -1.34267
C   4.51011  0.48200  1.41329
H   3.84313 -0.30657  1.78970
H   4.52838  1.28424  2.16866
H   5.53470  0.07825  1.34174
C   2.27264  2.39306 -2.71741
H   3.09184  2.42128 -3.42876
C   1.36361  3.33839 -2.34121
H   1.23394  4.37486 -2.63684
C  -0.35705  3.49891 -0.54844
C  -1.70716  3.63089 -0.94032
C  -2.18960  3.08717 -2.28000
H  -1.49801  2.27680 -2.57877
C  -2.09146  4.19405 -3.35485
H  -2.75882  5.03672 -3.10568
H  -2.38650  3.80110 -4.34236
H  -1.06690  4.59201 -3.43692
C  -3.60757  2.49580 -2.24096
H  -3.67713  1.65251 -1.53647
H  -3.88415  2.12467 -3.24099
H  -4.36039  3.24795 -1.95067
C  -2.54743  4.37471 -0.09262
H  -3.60043  4.50612 -0.35702
C  -2.04996  4.96271  1.07955
H  -2.72214  5.53606  1.72548
C  -0.69520  4.84621  1.41431
H  -0.31425  5.33791  2.31500
C   0.18968  4.11369  0.60072
C   1.68206  4.04812  0.92038
H   2.18937  3.55539  0.07418
C   2.28127  5.46508  1.03926
H   2.08251  6.06251  0.13437
H   3.37353  5.40569  1.18048
H   1.86374  6.01180  1.90175
C   1.98986  3.20810  2.17451
H   1.52333  3.64307  3.07511
H   3.07861  3.17869  2.34993
H   1.63316  2.17057  2.06913
C  -0.93445 -1.25564  1.37689
C  -3.05743 -1.81406  0.14949
C  -4.03494 -0.79819  0.28331
C  -4.11374  0.06940  1.53384
H  -3.14633 -0.00825  2.05674
C  -4.33880  1.55901  1.23044
H  -3.49875  1.97746  0.65644
H  -4.41838  2.12489  2.17399
H  -5.27128  1.73583  0.66795
C  -5.20854 -0.47993  2.47637
H  -6.20317 -0.41504  2.00295
H  -5.23681  0.10214  3.41287
H  -5.02839 -1.53616  2.73570
C  -4.98892 -0.68586 -0.74501
H  -5.76200  0.08511 -0.67765
C  -4.97728 -1.56184 -1.83906
H  -5.73363 -1.46224 -2.62357
C  -4.00643 -2.56626 -1.93442
```

H -4.01011 -3.23993 -2.79484
C -3.01542 -2.71321 -0.94375
C -1.97927 -3.82969 -1.01019
H -1.08397 -3.48237 -0.46141
C -1.54828 -4.16043 -2.44825
H -2.36144 -4.63918 -3.01971
H -0.70142 -4.86449 -2.43383
H -1.23737 -3.25629 -2.99406
C -2.49911 -5.10395 -0.30680
H -2.73121 -4.92433 0.75422
H -1.74086 -5.90335 -0.35640
H -3.41604 -5.46984 -0.79955
C -2.37267 -2.78195 2.33068
H -3.26613 -3.39531 2.38803
C -1.34560 -2.59113 3.20918
H -1.15810 -2.99279 4.20008
C 0.61923 -1.06445 3.32401
C 1.86991 -1.71780 3.35425
C 2.07849 -3.06442 2.67175
H 1.28986 -3.17337 1.90358
C 3.43807 -3.17993 1.96011
H 3.57076 -2.39046 1.20286
H 3.51095 -4.15322 1.44878
H 4.27958 -3.11771 2.67050
C 1.90212 -4.21321 3.69028
H 2.66146 -4.14353 4.48787
H 2.01411 -5.19177 3.19385
H 0.91034 -4.18525 4.16996
C 2.89685 -1.09671 4.08836
H 3.87984 -1.57255 4.14712
C 2.68108 0.12521 4.73978
H 3.49778 0.59466 5.29676
C 1.42313 0.73991 4.69864
H 1.26274 1.68238 5.22975
C 0.35434 0.14700 4.00219
C -1.03484 0.77541 3.98000
H -1.75579 -0.01842 3.71500
C -1.45957 1.32691 5.35494
H -1.37717 0.55956 6.14170
H -2.50682 1.66978 5.31504
H -0.84507 2.19203 5.65682
C -1.13118 1.87326 2.90661
H -0.47761 2.72147 3.15320
H -2.16067 2.25201 2.81140
H -0.82419 1.49825 1.90249

6⁺

SCF = -2840.47725340
H(0 K)= -2839.289882
G(298 K)= -2839.397523
SCF (C6H5F) = -2840.53037927
Lowest Frequencies = 15.8879cm-1, 27.4709cm-1
SCF [PBE0(C6H5F, D3)] = -2837.96588656

141
Ir 0.00288 -0.29700 -0.14644
Cd 1.37845 1.88433 -0.26879
Cd -1.43693 1.16159 1.41846
N 1.74233 -0.46793 2.46355
N -1.78797 1.25678 -2.23074
C 0.72296 -0.95401 1.64955
N 0.15824 -1.94243 2.44663
C 2.74199 0.49914 2.09084
N -0.16263 0.16620 -3.15203
C -0.72911 0.41470 -1.91082
C 0.88842 -0.79910 -3.34868
C 1.78485 -1.12247 3.69910
H 2.52791 -0.87221 4.44980
C 2.21997 -0.34932 -3.49897
C 0.78914 -2.05508 3.68555
H 0.48608 -2.79817 4.41673
C 2.67542 1.79433 2.65854
C -1.87462 1.48765 -3.60726
H -2.66288 2.10063 -4.03285
C 1.22427 -4.22005 1.06238
H 1.74536 -3.47949 1.69146
C -2.80551 1.70745 -1.31745
C -1.57814 3.95543 -1.16321
H -0.72386 3.32251 -1.47034
C -0.27688 -4.02314 1.25893
C -0.79309 -2.90693 1.95852
C -2.16354 -2.74305 2.26151
C 3.20516 -1.33106 -3.71170
H 4.24726 -1.02786 -3.84668
C -2.64048 -1.63605 3.19536
H -1.85558 -0.85596 3.21807
C 2.55616 1.13727 -3.54143
H 1.72881 1.68275 -3.04816
C 3.72238 2.68939 2.35841
H 3.70458 3.69720 2.78256
C -2.77883 -2.19411 4.63052
H -3.55592 -2.97660 4.66900
H -3.06356 -1.39173 5.33173
H -1.83703 -2.64253 4.98638
C -2.72822 3.02378 -0.79939
C 4.77038 2.31145 1.51344
H 5.56962 3.02325 1.28556
C 0.50824 -2.15855 -3.44956
C -0.84896 0.79876 -4.18863
H -0.55449 0.68251 -5.22718
C 3.85592 1.49409 -2.80052
H 4.73649 1.01321 -3.25905
H 3.81648 1.19511 -1.73988
H 4.02022 2.58302 -2.83440
C -3.04838 -3.72018 1.76872
H -4.11785 -3.63435 1.98158
C -2.57659 -4.81164 1.02668
H -3.28305 -5.55922 0.65279
C -4.89233 1.30022 -0.17979
H -5.74026 0.65118 0.05757
C -3.88142 0.82844 -1.03879
C -4.84329 2.59680 0.34963
H -5.64440 2.94658 1.00781
C -1.20508 -4.96983 0.78869
H -0.84763 -5.84685 0.24005
C 4.71622 -1.50398 -0.55579
H 4.38722 -0.82089 -1.35106
H 4.61852 -2.53141 -0.94237

```
H   5.78827 -1.32912 -0.35889
C   1.53455 -3.09888 -3.65053
H   1.28451 -4.16040 -3.73102
C  -1.95434  4.85001 -2.36503
H  -2.21033  4.25388 -3.25508
H  -1.11215  5.51131 -2.62937
H  -2.82379  5.48342 -2.11932
C   2.86915 -2.69021 -3.77028
H   3.65314 -3.43599 -3.93433
C  -1.11954  4.81780  0.02537
H  -1.87895  5.56720  0.30528
H  -0.19863  5.36240 -0.23561
H  -0.91504  4.19960  0.91455
C  -2.41203  2.25841  2.97269
H  -2.04845  1.91692  3.95474
H  -3.49465  2.08653  2.89217
H  -2.20322  3.33129  2.85056
C  -0.95724 -2.57473 -3.36219
H  -1.56914 -1.68602 -3.59882
C   0.95132  3.58407  3.17623
H   0.05588  3.80812  3.77743
H   0.66461  3.60002  2.11198
H   1.67616  4.39957  3.33746
C   3.80040  0.08055  1.23867
C   2.62318  1.62059 -5.00816
H   2.81876  2.70528 -5.04968
H   1.68277  1.42172 -5.54695
H   3.43418  1.10403 -5.54947
C  -4.40593 -1.66047 -0.78375
H  -5.36381 -1.46226 -0.27368
H  -3.63253 -1.82969 -0.01979
H  -4.53034 -2.59829 -1.35072
C  -3.77801  3.45064  0.03732
H  -3.75326  4.45900  0.45853
C   2.29940  3.78310 -0.61463
H   3.38972  3.64574 -0.62806
H   2.02919  4.47183  0.19924
H   1.95931  4.19288 -1.57885
C   1.98115  2.22667  5.05193
H   2.79420  2.95661  5.20592
H   2.35109  1.24099  5.37699
H   1.14070  2.50875  5.70790
C  -3.95150 -0.96616  2.75181
H  -3.85340 -0.48982  1.76334
H  -4.23419 -0.18448  3.47513
H  -4.78516 -1.68655  2.70035
C   4.80221  1.02559  0.95199
H   5.62875  0.74642  0.29386
C   1.53173  2.21468  3.57479
H   0.72423  1.46643  3.47023
C  -4.00392 -0.52228 -1.73375
H  -3.01425 -0.77958 -2.14497
C   1.67871 -5.61520  1.53610
H   1.37864 -5.80314  2.57990
H   2.77684 -5.69721  1.47317
H   1.25173 -6.41832  0.91165
C  -1.32411 -3.66301 -4.38973
H  -0.82899 -4.62291 -4.16430
H  -1.04131 -3.36584 -5.41266
H  -2.41130 -3.84587 -4.37135
C  -1.32718 -3.03139 -1.94012
H  -2.40266 -3.25370 -1.86143
H  -1.08878 -2.25046 -1.18193
H  -0.77305 -3.93851 -1.66194
C   3.86714 -1.35027  0.71356
H   2.83443 -1.64647  0.45836
C  -4.99072 -0.40205 -2.91738
H  -5.04458 -1.35481 -3.47068
H  -4.68077  0.38643 -3.62290
H  -6.00549 -0.15536 -2.56095
C   4.39199 -2.30950  1.80800
H   5.42264 -2.03856  2.09485
H   4.40751 -3.34499  1.42709
H   3.76857 -2.29388  2.71492
C   1.65852 -3.95987 -0.39139
H   1.20307 -4.68924 -1.08232
H   2.75353 -4.05084 -0.48867
H   1.36820 -2.94929 -0.72696
```

7⁺

```
SCF  =            -2840.47823459
H(0 K)=           -2839.294946
G(298 K)=         -2839.401377
SCF (C6H5F) =    -2840.52820648
Lowest Frequencies = 24.1281cm-1,
29.5245cm-1
SCF [PBE0(C6H5F, D3)] =
-2837.96635006

141
Ir -0.00967 -0.30886 -0.20257
H   1.37760 -0.52127  0.74334
Cd -1.67799  1.68165  0.05768
Cd  1.59638  1.50029  0.91826
N  -2.21208 -1.69401  1.59662
N  -0.83750 -0.56187  2.82304
N   0.82161  1.42111 -2.62839
N   2.30592 -0.11251 -2.25689
C  -1.08041 -0.89606  1.48886
C  -4.51844 -2.76821 -1.12451
H  -5.44137 -2.44855 -1.61654
C  -2.79947 -2.46117  0.52688
C  -2.14006 -3.64878  0.14058
C  -0.89421 -4.13849  0.87231
H  -0.85872 -3.62155  1.84754
C   0.39168 -3.78055  0.10660
H   0.42928 -4.29933 -0.86253
H   1.28899 -4.06447  0.67849
H   0.46439 -2.68745 -0.09241
C  -0.94658 -5.65076  1.16598
H  -1.86994 -5.92635  1.70094
H  -0.08521 -5.94303  1.78954
H  -0.89797 -6.24927  0.24049
C  -2.68468 -4.35947 -0.94531
H  -2.19557 -5.27533 -1.28979
C  -3.85721 -3.91917 -1.57470
H  -4.26952 -4.48812 -2.41373
C  -3.99746 -2.00452 -0.06465
C  -4.69664 -0.74125  0.43052
H  -3.94815 -0.14353  0.98554
```

C	-5.23917	0.14322	-0.70815
H	-4.44592	0.42088	-1.42129
H	-5.65945	1.07285	-0.29081
H	-6.04464	-0.35907	-1.26980
C	-5.83355	-1.10400	1.41226
H	-6.60370	-1.70910	0.90417
H	-6.31585	-0.19174	1.80176
H	-5.46385	-1.68770	2.27051
C	-2.62930	-1.85950	2.91724
H	-3.47986	-2.48473	3.16973
C	-1.76611	-1.14218	3.68974
H	-1.70761	-0.99613	4.76362
C	0.32182	0.10409	3.36379
C	1.47975	-0.67964	3.59835
C	1.49657	-2.17599	3.31122
H	0.67486	-2.38877	2.60861
C	1.22343	-2.95774	4.61614
H	2.02681	-2.78605	5.35290
H	1.17358	-4.04035	4.41045
H	0.27019	-2.65422	5.07898
C	2.79103	-2.65334	2.63618
H	2.92358	-2.17384	1.65524
H	2.74835	-3.74350	2.47426
H	3.68515	-2.45316	3.25090
C	2.58430	-0.03708	4.18661
H	3.49526	-0.60996	4.38210
C	2.52734	1.31718	4.54718
H	3.39685	1.79523	5.00822
C	1.36043	2.05808	4.32888
H	1.32555	3.11069	4.62184
C	0.22781	1.46883	3.72885
C	-1.06099	2.26112	3.54646
H	-1.67989	1.72572	2.80179
C	-0.81133	3.68388	3.01611
H	-0.27245	4.30630	3.74975
H	-1.76874	4.18166	2.79703
H	-0.21347	3.66910	2.08945
C	-1.86136	2.30658	4.86694
H	-2.11080	1.29584	5.22766
H	-2.80455	2.86050	4.72566
H	-1.28091	2.81354	5.65654
C	-3.19082	3.12595	0.47464
H	-3.97633	3.02732	-0.28798
H	-2.75178	4.13265	0.42096
H	-3.61460	2.95297	1.47583
C	2.81015	3.19961	1.34580
H	3.31054	3.55789	0.43226
H	3.56625	2.89796	2.08639
H	2.19573	4.00839	1.76714
C	1.09067	0.35153	-1.78995
C	-0.42315	2.13870	-2.67693
C	-1.58916	1.39898	-3.01810
C	-1.57527	-0.69204	-1.63686
H	-1.47278	-1.79510	-1.71175
H	-2.60730	-0.54284	-1.28493
C	-0.43568	3.53175	-2.44364
C	0.79959	4.28767	-1.97007
H	1.58552	3.54452	-1.74395
C	1.33572	5.24046	-3.05888
H	1.58474	4.70249	-3.98864
H	2.24522	5.75595	-2.70830
H	0.58643	6.00967	-3.31250
C	0.50354	5.05332	-0.66478
H	-0.24033	5.85252	-0.82076
H	1.42322	5.51832	-0.27459
H	0.10881	4.37431	0.10930
C	-1.66127	4.20646	-2.63044
H	-1.70616	5.28720	-2.46719
C	-2.81587	3.51460	-3.00880
H	-3.75385	4.05889	-3.15400
C	-2.78493	2.12031	-3.17938
H	-3.69850	1.58734	-3.45478
C	1.83056	1.59797	-3.58275
H	1.77290	2.37904	-4.33427
C	2.76421	0.63061	-3.34774
H	3.69301	0.38706	-3.85399
C	2.91222	-1.34330	-1.81419
C	3.94267	-1.28916	-0.84989
C	4.49703	0.04330	-0.35893
H	3.73356	0.81883	-0.56263
C	4.79946	0.07230	1.14864
H	3.90297	-0.15163	1.74848
H	5.16073	1.07232	1.43886
H	5.58160	-0.65482	1.42477
C	5.75605	0.41848	-1.17304
H	6.55626	-0.32431	-1.01360
H	6.13675	1.40646	-0.86391
H	5.54503	0.45483	-2.25424
C	4.50387	-2.51308	-0.44246
H	5.30642	-2.51667	0.30068
C	4.05543	-3.72635	-0.98272
H	4.50463	-4.66724	-0.64997
C	3.05904	-3.74057	-1.96706
H	2.74737	-4.69179	-2.40944
C	2.46645	-2.54485	-2.41290
C	1.49999	-2.53574	-3.59419
H	1.03783	-1.53602	-3.64511
C	0.35040	-3.55308	-3.48870
H	0.72005	-4.58920	-3.39819
H	-0.26993	-3.50664	-4.39945
H	-0.30074	-3.34152	-2.62705
C	2.30028	-2.74415	-4.90059
H	3.09873	-1.99133	-5.00773
H	1.63581	-2.66902	-5.77774
H	2.77597	-3.73973	-4.91468
C	-1.52570	-0.11497	-3.07881
H	-0.55493	-0.38795	-3.53197
C	-2.62724	-0.75202	-3.94375
H	-3.62736	-0.61206	-3.49864
H	-2.45960	-1.83938	-4.00617
H	-2.64092	-0.34213	-4.96804

8^+

SCF = -2842.89819645
H(0 K)= -2841.680345
G(298 K)= -2841.789416
SCF (C6H5F) = -2842.94612638
Lowest Frequencies = -2.4593cm-1, 24.4974cm-1
SCF [PBE0(C6H5F, D3)] =

```
-2840.38360425896

145
Ir -0.00997 -0.13081  0.00371
H  -1.62661 -0.41744 -0.27491
H   0.03687  1.64250  0.03176
H   1.59710 -0.50375  0.27974
H  -0.05364 -1.73673 -0.01815
Cd -1.85080  1.70958 -0.76470
Cd  1.96048  1.56070  0.79370
N  -1.32435  0.39453  2.79730
N   0.30746 -1.01563  2.97295
N  -0.36020 -0.90600 -2.99817
N   1.29700  0.46718 -2.77348
C  -0.36891 -0.25336  2.03523
C  -2.41834  1.18041  2.28911
C  -3.61090  0.50177  1.94258
C  -3.76077 -1.00384  2.12340
H  -2.77494 -1.40844  2.40307
C  -4.73286 -1.30172  3.28552
H  -4.40420 -0.81792  4.22039
H  -4.79386 -2.38833  3.46369
H  -5.74948 -0.93771  3.05834
C  -4.18484 -1.72405  0.83244
H  -5.16019 -1.36586  0.46069
H  -4.27952 -2.80667  1.01931
H  -3.43470 -1.58870  0.03846
C  -4.68901  1.28741  1.48758
H  -5.62720  0.79755  1.20928
C  -4.58319  2.68235  1.41211
H  -5.43501  3.27544  1.06616
C  -3.38916  3.32546  1.77023
H  -3.31845  4.41346  1.69457
C  -2.27286  2.58677  2.21104
C  -0.96766  3.26057  2.61697
H  -0.15544  2.53630  2.41745
C  -0.67558  4.52801  1.79774
H  -1.34550  5.35939  2.07580
H  -0.79469  4.34538  0.71779
H   0.35794  4.86484  1.97988
C  -0.95026  3.57391  4.12945
H  -1.77138  4.26221  4.39271
H   0.00262  4.05422  4.40845
H  -1.05998  2.66171  4.73678
C  -1.25781  0.02637  4.14535
H  -1.95515  0.42110  4.87753
C  -0.23090 -0.86322  4.25337
H   0.15799 -1.41690  5.10179
C   1.25609 -2.06279  2.67310
C   0.73082 -3.33077  2.33700
C  -0.76582 -3.61837  2.37438
H  -1.29224 -2.65593  2.47870
C  -1.10377 -4.47112  3.61675
H  -0.61521 -5.45911  3.56327
H  -2.19264 -4.63374  3.68748
H  -0.76782 -3.98171  4.54620
C  -1.29191 -4.26686  1.08497
H  -1.12973 -3.60872  0.21950
H  -2.37593 -4.45182  1.17016
H  -0.80639 -5.23663  0.88096
C   1.65287 -4.35623  2.05828
H   1.28223 -5.35138  1.79428
C   3.03136 -4.12258  2.12681
H   3.73351 -4.93224  1.90535
C   3.51795 -2.86382  2.50428
H   4.59686 -2.70490  2.58253
C   2.63919 -1.80305  2.79200
C   3.15539 -0.46489  3.30868
H   2.38517  0.30033  3.08499
C   3.31363 -0.51289  4.84584
H   2.36502 -0.76147  5.34727
H   3.65704  0.46240  5.22995
H   4.05613 -1.27718  5.13192
C   4.47515 -0.01459  2.65792
H   5.30452 -0.69751  2.90707
H   4.75112  0.98600  3.02609
H   4.40006  0.03798  1.55939
C  -2.98199  3.23171 -1.72990
H  -2.87507  3.16178 -2.82390
H  -2.62424  4.21715 -1.39285
H  -4.04162  3.12453 -1.45633
C   3.12121  3.07396  1.73678
H   4.18282  2.93326  1.48815
H   2.99064  3.03678  2.82980
H   2.79367  4.05749  1.36519
C   0.34009 -0.20358 -2.03225
C  -1.29766 -1.97398 -2.74002
C  -0.76244 -3.26787 -2.54962
C   0.72981 -3.55991 -2.67639
H   1.24283 -2.61469 -2.91843
C   1.34461 -4.08340 -1.36622
H   1.23904 -3.34837 -0.55387
H   2.41975 -4.28768 -1.50675
H   0.86656 -5.02352 -1.04299
C   0.99104 -4.52858 -3.84938
H   0.53329 -5.51556 -3.66610
H   2.07509 -4.68165 -3.98432
H   0.57775 -4.13768 -4.79406
C  -1.67506 -4.31041 -2.30217
H  -1.29678 -5.32502 -2.14322
C  -3.05296 -4.06742 -2.26144
H  -3.74616 -4.89057 -2.06288
C  -3.55174 -2.78006 -2.50222
H  -4.63216 -2.61188 -2.50313
C  -2.68332 -1.70304 -2.75747
C  -3.21584 -0.33193 -3.15419
H  -2.41692  0.40811 -2.94990
C  -3.48670 -0.29237 -4.67551
H  -2.58222 -0.53497 -5.25626
H  -3.83141  0.70984 -4.98133
H  -4.26682 -1.02362 -4.94763
C  -4.47055  0.09807 -2.37578
H  -5.32288 -0.57317 -2.57433
H  -4.77216  1.11202 -2.68233
H  -4.29616  0.10830 -1.28769
C   0.15234 -0.68306 -4.27897
H  -0.26255 -1.18076 -5.14970
C   1.19627  0.18207 -4.13962
H   1.88112  0.61414 -4.86233
C   2.41386  1.20886 -2.24781
```

C   2.33630 2.62031 -2.20650
C   1.06154 3.34920 -2.61050
H   0.22773 2.62966 -2.50458
C   0.76396 4.54607 -1.69174
H   0.82312 4.25615 -0.63064
H -0.24852 4.93375 -1.89051
H   1.47226 5.37581 -1.85508
C   1.11366 3.78961 -4.08938
H   1.95521 4.48269 -4.25933
H   0.18095 4.30809 -4.36790
H   1.23910 2.92972 -4.76652
C   3.48828 3.31838 -1.79041
H   3.46964 4.41067 -1.74931
C   4.64973 2.63022 -1.41648
H   5.53249 3.19074 -1.09468
C   4.68310 1.22806 -1.43404
H   5.59388 0.70464 -1.12961
C   3.56712 0.48054 -1.85860
C   3.61493 -1.03907 -1.96383
H   2.57416 -1.40100 -1.97765
C   4.29181 -1.71073 -0.76020
H   5.34556 -1.40573 -0.64139
H   4.27893 -2.80539 -0.88778
H   3.75034 -1.48648 0.17074
C   4.28420 -1.45861 -3.29155
H   3.76534 -1.02570 -4.16227
H   4.26682 -2.55658 -3.39628
H   5.33655 -1.12817 -3.32386

9$^+$

SCF =   -2841.69476232
H(0 K)=   -2840.492674
G(298 K)=   -2840.600427
Lowest Frequencies = -6.5452cm-1, 22.7459cm-1
SCF [PBE0(C6H5F, D3)] = -2839.18029312317

143
C -3.82132 0.45473 1.33167
C -2.72446 1.16011 1.88766
C -2.63138 2.57325 1.88449
C -3.69331 3.28929 1.29547
C -4.78113 2.62071 0.72189
C -4.84040 1.21969 0.73410
N -1.70207 0.39556 2.55474
C -0.65721 -0.28941 1.94757
N -0.08094 -0.96225 3.01298
C -0.74696 -0.72043 4.21489
C -1.76661 0.13940 3.92752
C   0.91639 -1.99372 2.87070
C   0.46060 -3.29345 2.55326
C   1.43749 -4.29625 2.41055
C   2.79489 -4.00983 2.60170
C   3.20580 -2.71930 2.96394
C   2.27204 -1.67765 3.10839
Ir 0.00009 -0.29077 0.00027
C   0.65729 -0.29332 -1.94705
N   1.70206 0.39062 -2.55553
C   1.76642 0.13217 -3.92788
C   0.74683 -0.72827 -4.21363
N   0.08098 -0.96809 -3.01122
C   2.72426 1.15671 -1.88996
C   3.82135 0.45274 -1.33266
C   4.84020 1.21917 -0.73658
C   4.78050 2.62020 -0.72707
C   3.69248 3.28733 -1.30192
C   2.63075 2.56980 -1.88952
C -0.91606 -1.99956 -2.86697
C -0.45991 -3.29851 -2.54685
C -1.43650 -4.30131 -2.40218
C -2.79398 -4.01569 -2.59400
C -3.20525 -2.72602 -2.95888
C -2.27179 -1.68439 -3.10539
C   3.93713 -1.06068 -1.46374
C   4.46309 -1.74480 -0.19380
C   1.45315 3.29134 -2.53379
C   1.79423 3.72296 -3.97693
C   1.02812 -3.60710 -2.40135
C   1.46135 -3.67807 -0.92526
C -2.68792 -0.30025 -3.58941
C -3.99961 0.20514 -2.96418
C -1.02735 -3.60282 2.40857
C -1.42604 -4.88813 3.16087
C   2.68778 -0.29242 3.58957
C   3.99943 0.21199 2.96345
C -1.45397 3.29636 2.52735
C -1.79424 3.72868 3.97049
C -3.93665 -1.05849 1.46559
C -4.80952 -1.40500 2.69329
Cd 1.51486 1.64855 0.91662
C   2.41730 3.30469 1.91338
Cd -1.51496 1.64636 -0.91978
C -2.41856 3.29995 -1.91975
C   4.80982 -1.40922 -2.69100
C   0.98382 4.50005 -1.70595
C   1.42731 -4.89389 -3.15085
C -2.78426 -0.28581 -5.13184
C -1.46073 -3.67706 0.93269
C   2.78390 -0.27472 5.13198
C -0.98637 4.50508 1.69852
C -4.46214 -1.74515 0.19682
H   1.58285 -0.47306 0.52632
H -1.58238 -0.47525 -0.52558
H   2.92482 -1.45676 -1.64345
H   4.40822 -0.96161 -3.61486
H   4.85161 -2.50254 -2.83187
H   5.84155 -1.04134 -2.55728
H   5.46496 -1.38473 0.09587
H   4.54656 -2.83154 -0.36025
H   3.77373 -1.59098 0.64910
H   5.70274 0.71268 -0.29450
H   5.59016 3.19603 -0.26862
H   3.65782 4.37976 -1.28480
H   0.61316 2.57316 -2.58655
H   1.72929 5.31294 -1.70766
H   0.79877 4.21798 -0.65682
H   0.04991 4.90684 -2.12439
H   2.64681 4.42337 -3.98086
H   0.93150 4.23076 -4.43977

H   2.06100 2.86174 -4.60965
H   2.53433 0.57489 -4.55419
H   0.44605 -1.21072 -5.13844
H   1.58785 -2.77743 -2.86809
H   0.97868 -5.79104 -2.69137
H   2.52194 -5.02377 -3.11909
H   1.11392 -4.86104 -4.20706
H   1.21092 -2.75017 -0.37683
H   2.55001 -3.83201 -0.84864
H   0.97003 -4.51744 -0.40952
H -1.12773 -5.32049 -2.15187
H -3.53803 -4.81011 -2.48016
H -4.26610 -2.52827 -3.13676
H -1.88448 0.40678 -3.30425
H -1.83428 -0.58749 -5.60189
H -3.04054 0.72384 -5.49471
H -3.56542 -0.98432 -5.47723
H -4.85698 -0.43154 -3.24004
H -4.21672 1.22393 -3.32360
H -3.94389 0.23798 -1.86395
H   2.05876 3.36357 2.95304
H   2.17321 4.23626 1.38272
H   3.50678 3.15560 1.90159
H -3.50784 3.14935 -1.90900
H -2.05892 3.35792 -2.95909
H -2.17639 4.23256 -1.39006
H -1.58728 -2.77232 2.87358
H -1.21066 -2.75026 0.38227
H -2.54935 -3.83150 0.85655
H -0.96923 -4.51739 0.41867
H -0.97719 -5.78612 2.70325
H -2.52063 -5.01842 3.12952
H -1.11252 -4.85293 4.21697
H   1.12900 -5.31602 2.16234
H   3.53918 -4.80426 2.48942
H   4.26661 -2.52087 3.14135
H   1.88420 0.41382 3.30285
H   1.83390 -0.57553 5.60252
H   3.04000 0.73572 5.49276
H   3.56511 -0.97241 5.47894
H   4.85696 -0.42373 3.24102
H   4.21608 1.23171 3.32049
H   3.94397 0.24218 1.86312
H -0.44621 -1.20121 5.14058
H -2.53473 0.58298 4.55295
H -0.61329 2.57897 2.57991
H -0.80257 4.22298 0.64920
H -0.05213 4.91244 2.11568
H -1.73223 5.31761 1.70117
H -2.64762 4.42811 3.97461
H -0.93166 4.23788 4.43210
H -2.05937 2.86769 4.60418
H -3.65898 4.38169 1.27617
H -5.59094 3.19542 0.26229
H -5.70276 0.71208 0.29296
H -2.92426 -1.45393 1.64624
H -5.46406 -1.38594 -0.09373
H -4.54531 -2.83160 0.36529
H -3.77265 -1.59270 -0.64623
H -4.40831 -0.95551 3.61640
H -4.85098 -2.49806 2.83622
H -5.84134 -1.03772 2.55861

Int(3^+-5^+)1

SCF =   -2960.47294635
H(0 K)=   -2959.272407
G(298 K)=   -2959.377991
SCF (C6H5F) =   -2960.51770964
Lowest Frequencies = 19.6956cm-1, 31.8196cm-1
SCF [PBE0(C6H5F, D3)] = -6061.93803248

143
Ir -0.10565 0.13232 -0.19847
Zn 1.80396 0.33145 1.37908
Zn 0.63206 -2.12729 0.40093
N -2.88366 -0.96239 0.86960
N -1.60241 -0.37123 2.50939
N   1.83696 1.84228 -1.91231
N   2.47166 -0.22909 -1.90601
C -1.62730 -0.43852 1.12148
C -3.60057 -1.19920 2.04461
H -4.60918 -1.59857 2.02260
C -2.79385 -0.82714 3.07722
H -2.95008 -0.81901 4.15114
C -3.49347 -1.10335 -0.43130
C -3.58468 -2.39154 -1.00236
C -4.15681 -2.48411 -2.28478
H -4.23950 -3.46181 -2.76733
C -4.59649 -1.33991 -2.96098
H -5.02003 -1.43162 -3.96592
C -4.51593 -0.07833 -2.35312
H -4.88715 0.80164 -2.88495
C -3.97997 0.06846 -1.06091
C -3.10024 -3.63448 -0.25969
H -2.37153 -3.30604 0.50489
C -4.28217 -4.31968 0.46480
H -4.78938 -3.63980 1.16743
H -3.93062 -5.19687 1.03333
H -5.03399 -4.66327 -0.26606
C -2.38791 -4.65511 -1.16790
H -3.07617 -5.09607 -1.90858
H -1.99089 -5.48191 -0.55672
H -1.54426 -4.19999 -1.71096
C -4.00125 1.41078 -0.33241
H -3.12334 1.44185 0.33508
C -5.26250 1.51083 0.55668
H -6.17625 1.47266 -0.06029
H -5.26330 2.46364 1.11321
H -5.31186 0.69097 1.29041
C -3.90758 2.63324 -1.25897
H -3.02149 2.59138 -1.90998
H -3.83287 3.55114 -0.65306
H -4.80142 2.73661 -1.89777
C -0.63352 0.33436 3.31098
C -0.81544 1.72868 3.46964
C   0.11742 2.41210 4.27048
H   0.00922 3.49163 4.41447
C   1.16824 1.72894 4.89838

H   1.88375 2.27944 5.51658
C   1.29664 0.34222 4.75412
H   2.10794 -0.18191 5.26607
C   0.39482 -0.39117 3.95629
C -2.01102 2.45871 2.86813
H -2.55970 1.74398 2.23393
C -2.97494 2.91030 3.98629
H -2.49285 3.64109 4.65785
H -3.30559 2.05634 4.60041
H -3.86921 3.38777 3.55172
C -1.60182 3.63698 1.96743
H -2.49793 4.10340 1.52416
H -0.94858 3.30579 1.14604
H -1.06720 4.41930 2.53290
C   0.45660 -1.91155 3.88117
H -0.09988 -2.22251 2.97851
C -0.25580 -2.53096 5.10473
H -0.24886 -3.63168 5.03678
H -1.30458 -2.20056 5.17709
H   0.25366 -2.24068 6.03946
C   1.88811 -2.45752 3.75755
H   2.40890 -2.02409 2.88788
H   1.86596 -3.55076 3.62799
H   2.49270 -2.23900 4.65370
C   3.51938 0.68866 2.22246
H   4.29593 0.91978 1.47424
H   3.85892 -0.16028 2.83391
H   3.38999 1.56130 2.88273
C   0.70096 -4.00976 0.89929
H   0.05751 -4.21267 1.77159
H   1.73442 -4.30084 1.14420
H   0.35958 -4.62593 0.05394
C   1.48781 0.60595 -1.40930
C   2.98294 1.77556 -2.70777
H   3.38729 2.65507 -3.19896
C   3.38118 0.47051 -2.70843
H   4.20330 -0.03332 -3.20602
C   1.00968 3.01526 -1.78968
C -0.00721 3.20323 -2.75345
C -0.84620 4.32110 -2.58941
H -1.64379 4.50568 -3.31581
C -0.66401 5.20448 -1.51699
H -1.32652 6.06844 -1.40610
C   0.37291 5.00124 -0.59590
H   0.51315 5.71080 0.22409
C   1.23512 3.89487 -0.70945
C -0.15395 2.28064 -3.96005
H   0.51984 1.41963 -3.81318
C -1.57635 1.71450 -4.12781
H -1.88967 1.12021 -3.25333
H -1.61520 1.05159 -5.00837
H -2.32182 2.51303 -4.28197
C   0.30586 3.01481 -5.23887
H -0.33953 3.88477 -5.44896
H   0.26226 2.33961 -6.10982
H   1.34043 3.38268 -5.13887
C   2.41363 3.69102 0.23445
H   2.69340 2.62209 0.19401
C   2.09115 4.02278 1.69933
H   2.97314 3.82525 2.32991
H   1.26147 3.40554 2.07589
H   1.82344 5.08453 1.83460
C   3.62928 4.50553 -0.26293
H   3.40491 5.58588 -0.25117
H   3.90643 4.23213 -1.29435
H   4.50374 4.32809 0.38533
C   2.45797 -1.66828 -1.83830
C   1.38314 -2.33838 -2.48255
C   1.44156 -3.74101 -2.54719
H   0.63470 -4.28790 -3.04140
C   2.52497 -4.44390 -2.00013
H   2.55745 -5.53518 -2.07144
C   3.55281 -3.75780 -1.34628
H   4.38053 -4.31843 -0.90240
C   3.54393 -2.35176 -1.24486
C   4.86602 -2.22340 0.90233
H   3.91721 -2.25405 1.46106
H   5.58382 -1.61439 1.47516
H   5.26215 -3.25155 0.85428
C   4.66155 -1.62819 -0.50512
H   4.35663 -0.57389 -0.37965
C   5.98284 -1.66083 -1.30301
H   6.32709 -2.69929 -1.44610
H   6.77290 -1.11236 -0.76366
H   5.87662 -1.20843 -2.30274
C   0.18450 -1.55538 -2.98158
H   0.54961 -0.59339 -3.38660
C -0.76615 -1.26141 -1.79300
H -1.08363 -2.24028 -1.39331
H -1.68789 -0.83223 -2.21444
C -0.58747 -2.25594 -4.11463
H   0.07028 -2.53082 -4.95659
H -1.37287 -1.58124 -4.49193
H -1.09802 -3.16769 -3.76045
H   0.29002 1.32906 0.92875
H -1.05831 1.62545 -0.59346
H -1.18700 1.13789 -1.26955

TS(3^{+}-5^{+})1
SCF = -2960.45895960
H(0 K)= -2959.258854
G(298 K)= -2959.363797
SCF (C6H5F) = -2960.50383564
Lowest Frequencies = -674.6689cm-1, 20.3831cm-1
SCF [PBE0(C6H5F, D3)] = -6061.92172973

143
Ir -0.14320 0.13674 -0.18492
Zn 1.89393 0.08784 1.31974
Zn 0.85111 -2.16132 0.27508
N -2.81220 -1.21673 0.82085
N -1.52445 -0.71341 2.48574
N   1.61977 2.16600 -1.75157
N   2.41851 0.16373 -1.96183
C -1.58449 -0.64238 1.10059
C -3.48312 -1.61131 1.98177
H -4.46853 -2.06334 1.94082
C -2.67304 -1.29403 3.02938

H	-2.80244	-1.39829	4.10177
C	-3.45036	-1.24543	-0.47253
C	-3.51895	-2.47005	-1.17322
C	-4.13467	-2.44990	-2.43924
H	-4.20456	-3.37484	-3.01817
C	-4.63285	-1.25737	-2.97805
H	-5.09031	-1.25959	-3.97230
C	-4.56558	-0.06227	-2.24629
H	-4.97974	0.85557	-2.67173
C	-3.99119	-0.03088	-0.96275
C	-2.95436	-3.76290	-0.59011
H	-2.20301	-3.48650	0.17332
C	-4.07068	-4.57603	0.10536
H	-4.56496	-4.00359	0.90577
H	-3.65722	-5.49615	0.55108
H	-4.84599	-4.86636	-0.62401
C	-2.24975	-4.64741	-1.63738
H	-2.96067	-5.04964	-2.37866
H	-1.77529	-5.50798	-1.13855
H	-1.46634	-4.09536	-2.18149
C	-4.02909	1.22410	-0.09404
H	-3.13738	1.20430	0.55341
C	-5.27914	1.19373	0.81525
H	-6.20206	1.21422	0.21093
H	-5.28648	2.07276	1.48224
H	-5.30523	0.29076	1.44598
C	-3.96598	2.53994	-0.88191
H	-3.05631	2.59698	-1.49637
H	-3.94241	3.38863	-0.17893
H	-4.84455	2.68144	-1.53480
C	-0.56649	-0.04126	3.32665
C	-0.81451	1.31714	3.63345
C	0.11201	1.96634	4.47039
H	-0.04782	3.01747	4.73000
C	1.21964	1.28278	4.98938
H	1.92875	1.80638	5.63757
C	1.41386	-0.07377	4.69980
H	2.26931	-0.60108	5.13018
C	0.52201	-0.77126	3.86072
C	-2.07383	2.03397	3.15832
H	-2.62560	1.34709	2.49687
C	-2.99164	2.34371	4.36060
H	-2.50916	3.04519	5.06246
H	-3.24351	1.42780	4.92065
H	-3.93194	2.80487	4.01467
C	-1.77178	3.30056	2.33865
H	-2.71453	3.76349	2.00106
H	-1.17094	3.06663	1.44698
H	-1.22878	4.05388	2.93449
C	0.65705	-2.27226	3.63286
H	0.09630	-2.52097	2.71268
C	0.00051	-3.04848	4.79671
H	0.06547	-4.13504	4.61944
H	-1.06298	-2.78523	4.91340
H	0.50990	-2.82365	5.74929
C	2.11085	-2.73316	3.43512
H	2.60818	-2.17205	2.62445
H	2.13678	-3.80175	3.17031
H	2.71415	-2.59836	4.34840
C	3.51934	0.72777	2.18742

H	4.24735	1.11351	1.45429
H	4.00058	-0.06613	2.77838
H	3.23425	1.54386	2.87020
C	0.86965	-4.11259	0.43702
H	0.27069	-4.44458	1.30199
H	1.90605	-4.46370	0.56893
H	0.46468	-4.57883	-0.47399
C	1.37997	0.86574	-1.37192
C	2.75315	2.27326	-2.55907
H	3.07773	3.22788	-2.96110
C	3.25371	1.01138	-2.69861
H	4.10292	0.62913	-3.25535
C	0.71625	3.25895	-1.49582
C	-0.32944	3.47173	-2.42113
C	-1.22130	4.52421	-2.14470
H	-2.04411	4.72776	-2.83725
C	-1.06184	5.31858	-1.00132
H	-1.76612	6.13244	-0.80318
C	0.00305	5.09118	-0.11850
H	0.12233	5.73203	0.75951
C	0.92079	4.04929	-0.34572
C	-0.44967	2.65116	-3.70187
H	0.29219	1.83583	-3.65232
C	-1.83005	1.99134	-3.87519
H	-2.07074	1.32268	-3.03307
H	-1.84809	1.39172	-4.80098
H	-2.63645	2.74051	-3.95043
C	-0.09283	3.52515	-4.92405
H	-0.80751	4.35820	-5.03640
H	-0.11973	2.92663	-5.85015
H	0.91503	3.96097	-4.82385
C	2.13090	3.82554	0.55347
H	2.47464	2.78628	0.40148
C	1.82060	3.98289	2.04957
H	2.73305	3.80233	2.64103
H	1.05785	3.26052	2.37543
H	1.46679	4.99843	2.29624
C	3.28101	4.76533	0.12590
H	2.98977	5.82159	0.25714
H	3.55063	4.62230	-0.93354
H	4.18019	4.57759	0.73649
C	2.53825	-1.27170	-2.01915
C	1.51556	-1.98531	-2.69664
C	1.68739	-3.36924	-2.86873
H	0.91621	-3.94496	-3.38665
C	2.83711	-4.01612	-2.39587
H	2.95865	-5.09258	-2.54768
C	3.82023	-3.29123	-1.71546
H	4.70582	-3.80832	-1.33516
C	3.69647	-1.90283	-1.50642
C	5.10352	-1.83739	0.59366
H	4.19107	-1.99894	1.18971
H	5.79263	-1.21452	1.18620
H	5.58561	-2.81731	0.44048
C	4.78036	-1.14825	-0.74742
H	4.39570	-0.13822	-0.51987
C	6.06207	-1.00047	-1.59618
H	6.48619	-1.99007	-1.83740
H	6.82605	-0.42870	-1.04374
H	5.87350	-0.48219	-2.55069

```
C   0.24919 -1.28029 -3.14311
H   0.50913 -0.24448 -3.42638
C  -0.75739 -1.23093 -1.97659
H  -0.87142 -2.23010 -1.52876
H  -1.74904 -1.00798 -2.40087
C  -0.41998 -1.92705 -4.37273
H   0.29048 -2.04411 -5.20757
H  -1.25400 -1.29164 -4.71213
H  -0.84586 -2.91742 -4.13899
H   0.28015  1.13088  1.06036
H  -1.00643  1.50166 -0.36755
H  -1.17778  0.30764 -1.40617
```

Int(3⁺-5⁺)2

```
SCF  =           -2960.48410511
H(0 K)=          -2959.280193
G(298 K)=        -2959.387156
SCF (C6H5F) =    -2960.53083397
Lowest Frequencies = 19.4889cm-1,
25.4343cm-1
SCF [PBE0(C6H5F, D3)] =
-6061.93803248

143
Ir -0.14177  0.02939 -0.15039
Zn  1.77778  0.77784  1.25861
Zn  1.38884 -1.77779  0.84673
N  -2.44767 -1.62409  1.16361
N  -1.45814 -0.39272  2.63995
N   1.10541  2.02785 -2.18922
N   2.31270  0.23188 -2.05498
C  -1.42256 -0.70679  1.29056
C  -3.10503 -1.84881  2.37667
H  -3.95027 -2.52571  2.44754
C  -2.48432 -1.06915  3.30603
H  -2.67785 -0.90925  4.36184
C  -2.95629 -2.12664 -0.08689
C  -2.60619 -3.43401 -0.48834
C  -3.17397 -3.90813 -1.68733
H  -2.94051 -4.92007 -2.03099
C  -4.01814 -3.09388 -2.45403
H  -4.43788 -3.47672 -3.38955
C  -4.34799 -1.80079 -2.02201
H  -5.03412 -1.18917 -2.61566
C  -3.84303 -1.29350 -0.81081
C  -1.68352 -4.30376  0.36164
H  -1.10138 -3.63057  1.01777
C  -2.51783 -5.24163  1.26375
H  -3.20533 -4.67830  1.91471
H  -1.85753 -5.84640  1.90762
H  -3.12480 -5.92994  0.65099
C  -0.67510 -5.12339 -0.46544
H  -1.17814 -5.84314 -1.13311
H  -0.02559 -5.70145  0.21125
H  -0.02250 -4.47922 -1.07656
C  -4.34553  0.02364 -0.22657
H  -3.64858  0.32191  0.57283
C  -5.73147 -0.20978  0.41782
H  -6.47701 -0.49316 -0.34474
H  -6.08310  0.70947  0.91600
H  -5.69780 -1.01542  1.16982
C  -4.38740  1.19199 -1.22312
H  -3.37388  1.46050 -1.55683
H  -4.82258  2.07942 -0.73366
H  -5.00667  0.97015 -2.10906
C  -0.70751  0.66015  3.27352
C  -1.23784  1.96975  3.21030
C  -0.51450  2.98867  3.85936
H  -0.89686  4.01387  3.83463
C   0.66714  2.70372  4.55496
H   1.21258  3.50929  5.05579
C   1.14863  1.38971  4.62646
H   2.06503  1.18071  5.18435
C   0.47204  0.33387  3.98611
C  -2.57945  2.26624  2.54948
H  -2.95569  1.32682  2.11306
C  -3.60630  2.72433  3.60716
H  -3.30571  3.67929  4.07101
H  -3.71477  1.97912  4.41267
H  -4.59424  2.87326  3.14011
C  -2.46249  3.27936  1.39837
H  -3.45178  3.45386  0.94343
H  -1.79300  2.90984  0.60697
H  -2.08022  4.25349  1.74752
C   0.93620 -1.11113  4.12982
H   0.54503 -1.67305  3.25972
C   0.33374 -1.75111  5.40074
H   0.65970 -2.80065  5.49263
H  -0.76730 -1.73801  5.38343
H   0.66564 -1.20707  6.30143
C   2.46722 -1.25706  4.13765
H   2.92942 -0.74884  3.27428
H   2.74504 -2.32180  4.09626
H   2.91434 -0.83106  5.05150
C   3.11238  2.02708  1.94973
H   3.73297  2.43659  1.13471
H   3.76982  1.51961  2.67338
H   2.60159  2.85418  2.46494
C   1.95181 -3.56270  1.42834
H   1.25366 -3.97558  2.17705
H   2.95709 -3.51034  1.87917
H   1.99823 -4.24700  0.56715
C   1.15645  0.80898 -1.54644
C   2.17454  2.19721 -3.07034
H   2.28500  3.09893 -3.66431
C   2.93007  1.06267 -2.99516
H   3.82794  0.75663 -3.52246
C  -0.00747  2.94474 -2.12241
C  -1.09393  2.71954 -2.99995
C  -2.16812  3.62408 -2.93339
H  -3.02867  3.48656 -3.59483
C  -2.14122  4.70836 -2.04520
H  -2.98420  5.40551 -2.01327
C  -1.03611  4.91524 -1.21070
H  -1.02356  5.77448 -0.53448
C   0.05787  4.02886 -1.22376
C  -1.05283  1.61292 -4.04842
H  -0.27950  0.89099 -3.73787
C  -2.36760  0.82682 -4.18487
H  -2.65597  0.35551 -3.23315
```

```
H  -2.25011  0.03259 -4.94169
H  -3.20335  1.46788 -4.51308
C  -0.62273  2.20464 -5.40976
H  -1.36592  2.93693 -5.76868
H  -0.53169  1.40871 -6.16825
H   0.34814  2.72168 -5.33716
C   1.29360  4.27147 -0.36537
H   1.83723  3.31334 -0.27881
C   0.95810  4.73349  1.06154
H   1.88741  4.87841  1.63592
H   0.34614  3.98686  1.58964
H   0.41650  5.69470  1.06898
C   2.23133  5.28238 -1.06316
H   1.73302  6.26034 -1.17725
H   2.52600  4.93849 -2.06826
H   3.14926  5.43129 -0.46993
C   2.72425 -1.13884 -1.86241
C   1.97435 -2.14560 -2.52196
C   2.42313 -3.47229 -2.41958
H   1.86708 -4.26992 -2.91968
C   3.58880 -3.77984 -1.70301
H   3.93254 -4.81656 -1.64059
C   4.31071 -2.76735 -1.06235
H   5.21230 -3.02215 -0.49866
C   3.89586 -1.42167 -1.12338
C   5.07959 -0.72651  1.00896
H   4.19736 -1.05638  1.58251
H   5.52878  0.13143  1.53367
H   5.81371 -1.54934  1.02405
C   4.69724 -0.32886 -0.42932
H   4.05659  0.56998 -0.37038
C   5.95824  0.03741 -1.24211
H   6.62521 -0.83603 -1.34097
H   6.52000  0.84146 -0.73828
H   5.70758  0.38320 -2.25794
C   0.68976 -1.77704 -3.24473
H   0.79212 -0.73591 -3.59366
C  -0.49181 -1.84328 -2.26016
H  -0.86833 -2.87057 -2.16156
H  -1.33168 -1.19573 -2.53556
C   0.38029 -2.64186 -4.47883
H   1.20918 -2.61401 -5.20413
H  -0.52842 -2.26771 -4.97861
H   0.19256 -3.69450 -4.20781
H  -0.26753  1.39607  0.61433
H  -1.39405  0.70230 -0.93707
H  -0.14235 -1.65178 -1.18811
```

TS(3⁺-5⁺)2

```
SCF  =           -2960.47254520
H(0 K)=          -2959.270037
G(298 K)=        -2959.377600
SCF (C6H5F) =    -2960.52109086
Lowest Frequencies = -301.4356cm-1,  21.9005cm-1
SCF [PBE0(C6H5F, D3)] = -6061.93798275

143
Ir -0.07153 -0.11195 -0.05571
Zn -0.02291  2.47751 -0.25444
Zn  1.43121  1.10755  1.45252
N  -1.00279 -1.56416  2.53070
N  -1.92259  0.39291  2.39775
N   0.43064 -0.01390 -3.11520
N   2.25099  0.50869 -2.06729
C  -1.06402 -0.45542  1.70873
C  -1.79565 -1.41566  3.66892
H  -1.87094 -2.20055  4.41470
C  -2.38146 -0.18584  3.58200
H  -3.09458  0.32081  4.22453
C  -0.37413 -2.81796  2.19343
C   0.89989 -3.10635  2.72486
C   1.44545 -4.36998  2.42758
H   2.42472 -4.64201  2.83175
C   0.75448 -5.27947  1.61808
H   1.20064 -6.25288  1.39165
C  -0.51364 -4.96088  1.11138
H  -1.05241 -5.69199  0.50140
C  -1.11834 -3.72571  1.40259
C   1.61806 -2.12737  3.64724
H   1.17106 -1.12850  3.49149
C   1.38610 -2.52369  5.12293
H   0.31291 -2.56368  5.37111
H   1.86662 -1.79710  5.79964
H   1.81364 -3.51978  5.32956
C   3.12246 -2.00692  3.34913
H   3.64598 -2.96972  3.47608
H   3.58565 -1.28963  4.04599
H   3.30097 -1.64787  2.32396
C  -2.55795 -3.43001  0.99384
H  -2.72135 -2.34510  1.10008
C  -3.52252 -4.15117  1.96283
H  -3.41290 -5.24624  1.88292
H  -4.56914 -3.89385  1.72722
H  -3.32874 -3.87110  3.01156
C  -2.87692 -3.77949 -0.46814
H  -2.29332 -3.15341 -1.15850
H  -3.94646 -3.59906 -0.66849
H  -2.67695 -4.83960 -0.69875
C  -2.48131  1.61043  1.86585
C  -3.60631  1.49380  1.01705
C  -4.13443  2.68506  0.48128
H  -5.00096  2.63352 -0.18453
C  -3.57816  3.92892  0.80156
H  -4.00266  4.84246  0.37442
C  -2.48861  4.01290  1.67958
H  -2.07519  4.99238  1.93291
C  -1.91482  2.85434  2.23588
C  -4.24936  0.14640  0.70897
H  -3.80682 -0.60282  1.38736
C  -5.76661  0.16750  0.98612
H  -6.29845  0.84356  0.29534
H  -5.98602  0.49447  2.01574
H  -6.18754 -0.84212  0.84730
C  -3.95238 -0.30103 -0.73356
H  -4.41901 -1.27746 -0.94142
H  -2.86711 -0.40191 -0.91157
H  -4.35151  0.42261 -1.46229
C  -0.78706  2.93913  3.25687
```

```
H  -0.22957  1.98397  3.21470
C  -1.36096  3.07663  4.68540
H  -0.54397  3.11893  5.42501
H  -2.01330  2.22925  4.94816
H  -1.95522  4.00185  4.77512
C   0.20374  4.08055  2.97280
H   0.58158  4.04565  1.93670
H   1.06588  4.01083  3.65452
H  -0.25806  5.07050  3.12458
C  -0.31048  4.25520 -1.01564
H   0.14780  4.34802 -2.01486
H   0.11429  5.03240 -0.36015
H  -1.39414  4.43112 -1.10056
C   2.59976  1.43458  2.98523
H   2.05706  1.21759  3.91990
H   2.90943  2.49130  2.97770
H   3.49120  0.79707  2.91388
C   0.93662  0.13204 -1.84402
C   1.39171  0.24775 -4.09369
H   1.16197  0.17277 -5.15196
C   2.54370  0.56595 -3.43368
H   3.54048  0.80115 -3.79301
C  -0.87736 -0.54629 -3.40772
C  -1.00197 -1.95102 -3.51745
C  -2.27780 -2.45519 -3.82753
H  -2.41861 -3.53605 -3.92347
C  -3.36090 -1.59173 -4.04080
H  -4.34277 -2.00420 -4.29286
C  -3.19208 -0.20397 -3.95268
H  -4.04273  0.45659 -4.14332
C  -1.94288  0.35268 -3.62163
C   0.21025 -2.87191 -3.42589
H   1.03960 -2.29132 -2.98984
C  -0.00145 -4.09262 -2.51503
H  -0.18366 -3.78349 -1.47471
H   0.90052 -4.72794 -2.53023
H  -0.84740 -4.71795 -2.84735
C   0.63654 -3.30377 -4.84714
H  -0.15406 -3.90390 -5.32915
H   1.55336 -3.91586 -4.80661
H   0.83385 -2.43200 -5.49302
C  -1.72351  1.86045 -3.58185
H  -0.80114  2.05191 -3.00288
C  -2.86463  2.61810 -2.88505
H  -2.65024  3.69880 -2.88009
H  -2.98308  2.29233 -1.84009
H  -3.82945  2.47813 -3.40168
C  -1.49533  2.39944 -5.01183
H  -2.38797  2.23053 -5.63822
H  -0.64322  1.90055 -5.50216
H  -1.29112  3.48318 -4.98922
C   3.27161  0.55005 -1.04892
C   3.83101 -0.68465 -0.63931
C   4.84678 -0.64387  0.33467
H   5.29771 -1.58078  0.67549
C   5.30741  0.57586  0.84450
H   6.10487  0.58790  1.59372
C   4.76152  1.78354  0.39059
H   5.13984  2.72868  0.78839
C   3.72863  1.80109 -0.56551
C   3.04161  4.19500 -0.01885
H   2.46196  3.82733  0.84477
H   2.52547  5.07780 -0.42689
H   4.02360  4.52930  0.35542
C   3.18419  3.11644 -1.10708
H   2.17826  2.91645 -1.52382
C   4.06824  3.63627 -2.26290
H   5.09466  3.82985 -1.90736
H   3.65970  4.57837 -2.66486
H   4.12933  2.91197 -3.09039
C   3.40339 -2.00944 -1.25595
H   2.69688 -1.79194 -2.07339
C   2.66490 -2.88719 -0.23442
H   3.31838 -3.15777  0.61037
H   2.31320 -3.82128 -0.70024
C   4.60310 -2.75306 -1.87821
H   5.12524 -2.12732 -2.62055
H   4.25645 -3.67036 -2.38262
H   5.33772 -3.05300 -1.11179
H  -1.22335  0.93850 -0.24989
H  -0.36959 -1.68791 -0.45139
H   1.77901 -2.36764  0.17226
```

TS(1,3-1,4)-4$^+$

SCF =   -2961.66285179
H(0 K)=   -2960.448024
G(298 K)=   -2960.562095
SCF (C6H5F) =   -2961.71334832
Lowest Frequencies = -106.0199cm-1, 19.7408cm-1
SCF [PBE0(C6H5F, D3)] = -6063.13840474

```
145
Ir  0.05142 -0.07329 -0.11414
Zn -1.68040 -0.59341 -2.09856
Zn  0.30983 -1.40416  2.02607
N  -2.94346  0.01464  1.07307
N  -1.72260  1.67209  1.78264
N   2.77112  0.38608 -1.62948
N   2.69047 -1.65359 -0.88703
C  -1.68465  0.59646  0.89703
C  -3.68867  0.68111  2.05057
H  -4.69170  0.36234  2.31731
C  -2.92754  1.71973  2.48704
H  -3.12417  2.49737  3.21878
C  -3.62218 -0.93625  0.20776
C  -3.52974 -2.32903  0.47941
C  -4.25998 -3.20228 -0.34982
H  -4.20158 -4.28040 -0.17335
C  -5.10141 -2.71406 -1.35886
H  -5.67181 -3.41287 -1.97921
C  -5.24695 -1.33563 -1.54510
H  -5.94612 -0.96391 -2.30063
C  -4.52147 -0.40860 -0.76782
C  -2.82006 -2.86207  1.72107
H  -2.09875 -2.09162  2.05011
C  -3.84828 -3.04300  2.86669
H  -4.37614 -2.10321  3.09904
H  -3.34131 -3.38425  3.78530
```

H	-4.60706	-3.79733	2.59585
C	-2.04096	-4.16948	1.48839
H	-2.71118	-5.00908	1.23572
H	-1.50355	-4.45173	2.40899
H	-1.29793	-4.06617	0.68136
C	-4.83636	1.08669	-0.90428
H	-4.12719	1.65158	-0.27600
C	-4.70189	1.62831	-2.34317
H	-3.67077	1.53758	-2.72079
H	-4.97995	2.69554	-2.36632
H	-5.36562	1.10048	-3.04897
C	-6.26155	1.36877	-0.36474
H	-7.02562	0.86368	-0.98020
H	-6.47090	2.45146	-0.38861
H	-6.38806	1.02000	0.67345
C	-0.79827	2.78804	1.93636
C	-0.88886	3.85802	1.00655
C	-0.11504	5.00599	1.26247
H	-0.16472	5.84772	0.56537
C	0.68539	5.10494	2.40642
H	1.26146	6.01654	2.59511
C	0.73788	4.04330	3.31567
H	1.35499	4.13473	4.21489
C	0.00226	2.85929	3.10430
C	-1.86202	3.85008	-0.17258
H	-2.13982	2.80112	-0.37472
C	-3.14936	4.62813	0.19646
H	-3.64811	4.20317	1.08383
H	-3.86748	4.60804	-0.64179
H	-2.91802	5.68494	0.41569
C	-1.24563	4.40907	-1.47022
H	-1.02353	5.48745	-1.39186
H	-1.95889	4.28959	-2.30395
H	-0.31246	3.88601	-1.73256
C	0.05858	1.74743	4.15244
H	-0.42863	0.84973	3.72808
C	-0.71634	2.14563	5.43162
H	-0.26884	3.03874	5.90094
H	-0.68979	1.32611	6.16988
H	-1.77377	2.37600	5.22041
C	1.51190	1.36359	4.50589
H	2.07673	1.05436	3.61067
H	1.52083	0.53051	5.22847
H	2.05389	2.20330	4.97292
C	-2.63118	-0.98623	-3.75934
H	-1.89975	-1.40237	-4.47409
H	-3.08513	-0.08976	-4.20789
H	-3.41982	-1.73304	-3.58164
C	0.30761	-2.13081	3.83640
H	-0.71739	-2.12772	4.24509
H	0.95293	-1.56157	4.52259
H	0.66754	-3.17265	3.80238
C	1.98089	-0.45930	-0.86679
C	3.90798	-0.26725	-2.10897
H	4.63930	0.24749	-2.72462
C	3.85731	-1.54611	-1.64600
H	4.53218	-2.38667	-1.77493
C	2.62255	1.80811	-1.89997
C	3.30307	2.71496	-1.04893
C	3.26706	4.07929	-1.39880

H	3.78300	4.80668	-0.76426
C	2.60896	4.51497	-2.55437
H	2.60631	5.57840	-2.81464
C	1.96805	3.59016	-3.38874
H	1.47418	3.94011	-4.30040
C	1.96163	2.21454	-3.08578
C	4.12619	2.26045	0.15725
H	3.87820	1.20348	0.36068
C	3.79691	3.05884	1.43432
H	4.07892	4.12152	1.33730
H	4.36238	2.65072	2.28977
H	2.72299	3.01443	1.67239
C	5.63974	2.34029	-0.15869
H	5.91224	1.72707	-1.03387
H	6.23299	1.98680	0.70204
H	5.94246	3.37996	-0.37235
C	1.32151	1.21840	-4.05238
H	1.18855	0.26416	-3.51128
C	-0.07124	1.67593	-4.53447
H	-0.73594	1.90503	-3.68408
H	-0.54328	0.88579	-5.14179
H	-0.01181	2.57893	-5.16599
C	2.25124	0.95225	-5.26107
H	2.43067	1.88092	-5.82993
H	1.79500	0.21756	-5.94672
H	3.23163	0.55832	-4.94525
C	2.40743	-2.88495	-0.17257
C	3.06596	-3.10119	1.06865
C	2.81520	-4.32489	1.72491
H	3.30402	-4.52691	2.68299
C	1.97818	-5.29387	1.15924
H	1.80427	-6.23860	1.68438
C	1.39072	-5.07243	-0.09393
H	0.77447	-5.85628	-0.54498
C	1.59712	-3.86875	-0.79521
C	4.11324	-2.13391	1.63105
H	4.00966	-1.16975	1.10182
C	3.95379	-1.85219	3.13915
H	2.95769	-1.44749	3.37817
H	4.70998	-1.11772	3.46379
H	4.10243	-2.76102	3.74706
C	5.53607	-2.67860	1.34682
H	5.69702	-3.63944	1.86542
H	6.29863	-1.96723	1.70672
H	5.70996	-2.84759	0.27154
C	1.05148	-3.69556	-2.21334
H	1.09211	-2.61988	-2.46062
C	-0.41453	-4.14989	-2.36403
H	-0.52911	-5.23649	-2.20805
H	-0.77453	-3.93129	-3.38355
H	-1.07636	-3.63324	-1.64850
C	1.95573	-4.44352	-3.22433
H	2.99898	-4.08886	-3.18105
H	1.58838	-4.29437	-4.25408
H	1.96140	-5.52802	-3.01933
H	-0.18700	-1.72573	0.34354
H	-0.08576	-0.84479	-1.61420
H	0.16733	1.37956	-0.83075
H	0.78782	0.76280	1.07943

4^{+}, 1,4-isomer

```
SCF  =          -2961.70137174
H(0 K)=         -2960.480651
G(298 K)=       -2960.590363
SCF (C6H5F) =   -2961.74383270
Lowest Frequencies = 8.8656cm-1,
15.6040cm-1
SCF [PBE0(C6H5F, D3)] =
-6063.16986967

145
Ir -0.00060  0.07418  0.03091
Zn -2.20311 -0.40260  1.13048
Zn  2.25623  0.16984 -1.06172
N  -1.40568  2.86330  0.21991
N   0.72757  3.11236  0.03281
N  -0.28159 -2.89137 -0.93404
N   0.85677 -2.80179  0.91111
C  -0.24106  2.12926  0.09252
C  -1.16327  4.24007  0.23364
H  -1.97303  4.95680  0.32524
C   0.18411  4.39730  0.12203
H   0.81254  5.28148  0.08254
C  -2.74806  2.35264  0.30192
C  -3.41600  2.42477  1.54806
C  -4.76961  2.03126  1.57397
H  -5.32329  2.07894  2.51625
C  -5.40644  1.57350  0.41589
H  -6.45586  1.26612  0.45865
C  -4.71185  1.50672 -0.80279
H  -5.22877  1.15896 -1.70140
C  -3.36957  1.91412 -0.89513
C  -2.73611  2.96934  2.80103
H  -1.66886  3.12674  2.56807
C  -3.34441  4.33495  3.18950
H  -3.27690  5.06267  2.36387
H  -2.81860  4.75683  4.06202
H  -4.41064  4.23100  3.45348
C  -2.80294  1.98265  3.98131
H  -3.84365  1.76071  4.27264
H  -2.29322  2.40838  4.86156
H  -2.31276  1.02971  3.72937
C  -2.63213  1.97161 -2.22820
H  -1.55298  1.89927 -2.01495
C  -2.97395  0.79741 -3.15519
H  -2.78444 -0.16562 -2.65845
H  -2.34302  0.83442 -4.05804
H  -4.02536  0.81631 -3.49036
C  -2.89215  3.33419 -2.90943
H  -3.96410  3.46368 -3.13686
H  -2.33141  3.39984 -3.85725
H  -2.57739  4.17440 -2.26795
C   2.13206  2.93632 -0.22273
C   2.59793  3.20114 -1.53397
C   3.99212  3.16943 -1.73613
H   4.39408  3.37504 -2.73270
C   4.86397  2.86876 -0.68343
H   5.94326  2.84713 -0.86288
C   4.36678  2.58544  0.59836
H   5.06199  2.35300  1.40991
C   2.98635  2.62996  0.86472
C   1.64627  3.54021 -2.67849
H   0.61766  3.54341 -2.28000
C   1.93403  4.94968 -3.23593
H   1.87421  5.71532 -2.44465
H   1.20340  5.20825 -4.02010
H   2.94100  5.00800 -3.68273
C   1.69132  2.47429 -3.79060
H   2.70128  2.37674 -4.22361
H   0.99758  2.74270 -4.60492
H   1.39168  1.48781 -3.39979
C   2.42444  2.44172  2.26788
H   1.36503  2.15315  2.16294
C   2.48126  3.78070  3.03707
H   3.52356  4.12346  3.15438
H   2.04469  3.66347  4.04324
H   1.92155  4.57283  2.51185
C   3.11886  1.31160  3.04081
H   3.06975  0.36391  2.48389
H   2.61714  1.15079  4.00874
H   4.17825  1.53655  3.25346
C  -3.74235 -1.08082  2.09910
H  -3.67353 -2.17400  2.22602
H  -4.66179 -0.83705  1.54996
H  -3.79750 -0.60961  3.09117
C   3.87429 -0.08836 -2.10119
H   4.04832 -1.16062 -2.28792
H   4.73921  0.32943 -1.56900
H   3.77270  0.42973 -3.06641
C   0.22062 -1.99026 -0.01131
C   0.02387 -4.20953 -0.58257
H  -0.28662 -5.04531 -1.20087
C   0.73481 -4.15355  0.57752
H   1.16672 -4.93044  1.19988
C  -1.00947 -2.62653 -2.15952
C  -0.27341 -2.36473 -3.34058
C  -0.99718 -2.25298 -4.54199
H  -0.46327 -2.04392 -5.47175
C  -2.38452 -2.43438 -4.57346
H  -2.92316 -2.36392 -5.52335
C  -3.08162 -2.70508 -3.39326
H  -4.16522 -2.84273 -3.42789
C  -2.41338 -2.79592 -2.15658
C   1.24846 -2.27976 -3.34462
H   1.55895 -1.82180 -2.38684
C   1.79155 -1.40183 -4.48415
H   1.63225 -1.87357 -5.46851
H   2.87592 -1.26093 -4.36616
H   1.31277 -0.40947 -4.49870
C   1.89175 -3.68373 -3.41180
H   1.60618 -4.31233 -2.55449
H   2.99161 -3.59622 -3.41344
H   1.58871 -4.20361 -4.33662
C  -3.20178 -3.10641 -0.88946
H  -2.59823 -2.77280 -0.02317
C  -4.54522 -2.35435 -0.84887
H  -4.40615 -1.26632 -0.95772
H  -5.05247 -2.54251  0.10931
H  -5.22414 -2.69585 -1.64765
C  -3.44222 -4.62494 -0.73605
```

H -4.01564 -5.01318 -1.59514
H -4.01866 -4.83085 0.18152
H -2.49863 -5.18887 -0.67571
C 1.59378 -2.41940 2.09872
C 3.00260 -2.31867 2.01206
C 3.71139 -2.12021 3.21235
H 4.80106 -2.04556 3.18500
C 3.04615 -2.00921 4.43743
H 3.61821 -1.85535 5.35749
C 1.65086 -2.09307 4.48752
H 1.14076 -2.00717 5.45028
C 0.89378 -2.31512 3.32212
C 3.75392 -2.45152 0.69146
H 3.06493 -2.14740 -0.12111
C 4.99072 -1.53577 0.63243
H 4.73252 -0.48447 0.84174
H 5.44717 -1.58479 -0.36739
H 5.76109 -1.85167 1.35533
C 4.17137 -3.91482 0.42185
H 4.83167 -4.28138 1.22633
H 4.72125 -3.98531 -0.53173
H 3.30242 -4.58742 0.35981
C -0.61375 -2.50924 3.40855
H -1.04402 -2.24111 2.42585
C -1.25757 -1.59947 4.46769
H -0.96383 -1.89170 5.48970
H -2.35421 -1.67085 4.41330
H -0.96570 -0.54677 4.31880
C -0.96829 -3.98874 3.67616
H -0.58767 -4.65001 2.88162
H -2.06288 -4.11636 3.72839
H -0.53754 -4.32395 4.63507
H -0.36984 0.02873 1.67503
H -1.53112 -0.11379 -0.63179
H 0.36059 0.18382 -1.60673
H 1.53901 0.21621 0.69378

TS(1,3-1,2)-4$^+$

SCF = -2961.69192502
H(0 K)= -2960.471754
G(298 K)= -2960.579897
SCF (C6H5F) = -2961.73923640
Lowest Frequencies = -153.5302cm-1, 18.7333cm-1
SCF [PBE0(C6H5F, D3)] = -6063.1561507

145
Ir 0.09643 0.02520 -0.00360
Zn -0.02652 -2.54989 -0.30389
Zn -1.52197 -0.23421 -1.95997
N 2.29851 0.44705 -2.17604
N 1.30333 2.31693 -1.72824
N -0.71915 -1.17056 2.76874
N -2.45281 -1.11875 1.46293
C 1.31416 0.98933 -1.36629
C 2.88027 1.41781 -2.99664
H 3.68969 1.17247 -3.67709
C 2.25419 2.59558 -2.71380
H 2.39347 3.60122 -3.09725
C 2.85711 -0.87693 -2.04662
C 2.27411 -1.94580 -2.76953
C 2.82912 -3.22526 -2.58592
H 2.40066 -4.07978 -3.11567
C 3.93570 -3.41702 -1.74448
H 4.35661 -4.41962 -1.62182
C 4.50620 -2.33352 -1.06899
H 5.37177 -2.49325 -0.41880
C 3.97289 -1.03551 -1.19601
C 1.11637 -1.70102 -3.72738
H 0.51389 -0.87299 -3.30564
C 1.63282 -1.23193 -5.10567
H 2.22778 -0.30862 -5.02339
H 0.78594 -1.03124 -5.78315
H 2.26709 -2.00772 -5.56708
C 0.19069 -2.91711 -3.88446
H 0.69012 -3.74852 -4.40978
H -0.69695 -2.64028 -4.47374
H -0.15283 -3.30061 -2.90707
C 4.60114 0.13138 -0.44277
H 4.05230 1.04907 -0.71235
C 4.47061 -0.04296 1.08253
H 3.41429 -0.11217 1.38651
H 4.92069 0.81853 1.60377
H 4.98836 -0.95335 1.42986
C 6.07015 0.33482 -0.86807
H 6.69224 -0.53624 -0.60042
H 6.49505 1.21739 -0.36153
H 6.15718 0.48724 -1.95675
C 0.59305 3.36363 -1.03841
C 1.19690 3.89954 0.12167
C 0.52461 4.95877 0.75839
H 0.95696 5.40348 1.65998
C -0.67057 5.46975 0.23469
H -1.16889 6.30659 0.73376
C -1.22884 4.92381 -0.92920
H -2.15853 5.33976 -1.32832
C -0.61540 3.84033 -1.58560
C 2.56452 3.42626 0.60355
H 2.80692 2.49328 0.06903
C 3.63928 4.47170 0.23275
H 3.64503 4.67769 -0.85067
H 4.64179 4.11103 0.51909
H 3.45868 5.42690 0.75490
C 2.59931 3.08757 2.10200
H 2.32246 3.95133 2.73028
H 3.61726 2.77949 2.39324
H 1.91908 2.25488 2.33083
C -1.19444 3.24554 -2.86450
H -0.73587 2.24767 -3.00389
C -0.80842 4.10979 -4.08516
H -1.23034 5.12500 -3.99060
H -1.19627 3.66204 -5.01557
H 0.28483 4.20930 -4.18381
C -2.71865 3.04697 -2.79759
H -3.00662 2.39578 -1.95647
H -3.07778 2.57659 -3.72712
H -3.25312 4.00533 -2.68446
C -0.22775 -4.46963 -0.10262
H -0.80920 -4.71881 0.80020

```
H   0.77614 -4.91469 -0.01025
H  -0.72578 -4.91216 -0.97824
C  -2.40984 -0.42286 -3.68128
H  -1.77933 -0.02883 -4.49646
H  -3.35222  0.14686 -3.65249
H  -2.65289 -1.47438 -3.89893
C  -1.10493 -0.78476  1.49882
C  -1.77641 -1.73671  3.48348
H  -1.64686 -2.08638  4.50302
C  -2.86737 -1.70230  2.66478
H  -3.89411 -2.02036  2.81507
C   0.51455 -0.77707  3.41126
C   0.52003  0.46252  4.09124
C   1.72683  0.85230  4.70075
H   1.77206  1.80745  5.23325
C   2.86284  0.03511  4.64153
H   3.79169  0.35785  5.12165
C   2.80956 -1.20262  3.98796
H   3.69563 -1.84410  3.96805
C   1.62955 -1.63711  3.35718
C  -0.73267  1.32140  4.23390
H  -1.54446  0.84017  3.66557
C  -0.55883  2.73852  3.65755
H   0.25444  3.28653  4.16224
H  -1.48703  3.31776  3.79990
H  -0.33376  2.71005  2.57940
C  -1.18025  1.36837  5.71087
H  -1.34102  0.35468  6.11422
H  -2.12362  1.93158  5.80902
H  -0.42356  1.86368  6.34277
C   1.55299 -3.00374  2.69445
H   0.59658 -3.05324  2.14156
C   2.68723 -3.22107  1.68010
H   2.67399 -2.44835  0.89613
H   2.59096 -4.20596  1.19505
H   3.67824 -3.18788  2.16336
C   1.51906 -4.12187  3.75769
H   2.45117 -4.13105  4.34827
H   1.40926 -5.10850  3.27691
H   0.67954 -3.98406  4.45928
C  -3.40221 -0.71032  0.45120
C  -3.84645  0.63807  0.47741
C  -4.80437  1.01689 -0.47894
H  -5.16577  2.04818 -0.49221
C  -5.31504  0.09317 -1.40278
H  -6.06437  0.41144 -2.13386
C  -4.87560 -1.23292 -1.38767
H  -5.28675 -1.94782 -2.10535
C  -3.90404 -1.66671 -0.46151
C  -3.35727  1.61725  1.53810
H  -2.31936  1.34142  1.78749
C  -3.31827  3.07181  1.05301
H  -2.71695  3.17123  0.13881
H  -2.85052  3.70689  1.82196
H  -4.32652  3.47815  0.86170
C  -4.21634  1.49479  2.81766
H  -5.26642  1.75993  2.60658
H  -3.84064  2.18296  3.59385
H  -4.19828  0.47506  3.23397
C  -3.49149 -3.13132 -0.41127
H  -2.56974 -3.20376  0.19372
C  -3.18483 -3.70729 -1.80533
H  -4.07123 -3.68813 -2.46071
H  -2.86311 -4.75740 -1.72009
H  -2.38513 -3.14105 -2.30991
C  -4.58006 -3.96982  0.29526
H  -4.78906 -3.59709  1.31124
H  -4.26343 -5.02301  0.37562
H  -5.52575 -3.93783 -0.27234
H   0.95821 -1.22417 -0.73922
H   1.36387 -0.16762  0.99062
H   0.06944  1.40147  0.84340
H  -1.24970  0.87960 -0.56898
```

4⁺, 1,2-isomer

```
SCF  =            -2961.69653868
H(0 K)=           -2960.476378
G(298 K)=         -2960.585717
SCF (C6H5F) =     -2961.74297706
Lowest Frequencies = 13.1299cm-1,
25.0043cm-1
SCF [PBE0(C6H5F, D3)] =
-6063.15986777

145
Ir -0.01373  0.14556  0.01196
Zn -0.76240 -1.90959 -1.32546
Zn  0.93032 -2.03430  0.98053
N  -2.39060 -0.70992  1.87142
N  -1.02159  0.60098  2.91584
N   0.95778  1.12278 -2.78156
N   2.39662 -0.26375 -1.94941
C  -1.21669  0.00122  1.69113
C  -2.90926 -0.52906  3.15737
H  -3.84622 -0.98516  3.46034
C  -2.04344  0.29303  3.81650
H  -2.05383  0.69920  4.82291
C  -3.14718 -1.35873  0.82956
C  -2.98989 -2.75036  0.62232
C  -3.75711 -3.33794 -0.40276
H  -3.65889 -4.40804 -0.60089
C  -4.64346 -2.57150 -1.17117
H  -5.22697 -3.04884 -1.96420
C  -4.79616 -1.20269 -0.91902
H  -5.50885 -0.61771 -1.50852
C  -4.05279 -0.56351  0.09146
C  -2.10310 -3.59047  1.53367
H  -1.29131 -2.93503  1.90405
C  -2.90132 -4.07435  2.76573
H  -3.30821 -3.23303  3.34783
H  -2.25293 -4.66662  3.43281
H  -3.74596 -4.71090  2.45156
C  -1.45569 -4.78873  0.82086
H  -2.20627 -5.53843  0.51918
H  -0.74543 -5.28821  1.49741
H  -0.90806 -4.47999 -0.08483
C  -4.27691  0.91071  0.40510
H  -3.54355  1.20996  1.17158
C  -4.03877  1.81325 -0.81682
H  -3.01361  1.70097 -1.19899
```

H -4.18094 2.87154 -0.54146
H -4.73990 1.58627 -1.63829
C -5.68361 1.12161 1.00465
H -6.47081 0.85386 0.27925
H -5.82605 2.17916 1.28331
H -5.83427 0.50482 1.90631
C -0.02752 1.60649 3.19832
C -0.34270 2.94404 2.86552
C 0.62130 3.92162 3.17157
H 0.41838 4.96924 2.92909
C 1.82201 3.57343 3.80499
H 2.55349 4.35030 4.04797
C 2.09009 2.23889 4.13787
H 3.03024 1.98499 4.63560
C 1.17345 1.21739 3.82566
C -1.70403 3.32580 2.29437
H -2.20773 2.39760 1.97837
C -2.56963 3.97188 3.39872
H -2.66415 3.31016 4.27583
H -3.58287 4.18426 3.01784
H -2.12685 4.92243 3.74213
C -1.62263 4.22413 1.04928
H -1.10038 5.17375 1.25623
H -2.63949 4.47528 0.70366
H -1.10306 3.71425 0.22475
C 1.43653 -0.23778 4.19531
H 0.76594 -0.86494 3.57795
C 1.07465 -0.48911 5.67586
H 1.70784 0.12369 6.34008
H 1.22785 -1.54960 5.93758
H 0.02304 -0.23339 5.88576
C 2.87929 -0.68065 3.89898
H 3.13072 -0.55152 2.83458
H 3.00366 -1.74637 4.14998
H 3.61422 -0.11391 4.49530
C -1.21053 -3.19311 -2.72329
H -0.57634 -3.05509 -3.61521
H -2.26327 -3.03034 -3.00499
H -1.10490 -4.23163 -2.37392
C 1.51686 -3.50207 2.12136
H 2.55804 -3.30409 2.42141
H 1.48993 -4.46511 1.58859
H 0.89153 -3.58152 3.02670
C 1.18839 0.34975 -1.66444
C 1.98745 1.00743 -3.71789
H 1.97367 1.56706 -4.64783
C 2.89770 0.13902 -3.19112
H 3.85250 -0.21994 -3.56126
C -0.07937 2.11835 -2.89445
C 0.21550 3.41970 -2.42944
C -0.80805 4.38044 -2.53158
H -0.62378 5.39928 -2.17655
C -2.04696 4.05392 -3.09748
H -2.82767 4.81689 -3.17461
C -2.28907 2.76085 -3.58208
H -3.25582 2.52739 -4.03633
C -1.30918 1.75666 -3.48305
C 1.59883 3.80320 -1.91216
H 2.22693 2.89706 -1.91677
C 1.58065 4.32822 -0.46498
H 0.95386 5.23054 -0.36740
H 2.60347 4.59768 -0.15138
H 1.19856 3.56770 0.23388
C 2.25951 4.81927 -2.86899
H 2.30605 4.42940 -3.89948
H 3.28735 5.04577 -2.53910
H 1.69650 5.76776 -2.89551
C -1.52554 0.35817 -4.04795
H -0.82856 -0.32337 -3.52439
C -2.94733 -0.17691 -3.81966
H -3.20093 -0.20070 -2.74882
H -3.02896 -1.20380 -4.21073
H -3.70581 0.43236 -4.33987
C -1.15699 0.32702 -5.54805
H -1.81332 1.00249 -6.12296
H -1.27227 -0.69250 -5.95300
H -0.11544 0.64623 -5.71671
C 3.19775 -1.01416 -1.01409
C 3.98674 -0.27727 -0.09894
C 4.79961 -1.01067 0.78458
H 5.42876 -0.47547 1.50171
C 4.82510 -2.41150 0.74883
H 5.46513 -2.96267 1.44444
C 4.04087 -3.11042 -0.17658
H 4.07352 -4.20269 -0.19547
C 3.20499 -2.42723 -1.08293
C 4.01756 1.24612 -0.11748
H 3.13340 1.59273 -0.67638
C 3.91436 1.86378 1.28430
H 2.98459 1.55480 1.78253
H 3.90166 2.96354 1.21192
H 4.76490 1.58600 1.93016
C 5.27332 1.74156 -0.86743
H 6.19382 1.42896 -0.34527
H 5.27256 2.84301 -0.92820
H 5.31546 1.34245 -1.89441
C 2.41181 -3.17755 -2.14602
H 1.57004 -2.52885 -2.45457
C 1.82244 -4.50349 -1.63579
H 2.61102 -5.23654 -1.39705
H 1.17983 -4.95135 -2.40949
H 1.21528 -4.35775 -0.72734
C 3.28706 -3.42989 -3.39456
H 3.66601 -2.49088 -3.82758
H 2.70494 -3.95223 -4.17200
H 4.15670 -4.05764 -3.13554
H 1.36218 -0.21810 0.88438
H -1.37329 -0.19561 -0.89553
H -0.80722 1.48828 -0.41686
H 0.68166 1.46079 0.64560

TS(1,4-1,4)-4^{+}
SCF = -2961.66204455
H(0 K)= -2960.442208
G(298 K)= -2960.551729
SCF (C6H5F) = -2961.70655960
Lowest Frequencies = -630.6879cm-1, 8.4937cm-1
SCF [PBE0(C6H5F, D3)] = -6063.12448953

```
145
Ir -0.05764  0.05462 -0.05235
Zn -2.33087  0.04442  1.22648
Zn  2.03276 -0.30940 -1.31975
N  -0.92486  3.04984 -0.18715
N   1.22551  2.93161 -0.31098
N  -0.57801 -2.92108 -0.83803
N   0.57095 -2.83204  1.00228
C   0.10993  2.12878 -0.18925
C  -0.46063  4.36323 -0.30434
H  -1.14322  5.20712 -0.31137
C   0.89698  4.28791 -0.37237
H   1.66244  5.05153 -0.46559
C  -2.31038  2.80269  0.11839
C  -2.73679  3.05388  1.44694
C  -4.11823  2.96416  1.70537
H  -4.48747  3.15975  2.71652
C  -5.02046  2.62115  0.69124
H  -6.08936  2.55469  0.91516
C  -4.56319  2.35533 -0.60749
H  -5.28015  2.09328 -1.39074
C  -3.19695  2.45941 -0.92891
C  -1.75908  3.45175  2.55007
H  -0.73763  3.40807  2.13577
C  -2.01725  4.90244  3.00887
H  -1.94881  5.60972  2.16590
H  -1.27689  5.20239  3.76884
H  -3.02125  5.00742  3.45406
C  -1.80267  2.47288  3.73869
H  -2.80368  2.42537  4.19953
H  -1.08632  2.78578  4.51654
H  -1.52732  1.45483  3.41694
C  -2.70179  2.31068 -2.36175
H  -1.61288  2.12243 -2.32271
C  -3.35859  1.13350 -3.10131
H  -3.26225  0.19144 -2.53883
H  -2.88181  0.98617 -4.08404
H  -4.43252  1.31073 -3.28170
C  -2.90066  3.63881 -3.12696
H  -3.97045  3.90383 -3.17665
H  -2.52041  3.54996 -4.15839
H  -2.36782  4.46959 -2.63544
C   2.59822  2.50341 -0.34798
C   3.28731  2.57518 -1.58169
C   4.66011  2.25978 -1.56575
H   5.23094  2.30285 -2.49798
C   5.29812  1.88837 -0.37718
H   6.36450  1.64302 -0.38709
C   4.58392  1.83491  0.83012
H   5.10307  1.56418  1.75379
C   3.21599  2.15537  0.87708
C   2.60779  3.05273 -2.86280
H   1.52590  3.14078 -2.66147
C   3.13471  4.45234 -3.25140
H   3.00082  5.18224 -2.43583
H   2.60555  4.83142 -4.14154
H   4.21188  4.41420 -3.48708
C   2.77283  2.06634 -4.03395
H   3.83401  1.90575 -4.28895
H   2.26886  2.45919 -4.93275
H   2.33497  1.08491 -3.79424
C   2.45078  2.23516  2.19406
H   1.37851  2.11711  1.96612
C   2.64733  3.63045  2.82836
H   3.70971  3.80816  3.06782
H   2.06812  3.71076  3.76403
H   2.31395  4.43425  2.15059
C   2.81634  1.11045  3.17144
H   2.66556  0.12330  2.71064
H   2.17471  1.16349  4.06606
H   3.86293  1.17461  3.51549
C  -4.03315 -0.28647  2.12251
H  -4.15141 -1.35794  2.35420
H  -4.88436  0.04889  1.51443
H  -4.03504  0.28028  3.06780
C   3.72244 -0.89949 -2.12884
H   3.77657 -2.00139 -2.17085
H   4.55887 -0.52405 -1.52169
H   3.83850 -0.50258 -3.14848
C  -0.02991 -2.01342  0.06274
C  -0.29699 -4.24141 -0.47436
H  -0.64128 -5.07896 -1.07208
C   0.41982 -4.18607  0.68070
H   0.82592 -4.96460  1.31782
C  -1.36082 -2.63391 -2.02021
C  -0.69482 -2.53879 -3.26629
C  -1.48567 -2.36828 -4.41821
H  -1.00036 -2.29304 -5.39457
C  -2.87997 -2.31649 -4.33496
H  -3.47847 -2.19659 -5.24325
C  -3.51349 -2.41961 -3.09167
H  -4.60344 -2.37457 -3.04256
C  -2.77407 -2.57962 -1.90444
C   0.81516 -2.67720 -3.38937
H   1.26003 -2.40318 -2.41438
C   1.40118 -1.73676 -4.45648
H   1.08385 -2.02327 -5.47303
H   2.50014 -1.77801 -4.43283
H   1.08954 -0.69247 -4.28488
C   1.21243 -4.14112 -3.68294
H   0.87111 -4.82474 -2.88964
H   2.30911 -4.23002 -3.75888
H   0.77291 -4.47942 -4.63688
C  -3.48918 -2.72187 -0.56456
H  -2.84201 -2.26255  0.21012
C  -4.84631 -1.99695 -0.54423
H  -4.75117 -0.93531 -0.82777
H  -5.28009 -2.04283  0.46488
H  -5.56831 -2.47569 -1.22672
C  -3.68739 -4.20590 -0.17966
H  -4.29177 -4.72388 -0.94377
H  -4.21838 -4.27835  0.78427
H  -2.73073 -4.73905 -0.07644
C   1.24605 -2.46113  2.22738
C   2.65895 -2.50066  2.25248
C   3.29000 -2.32174  3.49922
H   4.38094 -2.35952  3.55689
C   2.54581 -2.08638  4.65855
H   3.05582 -1.94483  5.61633
```

C	1.14788	-2.03023	4.59757
H	0.57791	-1.84816	5.51175
C	0.46352	-2.23857	3.38642
C	3.49290	-2.76115	1.00350
H	2.86426	-2.53481	0.12188
C	4.73160	-1.84895	0.94218
H	4.45096	-0.78522	0.99348
H	5.27366	-2.01477	-0.00139
H	5.43464	-2.06192	1.76486
C	3.92020	-4.24324	0.91544
H	4.53320	-4.52218	1.78967
H	4.52255	-4.41394	0.00736
H	3.05461	-4.92308	0.87934
C	-1.05951	-2.30626	3.35498
H	-1.38761	-1.92445	2.36912
C	-1.71637	-1.44018	4.44232
H	-1.54209	-1.85504	5.44950
H	-2.80547	-1.40415	4.29078
H	-1.33086	-0.40775	4.42920
C	-1.55890	-3.76453	3.46754
H	-1.18325	-4.39374	2.64586
H	-2.66149	-3.79106	3.43632
H	-1.23225	-4.21241	4.42148
H	-0.61604	0.29000	1.53273
H	-1.09723	0.45970	-1.41822
H	-1.14586	-0.40761	-1.38609
H	1.25357	0.03086	0.87972

TS(1,3-1,3)-4^{+}

SCF = -2961.66572836
H(0 K)= -2960.452155
G(298 K)= -2960.566207
SCF (C6H5F) = -2961.71221447
Lowest Frequencies = -490.6889cm-1, 22.1009cm-1
SCF [PBE0(C6H5F, D3)] = -6063.13389485

145

Ir	0.03918	0.11148	0.01081
Zn	-0.57355	-0.81652	-2.20533
Zn	0.36783	-0.93772	2.28430
N	-3.03555	-0.58794	0.57925
N	-2.46409	1.31512	1.46971
N	2.72660	0.76483	-1.47589
N	2.85867	-1.22121	-0.59503
C	-1.95399	0.28044	0.68693
C	-4.14719	-0.11196	1.28070
H	-5.08235	-0.66291	1.30266
C	-3.79220	1.08097	1.83091
H	-4.35025	1.78871	2.43643
C	-3.20013	-1.72043	-0.31898
C	-2.93390	-3.03129	0.15418
C	-3.17943	-4.09950	-0.73134
H	-2.98529	-5.12371	-0.40028
C	-3.69374	-3.87875	-2.01562
H	-3.87900	-4.72648	-2.68305
C	-4.00485	-2.58002	-2.43248
H	-4.44822	-2.42201	-3.42044
C	-3.78240	-1.46921	-1.59328
C	-2.52361	-3.30615	1.60067
H	-2.03701	-2.39461	1.99415
C	-3.78106	-3.57583	2.46573
H	-4.48932	-2.73140	2.44393
H	-3.49389	-3.74903	3.51696
H	-4.31544	-4.47245	2.10679
C	-1.52373	-4.46867	1.75013
H	-1.96807	-5.43468	1.45444
H	-1.22149	-4.56459	2.80578
H	-0.61334	-4.30906	1.15005
C	-4.29410	-0.08922	-2.01996
H	-3.88401	0.66158	-1.32225
C	-3.86374	0.32324	-3.44290
H	-2.76715	0.33522	-3.55248
H	-4.24415	1.33338	-3.67100
H	-4.26818	-0.35806	-4.21080
C	-5.83864	-0.04487	-1.90291
H	-6.30508	-0.76730	-2.59464
H	-6.21526	0.96016	-2.15858
H	-6.18355	-0.28842	-0.88463
C	-1.84175	2.58809	1.80032
C	-1.96010	3.65266	0.86552
C	-1.43544	4.90543	1.24007
H	-1.51312	5.74611	0.54422
C	-0.84435	5.10042	2.49362
H	-0.45551	6.08633	2.76798
C	-0.76720	4.04050	3.40441
H	-0.32224	4.20961	4.38949
C	-1.26418	2.76133	3.08416
C	-2.70942	3.51060	-0.46195
H	-2.77029	2.43491	-0.70698
C	-4.15681	4.04290	-0.31655
H	-4.71353	3.50913	0.47118
H	-4.70937	3.92366	-1.26429
H	-4.15360	5.11610	-0.05895
C	-2.00004	4.20796	-1.64170
H	-1.99769	5.30578	-1.52940
H	-2.53199	3.98301	-2.58158
H	-0.95379	3.87857	-1.75296
C	-1.23059	1.65053	4.13351
H	-1.39271	0.68733	3.61289
C	-2.37809	1.82626	5.15831
H	-2.27439	2.78239	5.69984
H	-2.35927	1.01091	5.90137
H	-3.36902	1.82105	4.67530
C	0.12593	1.56424	4.86477
H	0.96510	1.48280	4.15331
H	0.14327	0.68154	5.52500
H	0.30632	2.44845	5.49960
C	-0.74813	-1.72030	-3.94789
H	0.24527	-1.98655	-4.34999
H	-1.26151	-1.08759	-4.68987
H	-1.33499	-2.64492	-3.81813
C	0.20664	-2.04257	3.88370
H	-0.83716	-2.07185	4.23908
H	0.83677	-1.63932	4.69278
H	0.53746	-3.06944	3.66309
C	1.99187	-0.14040	-0.71053
C	3.97879	0.25050	-1.82096
H	4.68618	0.82482	-2.41145

```
C   4.05990 -0.99266 -1.27477
H   4.85260 -1.73417 -1.29010
C   2.40334  2.14804 -1.78247
C   2.75272  3.14180 -0.82660
C   2.51860  4.48663 -1.17429
H   2.77880  5.27474 -0.46108
C   1.98535  4.83191 -2.42147
H   1.82412  5.88469 -2.67504
C   1.67303  3.83385 -3.35256
H   1.27425  4.11765 -4.33087
C   1.87636  2.47006 -3.06152
C   3.43346  2.81009  0.50143
H   3.32227  1.72626  0.67861
C   2.78942  3.53335  1.70226
H   2.91523  4.62803  1.64040
H   3.27302  3.20559  2.63840
H   1.71176  3.31612  1.77713
C   4.94705  3.12571  0.41806
H   5.43783  2.56859 -0.39739
H   5.44913  2.85886  1.36376
H   5.11582  4.20158  0.23856
C   1.59152  1.41253 -4.12714
H   1.51418  0.43213 -3.62056
C   0.26072  1.65898 -4.86856
H  -0.58479  1.75487 -4.16645
H   0.04856  0.81754 -5.54775
H   0.29607  2.57406 -5.48408
C   2.75917  1.32820 -5.14100
H   2.88900  2.28916 -5.66831
H   2.55627  0.55018 -5.89639
H   3.71571  1.08255 -4.65113
C   2.73284 -2.38812  0.26059
C   3.31262 -2.32485  1.55888
C   3.24982 -3.48983  2.35152
H   3.68732 -3.47757  3.35458
C   2.66623 -4.66616  1.86804
H   2.63546 -5.56021  2.49909
C   2.15275 -4.71236  0.56513
H   1.73944 -5.65060  0.18370
C   2.18300 -3.58172 -0.27495
C   4.11261 -1.11518  2.05487
H   3.89046 -0.26056  1.39208
C   3.76448 -0.68373  3.49456
H   2.69709 -0.42739  3.60207
H   4.35613  0.20463  3.77294
H   3.99440 -1.47268  4.23078
C   5.62991 -1.41183  1.94878
H   5.91129 -2.25312  2.60533
H   6.21705 -0.53007  2.25696
H   5.92824 -1.67649  0.92125
C   1.74948 -3.69104 -1.73677
H   1.51638 -2.67214 -2.09763
C   0.49127 -4.55600 -1.94403
H   0.67275 -5.61482 -1.69038
H   0.19026 -4.52453 -3.00386
H  -0.35909 -4.19763 -1.34134
C   2.91688 -4.23689 -2.59748
H   3.81407 -3.59964 -2.53198
H   2.61759 -4.29172 -3.65801
H   3.20057 -5.25238 -2.27076
H  -0.08606 -1.50202  0.11577
H  -0.37044  1.80008 -0.32756
H   0.43033  1.73365 -0.62846
H   0.66134  0.69097  1.54853
```

TS(1,2-1,2)1-4$^+$

```
SCF  =            -2961.68362227
H(0 K)=           -2960.463251
G(298 K)=         -2960.570492
SCF (C6H5F) =      -2961.73005178
Lowest Frequencies = -468.1231cm-1,  26.7632cm-1
SCF [PBE0(C6H5F, D3)] =
-6063.14661246

145
Ir  0.01760  0.16140  0.08140
Zn  1.10525 -2.01262  0.87903
Zn -0.49577 -1.90322 -1.24096
N   2.40076  0.01742 -1.95503
N   0.78159  1.20930 -2.76165
N  -1.13665  0.43624  2.93686
N  -2.33375 -0.98510  1.82830
C   1.13598  0.48646 -1.64386
C   2.80818  0.44288 -3.22241
H   3.78007  0.17794 -3.62586
C   1.78638  1.19009 -3.73118
H   1.67945  1.72634 -4.66867
C   3.28528 -0.67523 -1.04936
C   3.44132 -2.07539 -1.17936
C   4.35320 -2.70588 -0.31003
H   4.49926 -3.78693 -0.37919
C   5.06252 -1.97192  0.64684
H   5.75941 -2.48317  1.31774
C   4.88845 -0.58464  0.74804
H   5.45969 -0.02217  1.49125
C   4.00521  0.10045 -0.10609
C   2.70832 -2.86674 -2.25582
H   1.82388 -2.27761 -2.56160
C   3.60596 -3.04997 -3.50020
H   3.92788 -2.08475 -3.92219
H   3.06366 -3.60110 -4.28644
H   4.51289 -3.62295 -3.24198
C   2.20919 -4.23399 -1.75607
H   3.04405 -4.92139 -1.53971
H   1.57889 -4.70817 -2.52460
H   1.61137 -4.13671 -0.83465
C   3.89266  1.62285 -0.07424
H   2.88228  1.89493 -0.42784
C   4.05685  2.22430  1.33021
H   3.35587  1.78036  2.05343
H   3.85340  3.30678  1.29639
H   5.08156  2.09576  1.71846
C   4.90513  2.25537 -1.05655
H   5.93879  2.01179 -0.75774
H   4.79742  3.35322 -1.05988
H   4.75340  1.89709 -2.08693
C  -0.33025  2.12746 -2.83180
C  -0.12651  3.42502 -2.30906
C  -1.21126  4.31796 -2.37860
```

```
H  -1.09807  5.33098 -1.97972
C  -2.42127  3.93465 -2.97231
H  -3.25071  4.64660 -3.02477
C  -2.56940  2.65358 -3.51977
H  -3.51198  2.37795 -4.00057
C  -1.52500  1.71242 -3.45371
C   1.22827  3.88247 -1.77565
H   1.91804  3.02346 -1.81798
C   1.81674  4.97810 -2.69062
H   1.88982  4.63202 -3.73502
H   2.82686  5.26191 -2.34991
H   1.18993  5.88595 -2.68150
C   1.17737  4.34586 -0.30842
H   0.50219  5.20839 -0.17825
H   2.18247  4.65851  0.02298
H   0.83026  3.53750  0.35397
C  -1.64013  0.33598 -4.09709
H  -0.88735 -0.31993 -3.62271
C  -1.30097  0.42602 -5.60220
H  -2.02322  1.07620 -6.12508
H  -1.33870 -0.57362 -6.06688
H  -0.29422  0.84260 -5.77006
C  -3.01309 -0.32024 -3.88619
H  -3.23265 -0.45174 -2.81626
H  -3.02938 -1.31489 -4.36037
H  -3.82781  0.27037 -4.33877
C   1.96120 -3.30673  2.07924
H   1.38243 -3.43247  3.01089
H   2.97569 -2.96176  2.33263
H   2.04849 -4.29070  1.58935
C  -0.97013 -3.03277 -2.76240
H  -2.03168 -2.87981 -3.00947
H  -0.82438 -4.09691 -2.51684
H  -0.35670 -2.79066 -3.64719
C  -1.23873 -0.15539  1.69280
C  -2.14127  0.00327  3.80440
H  -2.21985  0.38177  4.81852
C  -2.89937 -0.88841  3.10476
H  -3.79236 -1.44456  3.37117
C  -0.24491  1.52567  3.23944
C  -0.67423  2.83185  2.90713
C   0.21102  3.88641  3.19496
H  -0.08253  4.91306  2.95712
C   1.45432  3.64031  3.79250
H   2.12520  4.47593  4.01463
C   1.84241  2.33365  4.11800
H   2.81094  2.16138  4.59539
C   1.00219  1.23877  3.83823
C  -2.07035  3.09609  2.35399
H  -2.47134  2.13781  1.98459
C  -2.08962  4.06695  1.16216
H  -1.67525  5.05602  1.42223
H  -3.12888  4.22449  0.82860
H  -1.52149  3.65971  0.31266
C  -2.99249  3.58688  3.49267
H  -3.02149  2.86839  4.32868
H  -4.02237  3.72385  3.12220
H  -2.64191  4.55340  3.89344
C   1.39199 -0.18890  4.20828
H   0.80939 -0.87409  3.56358
C   2.88129 -0.49024  3.96864
H   3.16707 -0.33078  2.91588
H   3.09216 -1.54277  4.21460
H   3.53523  0.13297  4.60172
C   1.00151 -0.48494  5.67364
H   1.54912  0.18013  6.36309
H   1.24578 -1.52820  5.93481
H  -0.07655 -0.33299  5.84509
C  -2.99236 -1.68771  0.75684
C  -3.97529 -0.98136  0.02658
C  -4.63667 -1.68056 -1.00115
H  -5.40393 -1.16711 -1.58883
C  -4.34155 -3.02374 -1.26572
H  -4.86796 -3.54938 -2.06814
C  -3.38708 -3.70502 -0.49880
H  -3.17918 -4.75738 -0.70746
C  -2.68820 -3.05299  0.53537
C  -4.36605  0.45206  0.37056
H  -3.76567  0.76828  1.23948
C  -4.05252  1.43328 -0.77182
H  -2.97876  1.43709 -1.01149
H  -4.33666  2.45877 -0.48236
H  -4.61040  1.17923 -1.68876
C  -5.84931  0.52413  0.79033
H  -6.51825  0.25547 -0.04491
H  -6.10534  1.54890  1.10735
H  -6.06582 -0.16019  1.62746
C  -1.72272 -3.80675  1.44373
H  -0.99115 -3.07275  1.83432
C  -0.93964 -4.91125  0.71495
H  -1.59568 -5.74491  0.41301
H  -0.16553 -5.32395  1.38029
H  -0.44447 -4.53002 -0.19366
C  -2.47258 -4.39940  2.65815
H  -2.97044 -3.61857  3.25395
H  -1.76884 -4.93308  3.31857
H  -3.24243 -5.11564  2.32401
H  -1.29121  0.04773 -0.84909
H   1.53650  0.78742  0.71863
H   1.08550  0.61086  1.43512
H  -0.44691  1.72043  0.06500
```

TS(1,2-1,2)2-4$^+$

```
SCF  =           -2961.69132574
H(0 K)=          -2960.472676
G(298 K)=        -2960.580618
SCF (C6H5F) =    -2961.73637296
Lowest Frequencies = -335.9568cm-
1,  24.1906cm-1
SCF [PBE0(C6H5F, D3)] =
-6063.15452496

145
Ir -0.03243  0.19214 -0.01722
Zn  0.91489 -1.75439  1.11726
Zn -0.84555 -1.72529 -1.32507
N   2.52309 -0.48753 -1.73056
N   1.23816  0.92646 -2.74930
N  -1.26441  0.70705  2.78199
N  -2.52871 -0.67598  1.69851
```

C	1.33355	0.19784	-1.57752
C	3.14121	-0.18327	-2.94841
H	4.09372	-0.62294	-3.22558
C	2.33109	0.70731	-3.58954
H	2.42634	1.21549	-4.54391
C	3.20615	-1.23547	-0.70333
C	3.20787	-2.64849	-0.76980
C	3.98195	-3.33632	0.18618
H	4.01257	-4.42884	0.16590
C	4.69827	-2.64157	1.16652
H	5.28791	-3.19566	1.90325
C	4.66510	-1.23992	1.21344
H	5.23645	-0.71114	1.98125
C	3.93086	-0.49980	0.27014
C	2.44995	-3.39695	-1.85940
H	1.67251	-2.71636	-2.25301
C	3.39189	-3.76808	-3.02637
H	3.86027	-2.87920	-3.47801
H	2.83286	-4.29556	-3.81718
H	4.19976	-4.43294	-2.67576
C	1.74190	-4.65342	-1.32397
H	2.46231	-5.42680	-1.00850
H	1.10930	-5.09378	-2.11092
H	1.10367	-4.41510	-0.45908
C	3.98474	1.02328	0.23237
H	3.06966	1.37115	-0.27361
C	3.98235	1.67472	1.62173
H	3.08100	1.39529	2.18543
H	3.97865	2.77206	1.51709
H	4.87146	1.40565	2.21740
C	5.20110	1.48974	-0.59898
H	6.14511	1.17200	-0.12425
H	5.21071	2.59050	-0.67524
H	5.17981	1.07911	-1.62145
C	0.28645	1.98989	-2.95545
C	0.63505	3.27507	-2.47640
C	-0.32155	4.29683	-2.63143
H	-0.08926	5.30665	-2.27895
C	-1.55344	4.04063	-3.24637
H	-2.28349	4.84810	-3.35871
C	-1.84955	2.76227	-3.73966
H	-2.80727	2.58452	-4.23599
C	-0.93488	1.70271	-3.60142
C	2.01315	3.58065	-1.89327
H	2.55960	2.62908	-1.78757
C	2.81432	4.45731	-2.88091
H	2.88999	3.97797	-3.87097
H	3.83583	4.62930	-2.50221
H	2.33298	5.44021	-3.02068
C	1.95712	4.22848	-0.49627
H	1.40550	5.18388	-0.50998
H	2.97975	4.44449	-0.14358
H	1.48168	3.56900	0.24755
C	-1.20075	0.32442	-4.19223
H	-0.58321	-0.40146	-3.63158
C	-0.73392	0.28630	-5.66508
H	-1.30973	1.00365	-6.27458
H	-0.87971	-0.72127	-6.08933
H	0.33327	0.54605	-5.75994
C	-2.66424	-0.12643	-4.06869
H	-2.99108	-0.14581	-3.01766
H	-2.77627	-1.14340	-4.47740
H	-3.34800	0.53019	-4.63317
C	1.36656	-3.20848	2.33209
H	0.68541	-3.21846	3.20027
H	2.39931	-3.07913	2.68885
H	1.29832	-4.18138	1.82096
C	-1.17217	-3.08361	-2.68229
H	-2.19728	-2.95481	-3.06394
H	-1.08411	-4.10064	-2.27083
H	-0.46313	-2.98186	-3.52169
C	-1.35976	0.04971	1.57015
C	-2.33829	0.40618	3.62220
H	-2.42394	0.84090	4.61285
C	-3.13721	-0.46259	2.93895
H	-4.08175	-0.93102	3.19581
C	-0.28693	1.71866	3.10594
C	-0.53352	3.04061	2.66231
C	0.42091	4.01702	3.00026
H	0.27200	5.04975	2.67337
C	1.54661	3.68848	3.76786
H	2.27075	4.46576	4.03064
C	1.74534	2.37488	4.20980
H	2.62382	2.13612	4.81575
C	0.83695	1.35259	3.87591
C	-1.81932	3.40963	1.92450
H	-2.17981	2.50542	1.40162
C	-1.63308	4.50323	0.85871
H	-1.36229	5.47412	1.30742
H	-2.57761	4.64968	0.30943
H	-0.85856	4.23585	0.12367
C	-2.91330	3.82314	2.93549
H	-3.11727	3.02344	3.66500
H	-3.85556	4.05238	2.40950
H	-2.60359	4.72241	3.49451
C	1.02668	-0.07444	4.37690
H	0.41514	-0.73784	3.73785
C	2.48296	-0.56197	4.28155
H	2.85488	-0.52766	3.24563
H	2.54911	-1.60535	4.62888
H	3.16038	0.03879	4.91165
C	0.50510	-0.20423	5.82580
H	1.08166	0.44613	6.50575
H	0.60373	-1.24414	6.17976
H	-0.55548	0.08458	5.90674
C	-3.21414	-1.35048	0.62513
C	-4.06235	-0.56894	-0.19383
C	-4.74415	-1.23376	-1.22987
H	-5.40611	-0.66211	-1.88785
C	-4.60360	-2.61576	-1.41444
H	-5.14549	-3.11474	-2.22359
C	-3.78325	-3.36653	-0.56249
H	-3.69194	-4.44495	-0.71368
C	-3.06273	-2.75009	0.47981
C	-4.29874	0.91396	0.07102
H	-3.65052	1.21569	0.91009
C	-3.92120	1.80028	-1.12858
H	-2.86318	1.66877	-1.40099
H	-4.08110	2.86323	-0.88000
H	-4.53677	1.57089	-2.01521

```
C  -5.75832  1.15037  0.51451
H  -6.46798  0.88697 -0.28822
H  -5.91409  2.21255  0.76753
H  -6.01298  0.54449  1.39993
C  -2.22243 -3.55763  1.46268
H  -1.43022 -2.88923  1.84945
C  -1.53703 -4.77162  0.81620
H  -2.26673 -5.54341  0.51797
H  -0.84312 -5.23654  1.53409
H  -0.96582 -4.48232 -0.07935
C  -3.08100 -4.01012  2.66567
H  -3.51656 -3.15570  3.20674
H  -2.46585 -4.58411  3.37867
H  -3.90899 -4.65641  2.32767
H  -1.31690  0.17768 -1.11021
H   1.20319  0.38325  1.08319
H   0.07066  1.97283 -0.39615
H  -0.45377  1.93417  0.26026
```

[Ir(IMe)$_2$H$_2$]$^+$ Model System

```
SCF  =           -715.308400270
H(0 K)=          -715.041441
G(298 K)=        -715.092742
Lowest Frequencies = 11.8974cm-1,
36.6443cm-1

33
Ir  0.00003 -0.03086 -0.00007
C   2.04759 -0.00152  0.00240
N   2.85100  1.08203  0.29929
C   4.19890  0.76311  0.19967
C   4.25909 -0.55448 -0.17010
N   2.94935 -1.00367 -0.27936
C  -2.04758 -0.00156 -0.00258
N  -2.85106  1.08195 -0.29947
C  -4.19893  0.76301 -0.19964
C  -4.25907 -0.55454  0.17021
N  -2.94931 -1.00371  0.27934
H   4.98721  1.48279  0.40322
H   5.11245 -1.19960 -0.36093
H  -4.98729  1.48266 -0.40319
H  -5.11240 -1.19971  0.36107
H   0.00726 -1.10216  1.10917
H  -0.00702 -1.10589 -1.10569
C  -2.34479  2.38738 -0.71873
H  -1.24435  2.36872 -0.65545
H  -2.63786  2.60217 -1.75832
H  -2.72508  3.17902 -0.05474
C  -2.58265 -2.35870  0.69544
H  -2.45391 -2.40819  1.78851
H  -3.37590 -3.05502  0.38862
H  -1.64210 -2.63937  0.20355
C   2.34457  2.38726  0.71897
H   2.72646  3.17935  0.05644
H   1.24424  2.36923  0.65356
H   2.63583  2.60091  1.75930
C   2.58258 -2.35869 -0.69529
H   1.64525 -2.64183 -0.19867
H   2.44802 -2.40695 -1.78770
H   3.37873 -3.05400 -0.39376
```

ZnMe$_2$

```
SCF  =           -307.063352729
H(0 K)=          -306.994303
G(298 K)=        -307.024340
SCF (C6H5F) =    -307.071574940
Lowest Frequencies = 16.3999cm-1,
141.0734cm-1
SCF [PBE0(C6H5F, D3)] =
-1858.9015026

9
Zn -0.00000  0.00038  0.00016
C   1.94034 -0.00055 -0.00025
H   2.33716 -1.02927  0.05172
H   2.33822  0.55861  0.86432
H   2.33771  0.46842 -0.91709
C  -1.94033 -0.00051 -0.00028
H  -2.33707 -0.74238 -0.71489
H  -2.33846  0.98884 -0.28503
H  -2.33759 -0.24908  0.99917
```

CH$_4$

```
SCF  =           -40.5177381668
H(0 K)=          -40.473821
G(298 K)=        -40.493508
SCF (C6H5F) =    -40.5178923208
Lowest Frequencies = 1305.4191cm-
1,  1305.4379cm-1
SCF [PBE0(C6H5F, D3)] =
-40.4749526245

5
C   0.00000  0.00000  0.00000
H  -0.49650  0.90780 -0.37204
H  -0.26418 -0.85194 -0.64298
H  -0.32904 -0.20195  1.02955
H   1.08972  0.14610 -0.01452
```

Int(1$^+$-2$^+$)

```
SCF  =           -2692.68543095
H(0 K)=          -2691.524834
G(298 K)=        -2691.625443
SCF (C6H5F) =    -2692.72806056
Lowest Frequencies = 21.4668cm-1,
24.9372cm-1
SCF [PBE0(C6H5F, D3)] =
-4242.28893782

137
Ir  0.04884 -0.00525 -0.22521
H   1.19704 -0.03005  0.82622
N  -0.06930 -2.34459  1.77477
N  -1.83852 -2.40763  0.52820
N   0.21861  2.94607 -1.18849
N   2.07095  1.91057 -1.59367
C  -0.65610 -1.69862  0.69619
C  -0.85702 -3.39130  2.25380
H  -0.53153 -4.01284  3.08227
C  -1.97180 -3.43150  1.46953
```

H	-2.83254	-4.09292	1.46837
C	1.27264	-2.15575	2.27103
C	1.45711	-1.46777	3.48848
C	2.75555	-1.45695	4.03281
H	2.93439	-0.94889	4.98514
C	3.82027	-2.07242	3.36433
H	4.82330	-2.04884	3.80159
C	3.61102	-2.71376	2.13547
H	4.45213	-3.18653	1.62106
C	2.32495	-2.79432	1.57289
C	0.30602	-0.76531	4.19580
H	-0.55582	-0.75282	3.50324
C	-0.12279	-1.53032	5.46499
H	-0.42930	-2.56294	5.22949
H	-0.97184	-1.02429	5.95452
H	0.70627	-1.58421	6.19123
C	0.66083	0.69915	4.51808
H	1.50133	0.76701	5.22905
H	-0.20267	1.20979	4.97426
H	0.94757	1.24645	3.60526
C	2.06688	-3.53411	0.26608
H	1.00090	-3.82140	0.24967
C	2.32149	-2.60130	-0.93164
H	1.70387	-1.68038	-0.85537
H	2.07971	-3.10524	-1.88283
H	3.37299	-2.27916	-0.96382
C	2.88877	-4.82950	0.13311
H	3.96756	-4.62292	0.02954
H	2.57663	-5.38059	-0.76976
H	2.74900	-5.48986	1.00445
C	-2.80762	-2.25533	-0.53060
C	-2.50592	-2.79911	-1.80499
C	-3.51846	-2.74829	-2.77932
H	-3.32649	-3.15566	-3.77487
C	-4.77146	-2.18657	-2.49615
H	-5.54693	-2.16975	-3.26818
C	-5.03066	-1.64186	-1.23421
H	-6.00870	-1.19884	-1.02502
C	-4.05003	-1.66027	-0.22208
C	-1.14362	-3.42047	-2.10252
H	-0.38562	-2.79155	-1.59965
C	-1.03659	-4.85083	-1.52491
H	-1.19700	-4.87531	-0.43625
H	-0.03583	-5.26865	-1.72848
H	-1.78364	-5.51249	-1.99595
C	-0.80474	-3.44359	-3.60182
H	-1.43603	-4.16199	-4.15265
H	0.24143	-3.76179	-3.74149
H	-0.92412	-2.45275	-4.06920
C	-4.34954	-1.06833	1.14970
H	-3.39257	-0.97532	1.69522
C	-5.27186	-2.00048	1.96511
H	-6.24211	-2.13228	1.45626
H	-5.46563	-1.57441	2.96375
H	-4.82778	-2.99980	2.10136
C	-4.96096	0.34092	1.03820
H	-4.30402	1.01958	0.47081
H	-5.10988	0.77057	2.04161
H	-5.94445	0.31961	0.53913
C	0.82437	1.70907	-1.03149
C	1.04985	3.86053	-1.83941
H	0.71943	4.86810	-2.07447
C	2.22189	3.20716	-2.08895
H	3.14369	3.52922	-2.56434
C	-1.10836	3.26931	-0.73899
C	-1.25730	4.13210	0.37683
C	-2.57397	4.42880	0.77755
H	-2.73876	5.08962	1.63174
C	-3.67726	3.89276	0.09905
H	-4.68990	4.13453	0.43589
C	-3.49440	3.06283	-1.01534
H	-4.36427	2.67183	-1.55069
C	-2.20112	2.74680	-1.46833
C	-0.03979	4.70810	1.10247
H	0.71312	4.95825	0.33346
C	0.61116	3.66455	2.03787
H	-0.06034	3.43059	2.88001
H	1.55391	4.05648	2.45550
H	0.84246	2.72435	1.50783
C	-0.34834	6.00044	1.87696
H	-0.83503	6.75540	1.23791
H	0.58698	6.43400	2.26642
H	-1.00285	5.80933	2.74488
C	-1.96476	1.84906	-2.67476
H	-0.93185	2.02222	-3.02161
C	-2.08201	0.37279	-2.26482
H	-1.64483	0.19407	-1.23545
H	-1.62135	-0.30669	-2.99402
H	-3.13315	0.07566	-2.11866
C	-2.90984	2.14948	-3.85317
H	-3.96123	1.93148	-3.60062
H	-2.64515	1.52049	-4.71935
H	-2.84295	3.20526	-4.16123
C	3.15813	0.96286	-1.59493
C	3.98983	0.91684	-0.45720
C	5.08167	0.02814	-0.49402
H	5.74668	-0.03970	0.37239
C	5.34366	-0.73801	-1.63630
H	6.20814	-1.40910	-1.65534
C	4.51329	-0.64392	-2.76399
H	4.74027	-1.23930	-3.65216
C	3.39569	0.20876	-2.76681
C	3.77765	1.85269	0.72479
H	2.82268	2.38097	0.57123
C	3.66388	1.08684	2.04968
H	2.85473	0.34244	2.00325
H	3.44898	1.78052	2.88013
H	4.59420	0.54931	2.29655
C	4.89435	2.91745	0.77316
H	5.88188	2.45126	0.93116
H	4.71693	3.62362	1.60199
H	4.94195	3.49399	-0.16578
C	2.45632	0.32075	-3.96263
H	2.07118	1.35692	-3.98564
C	1.24373	-0.61520	-3.77643
H	1.56602	-1.66889	-3.78345
H	0.51218	-0.47253	-4.59050
H	0.73403	-0.42888	-2.81185
C	3.14606	0.05278	-5.31145
H	4.04163	0.68190	-5.44222

H 2.45112 0.26811 -6.13960
H 3.45161 -1.00295 -5.41117
Zn -1.29201 1.05074 1.43996
H -3.22852 2.27012 2.51656
C -2.41653 1.64436 2.91403
H -2.84175 0.77378 3.43940
H -1.81934 2.23588 3.62444

TS(2⁺-3⁺)1

SCF = -2959.26052400
H(0 K)= -2958.077151
G(298 K)= -2958.181569
SCF (C6H5F) = -2959.30599756
Lowest Frequencies = -764.3454cm-1, 23.2778cm-1
SCF [PBE0(C6H5F, D3)] = -6060.72347635

141
Ir 0.11377 -0.07698 -0.24716
Zn -1.86773 -0.45699 1.23801
Zn -0.14038 1.74788 1.31382
N 2.93855 -0.36014 1.13154
N 1.39213 -1.28827 2.32803
N -2.09100 1.67126 -1.62729
N -2.03172 -0.34544 -2.40986
C 1.57406 -0.61538 1.12221
C 3.56040 -0.84972 2.28008
H 4.62578 -0.72796 2.44659
C 2.58947 -1.44656 3.02807
H 2.63198 -1.98430 3.96962
C 3.69722 0.21087 0.04450
C 3.84301 1.61492 -0.01827
C 4.57270 2.13567 -1.10336
H 4.70254 3.21719 -1.19501
C 5.14904 1.28722 -2.05844
H 5.71336 1.71380 -2.89348
C 5.02682 -0.10310 -1.93912
H 5.49711 -0.75292 -2.68229
C 4.29567 -0.67614 -0.88260
C 3.33748 2.50728 1.10982
H 2.47875 1.99696 1.58285
C 4.44046 2.65193 2.18442
H 4.76266 1.67316 2.57444
H 4.07246 3.25299 3.03291
H 5.32746 3.15585 1.76385
C 2.85470 3.89125 0.65042
H 3.65490 4.46596 0.15425
H 2.52870 4.47707 1.52423
H 2.00100 3.81552 -0.03940
C 4.19092 -2.19192 -0.72822
H 3.29606 -2.40367 -0.11442
C 5.42614 -2.73854 0.02481
H 6.34610 -2.53902 -0.55056
H 5.34058 -3.82893 0.16730
H 5.54301 -2.27432 1.01621
C 4.02524 -2.94358 -2.06317
H 3.16965 -2.56617 -2.64540
H 3.85865 -4.01590 -1.86759
H 4.92762 -2.86542 -2.69268
C 0.19085 -1.95586 2.76804
C -0.11542 -3.21043 2.18467
C -1.23608 -3.90195 2.67706
H -1.50672 -4.86732 2.24051
C -1.98961 -3.38447 3.73960
H -2.84957 -3.94321 4.12083
C -1.64779 -2.15563 4.31559
H -2.24647 -1.76235 5.14137
C -0.55271 -1.40719 3.84120
C 0.72559 -3.76191 1.04361
H 1.72281 -3.29074 1.10468
C 0.93496 -5.28586 1.11497
H -0.00779 -5.83779 0.96210
H 1.35669 -5.59023 2.08655
H 1.63043 -5.60562 0.32143
C 0.09893 -3.36408 -0.29989
H 0.76187 -3.59050 -1.14883
H -0.11246 -2.26356 -0.33296
H -0.86327 -3.87304 -0.45990
C -0.17951 -0.07523 4.48014
H 0.45372 0.47330 3.75843
C 0.64051 -0.28597 5.77218
H 0.90656 0.68631 6.21956
H 1.57445 -0.83857 5.58461
H 0.05481 -0.85524 6.51410
C -1.41723 0.79187 4.77329
H -2.05666 0.89379 3.88185
H -1.10707 1.79870 5.09424
H -2.03499 0.36281 5.57997
C -3.67057 -0.93722 1.83694
H -4.41738 -0.70186 1.05869
H -3.93428 -0.38719 2.75524
H -3.71620 -2.01474 2.05880
C 0.09529 3.15934 2.64263
H 0.33878 4.10049 2.12906
H 0.91064 2.89728 3.33758
H -0.83396 3.30150 3.21473
C -1.42662 0.46687 -1.47450
C -3.06085 1.60036 -2.63454
H -3.66156 2.46238 -2.90624
C -3.02633 0.32922 -3.12689
H -3.59226 -0.15915 -3.91419
C -1.72417 2.91279 -0.99429
C -2.49912 3.38252 0.09344
C -2.15917 4.63905 0.63138
H -2.73508 5.03621 1.47142
C -1.09084 5.38071 0.11352
H -0.84148 6.35224 0.55108
C -0.34501 4.89115 -0.96760
H 0.47130 5.49212 -1.37935
C -0.65370 3.65216 -1.55731
C -3.69292 2.59531 0.62009
H -3.55989 1.54081 0.31561
C -3.79473 2.62901 2.15442
H -2.84881 2.31756 2.62480
H -4.58951 1.94678 2.49495
H -4.04030 3.63741 2.52802
C -5.00469 3.10737 -0.01586
H -5.17339 4.16654 0.24364
H -5.86351 2.52234 0.35334

```
H  -4.98744  3.02883 -1.11447
C   0.04588  3.18880 -2.82854
H  -0.18775  2.12118 -2.96408
C   1.57699  3.30942 -2.77587
H   2.01561  2.92314 -3.71127
H   2.00088  2.72897 -1.94209
H   1.90982  4.35580 -2.66692
C  -0.53289  3.94928 -4.04287
H  -0.32935  5.03079 -3.96262
H  -1.62478  3.81651 -4.11724
H  -0.07841  3.58333 -4.97895
C  -1.55976 -1.65664 -2.76131
C  -2.41164 -2.76532 -2.58037
C  -1.95585 -4.00982 -3.05999
H  -2.59506 -4.89167 -2.95177
C  -0.69900 -4.13346 -3.66246
H  -0.36683 -5.10746 -4.03482
C   0.14557 -3.01833 -3.77915
H   1.13102 -3.13286 -4.23866
C  -0.26792 -1.75178 -3.33364
C   1.41526 -0.33090 -2.08628
H   1.06348  0.91354 -1.08759
H   2.27186  0.33392 -2.27917
H   1.85006 -1.28109 -1.74418
C   0.63922 -0.53102 -3.40969
H  -0.00653  0.34990 -3.57958
C   1.63395 -0.58077 -4.58749
H   2.40490 -1.35713 -4.44658
H   2.16096  0.38424 -4.66591
H   1.12030 -0.77507 -5.54359
C  -3.75347 -2.64993 -1.86590
H  -3.86034 -1.61114 -1.50682
C  -3.79673 -3.56423 -0.62325
H  -3.69670 -4.62771 -0.90054
H  -4.75635 -3.44243 -0.09435
C  -4.92814 -2.95052 -2.81974
H  -4.92204 -2.28184 -3.69691
H  -5.89183 -2.82434 -2.29888
H  -4.88008 -3.98732 -3.19434
H  -2.98952 -3.31404  0.08443
```

Int(2^{+}-3^{+})1

```
SCF  =          -2959.27451968
H(0 K)=         -2958.089209
G(298 K)=       -2958.193231
SCF (C6H5F) =   -2959.32006632
Lowest Frequencies = 23.2492cm-1,
24.4876cm-1
SCF [PBE0(C6H5F, D3)] =
-6060.74127433

141
Ir  0.08023 -0.14251 -0.24330
Zn -1.83702  0.24569  1.40621
Zn  0.18364  1.86112  1.26345
N   2.92332 -0.74642  1.02233
N   1.33121 -1.12078  2.44252
N  -1.82348  1.74882 -1.82664
N  -2.24758 -0.33129 -2.25498
C   1.54455 -0.71998  1.12522
C   3.53222 -1.14339  2.21342
H   4.61108 -1.21469  2.30413
C   2.53078 -1.39384  3.10336
H   2.54702 -1.75580  4.12614
C   3.68294 -0.53622 -0.18899
C   4.05974  0.78137 -0.53182
C   4.76960  0.95003 -1.73520
H   5.07124  1.95341 -2.04586
C   5.10177 -0.15009 -2.53703
H   5.65085  0.00506 -3.47092
C   4.75724 -1.44799 -2.13991
H   5.04290 -2.29836 -2.76516
C   4.04046 -1.67380 -0.95097
C   3.79973  1.94490  0.41919
H   2.86344  1.73158  0.96606
C   4.93762  2.03246  1.46315
H   5.04452  1.09779  2.03592
H   4.73714  2.84785  2.17851
H   5.90179  2.23792  0.96748
C   3.62257  3.29941 -0.28165
H   4.52786  3.59966 -0.83620
H   3.43041  4.08136  0.46984
H   2.77278  3.28717 -0.97912
C   3.72301 -3.09230 -0.48255
H   2.87528 -3.03059  0.22470
C   4.93631 -3.68165  0.27466
H   5.80712 -3.75898 -0.39831
H   4.70417 -4.69177  0.65203
H   5.23241 -3.05735  1.13198
C   3.31046 -4.04890 -1.61745
H   2.45769 -3.65699 -2.19348
H   3.02076 -5.02513 -1.19413
H   4.14080 -4.23451 -2.31938
C   0.06492 -1.43606  3.06123
C  -0.57760 -2.63835  2.67350
C  -1.74993 -3.00867  3.35299
H  -2.26901 -3.92833  3.06923
C  -2.24837 -2.22338  4.40129
H  -3.15598 -2.53013  4.92951
C  -1.59227 -1.04428  4.77025
H  -1.99498 -0.43625  5.58482
C  -0.42115 -0.61982  4.11104
C  -0.04829 -3.44414  1.50046
H   1.02677 -3.22155  1.38784
C  -0.18960 -4.96835  1.66166
H  -1.24581 -5.28451  1.68977
H   0.29880 -5.31766  2.58540
H   0.28107 -5.48097  0.80669
C  -0.76309 -2.98383  0.22522
H  -0.25765 -3.30573 -0.69281
H  -0.88240 -1.85562  0.20196
H  -1.80801 -3.32929  0.20041
C   0.28415  0.65904  4.54343
H   0.98797  0.94448  3.73986
C   1.10018  0.43869  5.83626
H   1.61109  1.37066  6.13006
H   1.86621 -0.34361  5.71524
H   0.43910  0.13497  6.66585
C  -0.70969  1.82064  4.73404
H  -1.35754  1.94464  3.85052
```

H -0.16528 2.76414 4.89597
H -1.36385 1.65821 5.60681
C -3.66167 0.05054 2.08926
H -4.40149 0.23835 1.29202
H -3.83538 0.77137 2.90581
H -3.81731 -0.96331 2.48890
C   1.01424 3.39298 2.15365
H   1.41070 4.08137 1.39254
H   1.83634 3.07078 2.81521
H   0.26763 3.93512 2.75519
C -1.41162 0.46805 -1.50320
C -2.86150 1.73482 -2.76356
H -3.28420 2.65375 -3.15664
C -3.13217 0.42596 -3.03141
H -3.84628 -0.04431 -3.70006
C -1.15429 2.96190 -1.43294
C -1.68793 3.72848 -0.36916
C -1.03042 4.93277 -0.05245
H -1.40948 5.55286 0.76405
C   0.10347 5.34490 -0.76409
H   0.59965 6.28321 -0.49818
C   0.59694 4.57059 -1.82204
H   1.46476 4.91873 -2.39052
C -0.02610 3.36265 -2.18755
C -2.96948 3.31426 0.34489
H -3.08081 2.21990 0.22409
C -2.94461 3.62074 1.85187
H -2.04235 3.20897 2.33378
H -3.82708 3.17966 2.34145
H -2.96196 4.70560 2.05016
C -4.19726 3.98204 -0.31433
H -4.12420 5.08068 -0.24288
H -5.12429 3.66496 0.19188
H -4.28507 3.71859 -1.38007
C   0.42691 2.58690 -3.41879
H -0.09477 1.61628 -3.41502
C   1.93364 2.28289 -3.42707
H   2.19217 1.68595 -4.31766
H   2.23216 1.70513 -2.53910
H   2.54245 3.20275 -3.45989
C -0.00242 3.34131 -4.69649
H   0.49535 4.32403 -4.76107
H -1.09122 3.51460 -4.71474
H   0.26852 2.76166 -5.59482
C -2.11349 -1.75563 -2.40509
C -3.18915 -2.58907 -2.03285
C -3.05719 -3.96628 -2.30284
H -3.87454 -4.64409 -2.03766
C -1.89873 -4.47607 -2.90031
H -1.82115 -5.54722 -3.11109
C -0.83188 -3.62317 -3.22313
H   0.07263 -4.03582 -3.67781
C -0.91354 -2.24062 -2.98127
C   1.05315 -1.06982 -1.91993
H   0.86674 1.12038 -0.73419
H   1.94532 -0.47806 -2.17634
H   1.42189 -2.04683 -1.56319
C   0.25395 -1.30142 -3.22962
H -0.16421 -0.33040 -3.55498
C   1.19742 -1.77327 -4.35251
H   1.74920 -2.68690 -4.07289
H   1.95264 -0.99440 -4.54677
H   0.65289 -1.97397 -5.29093
C -4.43647 -2.05189 -1.33789
H -4.27718 -0.97890 -1.13082
C -4.66406 -2.74561 0.02184
H -4.83358 -3.82908 -0.09916
H -5.55004 -2.32072 0.52183
C -5.68119 -2.18773 -2.24010
H -5.54384 -1.67502 -3.20690
H -6.56748 -1.75625 -1.74584
H -5.89739 -3.24758 -2.45784
H -3.80261 -2.60227 0.69436

Int(2^+-3^+)1′
SCF =   -2959.26993943
H(0 K)=   -2958.084699
G(298 K)=   -2958.189153
SCF (C6H5F) =   -2959.31936048
Lowest Frequencies = 19.7332cm-1, 28.3526cm-1
SCF [PBE0(C6H5F, D3)] = -6060.74234403

141
Ir 0.07488 -0.08365 -0.25136
Zn -1.82584 0.38966 1.38280
Zn 0.35206 1.73632 1.48473
N   2.88454 -0.96571 0.94785
N   1.21764 -1.50947 2.22146
N -1.67297 2.07359 -1.64614
N -2.34148 0.07377 -2.15017
C   1.50492 -0.90866 1.00455
C   3.42340 -1.56212 2.09185
H   4.49424 -1.68526 2.21589
C   2.37456 -1.91586 2.88850
H   2.33483 -2.42492 3.84601
C   3.69924 -0.67288 -0.21169
C   4.17933 0.64219 -0.40177
C   4.96840 0.87729 -1.54345
H   5.35080 1.88281 -1.73417
C   5.26836 -0.15644 -2.43967
H   5.88005 0.05094 -3.32315
C   4.80427 -1.45606 -2.20345
H   5.05817 -2.25716 -2.90303
C   4.01209 -1.74845 -1.07805
C   3.90530 1.73805 0.62174
H   2.87615 1.58989 0.99896
C   4.86163 1.61448 1.83008
H   4.75577 0.64781 2.34590
H   4.65109 2.41178 2.56253
H   5.91069 1.71574 1.50330
C   3.98412 3.15613 0.03864
H   5.01408 3.41820 -0.25872
H   3.66996 3.88842 0.79904
H   3.32675 3.27225 -0.83486
C   3.56709 -3.17969 -0.78738
H   2.68381 -3.13139 -0.12591
C   4.68136 -3.94757 -0.03846
H   5.58690 -4.01820 -0.66472

```
H   4.34856 -4.97189  0.19954
H   4.96392 -3.45548  0.90474
C   3.16147 -3.96549 -2.04824
H   2.41849 -3.41687 -2.64607
H   2.72574 -4.93639 -1.75843
H   4.03019 -4.18089 -2.69307
C  -0.11025 -1.83066  2.67742
C  -0.80198 -2.87967  2.01678
C  -2.07126 -3.22716  2.51642
H  -2.63253 -4.03192  2.03548
C  -2.61207 -2.57830  3.63452
H  -3.59559 -2.87352  4.01225
C  -1.90395 -1.54953  4.26593
H  -2.34281 -1.04202  5.12922
C  -0.63934 -1.14297  3.79730
C  -0.15492 -3.66908  0.88234
H   0.46790 -2.97009  0.29701
C   0.78228 -4.75762  1.45378
H   0.21113 -5.48061  2.06108
H   1.57554 -4.32821  2.08651
H   1.26700 -5.31000  0.63083
C  -1.17342 -4.27554 -0.08943
H  -0.64982 -4.70492 -0.95726
H  -1.87452 -3.51549 -0.46628
H  -1.75848 -5.08844  0.37425
C   0.12244 -0.02273  4.49614
H   0.92395  0.31632  3.81374
C   0.78382 -0.53065  5.79642
H   1.35349  0.27998  6.28027
H   1.47497 -1.36811  5.61055
H   0.01788 -0.88289  6.50828
C  -0.77792  1.19024  4.79922
H  -1.31569  1.52983  3.89787
H  -0.17031  2.03064  5.17042
H  -1.53351  0.95709  5.56787
C  -3.68537  0.35788  1.99939
H  -4.35860  0.77599  1.23177
H  -3.77162  0.96735  2.91453
H  -3.99910 -0.66904  2.23442
C   1.28676  3.06470  2.58049
H   1.76552  3.81433  1.93198
H   2.05712  2.58173  3.20562
H   0.57160  3.58294  3.23962
C  -1.39038  0.74024 -1.40918
C  -2.74919  2.22043 -2.52573
H  -3.08936  3.19823 -2.85140
C  -3.17386  0.96274 -2.84089
H  -3.96491  0.60864 -3.49422
C  -0.86431  3.17348 -1.18700
C  -1.29627  3.92478 -0.06731
C  -0.48505  5.00305  0.33521
H  -0.78152  5.60427  1.19875
C   0.69434  5.31573 -0.35283
H   1.30839  6.15918 -0.02292
C   1.08303  4.56450 -1.46901
H   1.98775  4.83926 -2.01949
C   0.31267  3.47362 -1.91349
C  -2.62500  3.63791  0.62213
H  -2.89987  2.59012  0.39907
C  -2.55787  3.79016  2.15166
H  -1.73470  3.19673  2.58369
H  -3.50146  3.44936  2.60638
H  -2.40367  4.83973  2.45336
C  -3.73661  4.54327  0.04497
H  -3.50396  5.60602  0.22891
H  -4.70453  4.31529  0.52172
H  -3.85230  4.40874 -1.04217
C   0.68397  2.72101 -3.18561
H   0.03913  1.82964 -3.24968
C   2.13853  2.22196 -3.18358
H   2.33645  1.62604 -4.09011
H   2.34513  1.58272 -2.31099
H   2.86173  3.05549 -3.17560
C   0.38846  3.59885 -4.42128
H   1.00956  4.51094 -4.41650
H  -0.66848  3.91198 -4.44763
H   0.60597  3.04325 -5.34883
C  -2.36928 -1.34844 -2.37984
C  -3.54026 -2.06967 -2.05865
C  -3.61423 -3.40700 -2.49689
H  -4.51378 -3.99108 -2.28009
C  -2.55452 -3.99641 -3.19403
H  -2.63544 -5.03327 -3.53447
C  -1.37773 -3.27271 -3.43698
H  -0.54733 -3.75295 -3.96070
C  -1.25837 -1.92931 -3.04302
C   0.84829 -1.11539 -1.95109
H   0.93588  1.13397 -0.67417
H   1.85031 -0.71094 -2.17338
H   1.00475 -2.16486 -1.63194
C   0.01704 -1.13206 -3.25945
H  -0.27610 -0.09181 -3.49708
C   0.86178 -1.62955 -4.44711
H   1.27383 -2.63763 -4.27095
H   1.72025 -0.95420 -4.59506
H   0.27725 -1.65945 -5.38242
C  -4.67576 -1.46603 -1.24028
H  -4.36356 -0.45854 -0.91285
C  -4.92311 -2.30353  0.03224
H  -5.27882 -3.31791 -0.21629
H  -5.68907 -1.82300  0.66306
C  -5.96994 -1.32639 -2.06814
H  -5.81952 -0.70481 -2.96665
H  -6.76793 -0.86433 -1.46341
H  -6.33068 -2.31259 -2.40667
H  -4.00005 -2.40067  0.62647
```

TS(2^+-3^+)2

```
SCF  =          -2959.26906942
H(0 K)=         -2958.084484
G(298 K)=       -2958.188040
SCF (C6H5F) =   -2959.31690490
Lowest Frequencies = -83.7970cm-1,
20.7873cm-1
SCF [PBE0(C6H5F, D3)] =
-6060.7394879

141
Ir  0.08965 -0.04992 -0.27391
Zn -1.72923  0.44260  1.34749
```

Zn	0.52397	1.69389	1.59323
N	2.82308	-1.17865	0.88880
N	1.13385	-1.62114	2.17186
N	-1.54074	2.21036	-1.63239
N	-2.37300	0.26731	-2.11818
C	1.45400	-1.01674	0.96522
C	3.32873	-1.84249	2.01095
H	4.38846	-2.04887	2.11848
C	2.26477	-2.13219	2.81279
H	2.19634	-2.65825	3.75936
C	3.63973	-0.90791	-0.27414
C	4.19196	0.38127	-0.44120
C	4.97948	0.59776	-1.58725
H	5.41947	1.58303	-1.75877
C	5.20408	-0.42891	-2.51310
H	5.81471	-0.23610	-3.40060
C	4.66606	-1.70434	-2.30212
H	4.86223	-2.50128	-3.02468
C	3.87571	-1.97846	-1.17051
C	3.99078	1.46611	0.60999
H	2.95980	1.36246	0.99853
C	4.95341	1.26372	1.80209
H	4.80288	0.29090	2.29510
H	4.79430	2.05252	2.55634
H	6.00217	1.31780	1.46342
C	4.13366	2.88977	0.05495
H	5.17224	3.10709	-0.24834
H	3.86218	3.62176	0.83190
H	3.47365	3.05367	-0.80899
C	3.35801	-3.38935	-0.90141
H	2.49522	-3.30884	-0.21623
C	4.44729	-4.23459	-0.20045
H	5.32967	-4.34182	-0.85401
H	4.06389	-5.24371	0.02586
H	4.78437	-3.78025	0.74394
C	2.87737	-4.11848	-2.16930
H	2.15046	-3.51371	-2.73042
H	2.39679	-5.07163	-1.89183
H	3.71562	-4.36344	-2.84332
C	-0.20751	-1.85647	2.64491
C	-0.97294	-2.85852	1.99218
C	-2.25396	-3.12921	2.50727
H	-2.86985	-3.89563	2.03018
C	-2.73641	-2.45653	3.63803
H	-3.73053	-2.69302	4.02935
C	-1.95419	-1.48256	4.26782
H	-2.34482	-0.96048	5.14538
C	-0.67374	-1.15120	3.78251
C	-0.39145	-3.68486	0.85158
H	0.31399	-3.04214	0.29895
C	0.41374	-4.87815	1.41451
H	-0.24112	-5.55505	1.98945
H	1.22902	-4.54496	2.07720
H	0.86256	-5.45573	0.58849
C	-1.45065	-4.15247	-0.15421
H	-0.96104	-4.61294	-1.02640
H	-2.05972	-3.31093	-0.51812
H	-2.12813	-4.91002	0.27590
C	0.17301	-0.10285	4.49464
H	0.98487	0.20013	3.80688
C	0.82262	-0.69062	5.76724
H	1.45424	0.06640	6.26094
H	1.45236	-1.56667	5.54518
H	0.04751	-1.01059	6.48435
C	-0.63727	1.15820	4.85041
H	-1.15885	1.56753	3.96892
H	0.03157	1.93939	5.24444
H	-1.39851	0.95018	5.62057
C	-3.54357	0.60222	2.06042
H	-4.19925	1.09248	1.32161
H	-3.50996	1.21746	2.97402
H	-3.94993	-0.38661	2.31215
C	1.53387	2.91832	2.74002
H	2.03726	3.66763	2.10820
H	2.29507	2.37769	3.32784
H	0.86471	3.44914	3.43589
C	-1.34302	0.85647	-1.41311
C	-2.63817	2.44268	-2.46316
H	-2.91633	3.44462	-2.77366
C	-3.16507	1.22156	-2.76668
H	-4.00420	0.93091	-3.39056
C	-0.64239	3.24692	-1.19496
C	-0.97585	4.00519	-0.04613
C	-0.06255	4.99859	0.35482
H	-0.27887	5.59998	1.24171
C	1.11665	5.22909	-0.36662
H	1.81119	6.00793	-0.03756
C	1.40238	4.47930	-1.51396
H	2.31014	4.68936	-2.08781
C	0.53004	3.46577	-1.95494
C	-2.30661	3.81758	0.67324
H	-2.66992	2.79839	0.44587
C	-2.19670	3.94605	2.20212
H	-1.42603	3.27277	2.61322
H	-3.15986	3.69033	2.67156
H	-1.93953	4.97304	2.51119
C	-3.35230	4.81897	0.13138
H	-3.03105	5.85629	0.32664
H	-4.32650	4.66196	0.62381
H	-3.49877	4.71299	-0.95513
C	0.82632	2.68458	-3.22957
H	0.02455	1.93948	-3.36416
C	2.15314	1.91002	-3.13262
H	2.30336	1.29124	-4.03308
H	2.16251	1.24292	-2.25648
H	3.01735	2.59113	-3.05226
C	0.80213	3.61865	-4.45773
H	1.59559	4.38292	-4.39530
H	-0.16382	4.14292	-4.54524
H	0.96511	3.03930	-5.38180
C	-2.55223	-1.14395	-2.35288
C	-3.77798	-1.74585	-1.99359
C	-4.00617	-3.06280	-2.44126
H	-4.95129	-3.55464	-2.19191
C	-3.04401	-3.74684	-3.19052
H	-3.24594	-4.76381	-3.54042
C	-1.80848	-3.14474	-3.47071
H	-1.05132	-3.70140	-4.02848
C	-1.53381	-1.83002	-3.06001
C	0.60986	-1.28806	-1.92854

H   0.78705 1.34262 -0.38537
H   1.67451 -1.05081 -2.10976
H   0.59119 -2.34408 -1.59365
C -0.17491 -1.18472 -3.26108
H -0.33282 -0.11350 -3.48822
C   0.62411 -1.77093 -4.43848
H   0.88724 -2.82983 -4.27652
H   1.57089 -1.21783 -4.55248
H   0.06513 -1.70138 -5.38730
C -4.81202 -1.04826 -1.11737
H -4.38854 -0.08203 -0.79050
C -5.08250 -1.88334 0.15219
H -5.55056 -2.85101 -0.09593
H -5.76586 -1.34259 0.82768
C -6.12303 -0.76812 -1.88054
H -5.95541 -0.14432 -2.77439
H -6.84454 -0.24523 -1.23082
H -6.59216 -1.70793 -2.21812
H -4.14661 -2.08670 0.69752

Int(2^+-3^+)2

SCF =   -2959.28224294
H(0 K)=   -2958.097106
G(298 K)=   -2958.203136
SCF (C6H5F) =   -2959.32816558
Lowest Frequencies = 18.4599cm-1, 26.2936cm-1
SCF [PBE0(C6H5F, D3)] = -6060.75316081

141
Ir 0.07765 0.04300 -0.13101
Zn -1.97031 -0.46854 1.14978
Zn 0.69798 2.02851 1.18194
N   2.89082 -1.04977 0.87903
N   1.26444 -1.55223 2.21514
N -1.78424 2.18708 -1.40522
N -2.28218 0.20442 -2.12737
C   1.51684 -0.91001 1.01308
C   3.45225 -1.73602 1.95876
H   4.51766 -1.93512 2.01253
C   2.42766 -2.06219 2.79674
H   2.40358 -2.61373 3.73138
C   3.64796 -0.75834 -0.31821
C   4.13455 0.55222 -0.52298
C   4.78059 0.81322 -1.74527
H   5.15597 1.81843 -1.95225
C   4.95867 -0.20049 -2.69647
H   5.45894 0.02519 -3.64332
C   4.52984 -1.50664 -2.42974
H   4.70520 -2.29461 -3.16763
C   3.86394 -1.81915 -1.23094
C   4.06007 1.59185 0.58859
H   3.12596 1.40866 1.15234
C   5.23503 1.38304 1.57273
H   5.23739 0.36712 1.99825
H   5.16924 2.10171 2.40701
H   6.19963 1.53766 1.05977
C   4.03307 3.04750 0.09950
H   4.94934 3.30997 -0.45594
H   3.97432 3.72624 0.96444
H   3.16568 3.24723 -0.54768
C   3.46152 -3.25812 -0.91324
H   2.71584 -3.23317 -0.09954
C   4.69490 -4.03697 -0.39865
H   5.46396 -4.10733 -1.18664
H   4.41076 -5.06113 -0.10406
H   5.15776 -3.54569 0.47217
C   2.82290 -4.01249 -2.09466
H   1.91858 -3.50388 -2.46331
H   2.53545 -5.02786 -1.77379
H   3.52311 -4.12526 -2.93950
C -0.03654 -1.90911 2.71781
C -0.69569 -3.01820 2.12371
C -1.89461 -3.44850 2.72527
H -2.42656 -4.30519 2.30155
C -2.40107 -2.80902 3.86564
H -3.32570 -3.17022 4.32584
C -1.74422 -1.69601 4.40581
H -2.16627 -1.18841 5.27782
C -0.54835 -1.21443 3.83769
C -0.10338 -3.75495 0.92922
H   0.65898 -3.09960 0.47674
C   0.59920 -5.05034 1.38860
H -0.11915 -5.75148 1.84699
H   1.38825 -4.83906 2.12946
H   1.06602 -5.55632 0.52654
C -1.14678 -4.02163 -0.16509
H -0.66884 -4.49415 -1.03725
H -1.60165 -3.07898 -0.51053
H -1.95431 -4.69202 0.17467
C   0.16593 0.00562 4.40244
H   0.90649 0.33132 3.64904
C   0.92633 -0.33999 5.70000
H   1.44807 0.55008 6.08932
H   1.67756 -1.12921 5.53374
H   0.23039 -0.69578 6.47892
C -0.81277 1.17195 4.62348
H -1.39002 1.37421 3.70763
H -0.26165 2.08695 4.89591
H -1.52878 0.96104 5.43563
C -3.76081 -0.34800 1.89972
H -4.45260 0.09981 1.16857
H -3.69824 0.30778 2.78408
H -4.13936 -1.33021 2.21137
C   1.48428 3.30536 2.42155
H   1.88313 4.17431 1.88023
H   2.29188 2.82400 2.99717
H   0.70142 3.64892 3.11654
C -1.40283 0.86427 -1.28251
C -2.82363 2.34618 -2.32694
H -3.20851 3.32671 -2.58896
C -3.13891 1.09844 -2.77758
H -3.86851 0.75701 -3.50470
C -1.05800 3.30824 -0.86615
C -1.52085 3.91802 0.32706
C -0.83736 5.06978 0.76195
H -1.16056 5.57243 1.67672
C   0.26501 5.56812 0.05334
H   0.78352 6.46042 0.41713

```
C   0.71059  4.92890 -1.11022
H   1.57026  5.32921 -1.65655
C   0.04617  3.79174 -1.60915
C  -2.71486  3.35205  1.09060
H  -2.69267  2.25368  0.95697
C  -2.64708  3.64662  2.59889
H  -1.65959  3.40195  3.01958
H  -3.40716  3.05261  3.13203
H  -2.85486  4.70875  2.81451
C  -4.05443  3.86712  0.51731
H  -4.09893  4.96833  0.56957
H  -4.89681  3.46244  1.10300
H  -4.19990  3.56583 -0.53092
C   0.46212  3.16071 -2.93188
H  -0.17804  2.27991 -3.10208
C   1.91561  2.65827 -2.90525
H   2.15826  2.14176 -3.84897
H   2.07154  1.94536 -2.08088
H   2.63254  3.48883 -2.78786
C   0.22075  4.14263 -4.09789
H   0.84866  5.04447 -3.99782
H  -0.83178  4.46874 -4.13781
H   0.46916  3.66301 -5.05930
C  -2.23199 -1.19185 -2.48997
C  -3.36862 -2.00613 -2.29156
C  -3.32394 -3.31498 -2.81393
H  -4.19221 -3.96834 -2.68737
C  -2.19151 -3.79067 -3.48098
H  -2.18226 -4.80825 -3.88366
C  -1.06311 -2.97069 -3.62662
H  -0.17815 -3.35263 -4.14213
C  -1.06266 -1.65236 -3.14370
C   0.89258 -0.78892 -1.87322
H  -0.61252  0.74811  1.34403
H   1.84951 -0.24631 -1.99457
H   1.17126 -1.83610 -1.65951
C   0.15641 -0.75812 -3.23214
H  -0.19206  0.27470 -3.42119
C   1.11882 -1.12131 -4.37733
H   1.60600 -2.09714 -4.21290
H   1.92554 -0.37190 -4.42825
H   0.60624 -1.15058 -5.35394
C  -4.60098 -1.54095 -1.52483
H  -4.36580 -0.56668 -1.06011
C  -4.94185 -2.52906 -0.38914
H  -5.26918 -3.50316 -0.78962
H  -5.76154 -2.13085  0.23102
C  -5.81591 -1.35159 -2.45784
H  -5.61394 -0.62347 -3.26085
H  -6.69042 -0.99585 -1.88783
H  -6.09054 -2.30499 -2.94046
H  -4.07043 -2.70467  0.26182
```

Int(2⁺-3⁺)2′

```
SCF  =           -2959.28288955
H(0 K)=          -2958.097469
G(298 K)=        -2958.203521
SCF (C6H5F) =    -2959.32845967
Lowest Frequencies = 15.7725cm-1,
24.3696cm-1
SCF [PBE0(C6H5F, D3)] =
-6060.75322586

141
Ir  0.06427  0.04160 -0.12018
Zn -1.81777 -1.00378  1.10433
Zn -0.01556  2.15162  1.10988
N   3.04918 -0.18635  0.98148
N   1.60569 -1.15571  2.26760
N  -2.32653  1.53368 -1.46739
N  -2.10512 -0.48030 -2.23813
C   1.69199 -0.46021  1.07104
C   3.75717 -0.69180  2.07386
H   4.83299 -0.57497  2.15583
C   2.84940 -1.30932  2.88201
H   2.96192 -1.85818  3.81173
C   3.72539  0.36597 -0.17134
C   3.80202  1.76984 -0.31716
C   4.38337  2.26005 -1.50089
H   4.44457  3.33875 -1.66426
C   4.89596  1.38475 -2.46844
H   5.34007  1.78759 -3.38398
C   4.87257  0.00118 -2.25637
H   5.30626 -0.66846 -3.00460
C   4.28569 -0.54325 -1.09987
C   3.36948  2.69746  0.81264
H   2.48393  2.24201  1.29581
C   4.48461  2.77738  1.88190
H   4.73138  1.78695  2.29452
H   4.16765  3.42369  2.71779
H   5.40461  3.20367  1.44690
C   2.98159  4.11277  0.35864
H   3.83629  4.64367 -0.09378
H   2.65860  4.70379  1.22949
H   2.15628  4.10175 -0.36924
C   4.32085 -2.04840 -0.84163
H   3.58208 -2.27593 -0.05303
C   5.71749 -2.44743 -0.31073
H   6.49087 -2.25508 -1.07377
H   5.74455 -3.52078 -0.05847
H   5.99284 -1.87583  0.59029
C   3.95656 -2.90885 -2.06637
H   2.94317 -2.68546 -2.43478
H   3.98944 -3.97657 -1.79200
H   4.66618 -2.76454 -2.89848
C   0.45183 -1.87203  2.74701
C   0.19910 -3.15681  2.20565
C  -0.86905 -3.89007  2.75988
H  -1.09578 -4.88475  2.36402
C  -1.62365 -3.37371  3.82137
H  -2.43840 -3.96583  4.24885
C  -1.35188 -2.09603  4.33011
H  -1.96050 -1.69879  5.14717
C  -0.31049 -1.31128  3.79877
C   1.04962 -3.74282  1.08748
H   1.85004 -3.02061  0.85564
C   1.72348 -5.05941  1.52361
H   0.97820 -5.84466  1.73629
H   2.33431 -4.91868  2.43060
H   2.38016 -5.43248  0.72000
```

C   0.22236 -3.92183 -0.19603
H   0.85191 -4.30127 -1.01619
H -0.20518 -2.95806 -0.52339
H -0.60737 -4.63366 -0.05008
C -0.00483 0.08059 4.33499
H   0.61172 0.59609 3.57585
C   0.81406 0.00645 5.64134
H   1.04000 1.02030 6.01182
H   1.76966 -0.52213 5.49287
H   0.25089 -0.52703 6.42606
C -1.28684 0.90720 4.52833
H -1.89274 0.90600 3.60857
H -1.03393 1.95111 4.77564
H -1.91271 0.51487 5.34748
C -3.58877 -1.50843 1.72457
H -4.34206 -1.31416 0.94437
H -3.81769 -0.88873 2.60772
H -3.62507 -2.56675 2.01578
C   0.23679 3.61311 2.36965
H   0.38104 4.56365 1.83817
H   1.11170 3.40647 3.00751
H -0.66234 3.69065 3.00083
C -1.54243 0.40235 -1.33052
C -3.30312 1.37201 -2.45643
H -3.97290 2.18031 -2.73271
C -3.16592 0.10349 -2.93722
H -3.70049 -0.43680 -3.71191
C -2.04630 2.81950 -0.88077
C -2.75704 3.21762 0.28030
C -2.54090 4.52957 0.74294
H -3.06981 4.87878 1.63296
C -1.64235 5.38807 0.09426
H -1.48847 6.40090 0.47885
C -0.93355 4.95531 -1.03290
H -0.23375 5.63375 -1.53055
C -1.13336 3.66457 -1.55948
C -3.71488 2.26279 0.98711
H -3.28755 1.24628 0.88686
C -3.85837 2.57014 2.48764
H -2.88080 2.71048 2.97412
H -4.37966 1.74026 2.99197
H -4.45745 3.48112 2.65828
C -5.11056 2.24811 0.32370
H -5.55478 3.25801 0.33697
H -5.78323 1.57140 0.87680
H -5.07098 1.90144 -0.71969
C -0.44878 3.23488 -2.85046
H -0.70647 2.17978 -3.03460
C   1.08438 3.30813 -2.75189
H   1.54414 2.93582 -3.68257
H   1.45594 2.68653 -1.92253
H   1.43641 4.34281 -2.59880
C -0.98221 4.05975 -4.04109
H -0.74081 5.13022 -3.92535
H -2.07729 3.97015 -4.13331
H -0.52812 3.70983 -4.98311
C -1.56531 -1.76590 -2.60840
C -2.35329 -2.92535 -2.44851
C -1.83445 -4.13211 -2.96009
H -2.42289 -5.04930 -2.86280
C -0.58452 -4.17488 -3.58502
H -0.20562 -5.12166 -3.98236
C   0.18998 -3.01059 -3.69661
H   1.16898 -3.05479 -4.18076
C -0.28556 -1.78048 -3.21521
C   1.19810 -0.32439 -1.85089
H -0.74945 0.44119 1.39701
H   1.88183 0.54358 -1.90754
H   1.84113 -1.19964 -1.65878
C   0.55101 -0.51725 -3.24275
H -0.12737 0.33387 -3.44086
C   1.62570 -0.49973 -4.34441
H   2.41327 -1.25009 -4.16204
H   2.12456 0.48316 -4.35297
H   1.19650 -0.68282 -5.34425
C -3.70269 -2.91538 -1.74045
H -3.83472 -1.92267 -1.27389
C -3.74190 -3.96671 -0.61195
H -3.67857 -4.99178 -1.01420
H -4.68610 -3.88727 -0.04864
C -4.86392 -3.14050 -2.73224
H -4.87852 -2.38081 -3.53131
H -5.83392 -3.10352 -2.20876
H -4.77775 -4.12723 -3.21832
H -2.90772 -3.82451 0.09368

TS(2^+-3^+)3

SCF =   -2959.27491889
H(0 K)=   -2958.089687
G(298 K)=   -2958.193912
SCF (C6H5F) =   -2959.32109888
Lowest Frequencies = -8.8416cm-1, 18.5591cm-1
SCF [PBE0(C6H5F, D3)] =   -6060.74397719

141
Ir -0.03156 0.03932 -0.20747
Zn -1.96759 -1.00315 0.75786
Zn 1.05336 1.32737 1.68321
N   2.73271 -1.44381 0.14668
N   1.22757 -2.25159 1.47953
N -1.11491 2.88103 -0.78587
N -2.44372 1.42895 -1.67823
C   1.37785 -1.33322 0.45102
C   3.37783 -2.36328 0.97916
H   4.43977 -2.56297 0.88283
C   2.43281 -2.87751 1.81198
H   2.48404 -3.63063 2.59203
C   3.43495 -0.89513 -0.99617
C   4.22961 0.26039 -0.82938
C   4.94384 0.72350 -1.95199
H   5.57173 1.61388 -1.85810
C   4.86605 0.06065 -3.18123
H   5.41741 0.44556 -4.04468
C   4.11620 -1.11652 -3.30029
H   4.10025 -1.65365 -4.25244
C   3.40905 -1.64055 -2.20308
C   4.40965 0.92114 0.53082
H   3.55004 0.63303 1.16220

C	5.69057	0.38607	1.21158
H	5.67357	-0.71001	1.31985
H	5.80340	0.82602	2.21673
H	6.58288	0.64985	0.61837
C	4.44811	2.45629	0.46627
H	5.29145	2.82168	-0.14357
H	4.57722	2.87107	1.47844
H	3.51619	2.86831	0.05205
C	2.76403	-3.02105	-2.28593
H	1.95418	-3.06244	-1.53824
C	3.80530	-4.10663	-1.92239
H	4.65020	-4.08080	-2.63138
H	3.34596	-5.10810	-1.97274
H	4.20916	-3.97252	-0.90756
C	2.15381	-3.33892	-3.66110
H	1.48886	-2.53567	-4.00653
H	1.56859	-4.27205	-3.60212
H	2.93289	-3.49716	-4.42630
C	0.00173	-2.75010	2.05450
C	-0.68152	-3.78420	1.36112
C	-1.70373	-4.45694	2.05375
H	-2.24246	-5.26865	1.55708
C	-2.03562	-4.10767	3.37038
H	-2.82341	-4.65583	3.89613
C	-1.38384	-3.04381	4.00616
H	-1.67092	-2.76204	5.02353
C	-0.34639	-2.34287	3.36210
C	-0.29600	-4.17927	-0.05808
H	0.24776	-3.32501	-0.49559
C	0.65441	-5.39539	-0.05652
H	0.16381	-6.27725	0.38994
H	1.57391	-5.18835	0.51474
H	0.94627	-5.65359	-1.08866
C	-1.52542	-4.42558	-0.94915
H	-1.20316	-4.58835	-1.99125
H	-2.20993	-3.56159	-0.93866
H	-2.09301	-5.31902	-0.63804
C	0.38671	-1.21031	4.06428
H	1.02834	-0.71335	3.31344
C	1.30754	-1.73921	5.18305
H	1.84822	-0.90538	5.66103
H	2.05399	-2.44866	4.78983
H	0.72457	-2.25976	5.96198
C	-0.60225	-0.16047	4.60084
H	-1.24946	0.22003	3.79333
H	-0.05433	0.69009	5.03850
H	-1.25266	-0.57566	5.38878
C	-3.65475	-1.58313	1.51188
H	-4.08022	-0.76279	2.11159
H	-3.48055	-2.45269	2.15853
H	-4.34162	-1.84645	0.69463
C	2.23488	1.95802	3.09741
H	2.75853	2.87217	2.78379
H	2.97263	1.18767	3.37559
H	1.62220	2.19544	3.98326
C	-1.30540	1.51469	-0.89104
C	-2.05342	3.60125	-1.53132
H	-2.02606	4.68552	-1.58345
C	-2.88757	2.68471	-2.09639
H	-3.74138	2.80064	-2.75512

C	-0.12677	3.57429	0.00205
C	-0.43350	3.86047	1.35716
C	0.48486	4.66239	2.06401
H	0.29399	4.89990	3.11331
C	1.63389	5.16361	1.44015
H	2.32787	5.79203	2.00657
C	1.89786	4.87535	0.09383
H	2.78886	5.29227	-0.38200
C	1.02004	4.07547	-0.66038
C	-1.73263	3.37501	1.99855
H	-1.97676	2.39656	1.54524
C	-1.60683	3.17228	3.51826
H	-0.72517	2.56648	3.77885
H	-2.50200	2.65853	3.90475
H	-1.52869	4.13464	4.05243
C	-2.89721	4.34549	1.69297
H	-2.67576	5.35064	2.09053
H	-3.82469	3.98628	2.16989
H	-3.08644	4.43666	0.61245
C	1.25631	3.75004	-2.13218
H	0.27050	3.78340	-2.63197
C	1.80395	2.31632	-2.29055
H	1.73830	1.97691	-3.33772
H	1.24216	1.59296	-1.66745
H	2.85810	2.26206	-1.98404
C	2.17214	4.75734	-2.84666
H	3.20618	4.70736	-2.46364
H	1.81189	5.79331	-2.73454
H	2.21458	4.52469	-3.92305
C	-3.10094	0.19835	-2.02546
C	-4.45083	-0.01098	-1.64792
C	-5.01289	-1.26071	-1.97517
H	-6.05174	-1.46540	-1.70051
C	-4.27134	-2.23792	-2.64983
H	-4.73028	-3.20346	-2.88394
C	-2.95631	-1.97344	-3.05784
H	-2.40279	-2.72614	-3.62492
C	-2.34887	-0.73991	-2.76926
C	0.08145	-0.82932	-2.13960
H	-0.47743	0.51254	1.40106
H	1.10237	-0.61725	-2.51288
H	0.02222	-1.92931	-2.03368
C	-0.93802	-0.40020	-3.21682
H	-0.87823	0.69927	-3.32166
C	-0.60707	-0.99345	-4.59989
H	-0.57371	-2.09581	-4.58018
H	0.38505	-0.64103	-4.92840
H	-1.34734	-0.69125	-5.36013
C	-5.31783	1.07575	-1.01473
H	-4.65314	1.86913	-0.62924
C	-6.17668	0.58835	0.16728
H	-6.89633	-0.18879	-0.14073
H	-6.76202	1.43086	0.57169
C	-6.23490	1.69348	-2.09861
H	-5.67380	2.03663	-2.98416
H	-6.79792	2.54973	-1.69099
H	-6.96315	0.94455	-2.45377
H	-5.55919	0.17632	0.97710

Int(2$^+$-3$^+$)3

```
SCF  =          -2959.27496655
H(0 K)=         -2958.089326
G(298 K)=       -2958.195017
SCF (C6H5F) =   -2959.32098854
Lowest Frequencies = 12.0100cm-1, 23.5725cm-1
SCF [PBE0(C6H5F, D3)] = -6060.74355453

141
Ir -0.03715  0.04108 -0.21031
Zn -1.98318 -1.03876  0.69328
Zn  1.02653  1.32019  1.70363
N   2.72529 -1.44187  0.16544
N   1.21158 -2.25283  1.48673
N  -1.08155  2.89153 -0.81323
N  -2.41651  1.44887 -1.70855
C   1.36750 -1.33355  0.45928
C   3.36512 -2.36203  1.00142
H   4.42847 -2.55835  0.91384
C   2.41462 -2.87887  1.82608
H   2.46053 -3.63408  2.60446
C   3.44682 -0.87876 -0.95854
C   4.25223  0.26376 -0.75634
C   4.99623  0.73658 -1.85539
H   5.63328  1.61689 -1.73236
C   4.93515  0.09684 -3.09751
H   5.50921  0.48909 -3.94266
C   4.17171 -1.06732 -3.25255
H   4.16837 -1.58739 -4.21419
C   3.43700 -1.60259 -2.17905
C   4.40916  0.90569  0.61563
H   3.54209  0.60469  1.23047
C   5.68381  0.37097  1.30793
H   5.67356 -0.72653  1.40200
H   5.77895  0.79881  2.32012
H   6.58277  0.64878  0.73140
C   4.43755  2.44181  0.56907
H   5.28701  2.81981 -0.02435
H   4.54768  2.84665  1.58744
H   3.50905  2.85106  0.14458
C   2.78098 -2.97505 -2.29922
H   1.94431 -3.01277 -1.58175
C   3.79416 -4.07790 -1.90939
H   4.66706 -4.05340 -2.58368
H   3.32481 -5.07261 -1.99253
H   4.15751 -3.96353 -0.87716
C   2.21674 -3.27238 -3.69819
H   1.57426 -2.45822 -4.06023
H   1.61795 -4.19817 -3.66882
H   3.02094 -3.43427 -4.43619
C  -0.01737 -2.77319  2.03560
C  -0.64607 -3.83986  1.34199
C  -1.66725 -4.53386  2.01493
H  -2.16146 -5.37335  1.51722
C  -2.05395 -4.16953  3.31210
H  -2.84171 -4.73137  3.82317
C  -1.45569 -3.07402  3.94732
H  -1.78331 -2.78369  4.94987
C  -0.41706 -2.35562  3.32511
C  -0.21374 -4.24548 -0.05985
H   0.41094 -3.42897 -0.45837
C   0.64696 -5.52530 -0.02966
H   0.07481 -6.37809  0.37422
H   1.54496 -5.38971  0.59498
H   0.97702 -5.78910 -1.04869
C  -1.41574 -4.38858 -1.01035
H  -1.06175 -4.58663 -2.03623
H  -2.02019 -3.46675 -1.03390
H  -2.07674 -5.22307 -0.72098
C   0.27229 -1.20090  4.03509
H   0.91522 -0.69207  3.29330
C   1.18790 -1.70388  5.17013
H   1.70185 -0.85573  5.65244
H   1.95633 -2.39710  4.79043
H   0.60526 -2.23514  5.94204
C  -0.74906 -0.17255  4.55116
H  -1.38528  0.19854  3.73046
H  -0.22780  0.68713  5.00324
H  -1.40842 -0.60204  5.32383
C  -3.66516 -1.70430  1.39021
H  -4.06196 -0.99233  2.13126
H  -3.48879 -2.67187  1.87695
H  -4.37540 -1.81918  0.55954
C   2.18051  1.94352  3.14400
H   2.69403  2.87111  2.85399
H   2.92619  1.18007  3.41989
H   1.55205  2.15541  4.02547
C  -1.28795  1.52657 -0.90892
C  -2.00182  3.61669 -1.57693
H  -1.96066  4.70010 -1.63731
C  -2.84078  2.70542 -2.14387
H  -3.68690  2.82382 -2.81263
C  -0.10220  3.57965 -0.01020
C  -0.43073  3.87123  1.33857
C   0.47954  4.67057  2.05865
H   0.27217  4.91204  3.10394
C   1.64103  5.16494  1.45261
H   2.32800  5.79238  2.02859
C   1.92695  4.87066  0.11201
H   2.82830  5.28123 -0.34958
C   1.05888  4.07146 -0.65405
C  -1.74354  3.39451  1.95840
H  -1.98579  2.41676  1.50259
C  -1.64526  3.19304  3.48027
H  -0.77377  2.57932  3.75629
H  -2.55149  2.68808  3.85227
H  -1.56712  4.15533  4.01452
C  -2.89675  4.37149  1.63113
H  -2.67787  5.37505  2.03410
H  -3.83540  4.01670  2.08911
H  -3.06384  4.46502  0.54714
C   1.31958  3.73540 -2.11927
H   0.34541  3.78620 -2.64023
C   1.84255  2.29060 -2.25990
H   1.79943  1.95003 -3.30785
H   1.24946  1.57898 -1.65187
H   2.88630  2.21675 -1.92386
C   2.27100  4.72039 -2.81798
```

```
H   3.29478  4.65193 -2.41100
H   1.92895  5.76390 -2.71954
H   2.33316  4.48097 -3.89190
C  -3.09605  0.22709 -2.03840
C  -4.44675  0.05215 -1.65107
C  -5.04312 -1.18228 -1.97636
H  -6.08624 -1.36000 -1.69796
C  -4.32737 -2.18018 -2.64860
H  -4.81168 -3.13382 -2.88022
C  -3.00411 -1.95374 -3.05491
H  -2.47057 -2.72379 -3.61786
C  -2.36543 -0.73413 -2.77388
C   0.06769 -0.84797 -2.13666
H  -0.50230  0.51823  1.38587
H   1.08965 -0.64267 -2.51153
H   0.00495 -1.94657 -2.02036
C  -0.94745 -0.42603 -3.22240
H  -0.87245  0.67047 -3.34779
C  -0.62399 -1.05155 -4.59277
H  -0.58687 -2.15305 -4.54491
H   0.36445 -0.70419 -4.93712
H  -1.36972 -0.76971 -5.35553
C  -5.27405  1.15353 -0.99075
H  -4.58742  1.95274 -0.66107
C  -6.04898  0.68621  0.25570
H  -6.75964 -0.12377  0.01996
H  -6.63385  1.52625  0.66609
C  -6.25138  1.75693 -2.02713
H  -5.73568  2.09747 -2.94106
H  -6.79503  2.61420 -1.59614
H  -6.99531  1.00443 -2.33968
H  -5.36975  0.32289  1.03974
```

TS(2⁺-3⁺)4

```
SCF  =           -2959.26713784
H(0 K)=          -2958.081605
G(298 K)=        -2958.185835
SCF (C6H5F) =    -2959.31322906
Lowest Frequencies = -151.7481cm-1,  12.9800cm-1
SCF [PBE0(C6H5F, D3)] = -6060.73652394

141
Ir  0.06215  0.14752 -0.20483
Zn -2.18413 -0.27386  0.49263
Zn  1.07302  0.23925  2.21558
N   2.04387 -2.25042  0.15341
N   0.16123 -2.74367  1.11596
N   0.16511  3.19167 -0.74587
N  -1.64335  2.38493 -1.60990
C   0.76019 -1.74699  0.34485
C   2.23728 -3.46362  0.81984
H   3.19630 -3.97184  0.79677
C   1.05635 -3.77577  1.42010
H   0.75029 -4.62628  2.02135
C   3.08548 -1.73930 -0.71568
C   4.13618 -0.98862 -0.14776
C   5.20706 -0.61838 -0.98458
H   6.03567 -0.03898 -0.56575
C   5.22569 -0.98588 -2.33330
H   6.06311 -0.68745 -2.97137
C   4.17934 -1.75000 -2.86638
H   4.21530 -2.05361 -3.91587
C   3.09869 -2.16773 -2.06841
C   4.15762 -0.59864  1.31977
H   3.23697 -0.98944  1.78783
C   5.34698 -1.24045  2.06159
H   5.33803 -2.33853  1.96228
H   5.30552 -0.99098  3.13491
H   6.31050 -0.87560  1.66676
C   4.15217  0.93179  1.46795
H   5.02269  1.38654  0.96733
H   4.18315  1.23107  2.52725
H   3.25181  1.37422  1.00873
C   2.06114 -3.13702 -2.62503
H   1.12414 -2.98403 -2.06445
C   2.51588 -4.59881 -2.40386
H   3.48077 -4.78355 -2.90591
H   1.77238 -5.29634 -2.82505
H   2.63314 -4.83835 -1.33597
C   1.75845 -2.90858 -4.11634
H   1.56537 -1.84722 -4.33529
H   0.86711 -3.48594 -4.41126
H   2.59076 -3.24539 -4.75765
C  -1.24520 -2.96588  1.39490
C  -1.99017 -3.69829  0.43379
C  -3.27700 -4.13329  0.79900
H  -3.86934 -4.70676  0.08024
C  -3.79675 -3.87205  2.07300
H  -4.79281 -4.23613  2.34223
C  -3.04830 -3.13469  2.99613
H  -3.46378 -2.92062  3.98543
C  -1.75892 -2.66889  2.67982
C  -1.41482 -4.07971 -0.92219
H  -0.51994 -3.45843 -1.09167
C  -0.96650 -5.55701 -0.93451
H  -1.82062 -6.22866 -0.74243
H  -0.19925 -5.75232 -0.16815
H  -0.54019 -5.81982 -1.91716
C  -2.40321 -3.78938 -2.06674
H  -1.90432 -3.92714 -3.04102
H  -2.78218 -2.75635 -2.01223
H  -3.26988 -4.47153 -2.04226
C  -0.95669 -1.90356  3.71629
H   0.01496 -1.65016  3.25850
C  -0.65520 -2.77042  4.95504
H  -0.01403 -2.21809  5.66166
H  -0.13480 -3.70089  4.67415
H  -1.58179 -3.04959  5.48497
C  -1.65555 -0.58503  4.10541
H  -1.78849  0.07193  3.22733
H  -1.06308 -0.03806  4.85684
H  -2.65464 -0.77035  4.53388
C  -4.01913 -0.53358  1.07818
H  -4.14127 -0.17635  2.11253
H  -4.23642 -1.60947  1.04020
H  -4.69938  0.00577  0.40827
C   1.91829 -0.14289  3.92890
H   2.77469  0.53210  4.08511
```

```
H   2.28114 -1.18358  3.96995
H   1.20617  0.00785  4.75586
C  -0.54353  2.00736 -0.86165
C  -0.44771  4.23709 -1.44060
H  -0.00796  5.22939 -1.47221
C  -1.58497  3.72697 -1.99097
H  -2.35335  4.17556 -2.61277
C   1.33741  3.44570  0.05637
C   1.15240  3.72731  1.43041
C   2.29583  4.09604  2.16454
H   2.20192  4.31811  3.23111
C   3.54435  4.21707  1.53920
H   4.41492  4.53037  2.12360
C   3.68888  3.93626  0.17357
H   4.67076  4.03083 -0.29682
C   2.58661  3.52570 -0.59798
C  -0.23580  3.69834  2.06642
H  -0.84969  2.97961  1.49493
C  -0.22126  3.22190  3.52950
H   0.30498  2.25728  3.64374
H  -1.25357  3.08952  3.89274
H   0.27086  3.94891  4.19762
C  -0.91097  5.08434  1.95478
H  -0.32626  5.84801  2.49538
H  -1.92354  5.05422  2.39161
H  -1.00305  5.40552  0.90480
C   2.70250  3.14033 -2.06926
H   1.81560  3.54751 -2.59003
C   2.66155  1.60299 -2.21082
H   2.58724  1.30368 -3.26972
H   1.79632  1.16521 -1.67571
H   3.57093  1.15188 -1.78777
C   3.95078  3.70952 -2.76217
H   4.87594  3.26115 -2.36022
H   4.02067  4.80412 -2.64946
H   3.91993  3.47833 -3.83928
C  -2.78240  1.55809 -1.89063
C  -4.05195  1.95964 -1.41349
C  -5.14736  1.14616 -1.76661
H  -6.14914  1.42339 -1.42395
C  -4.97261 -0.01482 -2.52807
H  -5.83864 -0.63374 -2.78186
C  -3.69635 -0.38406 -2.98045
H  -3.57976 -1.27197 -3.60661
C  -2.57308  0.40757 -2.68546
C  -0.34351 -0.66858 -2.13014
H   0.46815  1.24107  1.04164
H   0.67841 -0.79067 -2.54285
H  -0.76214 -1.68504 -2.05196
C  -1.17541  0.09676 -3.18759
H  -0.67733  1.06854 -3.36766
C  -1.18967 -0.66137 -4.52881
H  -1.53230 -1.70323 -4.40357
H  -0.17232 -0.70348 -4.94813
H  -1.84243 -0.16992 -5.27003
C  -4.26866  3.20137 -0.55027
H  -3.28833  3.67095 -0.36305
C  -4.85967  2.85294  0.83100
H  -5.84057  2.35578  0.74354
H  -5.00229  3.77389  1.42104
C  -5.14931  4.23342 -1.28649
H  -4.72454  4.50917 -2.26672
H  -5.25301  5.15189 -0.68518
H  -6.16162  3.83455 -1.46879
H  -4.19123  2.18081  1.39158
```

Int(2⁺-3⁺)4

```
SCF  =            -2959.28087920
H(0 K)=           -2958.094077
G(298 K)=         -2958.196759
SCF (C6H5F) =    -2959.32901018
Lowest Frequencies = 15.6386cm-1,
26.5386cm-1
SCF [PBE0(C6H5F, D3)] =
-6060.75235272

141
Ir  0.10544  0.00866 -0.14336
Zn -2.27150  0.47722 -0.02123
Zn -0.52114  0.82795  2.11515
N   0.85994 -2.93683 -0.17900
N  -1.21511 -2.83241  0.41328
N   1.72278  2.66656 -0.36705
N  -0.18477  2.86915 -1.37217
C  -0.13673 -2.02269  0.11565
C   0.40946 -4.25531 -0.08540
H   1.06898 -5.09683 -0.27105
C  -0.90020 -4.19130  0.27941
H  -1.63586 -4.96371  0.47972
C   2.25198 -2.61544 -0.37791
C   3.00027 -2.22388  0.76116
C   4.36698 -1.95794  0.57078
H   4.97218 -1.63086  1.41973
C   4.97117 -2.15275 -0.67871
H   6.04274 -1.96547 -0.79947
C   4.21799 -2.60980 -1.76628
H   4.70716 -2.78434 -2.72946
C   2.83236 -2.83652 -1.64671
C   2.39469 -2.27454  2.16021
H   1.30857 -2.11450  2.06858
C   2.61786 -3.68544  2.75213
H   2.16349 -4.46691  2.12110
H   2.16940 -3.75708  3.75728
H   3.69571 -3.90419  2.84061
C   2.92904 -1.19018  3.10399
H   3.99592 -1.33477  3.34648
H   2.37403 -1.21709  4.05742
H   2.81769 -0.19209  2.65636
C   2.02897 -3.36405 -2.83133
H   0.95719 -3.28577 -2.57482
C   2.35707 -4.85377 -3.08086
H   3.41860 -4.97721 -3.35541
H   1.74344 -5.25273 -3.90579
H   2.17465 -5.47399 -2.18801
C   2.25952 -2.54878 -4.11881
H   2.02930 -1.48298 -3.96900
H   1.61444 -2.93114 -4.92724
H   3.30366 -2.62234 -4.46709
C  -2.52470 -2.44322  0.87456
C  -3.59443 -2.50984 -0.04910
```

C -4.89645 -2.30558 0.44665
H -5.74613 -2.35665 -0.24057
C -5.11355 -2.04417 1.80205
H -6.13173 -1.88902 2.17167
C -4.03269 -1.97368 2.69351
H -4.22394 -1.76995 3.74944
C -2.71223 -2.18978 2.26000
C -3.37500 -2.84685 -1.51932
H -2.28793 -2.83127 -1.71127
C -3.89049 -4.26688 -1.83827
H -4.97657 -4.34172 -1.65867
H -3.39517 -5.02935 -1.21515
H -3.70297 -4.51613 -2.89608
C -4.02599 -1.80431 -2.44715
H -3.78330 -2.02795 -3.49982
H -3.67056 -0.78607 -2.22207
H -5.12520 -1.80323 -2.35262
C -1.55010 -2.21780 3.25405
H -0.70362 -1.67167 2.78689
C -1.06967 -3.66090 3.53842
H -0.27412 -3.64433 4.30188
H -0.66715 -4.16185 2.64701
H -1.90355 -4.26714 3.93163
C -1.88180 -1.54013 4.59460
H -2.33629 -0.54829 4.46368
H -0.95995 -1.41901 5.18669
H -2.57144 -2.16107 5.19198
C -4.12463 0.95431 0.32854
H -4.43809 0.46471 1.26127
H -4.75660 0.60802 -0.50189
H -4.20968 2.04522 0.42087
C -1.52082 1.70711 3.53422
H -1.36763 2.79528 3.45499
H -1.18431 1.37394 4.52769
H -2.59758 1.49826 3.42540
C   0.55029 1.96750 -0.61672
C   1.71636 3.92499 -0.97515
H   2.57197 4.59032 -0.90801
C   0.51879 4.05060 -1.61257
H   0.11324 4.84250 -2.23430
C   2.90915 2.19267 0.31309
C   2.99266 2.33878 1.71615
C   4.19401 1.94555 2.33509
H   4.29651 2.03859 3.41903
C   5.26149 1.44303 1.58246
H   6.18622 1.14160 2.08449
C   5.16441 1.35516 0.18870
H   6.01992 1.00007 -0.39014
C   3.99363 1.75000 -0.48293
C   1.86824 2.98407 2.51764
H   0.91374 2.73225 2.01258
C   1.78719 2.48276 3.96909
H   1.72745 1.38273 4.02256
H   0.89561 2.89938 4.46218
H   2.66385 2.80314 4.55661
C   1.98939 4.52447 2.48936
H   2.94262 4.84453 2.94327
H   1.16486 4.98318 3.06083
H   1.94968 4.91828 1.46172
C   3.90715 1.76395 -2.00689
H   3.37706 2.69179 -2.29238
C   3.07655 0.57972 -2.53247
H   2.86957 0.69808 -3.60990
H   2.11656 0.48598 -2.00138
H   3.62526 -0.36046 -2.38356
C   5.28026 1.79505 -2.69898
H   5.82658 0.84669 -2.55552
H   5.91273 2.61798 -2.32726
H   5.14642 1.93016 -3.78468
C -1.52213 2.66649 -1.86274
C -2.55067 3.54832 -1.43710
C -3.82158 3.36137 -2.01200
H -4.64540 4.01387 -1.71597
C -4.05585 2.34750 -2.94930
H -5.05537 2.22666 -3.37804
C -3.01958 1.49396 -3.34664
H -3.20827 0.72272 -4.09676
C -1.72354 1.64060 -2.81840
C -0.40851 -0.40680 -2.17814
H   0.94614 0.15267 1.34963
H   0.45703 -1.02889 -2.48896
H -1.28307 -1.07818 -2.27100
C -0.57539 0.73034 -3.21458
H   0.34611 1.34149 -3.20005
C -0.72308 0.15170 -4.63410
H -1.55124 -0.57630 -4.69091
H   0.19690 -0.38669 -4.91302
H -0.90168 0.93833 -5.38657
C -2.30446 4.63048 -0.38095
H -1.42032 5.21493 -0.69377
C -1.96206 4.01015 0.99137
H -2.81904 3.44688 1.39368
H -1.70383 4.80095 1.71529
C -3.46657 5.62614 -0.23388
H -3.72621 6.10347 -1.19303
H -3.18417 6.42203 0.47394
H -4.37196 5.13817 0.16715
H -1.10640 3.32009 0.92814

Int(2^+-3^+)4′

SCF =   -2959.28041473
H(0 K)=   -2958.094244
G(298 K)=   -2958.198406
SCF (C6H5F) =   -2959.32891974
Lowest Frequencies = 18.7531cm-1, 25.1551cm-1
SCF [PBE0(C6H5F, D3)] = -6060.75373926

141
Ir -0.10701 -0.02378 -0.11748
Zn 2.29593 -0.29257 0.01857
Zn 0.52331 -0.92670 2.12229
N -1.05893 2.89137 -0.11632
N   1.00473 2.88177 0.51582
N -1.42235 -2.83352 -0.27819
N   0.47214 -2.86177 -1.32396
C -0.01893 2.02224 0.16597
C -0.68827 4.22830 0.04409
H -1.39367 5.03650 -0.11843

C	0.61458	4.22454	0.43450
H	1.30196	5.02764	0.68032
C	-2.43135	2.52155	-0.35944
C	-3.19234	2.07917	0.75088
C	-4.54991	1.79414	0.52449
H	-5.16656	1.43419	1.35187
C	-5.13176	2.01333	-0.73141
H	-6.19736	1.81110	-0.87915
C	-4.36641	2.51527	-1.78984
H	-4.83930	2.70523	-2.75801
C	-2.99015	2.76956	-1.63434
C	-2.61375	2.09661	2.16235
H	-1.52163	1.97103	2.08633
C	-2.88936	3.47580	2.80458
H	-2.45033	4.29526	2.21224
H	-2.45797	3.52131	3.81876
H	-3.97467	3.65810	2.88452
C	-3.13354	0.96126	3.05216
H	-4.21018	1.06203	3.27309
H	-2.60313	0.97019	4.01971
H	-2.97749	-0.01480	2.57118
C	-2.17694	3.36881	-2.77889
H	-1.10851	3.30275	-2.50536
C	-2.53743	4.86062	-2.96619
H	-3.59607	4.96988	-3.25735
H	-1.91781	5.31203	-3.75902
H	-2.38897	5.44426	-2.04320
C	-2.36381	2.61507	-4.10990
H	-2.10152	1.55100	-4.01275
H	-1.71934	3.05837	-4.88700
H	-3.40456	2.67486	-4.47056
C	2.34178	2.56555	0.95011
C	3.39208	2.74997	0.01981
C	4.71137	2.64876	0.50120
H	5.54733	2.79341	-0.18957
C	4.96244	2.37154	1.84812
H	5.99376	2.30057	2.20695
C	3.90053	2.17113	2.74307
H	4.11824	1.94858	3.79020
C	2.56274	2.27892	2.32298
C	3.12495	3.09523	-1.44080
H	2.03919	3.00019	-1.61739
C	3.52786	4.55430	-1.74376
H	4.60790	4.70809	-1.57854
H	2.98558	5.26743	-1.10162
H	3.30624	4.80465	-2.79469
C	3.83455	2.11639	-2.39451
H	3.57185	2.34743	-3.44077
H	3.54328	1.07360	-2.19110
H	4.93231	2.18113	-2.30479
C	1.40521	2.15320	3.31287
H	0.60203	1.58241	2.80166
C	0.82018	3.53342	3.69442
H	0.01845	3.40505	4.44077
H	0.39366	4.06752	2.83337
H	1.60339	4.16763	4.14373
C	1.79164	1.40252	4.59875
H	2.31268	0.45674	4.39082
H	0.88533	1.17277	5.18257
H	2.44175	2.02182	5.24043

C	4.18498	-0.64847	0.32512
H	4.49353	-0.13962	1.24866
H	4.76647	-0.26132	-0.52408
H	4.34804	-1.73036	0.41188
C	1.46576	-1.86513	3.54131
H	1.28387	-2.94753	3.44160
H	1.11529	-1.53703	4.53195
H	2.55072	-1.68658	3.46563
C	-0.34285	-2.01973	-0.58007
C	-1.29683	-4.10231	-0.84729
H	-2.08636	-4.84233	-0.76131
C	-0.10593	-4.11927	-1.50977
H	0.36610	-4.88241	-2.11999
C	-2.68663	-2.38788	0.25676
C	-2.95077	-2.54641	1.63449
C	-4.23220	-2.18128	2.08545
H	-4.48198	-2.29133	3.14422
C	-5.19148	-1.67263	1.19837
H	-6.17978	-1.38930	1.57381
C	-4.90045	-1.54368	-0.16457
H	-5.66321	-1.16707	-0.85260
C	-3.64344	-1.92209	-0.67088
C	-1.91599	-3.16092	2.56850
H	-0.91909	-2.98249	2.11820
C	-1.91637	-2.53939	3.97546
H	-1.78407	-1.44558	3.93849
H	-1.09569	-2.96397	4.57489
H	-2.85698	-2.74753	4.51239
C	-2.10433	-4.69233	2.65141
H	-3.09785	-4.93763	3.06377
H	-1.33917	-5.14032	3.30751
H	-2.02272	-5.16617	1.66004
C	-3.37247	-1.92514	-2.17064
H	-2.32392	-2.22662	-2.33187
C	-3.53363	-0.53111	-2.78877
H	-3.27218	-0.55438	-3.86025
H	-2.88136	0.19448	-2.28058
H	-4.56752	-0.15957	-2.70248
C	-4.26708	-2.96678	-2.87589
H	-5.33496	-2.71103	-2.76810
H	-4.12092	-3.97566	-2.45549
H	-4.03539	-3.00602	-3.95350
C	1.75392	-2.51529	-1.87542
C	2.89559	-3.27415	-1.50330
C	4.10926	-2.93354	-2.12963
H	5.01694	-3.48377	-1.87490
C	4.18278	-1.89392	-3.06495
H	5.14258	-1.65230	-3.53175
C	3.03648	-1.17031	-3.41391
H	3.10058	-0.37981	-4.16505
C	1.79061	-1.47193	-2.83425
C	0.27019	0.42123	-2.16068
H	-0.92530	-0.21887	1.38480
H	-0.69534	0.90817	-2.42338
H	1.03871	1.20425	-2.30648
C	0.52772	-0.70600	-3.18890
H	-0.31739	-1.41731	-3.12514
C	0.54670	-0.14385	-4.62279
H	1.27637	0.67813	-4.72680
H	-0.44248	0.27249	-4.87202

H 0.78998 -0.91916 -5.36877
C 2.82662 -4.39973 -0.46357
H 2.04954 -5.11346 -0.79333
C 2.39319 -3.88615 0.92742
H 3.15101 -3.20975 1.35278
H 2.26911 -4.73344 1.62187
C 4.13258 -5.20200 -0.33813
H 4.45313 -5.62680 -1.30344
H 3.98569 -6.03768 0.36487
H 4.95451 -4.58063 0.05830
H 1.43836 -3.34021 0.89231

TS(2^+-3^+)5
SCF = -2959.26750373
H(0 K)= -2958.080825
G(298 K)= -2958.183575
SCF (C6H5F) = -2959.31461273
Lowest Frequencies = -16.8536cm-1, 14.0608cm-1
SCF [PBE0(C6H5F, D3)] = -6060.73941958

141
Ir -0.06162 -0.08113 -0.19373
Zn 1.83756 1.24128 -0.84583
Zn 1.32763 0.30177 1.84190
N -2.57397 1.77033 -0.50739
N -1.10467 2.74682 0.74546
N 0.84653 -3.02898 0.29320
N 2.15056 -2.09535 -1.16105
C -1.28676 1.59358 -0.01341
C -3.17171 2.93578 -0.02057
H -4.19881 3.18706 -0.26387
C -2.25078 3.54793 0.76772
H -2.28762 4.46715 1.34228
C -3.41387 0.80081 -1.17474
C -4.14196 -0.09158 -0.35657
C -5.01897 -0.98636 -0.99884
H -5.59214 -1.69837 -0.39702
C -5.16915 -0.96949 -2.38967
H -5.85038 -1.67640 -2.87332
C -4.47338 -0.03212 -3.16696
H -4.62964 -0.00646 -4.24871
C -3.59644 0.89468 -2.57445
C -4.03436 -0.06610 1.16331
H -3.42795 0.80986 1.44627
C -5.41682 0.11340 1.82277
H -5.92939 1.01431 1.44724
H -5.30436 0.21100 2.91574
H -6.07433 -0.75213 1.63426
C -3.31715 -1.31643 1.69383
H -3.86417 -2.23335 1.42431
H -3.23489 -1.28385 2.79315
H -2.29662 -1.39976 1.28197
C -2.95125 2.01195 -3.38876
H -2.01618 2.29888 -2.87472
C -3.87634 3.25199 -3.42331
H -4.84788 2.99538 -3.87885
H -3.41716 4.05377 -4.02590
H -4.06916 3.65711 -2.41822
C -2.60195 1.59275 -4.82655
H -2.02895 0.65536 -4.84823
H -2.00099 2.37935 -5.31222
H -3.50844 1.45541 -5.44067
C -0.03307 3.05439 1.66311
C 0.98450 3.97055 1.28481
C 1.93082 4.30358 2.27480
H 2.74043 4.99333 2.02910
C 1.84261 3.79843 3.57853
H 2.58933 4.08777 4.32404
C 0.79503 2.94500 3.93506
H 0.71637 2.57937 4.96260
C -0.16971 2.55462 2.98703
C 1.03729 4.63601 -0.09631
H 0.93509 3.83441 -0.85494
C -0.11827 5.64449 -0.32179
H -0.17472 6.36195 0.51497
H -1.09688 5.16057 -0.43679
H 0.07338 6.21974 -1.24279
C 2.35685 5.38971 -0.35303
H 2.40598 5.69285 -1.41132
H 3.25072 4.78987 -0.13359
H 2.40176 6.31294 0.25078
C -1.37602 1.71971 3.41191
H -1.82281 1.28727 2.50248
C -2.43574 2.61194 4.09529
H -3.32145 2.01232 4.36519
H -2.76862 3.42980 3.43756
H -2.03193 3.06112 5.01867
C -0.99884 0.54290 4.32435
H -0.24388 -0.10245 3.84855
H -1.88835 -0.07671 4.52565
H -0.59754 0.87454 5.29670
C 3.39098 2.34750 -1.19976
H 3.78157 2.72203 -0.24100
H 3.12939 3.19312 -1.85202
H 4.14873 1.72639 -1.69429
C 2.80025 0.43995 3.10699
H 3.43863 -0.45604 3.06068
H 2.39361 0.54134 4.12532
H 3.40916 1.32990 2.88266
C 1.05682 -1.81641 -0.34452
C 1.72042 -4.02027 -0.16305
H 1.64068 -5.04506 0.18579
C 2.54017 -3.43432 -1.07649
H 3.33152 -3.83911 -1.69919
C -0.29678 -3.43134 1.08117
C -0.21387 -3.41679 2.49418
C -1.30341 -3.96106 3.19861
H -1.28686 -3.96782 4.29097
C -2.40013 -4.51854 2.52539
H -3.22719 -4.94726 3.09978
C -2.43972 -4.53363 1.12762
H -3.29697 -4.97591 0.61051
C -1.38315 -3.99144 0.37227
C 1.01454 -2.87635 3.22201
H 1.30371 -1.93474 2.71201
C 0.75488 -2.55253 4.70371
H -0.16676 -1.97097 4.85344
H 1.59845 -1.97087 5.10916

```
H   0.67530 -3.47590  5.30339
C   2.22646 -3.83306  3.12938
H   1.96746 -4.82188  3.54510
H   3.06704 -3.42728  3.71718
H   2.57855 -3.97201  2.09712
C  -1.42441 -4.02681 -1.15164
H  -0.45246 -3.67119 -1.53266
C  -2.50024 -3.06472 -1.68443
H  -2.49564 -3.04223 -2.78726
H  -2.33394 -2.03930 -1.31877
H  -3.50744 -3.37025 -1.35691
C  -1.62820 -5.45836 -1.68564
H  -2.60651 -5.86908 -1.38357
H  -0.84729 -6.14197 -1.31359
H  -1.59285 -5.46228 -2.78788
C   2.76837 -1.21283 -2.11400
C   4.16847 -0.98389 -2.04364
C   4.73860 -0.22167 -3.07992
H   5.81354 -0.02910 -3.07371
C   3.95216  0.31868 -4.10712
H   4.42250  0.92352 -4.88833
C   2.57271  0.08565 -4.14138
H   1.97181  0.49573 -4.95706
C   1.95657 -0.72096 -3.16567
C  -0.30663 -0.04579 -2.32033
H  -0.31542 -0.35600  1.47377
H  -1.38202 -0.31363 -2.38057
H  -0.23098  0.95836 -2.78180
C   0.47729 -1.04806 -3.19918
H   0.35096 -2.04972 -2.74872
C  -0.08351 -1.13911 -4.62919
H  -0.06785 -0.16414 -5.14423
H  -1.13339 -1.47450 -4.59273
H   0.48467 -1.85790 -5.24384
C   5.00956 -1.47781 -0.86546
H   4.78388 -2.54668 -0.70021
C   4.60450 -0.73435  0.42533
H   4.80855  0.34546  0.33821
H   5.16970 -1.12171  1.28903
C   6.52550 -1.36976 -1.09516
H   6.84215 -1.88662 -2.01612
H   7.06115 -1.82862 -0.24862
H   6.85375 -0.31776 -1.15739
H   3.53232 -0.85987  0.64382
```

TS(2$^+$-3$^+$)1B

```
SCF  =          -2959.24802370
H(0 K)=         -2958.064862
G(298 K)=       -2958.169280
Lowest Frequencies = -721.5198cm-
1,  21.6905cm-1
SCF [PBE0(C6H5F, D3)] =
-6060.71044646241

141
Ir -0.08115  0.17969 -0.17189
Zn  1.58068 -1.25584  1.04431
Zn -0.88249 -2.10686 -0.03473
N  -2.78387  0.81772  1.34181
N  -1.29116  0.02977  2.69561
N   2.51262  0.74379 -1.82349
N   1.56300 -1.10775 -2.41772
C  -1.47650  0.35015  1.35308
C  -3.36538  0.79837  2.60943
H  -4.37949  1.14775  2.77557
C  -2.42312  0.30837  3.46460
H  -2.43293  0.15399  4.53889
C  -3.43560  1.44227  0.21805
C  -4.32429  0.67394 -0.56508
C  -4.93356  1.31440 -1.66005
H  -5.62815  0.75602 -2.29366
C  -4.64558  2.65252 -1.96006
H  -5.11895  3.12907 -2.82412
C  -3.77002  3.39199 -1.15164
H  -3.57800  4.44367 -1.38340
C  -3.15520  2.80687 -0.02887
C  -4.61103 -0.78619 -0.22708
H  -3.75362 -1.16604  0.35982
C  -5.87442 -0.89795  0.65584
H  -5.77303 -0.32242  1.58973
H  -6.06649 -1.95050  0.92347
H  -6.75743 -0.51305  0.11772
C  -4.74727 -1.68119 -1.47167
H  -5.63274 -1.41764 -2.07436
H  -4.86019 -2.73345 -1.16427
H  -3.85712 -1.61392 -2.11749
C  -2.32920  3.63336  0.95186
H  -1.71292  2.93410  1.54005
C  -3.28263  4.36091  1.92814
H  -3.91615  5.08536  1.38838
H  -2.70696  4.91048  2.69200
H  -3.94970  3.65281  2.44625
C  -1.36303  4.62876  0.29048
H  -0.59781  4.10918 -0.30530
H  -0.84498  5.21412  1.06898
H  -1.88327  5.34984 -0.36249
C  -0.03987 -0.30079  3.33517
C   0.83794  0.76889  3.64224
C   2.01143  0.45963  4.35281
H   2.71226  1.25953  4.60638
C   2.27702 -0.85247  4.76989
H   3.19038 -1.07081  5.33117
C   1.37684 -1.88299  4.47724
H   1.59439 -2.90123  4.81052
C   0.19554 -1.63251  3.75182
C   0.51680  2.19032  3.20126
H  -0.58114  2.26952  3.10905
C   0.96464  3.25941  4.21602
H   2.06379  3.32656  4.28298
H   0.57090  3.05189  5.22426
H   0.59839  4.24985  3.89949
C   1.13774  2.47582  1.82520
H   0.85725  3.46896  1.44437
H   0.80856  1.72120  1.05635
H   2.23302  2.42207  1.87325
C  -0.80098 -2.74740  3.46425
H  -1.46201 -2.39826  2.64987
C  -1.68311 -3.02753  4.70082
H  -2.41437 -3.82281  4.47920
H  -2.24089 -2.13183  5.01810
```

H -1.06478 -3.35848 5.55268
C -0.11197 -4.03907 2.98872
H 0.55402 -3.84698 2.13159
H -0.86599 -4.77930 2.67813
H 0.49531 -4.49700 3.78734
C 3.20027 -2.20255 1.61113
H 3.91790 -2.29734 0.77723
H 2.94918 -3.21447 1.96952
H 3.68565 -1.66021 2.43765
C -2.01725 -3.68918 0.09706
H -2.62398 -3.77257 -0.81656
H -2.68156 -3.60910 0.97337
H -1.39482 -4.59169 0.19450
C 1.42213 -0.05356 -1.53587
C 3.29159 0.20331 -2.85367
H 4.18323 0.70652 -3.21396
C 2.69261 -0.96147 -3.23279
H 2.94404 -1.68913 -3.99789
C 2.73779 2.05782 -1.27486
C 2.00928 3.13799 -1.83070
C 2.24752 4.41675 -1.29611
H 1.70364 5.27711 -1.69762
C 3.20637 4.60816 -0.29108
H 3.39132 5.61392 0.09896
C 3.94666 3.52525 0.19898
H 4.70706 3.69416 0.96718
C 3.71761 2.21762 -0.27150
C 1.14044 2.94704 -3.06934
H 0.89957 1.87506 -3.14324
C -0.19513 3.70684 -3.04395
H -0.83051 3.38117 -2.20638
H -0.74684 3.51947 -3.98068
H -0.05439 4.79804 -2.96235
C 1.96346 3.32522 -4.32276
H 2.23207 4.39526 -4.30576
H 1.38285 3.13082 -5.24042
H 2.89914 2.74517 -4.37951
C 4.53977 1.03801 0.23698
H 4.01366 0.11021 -0.05166
C 4.69132 1.01839 1.76885
H 5.28884 0.14371 2.07270
H 3.71418 0.94243 2.27135
H 5.20816 1.91795 2.14429
C 5.92765 1.02905 -0.44294
H 6.49687 1.93654 -0.17785
H 5.84491 0.99805 -1.54165
H 6.51109 0.15114 -0.11866
C 0.55432 -2.10572 -2.63159
C -0.69522 -1.66595 -3.14025
C -1.64013 -2.65148 -3.46965
H -2.60495 -2.35536 -3.88852
C -1.34993 -4.01444 -3.29636
H -2.09419 -4.76712 -3.57317
C -0.12389 -4.41373 -2.75577
H 0.07882 -5.47727 -2.59912
C 0.85793 -3.46581 -2.40060
C 1.92633 -4.78005 -0.52865
H 1.27201 -4.26264 0.19210
H 2.87859 -5.00579 -0.02232
H 1.44587 -5.73750 -0.79166
C 2.17469 -3.90494 -1.77290
H 2.70706 -2.99780 -1.43551
C 3.06956 -4.63845 -2.79386
H 2.58126 -5.55966 -3.15520
H 4.02964 -4.92215 -2.33186
H 3.28628 -4.01147 -3.67470
C -0.98658 -0.17977 -3.28286
H -0.04605 0.31902 -3.57626
C -1.49656 0.43391 -1.96017
H -2.30396 -0.17959 -1.54756
H -1.95770 1.40640 -2.19839
C -2.02420 0.13456 -4.38177
H -1.75756 -0.34680 -5.33687
H -2.07359 1.22331 -4.54385
H -3.03710 -0.19587 -4.09504
H -0.74775 1.45126 -0.96346

Int(2^+-3^+)1B

SCF = -2959.26246087
H(0 K)= -2958.077766
G(298 K)= -2958.182221
SCF (C6H5F) = -2959.30900541
Lowest Frequencies = 23.2061cm-1, 27.4082cm-1
SCF [PBE0(C6H5F, D3)] = -6060.72686641987

141
Ir -0.10451 0.26176 -0.12210
Zn 1.60923 -1.34497 0.91038
Zn -0.49869 -2.24058 -0.26884
N -3.02893 0.26794 1.09127
N -1.57947 -0.52907 2.49019
N 2.47257 1.41621 -1.46893
N 1.98736 -0.54535 -2.24935
C -1.67750 -0.00727 1.20325
C -3.72751 -0.04575 2.25821
H -4.79489 0.12857 2.34663
C -2.81506 -0.54162 3.14192
H -2.91332 -0.88058 4.16834
C -3.63563 0.97303 -0.01182
C -4.35044 0.23778 -0.98341
C -4.87983 0.95982 -2.06893
H -5.43334 0.43047 -2.84917
C -4.69433 2.34481 -2.17152
H -5.10273 2.88497 -3.03132
C -4.01136 3.04802 -1.16932
H -3.90442 4.13364 -1.24677
C -3.47546 2.37789 -0.05452
C -4.56749 -1.26653 -0.84284
H -3.75087 -1.66681 -0.21322
C -5.90787 -1.54692 -0.12398
H -5.95347 -1.07013 0.86772
H -6.05309 -2.63161 0.01294
H -6.75211 -1.15921 -0.71921
C -4.53086 -2.02293 -2.18283
H -5.36823 -1.73610 -2.84108
H -4.61363 -3.10672 -2.00114
H -3.58972 -1.84148 -2.72614
C -2.86485 3.13786 1.11877

H	-2.21719	2.43445	1.66676
C	-3.99470	3.58538	2.07485
H	-4.67220	4.29677	1.57248
H	-3.57363	4.08288	2.96505
H	-4.59989	2.72921	2.41540
C	-1.98215	4.32925	0.71749
H	-1.12201	4.00255	0.11432
H	-1.59414	4.82430	1.62406
H	-2.53896	5.09231	0.14728
C	-0.35881	-0.82303	3.20374
C	0.37209	0.26925	3.73248
C	1.51303	-0.01605	4.50264
H	2.09702	0.80701	4.92386
C	1.89542	-1.33999	4.75531
H	2.78247	-1.54562	5.36163
C	1.14682	-2.40143	4.23388
H	1.45365	-3.43043	4.44047
C	-0.00014	-2.17064	3.44889
C	-0.04620	1.69761	3.42604
H	-1.11997	1.68961	3.17293
C	0.13669	2.66805	4.60827
H	1.19993	2.80872	4.86594
H	-0.38987	2.30849	5.50685
H	-0.26830	3.65880	4.34436
C	0.73354	2.20219	2.20633
H	0.34524	3.15009	1.80678
H	0.69741	1.46701	1.34696
H	1.79893	2.32368	2.43926
C	-0.83306	-3.33484	2.93048
H	-1.49502	-2.94451	2.13631
C	-1.72809	-3.90984	4.05031
H	-2.34741	-4.73526	3.66153
H	-2.40301	-3.14535	4.46772
H	-1.11341	-4.30283	4.87807
C	0.03696	-4.44495	2.31236
H	0.71096	-4.04298	1.53541
H	-0.59971	-5.20970	1.84050
H	0.66212	-4.94679	3.06947
C	3.39264	-1.65687	1.65713
H	4.17272	-1.48948	0.89460
H	3.47783	-2.69123	2.02708
H	3.56669	-0.97282	2.50170
C	-1.67515	-3.76839	-0.61720
H	-2.24381	-3.60579	-1.54461
H	-2.38438	-3.91984	0.21431
H	-1.07314	-4.68318	-0.73920
C	1.53893	0.40702	-1.34398
C	3.44098	1.11626	-2.43082
H	4.23821	1.81178	-2.67173
C	3.13332	-0.11547	-2.92718
H	3.59836	-0.71974	-3.69932
C	2.39393	2.69245	-0.80535
C	1.49514	3.64980	-1.33217
C	1.41973	4.88812	-0.66948
H	0.73804	5.65754	-1.04455
C	2.23319	5.16062	0.43996
H	2.16773	6.13468	0.93481
C	3.15152	4.20793	0.90034
H	3.80015	4.44846	1.74790
C	3.24867	2.94186	0.29141
C	0.76621	3.40809	-2.65063
H	0.80035	2.32632	-2.85773
C	-0.71722	3.80859	-2.64241
H	-1.28231	3.22306	-1.90118
H	-1.15965	3.61758	-3.63474
H	-0.86090	4.88000	-2.42098
C	1.53288	4.12491	-3.78564
H	1.52598	5.21802	-3.63513
H	1.06704	3.91041	-4.76231
H	2.58561	3.79919	-3.82774
C	4.29406	1.92055	0.73558
H	3.99233	0.93412	0.34020
C	4.43173	1.77733	2.26300
H	5.22159	1.04418	2.49466
H	3.50178	1.41459	2.72820
H	4.71531	2.72835	2.74483
C	5.66623	2.28658	0.12187
H	6.01699	3.25764	0.51138
H	5.61877	2.36981	-0.97570
H	6.42013	1.52251	0.37516
C	1.28065	-1.74090	-2.62438
C	0.00936	-1.58150	-3.23328
C	-0.62397	-2.72854	-3.73850
H	-1.60054	-2.63518	-4.22033
C	-0.01517	-3.98747	-3.63683
H	-0.51831	-4.87015	-4.04236
C	1.22269	-4.12142	-2.99941
H	1.67690	-5.11210	-2.90446
C	1.90268	-3.00298	-2.47620
C	3.13389	-4.23545	-0.64664
H	2.31324	-3.99437	0.04992
H	4.07008	-4.27364	-0.06675
H	2.94492	-5.24341	-1.05212
C	3.23947	-3.18207	-1.76774
H	3.50584	-2.21970	-1.29542
C	4.35969	-3.55833	-2.76098
H	4.14137	-4.52223	-3.25151
H	5.32441	-3.65530	-2.23588
H	4.47858	-2.80365	-3.55589
C	-0.65316	-0.21944	-3.23385
H	0.12628	0.54670	-3.39520
C	-1.31599	0.03734	-1.86817
H	-2.03490	-0.77554	-1.66931
H	-1.88552	0.97423	-1.92559
C	-1.70238	-0.02546	-4.34622
H	-1.27598	-0.24665	-5.33882
H	-2.05556	1.01807	-4.34162
H	-2.58849	-0.66494	-4.20018
H	-0.56285	1.77315	-0.44148

TS(2^+-3^+)2B

SCF = -2959.24676997
H(0 K)= -2958.063116
G(298 K)= -2958.168292
SCF (C6H5F) = -2959.29396307
Lowest Frequencies = -465.0916cm-1, 14.0007cm-1
SCF [PBE0(C6H5F, D3)] = -6060.71255188344

```
141
Ir -0.15777  0.14009 -0.17036
Zn  1.79965 -0.37416  1.17313
Zn  0.47474 -2.36199  0.11439
N  -2.99104 -0.84257  0.83404
N  -1.59434 -0.59028  2.46633
N   1.85860  2.04132 -1.62916
N   2.40411 -0.03506 -1.93824
C  -1.68377 -0.46694  1.09086
C  -3.68627 -1.14714  2.00877
H  -4.73453 -1.42660  1.99248
C  -2.80699 -0.98774  3.03768
H  -2.92146 -1.09810  4.11130
C  -3.62656 -0.73715 -0.45553
C  -3.77610 -1.90169 -1.24421
C  -4.31438 -1.73416 -2.53278
H  -4.43690 -2.60416 -3.18334
C  -4.68561 -0.46472 -3.00034
H  -5.08993 -0.35652 -4.01163
C  -4.57113  0.65936 -2.17282
H  -4.90155  1.63721 -2.53528
C  -4.04920  0.54719 -0.86987
C  -3.40329 -3.27983 -0.70294
H  -2.60541 -3.14018  0.05144
C  -4.62102 -3.91841  0.00631
H  -5.00109 -3.28773  0.82456
H  -4.34912 -4.89926  0.43091
H  -5.44607 -4.06998 -0.71032
C  -2.87007 -4.24821 -1.77383
H  -3.64435 -4.50613 -2.51591
H  -2.54801 -5.18794 -1.29732
H  -2.00505 -3.83040 -2.31399
C  -4.06812  1.72889  0.09434
H  -3.42163  1.47426  0.94929
C  -5.50541  1.90861  0.63476
H  -6.20164  2.17686 -0.17828
H  -5.53464  2.71265  1.38937
H  -5.87873  0.98274  1.10350
C  -3.52360  3.03969 -0.49388
H  -2.46008  2.94128 -0.75775
H  -3.61220  3.84562  0.25386
H  -4.08356  3.36099 -1.38893
C  -0.48629 -0.12256  3.25722
C  -0.44345  1.26049  3.56430
C   0.59298  1.69891  4.40922
H   0.64955  2.75763  4.67959
C   1.52435  0.79518  4.93764
H   2.31629  1.15579  5.60120
C   1.44244 -0.56960  4.63273
H   2.16997 -1.26362  5.06228
C   0.43450 -1.06364  3.78239
C  -1.54613  2.21177  3.11607
H  -2.14939  1.69080  2.35624
C  -2.47193  2.53362  4.30987
H  -1.92472  3.07159  5.10288
H  -2.89283  1.61620  4.75410
H  -3.30984  3.17137  3.98176
C  -1.01450  3.48858  2.44890
H  -1.85767  4.12427  2.13143
H  -0.42618  3.24447  1.55187
H  -0.38764  4.08753  3.13133
C   0.28720 -2.55788  3.51747
H  -0.32793 -2.67485  2.60548
C  -0.46864 -3.24013  4.67945
H  -0.59911 -4.31583  4.47456
H  -1.46672 -2.79902  4.83080
H   0.09338 -3.13544  5.62317
C   1.63393 -3.26026  3.26910
H   2.20063 -2.77477  2.45455
H   1.46648 -4.30978  2.98074
H   2.27212 -3.25675  4.16848
C   3.43488  0.17493  2.08753
H   4.17755  0.49495  1.33973
H   3.83480 -0.66115  2.67859
H   3.18754  1.01286  2.75339
C   0.26463 -4.30843  0.20037
H  -0.38061 -4.60738  1.04387
H   1.25449 -4.77596  0.33045
H  -0.17385 -4.69102 -0.73402
C   1.43876  0.76224 -1.31694
C   3.00988  2.04348 -2.41382
H   3.45883  2.96831 -2.76213
C   3.35102  0.73783 -2.61504
H   4.15467  0.28258 -3.18459
C   1.14397  3.25224 -1.31293
C   0.09123  3.63599 -2.17185
C  -0.60762  4.81043 -1.83973
H  -1.43931  5.13696 -2.47195
C  -0.24062  5.57341 -0.72261
H  -0.79318  6.48724 -0.48342
C   0.84476  5.18845  0.07570
H   1.13593  5.80893  0.92831
C   1.56299  4.00976 -0.19929
C  -0.24139  2.85305 -3.43795
H   0.45593  1.99984 -3.49915
C  -1.66532  2.27008 -3.41032
H  -1.81543  1.63957 -2.52111
H  -1.84684  1.65724 -4.30977
H  -2.42746  3.06720 -3.38850
C  -0.01246  3.72424 -4.69087
H  -0.69632  4.58991 -4.70608
H  -0.19391  3.13664 -5.60663
H   1.01915  4.11167 -4.72765
C   2.79094  3.61295  0.61340
H   3.01915  2.55667  0.38521
C   2.57431  3.70858  2.13252
H   3.49918  3.42387  2.66157
H   1.77083  3.03310  2.46052
H   2.31646  4.73322  2.45025
C   4.00389  4.46608  0.17780
H   3.82694  5.53361  0.39395
H   4.20019  4.37522 -0.90337
H   4.91271  4.15305  0.71898
C   2.37987 -1.46984 -2.07827
C   1.27657 -2.04426 -2.76340
C   1.30557 -3.42264 -3.02910
H   0.46519 -3.88675 -3.55141
C   2.40015 -4.20534 -2.63545
H   2.41330 -5.27644 -2.85670
C   3.46533 -3.62019 -1.94472
```

```
H   4.30597 -4.24226 -1.62382
C   3.48443 -2.24179 -1.64757
C   4.88201 -2.43408  0.44646
H   3.95184 -2.50700  1.03330
H   5.64016 -1.92712  1.06530
H   5.23927 -3.45900  0.25190
C   4.65396 -1.65617 -0.86635
H   4.39766 -0.61598 -0.59918
C   5.94641 -1.63430 -1.71064
H   6.24872 -2.65781 -1.99029
H   6.77299 -1.18123 -1.13856
H   5.82492 -1.06136 -2.64483
C   0.06722 -1.18689 -3.06548
H   0.40646 -0.17375 -3.34532
C  -0.77793 -1.07309 -1.79011
H  -1.14603 -2.08373 -1.52891
H  -1.66659 -0.44483 -1.98502
C  -0.81843 -1.70006 -4.21777
H  -0.23023 -1.81691 -5.14292
H  -1.63233 -0.98212 -4.40635
H  -1.29095 -2.66771 -3.98282
H  -0.74502  1.69325 -0.17031
```

TS(1⁺-10⁺)

```
SCF  =           -2426.04754148
H(0 K)=          -2424.918379
G(298 K)=        -2425.013606
SCF (C6H5F) =    -2426.08598068
Lowest Frequencies = -457.1887cm-1,  17.4508cm-1
SCF [PBE0(C6H5F, D3)] = -2423.78515901101

133
Ir -0.01089 -0.11795 -0.26247
H   0.96073  0.04697  0.94625
H   0.81357 -1.47917 -0.16815
N  -1.05962 -1.08395  2.44192
N  -2.70364 -1.21979  1.04411
N   1.58474  1.93553 -1.93593
N   2.55095  0.00221 -2.01661
C  -1.37782 -0.84393  1.12143
C  -3.18694 -1.66082  2.28158
H  -4.22225 -1.95946  2.41162
C  -2.14637 -1.58121  3.16039
H  -2.06935 -1.82172  4.21618
C   0.18352 -0.69352  3.05985
C   1.25333 -1.61348  3.07393
C   1.10561 -3.00837  2.48233
H   0.19130 -3.01166  1.86264
C   2.28163 -3.37505  1.56255
H   2.41963 -2.62287  0.76970
H   2.10261 -4.34872  1.07877
H   3.23188 -3.45451  2.11778
C   0.91633 -4.05028  3.60607
H   1.79921 -4.07890  4.26747
H   0.77596 -5.05840  3.18102
H   0.03782 -3.81475  4.22972
C   2.45061 -1.19466  3.68297
H   3.30021 -1.88273  3.72603
C   2.56593  0.08787  4.23513
H   3.50718  0.39619  4.70052
C   1.48255  0.97688  4.20259
H   1.58502  1.97260  4.64417
C   0.25950  0.60582  3.61556
C  -0.94601  1.53929  3.63565
H  -1.69673  1.13238  2.93778
C  -1.57392  1.55283  5.04706
H  -1.85113  0.53757  5.37606
H  -2.48250  2.17815  5.06019
H  -0.86647  1.96277  5.78805
C  -0.62402  2.96528  3.15607
H   0.14166  3.45008  3.78515
H  -1.53244  3.58856  3.20724
H  -0.26917  2.96517  2.11401
C  -3.58714 -0.93146 -0.06153
C  -3.84926 -1.93645 -1.02203
C  -3.33045 -3.35917 -0.84259
H  -2.38195 -3.30032 -0.27960
C  -3.05994 -4.08454 -2.17147
H  -3.99732 -4.32230 -2.70262
H  -2.54707 -5.04157 -1.98057
H  -2.42870 -3.48535 -2.84478
C  -4.33151 -4.17777  0.00732
H  -4.48461 -3.73533  1.00396
H  -3.96307 -5.20789  0.14757
H  -5.31243 -4.22893 -0.49489
C  -4.70124 -1.59600 -2.08939
H  -4.92048 -2.34037 -2.85954
C  -5.29409 -0.32769 -2.16475
H  -5.95786 -0.08725 -3.00106
C  -5.06630  0.62025 -1.16034
H  -5.56381  1.59417 -1.20834
C  -4.20844  0.33818 -0.07903
C  -4.01370  1.36093  1.03638
H  -3.40092  0.89412  1.82571
C  -3.25446  2.60950  0.55064
H  -2.23290  2.35797  0.22707
H  -3.17533  3.35070  1.36308
H  -3.77614  3.09439 -0.29258
C  -5.36670  1.73628  1.67655
H  -6.02302  2.26126  0.96190
H  -5.20586  2.40833  2.53598
H  -5.90489  0.84250  2.03291
C   1.46585  0.64895 -1.46207
C   2.68061  2.07661 -2.78852
H   2.91169  3.02377 -3.26649
C   3.28864  0.85533 -2.84258
H   4.15403  0.50262 -3.39560
C   0.76054  3.03898 -1.51520
C   1.26272  3.88980 -0.50738
C   2.64263  3.68755  0.11221
H   3.14354  2.86593 -0.42734
C   2.54277  3.25712  1.58833
H   2.05834  4.03690  2.19988
H   3.54959  3.07957  2.00250
H   1.95998  2.32902  1.69649
C   3.51748  4.94743 -0.05077
H   3.60695  5.24216 -1.10958
H   4.53101  4.76327  0.34299
```

```
H   3.09697  5.80519  0.50115
C   0.42536  4.94184 -0.08952
H   0.76947  5.61995  0.69757
C  -0.83375  5.13714 -0.67211
H  -1.46464  5.96596 -0.33683
C  -1.29024  4.28268 -1.68621
H  -2.27350  4.45034 -2.13465
C  -0.50096  3.20598 -2.12377
C  -0.99741  2.19172 -3.14635
H  -0.11484  1.67494 -3.56194
C  -1.76559  2.82463 -4.32154
H  -2.69962  3.30665 -3.98681
H  -2.04115  2.05202 -5.05829
H  -1.15318  3.58490 -4.83217
C  -1.87469  1.14075 -2.44526
H  -1.42361  0.81733 -1.46431
H  -2.03829  0.23980 -3.05704
H  -2.85076  1.55371 -2.15093
C   2.93780 -1.36575 -1.78176
C   4.17616 -1.61606 -1.14219
C   4.98135 -0.48564 -0.50167
H   4.97722  0.37981 -1.18834
C   6.45111 -0.84599 -0.23236
H   6.54458 -1.61976  0.54919
H   6.99356  0.04351  0.12684
H   6.95841 -1.21141 -1.14053
C   4.28200 -0.03510  0.80292
H   3.23963  0.27533  0.62750
H   4.82003  0.81332  1.25823
H   4.26164 -0.85972  1.53366
C   4.57523 -2.96152 -1.03555
H   5.52951 -3.20135 -0.56090
C   3.76446 -3.99951 -1.51442
H   4.10117 -5.03668 -1.42356
C   2.51769 -3.71928 -2.08747
H   1.88253 -4.53834 -2.43509
C   2.08076 -2.39138 -2.23704
C   0.69983 -2.04399 -2.76463
H   0.76463 -1.06445 -3.27289
C  -0.26312 -1.89293 -1.56761
C   0.16092 -3.05581 -3.79335
H   0.88196 -3.21433 -4.61238
H  -0.77752 -2.68208 -4.23510
H  -0.05966 -4.03408 -3.33448
H  -0.37264 -2.86193 -1.05391
H  -1.27734 -1.60549 -1.90918
H  -0.17207  1.36256  0.41917
```

10$^+$

```
SCF  =           -2426.05976923
H(0 K)=          -2424.929824
G(298 K)=        -2425.028030
SCF (C6H5F) =    -2426.09821357
Lowest Frequencies = 6.2474cm-1,
20.1915cm-1
SCF [PBE0(C6H5F, D3)] =
-2423.80277604494

133
Ir  0.00261 -0.04266 -0.07024
H  -0.92779  0.52350  1.32846
H  -0.14450  1.46728 -0.39842
N   1.80090  1.23395  2.10179
N   3.02593 -0.07394  0.89809
N  -2.67207 -1.26001 -1.15605
N  -2.14266  0.55645 -2.19666
C   1.72387  0.37377  1.02448
C   3.86393  0.47961  1.86893
H   4.91623  0.21918  1.92633
C   3.08993  1.31227  2.62489
H   3.32355  1.94799  3.47340
C   0.65773  1.88469  2.68491
C   0.25052  3.12740  2.15606
C   1.09563  3.85817  1.12013
H   1.84246  3.14409  0.73242
C   0.28009  4.35240 -0.08350
H  -0.23146  3.51904 -0.58904
H   0.94176  4.83245 -0.82250
H  -0.48306  5.09483  0.20798
C   1.85992  5.01635  1.79985
H   1.15903  5.76441  2.20844
H   2.51582  5.52495  1.07350
H   2.48442  4.65315  2.63322
C  -0.92500  3.69164  2.68603
H  -1.27723  4.65523  2.30568
C  -1.63962  3.04183  3.70192
H  -2.55236  3.49638  4.09934
C  -1.18137  1.82661  4.23204
H  -1.73431  1.34726  5.04580
C  -0.01654  1.21599  3.73241
C   0.52309 -0.08043  4.33073
H   1.34145 -0.43690  3.68165
C   1.12488  0.19838  5.72582
H   1.90965  0.97124  5.67688
H   1.56945 -0.71859  6.14768
H   0.34912  0.55444  6.42499
C  -0.52261 -1.20886  4.39665
H  -1.37125 -0.94684  5.05094
H  -0.06232 -2.12395  4.80594
H  -0.92602 -1.45061  3.39938
C   3.45020 -1.10988 -0.00770
C   4.31437 -0.78188 -1.07605
C   4.92330  0.60842 -1.22900
H   4.38485  1.29231 -0.54916
C   4.78386  1.17931 -2.65269
H   5.30298  0.55612 -3.40005
H   5.22663  2.18808 -2.69922
H   3.72548  1.25527 -2.94682
C   6.40809  0.57849 -0.79927
H   6.53216  0.19159  0.22622
H   6.84566  1.58985 -0.84150
H   6.99365 -0.07499 -1.46823
C   4.62579 -1.81051 -1.98357
H   5.28406 -1.59589 -2.83058
C   4.09807 -3.09931 -1.82724
H   4.34188 -3.87885 -2.55546
C   3.28108 -3.40176 -0.72958
H   2.89925 -4.41896 -0.60061
C   2.95431 -2.41316  0.21562
C   2.07085 -2.73002  1.41543
```

```
H   2.12325 -1.87924  2.11483
C   0.61040 -2.89222  0.97782
H   0.28105 -2.08442  0.26692
H  -0.09089 -2.90188  1.82471
H   0.46734 -3.81515  0.39786
C   2.53760 -3.98278  2.18265
H   2.47087 -4.89183  1.56152
H   1.90449 -4.14235  3.07158
H   3.58195 -3.87646  2.51692
C  -1.70372 -0.28250 -1.19554
C  -3.66194 -1.05238 -2.11954
H  -4.48846 -1.74485 -2.24614
C  -3.32172  0.09134 -2.78266
H  -3.78011  0.60014 -3.62514
C  -2.66751 -2.36083 -0.23083
C  -3.18985 -2.13476  1.06581
C  -3.91405 -0.83031  1.39288
H  -3.47978 -0.03362  0.76354
C  -3.78468 -0.37819  2.85498
H  -4.23881 -1.10022  3.55503
H  -4.30815  0.58222  2.99204
H  -2.73491 -0.22810  3.14508
C  -5.40518 -0.96954  1.00366
H  -5.52625 -1.22836 -0.06033
H  -5.94018 -0.02264  1.18703
H  -5.88921 -1.76060  1.60132
C  -3.12586 -3.20763  1.97276
H  -3.51186 -3.08018  2.98802
C  -2.60214 -4.45000  1.58007
H  -2.56908 -5.27472  2.29887
C  -2.14787 -4.65174  0.27083
H  -1.76951 -5.63485 -0.02667
C  -2.16627 -3.60368 -0.67109
C  -1.70468 -3.82348 -2.11075
H  -1.93646 -2.90854 -2.68223
C  -2.48544 -4.98510 -2.76120
H  -2.25656 -5.94890 -2.27534
H  -2.21588 -5.07745 -3.82637
H  -3.57423 -4.82614 -2.69258
C  -0.18249 -4.04584 -2.22480
H   0.38517 -3.17056 -1.87193
H   0.09378 -4.22548 -3.27732
H   0.14080 -4.92649 -1.64202
C  -1.42782  1.71455 -2.66023
C  -2.03803  2.98624 -2.55411
C  -3.36839  3.16417 -1.82010
H  -4.05294  2.36262 -2.15209
C  -4.06189  4.50339 -2.11587
H  -3.48719  5.35507 -1.71238
H  -5.05453  4.52530 -1.63755
H  -4.20103  4.66417 -3.19766
C  -3.17202  2.98260 -0.29511
H  -2.65975  2.03756 -0.05245
H  -4.14677  2.99146  0.22122
H  -2.56066  3.80177  0.11575
C  -1.32215  4.07442 -3.08687
H  -1.75421  5.07684 -3.04177
C  -0.05291  3.89619 -3.65611
H   0.48382  4.76019 -4.06023
C   0.53690  2.62606 -3.70548
H   1.53005  2.50453 -4.14639
C  -0.14802  1.49907 -3.21740
C   0.45961  0.10698 -3.19733
H  -0.36394 -0.62355 -3.30593
C   1.14547 -0.15152 -1.83145
C   1.43748 -0.15740 -4.35696
H   0.96179  0.01695 -5.33664
H   1.78313 -1.20355 -4.31894
H   2.33639  0.47788 -4.29737
H   1.99984  0.54290 -1.74864
H   1.57471 -1.17256 -1.85705
H  -0.99541 -0.33791  1.38874
```

11^+

```
SCF  =           -2428.46818735
H(0 K)=          -2427.303762
G(298 K)=        -2427.407295
SCF (C6H5F) =    -2428.50695509
Lowest Frequencies = 10.2750cm-1,
18.6296cm-1
SCF [PBE0(C6H5F, D3)] =
-2426.20609523945
```

```
137
Ir  0.01923 -0.01038 -0.03725
H  -1.25176 -0.00033  0.91864
H  -0.76319 -0.09540 -1.68283
H   1.75821  0.04279 -0.46856
H  -1.39239  0.08772 -1.13436
H   1.39796 -0.07293 -1.23068
N  -0.99568  2.92025 -0.23998
N   1.10421  2.88233  0.26650
N  -1.11471 -2.88199  0.32655
N   0.98484 -2.96993 -0.16994
C   0.04040  2.04940  0.00932
C   0.74165  4.22769  0.17798
H   1.45519  5.02309  0.36871
C  -0.58395  4.25155 -0.14943
H  -1.26978  5.07353 -0.32782
C  -2.33047  2.51833 -0.60575
C  -2.62157  2.36177 -1.97928
C  -1.56560  2.61513 -3.05142
H  -0.57489  2.57187 -2.56341
C  -1.57200  1.55154 -4.16600
H  -1.45689  0.53215 -3.76172
H  -0.74100  1.73625 -4.86675
H  -2.50572  1.57476 -4.75268
C  -1.73379  4.03185 -3.64396
H  -2.71707  4.13511 -4.13367
H  -0.95311  4.23426 -4.39635
H  -1.66472  4.80591 -2.86239
C  -3.93925  1.99742 -2.31584
H  -4.21436  1.88349 -3.36851
C  -4.89789  1.77259 -1.31920
H  -5.91431  1.48092 -1.60119
C  -4.56636  1.91497  0.03485
H  -5.32321  1.72334  0.79925
C  -3.27214  2.30274  0.42576
C  -2.90125  2.53119  1.88737
H  -1.83154  2.27321  1.99829
```

C -3.07959 4.01997 2.26617
H -2.45285 4.68372 1.65070
H -2.80535 4.18094 3.32237
H -4.13131 4.32695 2.13537
C -3.69528 1.64209 2.85612
H -4.75879 1.93490 2.90084
H -3.28846 1.74375 3.87557
H -3.64112 0.58370 2.56296
C 2.43720 2.44039 0.58736
C 3.34041 2.21394 -0.47746
C 2.92755 2.45327 -1.92692
H 1.83201 2.31281 -1.98880
C 3.57664 1.46469 -2.91041
H 4.66264 1.63651 -3.00464
H 3.14047 1.59269 -3.91491
H 3.42863 0.41942 -2.59701
C 3.23487 3.91056 -2.34043
H 2.71136 4.63605 -1.69775
H 2.92067 4.09045 -3.38237
H 4.31715 4.11212 -2.26861
C 4.64210 1.80650 -0.13460
H 5.37064 1.61090 -0.92570
C 5.01954 1.66116 1.20764
H 6.04249 1.36022 1.45414
C 4.09783 1.89605 2.23588
H 4.40667 1.77950 3.27933
C 2.77249 2.27384 1.94687
C 1.76005 2.51727 3.05977
H 0.77184 2.66484 2.59085
C 1.63625 1.30391 4.00179
H 1.36086 0.39567 3.44121
H 0.85618 1.49071 4.75867
H 2.57944 1.10339 4.53771
C 2.10942 3.80349 3.83842
H 3.09294 3.71404 4.33078
H 1.35608 3.99997 4.61957
H 2.14815 4.67973 3.16992
C -0.03749 -2.07364 0.05100
C -0.77424 -4.23487 0.27674
H -1.50251 -5.01204 0.48604
C 0.55202 -4.29085 -0.04471
H 1.22381 -5.12828 -0.20485
C -2.44338 -2.41825 0.63631
C -3.34306 -2.20825 -0.43309
C -2.93585 -2.49703 -1.87463
H -1.83816 -2.38005 -1.94139
C -3.56926 -1.52655 -2.88505
H -4.65746 -1.68585 -2.97650
H -3.13477 -1.68567 -3.88602
H -3.40982 -0.47634 -2.59553
C -3.27089 -3.95989 -2.24514
H -2.75937 -4.67622 -1.58273
H -2.96327 -4.17605 -3.28224
H -4.35653 -4.13906 -2.16467
C -4.64141 -1.78265 -0.10081
H -5.36785 -1.59810 -0.89653
C -5.01914 -1.60594 1.23754
H -6.03928 -1.28961 1.47633
C -4.10159 -1.83089 2.27165
H -4.41034 -1.69117 3.31245
C -2.77999 -2.22817 1.99210
C -1.76806 -2.44008 3.11214
H -0.80432 -2.71652 2.65076
C -2.19656 -3.60035 4.03405
H -3.15318 -3.37879 4.53742
H -1.43792 -3.77231 4.81586
H -2.32555 -4.53737 3.46714
C -1.53213 -1.14099 3.90938
H -1.20584 -0.32518 3.24428
H -0.75153 -1.30010 4.67247
H -2.44832 -0.81559 4.43057
C 2.32348 -2.59531 -0.54588
C 2.61820 -2.48475 -1.92124
C 1.57507 -2.76858 -2.99719
H 0.60397 -2.92687 -2.49613
C 1.92195 -4.06294 -3.76305
H 2.88653 -3.96537 -4.28988
H 1.14673 -4.28968 -4.51403
H 2.00019 -4.92328 -3.07804
C 1.40187 -1.57759 -3.96185
H 1.12676 -0.65605 -3.42113
H 0.60611 -1.79369 -4.69439
H 2.32654 -1.37142 -4.52673
C 3.92804 -2.09926 -2.26670
H 4.20331 -2.01700 -3.32281
C 4.87447 -1.80806 -1.27634
H 5.88346 -1.49734 -1.56452
C 4.53930 -1.90784 0.08144
H 5.28613 -1.66471 0.84091
C 3.25392 -2.31576 0.48130
C 2.87753 -2.49158 1.94902
H 1.80824 -2.22655 2.04711
C 3.67028 -1.57204 2.88982
H 4.73313 -1.86504 2.94775
H 3.26037 -1.63842 3.91087
H 3.61909 -0.52329 2.56240
C 3.04780 -3.96753 2.37823
H 2.42122 -4.64819 1.78120
H 2.76697 -4.09232 3.43756
H 4.09886 -4.28302 2.26341
H 0.78679 -0.01346 1.35813

TS(11^+-$11a^+$)

SCF = -2428.45930498
H(0 K)= -2427.296063
G(298 K)= -2427.398844
SCF (C6H5F) = -2428.49778230
Lowest Frequencies = -339.2237cm-1, 13.4926cm-1
SCF [PBE0(C6H5F, D3)] = -2426.19342503957

137
Ir 0.07897 -0.00743 -0.03012
H -1.27938 -0.00051 0.80525
H 0.07954 -0.05158 -1.68791
H 1.80927 0.41273 -0.19703
H -1.23851 -0.01439 -0.94703
H 1.79342 -0.44231 -0.31112
N -1.07441 2.86690 -0.36087

N	1.02488	2.95042	0.14428
N	-1.06620	-2.87476	0.38947
N	1.03407	-2.96654	-0.10868
C	-0.00345	2.06108	-0.06806
C	0.60539	4.27175	-0.02080
H	1.28476	5.10652	0.11903
C	-0.71943	4.21834	-0.34583
H	-1.44099	4.99622	-0.57367
C	-2.40150	2.40590	-0.68440
C	-2.70763	2.17499	-2.04227
C	-1.67068	2.37155	-3.14308
H	-0.67934	2.44670	-2.66224
C	-1.61041	1.17746	-4.11479
H	-1.40264	0.23751	-3.57855
H	-0.80894	1.33515	-4.85586
H	-2.55405	1.05182	-4.67213
C	-1.93065	3.69426	-3.89615
H	-2.91659	3.67747	-4.39127
H	-1.16288	3.85787	-4.67096
H	-1.91507	4.55738	-3.21007
C	-4.02928	1.79196	-2.34126
H	-4.31822	1.62796	-3.38386
C	-4.97342	1.61506	-1.32203
H	-5.99317	1.30855	-1.57484
C	-4.62308	1.82369	0.01903
H	-5.36863	1.66777	0.80249
C	-3.32644	2.23830	0.37127
C	-2.93884	2.54401	1.81520
H	-1.86323	2.30938	1.92462
C	-3.13623	4.04566	2.12797
H	-2.52618	4.69044	1.47679
H	-2.85415	4.25856	3.17289
H	-4.19400	4.32982	1.99470
C	-3.70549	1.69054	2.83730
H	-4.77167	1.97261	2.88558
H	-3.28415	1.84385	3.84434
H	-3.64380	0.62031	2.59266
C	2.36668	2.57194	0.50267
C	3.28850	2.31213	-0.53995
C	2.88753	2.46626	-2.00386
H	1.79604	2.30111	-2.06851
C	3.56148	1.43146	-2.92033
H	4.64680	1.60970	-3.01186
H	3.13577	1.49560	-3.93519
H	3.41584	0.40555	-2.54805
C	3.17706	3.90358	-2.49242
H	2.63778	4.65472	-1.89335
H	2.86838	4.02220	-3.54459
H	4.25558	4.12581	-2.42395
C	4.58819	1.92864	-0.16140
H	5.32956	1.70762	-0.93350
C	4.94343	1.83194	1.19090
H	5.96329	1.54434	1.46456
C	4.00329	2.09497	2.19597
H	4.29530	2.01129	3.24711
C	2.68096	2.45450	1.87393
C	1.64834	2.73273	2.95949
H	0.65773	2.78703	2.47548
C	1.57892	1.60190	4.00336
H	1.35754	0.63589	3.52123
H	0.78072	1.81342	4.73426
H	2.52310	1.50038	4.56504
C	1.92524	4.09915	3.62338
H	2.91026	4.10375	4.12081
H	1.15875	4.32210	4.38429
H	1.92051	4.91521	2.88160
C	0.00332	-2.07361	0.08200
C	-0.71011	-4.22599	0.40186
H	-1.43123	-4.99899	0.64742
C	0.61531	-4.28482	0.08034
H	1.29545	-5.12175	-0.04166
C	-2.39687	-2.41399	0.69705
C	-3.31193	-2.25696	-0.36883
C	-2.90779	-2.57189	-1.80608
H	-1.82714	-2.35518	-1.90065
C	-3.64436	-1.70760	-2.84138
H	-4.71526	-1.96813	-2.90264
H	-3.21393	-1.87227	-3.84293
H	-3.56665	-0.63747	-2.59914
C	-3.12112	-4.07195	-2.11482
H	-2.53275	-4.71998	-1.44662
H	-2.82208	-4.29614	-3.15269
H	-4.18502	-4.34063	-1.99960
C	-4.61429	-1.84786	-0.03260
H	-5.35304	-1.70089	-0.82435
C	-4.97984	-1.63618	1.30427
H	-6.00413	-1.33528	1.54525
C	-4.04491	-1.80271	2.33353
H	-4.34423	-1.63530	3.37293
C	-2.71773	-2.17870	2.04982
C	-1.68868	-2.34041	3.16295
H	-0.70752	-2.52674	2.69276
C	-2.02706	-3.55994	4.04595
H	-3.00187	-3.42949	4.54627
H	-1.26132	-3.69656	4.82788
H	-2.07861	-4.48595	3.44927
C	-1.54783	-1.05559	4.00300
H	-1.27569	-0.19762	3.36759
H	-0.75912	-1.18567	4.76315
H	-2.48358	-0.81057	4.53331
C	2.37269	-2.58996	-0.48074
C	2.67740	-2.48799	-1.85665
C	1.64004	-2.78259	-2.93332
H	0.65689	-2.87315	-2.43947
C	1.94698	-4.13065	-3.62056
H	2.92483	-4.10046	-4.13094
H	1.17697	-4.36475	-4.37450
H	1.97524	-4.95612	-2.88995
C	1.52867	-1.63806	-3.95942
H	1.29160	-0.68285	-3.46257
H	0.72665	-1.85739	-4.68382
H	2.46334	-1.50557	-4.53032
C	3.99123	-2.10965	-2.19279
H	4.27415	-2.03224	-3.24693
C	4.93301	-1.82001	-1.19698
H	5.94574	-1.51735	-1.48068
C	4.58853	-1.90674	0.15927
H	5.33079	-1.66150	0.92295
C	3.29813	-2.30430	0.55237
C	2.90327	-2.43863	2.01858

```
H   1.81515 -2.25477  2.08456
C   3.59563 -1.40530  2.92135
H   4.67948 -1.59548  3.00839
H   3.17518 -1.45616  3.93911
H   3.45565 -0.38277  2.53943
C   3.17679 -3.87491  2.52032
H   2.62484 -4.62683  1.93405
H   2.87246 -3.97717  3.57545
H   4.25208 -4.11169  2.44912
H   0.49149  0.03451  1.56892
```

11a⁺

```
SCF  =          -2428.45954991
H(0 K)=         -2427.295169
G(298 K)=       -2427.397916
SCF (C6H5F) =   -2428.49804933
Lowest Frequencies = 14.4618cm-1,
15.8578cm-1
SCF [PBE0(C6H5F, D3)] =
-2426.19326360661

137
Ir  0.08260 -0.00000  0.00020
H  -1.27105  0.01345  0.84796
H   0.31585 -0.05708 -1.63771
H   1.81266  0.43082  0.05040
H  -1.27225 -0.01339 -0.84558
H   1.81271 -0.43079 -0.04874
N  -1.07132  2.86571 -0.39945
N   1.02874  2.96039  0.10020
N  -1.07155 -2.86575  0.39912
N   1.02848 -2.96040 -0.10067
C  -0.00018  2.06652 -0.09193
C   0.60855  4.27807 -0.09089
H   1.28780  5.11581  0.03044
C  -0.71662  4.21740 -0.41283
H  -1.43868  4.98966 -0.65772
C  -2.39991  2.40066 -0.70996
C  -2.71198  2.14955 -2.06240
C  -1.67754  2.31284 -3.17057
H  -0.69026  2.44389 -2.69414
C  -1.58210  1.06114 -4.06431
H  -1.34219  0.16684 -3.46718
H  -0.78781  1.19556 -4.81775
H  -2.52377  0.87133 -4.60665
C  -1.97183  3.57934 -4.00259
H  -2.95343  3.50518 -4.50114
H  -1.20447  3.71838 -4.78256
H  -1.98552  4.48202 -3.36922
C  -4.03669  1.76824 -2.35025
H  -4.32996  1.59004 -3.38945
C  -4.97746  1.61080 -1.32484
H  -5.99967  1.30534 -1.56885
C  -4.62040  1.83693  0.01181
H  -5.36334  1.69561  0.80057
C  -3.32086  2.25198  0.35218
C  -2.92547  2.57795  1.78958
H  -1.84834  2.34874  1.89554
C  -3.12515  4.08312  2.08297
H  -2.52172  4.71991  1.41775
H  -2.83602  4.31185  3.12262
H  -4.18466  4.36244  1.95331
C  -3.68237  1.73463  2.82747
H  -4.74959  2.01222  2.87811
H  -3.25639  1.90289  3.83020
H  -3.61776  0.66135  2.59657
C   2.36924  2.58679  0.46797
C   3.29135  2.30217 -0.56848
C   2.88970  2.42728 -2.03425
H   1.80008  2.24968 -2.09466
C   3.57176  1.38239 -2.93214
H   4.65634  1.56496 -3.02501
H   3.14737  1.42779 -3.94853
H   3.42856  0.36292 -2.54263
C   3.16769  3.85814 -2.54826
H   2.62262  4.61636 -1.96358
H   2.85790  3.95469 -3.60232
H   4.24448  4.09009 -2.48452
C   4.58723  1.91643 -0.18023
H   5.32790  1.67446 -0.94658
C   4.93913  1.84073  1.17452
H   5.95605  1.54931  1.45501
C   3.99939  2.12761  2.17334
H   4.28853  2.05850  3.22630
C   2.68058  2.49211  1.84236
C   1.64663  2.79105  2.92112
H   0.65765  2.84676  2.43380
C   1.56676  1.67295  3.97813
H   1.34111  0.70200  3.50767
H   0.76762  1.89782  4.70398
H   2.50853  1.57225  4.54392
C   1.93115  4.16335  3.56937
H   2.91456  4.16705  4.06996
H   1.16376  4.40093  4.32490
H   1.93453  4.97006  2.81749
C  -0.00035 -2.06653  0.09182
C  -0.71695 -4.21746  0.41201
H  -1.43907 -4.98975  0.65661
C   0.60819 -4.27813  0.08994
H   1.28740 -5.11587 -0.03163
C  -2.39998 -2.40056  0.71006
C  -3.32130 -2.25190 -0.35175
C  -2.92647 -2.57804 -1.78925
H  -1.84924 -2.34942 -1.89548
C  -3.68316 -1.73419 -2.82685
H  -4.75053 -2.01123 -2.87732
H  -3.25747 -1.90249 -3.82970
H  -3.61797 -0.66099 -2.59577
C  -3.12711 -4.08307 -2.08266
H  -2.52388 -4.72025 -1.41763
H  -2.83838 -4.31193 -3.12240
H  -4.18674 -4.36179 -1.95274
C  -4.62065 -1.83658 -0.01096
H  -5.36385 -1.69526 -0.79947
C  -4.97716 -1.61016  1.32577
H  -5.99924 -1.30451  1.57012
C  -4.03603 -1.76757  2.35086
H  -4.32890 -1.58919  3.39014
C  -2.71149 -2.14914  2.06258
C  -1.67668 -2.31268  3.17037
```

H	-0.68942	-2.44260	2.69357
C	-1.96995	-3.58019	4.00123
H	-2.95155	-3.50718	4.49995
H	-1.20238	-3.71947	4.78094
H	-1.98309	-4.48226	3.36697
C	-1.58179	-1.06181	4.06529
H	-1.34268	-0.16676	3.46896
H	-0.78713	-1.19643	4.81831
H	-2.52334	-0.87310	4.60821
C	2.36902	-2.58683	-0.46831
C	2.68041	-2.49200	-1.84270
C	1.64645	-2.79073	-2.92151
H	0.65748	-2.84650	-2.43420
C	1.93097	-4.16296	-3.56995
H	2.91439	-4.16661	-4.07050
H	1.16359	-4.40044	-4.32552
H	1.93432	-4.96976	-2.81815
C	1.56659	-1.67247	-3.97834
H	1.34092	-0.70162	-3.50771
H	0.76746	-1.89723	-4.70424
H	2.50837	-1.57166	-4.54411
C	3.99926	-2.12759	-2.17358
H	4.28845	-2.05838	-3.22652
C	4.93900	-1.84087	-1.17469
H	5.95594	-1.54950	-1.45512
C	4.58704	-1.91671	0.18003
H	5.32768	-1.67490	0.94645
C	3.29110	-2.30241	0.56818
C	2.88944	-2.42769	2.03395
H	1.79981	-2.25019	2.09437
C	3.57142	-1.38285	2.93196
H	4.65600	-1.56540	3.02487
H	3.14698	-1.42836	3.94832
H	3.42823	-0.36335	2.54254
C	3.16750	-3.85860	2.54776
H	2.62247	-4.61675	1.96297
H	2.85769	-3.95533	3.60181
H	4.24429	-4.09051	2.48402
H	0.31536	0.05711	1.63819

S3. References

(1) Fulmer, G. R.; Miller, A. J. M.; Sherden, N. H.; Gottlieb, H. E.; Nudelman, A.; Stoltz, B. M.; Bercaw, J. E.; Goldberg, K. I. NMR Chemical shifts of trace impurities: Common laboratory solvents, organics, and gases in deuterated solvents relevant to the organometallic chemist. *Organometallics* **2010**, *29*, 2176-2179.

(2) Martínez-Martínez, A. J.; Weller, A. S. Solvent-free anhydrous Li^+, Na^+ and K^+ salts of $[B(3,5-(CF_3)_2C_6H_3)_4]^-$, $[BAr_4^F]^-$. Improved synthesis and solid-state structures. *Dalton Trans.* **2019**, *48*, 3551–3554.

(3) https://pubchem.ncbi.nlm.nih.gov/compound/11010

(4) https://pubchem.ncbi.nlm.nih.gov/compound/10479.

(5) Tang, C. Y.; Thompson, A. L.; Aldridge, S. Dehydrogenation of saturated CC and BN bonds at cationic N-heterocyclic carbene stabilized M(III) centers (M = Rh, Ir). *J. Am. Chem. Soc.* **2010**, *132*, 10578-10591.

(6) DeBoef, B.; Pastine, S. J.; Sames, D. Cross-coupling of sp^3 C-H bonds and alkenes: Catalytic cyclization of alkene-amide substrates. *J. Am. Chem. Soc.* **2004**, *126*, 6556-6557.

(7) Sheldrick, G. M. SHELXT - Integrated space-group and crystal structure determination. *Acta Cryst.* **2015**, *A71*, 3-8.

(8) Sheldrick, G. M. Crystal structure refinement with SHELXL. *Acta Cryst.* **2015**, *C71*, 3-8.

(9) Dolomanov, O. V.; Bourhis, L. J.; Gildea, R. J.; Howard, J. A. K.; Puschmann, H. OLEX2: A complete structure solution, refinement and analysis program. *J. Appl. Cryst.* **2009**, *42*, 339-341.

(10) Heinekey, D. M.; Payne, N. G.; Schulte, G. K. Trihydrogen complexes of iridium. *J. Am. Chem. Soc.* **1988**, *110*, 2303-2305.

(11) Desrosiers, P. J.; Cai, L. H.; Lin, Z. R.; Richards, R.; Halpern, J. Assesment of the "T_1 criterion" for distinguishing between classical and non classical transition-metal hydrides:

Hydride relaxation rates in tris(triarylphosphine)osmium tetrahydrides and related polyhydrides. *J. Am. Chem. Soc.* **1991**, *113*, 4173-4184.

(12) Albinati, A.; Bakhmutov, V. I.; Caulton, K. G.; Clot, E.; Eckert, J.; Eisenstein, O.; Gusev, D. G.; Grushin, V. V.; Hauger, B. E.; Klooster, W. T.; Koetzle, T. F.; McMullan, R. K.; O'Loughlin, T. J.; Pélissier, M.; Ricci, J. S.; Sigalas, M. P.; Vymenits, A. B. Reaction of H_2 with $IrHCl_2P_2$ ($P = P^iPr_3$ or P^tBu_2Ph): Stereoelectronic control of the stability of molecular H_2 transition metal complexes. *J. Am. Chem. Soc.* **1993**, *115*, 7300-7312.

(13) Paneque, M.; Poveda, M. L.; Taboada, S. Deuteration studies of Tp^*IrH_4 (Tp^* = HB(3,5-$Me_2pz)_3$): Observation of very unusual ^{1}H NMR chemical shift effects. *J. Am. Chem. Soc.* **1994**, *116*, 4519-4520.

(14) Oldham, W. J., Jr.; Hinkle, A. S.; Heinekey, D. M. Synthesis and characterization of hydrotris(pyrazolyl)borate dihydrogen/hydride complexes of rhodium and iridium. *J. Am. Chem. Soc.* **1997**, *119*, 11028-11036.

(15) Gutiérrez-Puebla, E.; Monge, A.; Paneque, M.; Poveda, M. L.; Taboada, S.; Trujillo, M.; Carmona, E. Synthesis and properties of $Tp^{Me2}IrH_4$ and $Tp^{Me2}IrH_3(SiEt_3)$: Ir(V) polyhydride species with C_{3v} geometry. *J. Am. Chem. Soc.* **1999**, *121*, 346-354.

(16) Webster, C. E.; Singleton, D. A.; Szymanski, M. J.; Hall, M. B.; Zhao, C. Y.; Jia, G. C.; Lin, Z. Y. Minimum energy structure of hydridotris(pyrazolyl)borato iridium(V) tetrahydride is not a C_{3v} capped octahedron. *J. Am. Chem. Soc.* **2001**, *123*, 9822-9829.

(17) Gross, C. L.; Girolami, G. S. Synthesis and NMR studies of $[(C_5Me_5)Os(L)H_2(H_2)]^+$ complexes. Evidence of the adoption of different structures by a dihydrogen complex in solution and the solid state. *Organometallics* **2007**, *26*, 1658-1664.

(18) Hebden, T. J.; Goldberg, K. I.; Heinekey, D. M.; Zhang, X. W.; Emge, T. J.; Goldman, A. S.; Krogh-Jespersen, K. Dihydrogen/dihydride or tetrahydride? An experimental and

computational investigation of pincer iridium polyhydrides. *Inorg*. *Chem*. **2010**, *49*, 1733-1742.

(19) Hartwig, J. F.; De Gala, S. R. A continuum resulting from equilibrium between two structural extremes in tungstenocene and niobocene boryl and hydroborate complexes. π-Bonding in a d^2 boryl system and the first d^0 boryl complex. *J. Am. Chem. Soc.* **1994**, *116*, 3661-3662.

(20) Eastwood, J. B.; Procacci, B.; Gurung, S.; Lynam, J. M.; Hunt, N. T. Understanding the vibrational structure and ultrafast dynamics of the metal carbonyl precatalyst [Mn(ppy)$(CO)_4$]. *ACS Phys. Chem. Au* **2024**, DOI: 10.1021/acsphyschemau.4c00037.

(21) Deflores, L. P.; Nicodemus, R. A.; Tokmakoff, A. Two dimensonal Fourier transform spectroscopy in the pump-probe geometry. *Opt. Lett.* **2007**, *32*, 2966-2968.

(22) Shim, S. H.; Zanni, M. T. How to turn your pump-probe instrument into a multidimensional spectrometer: 2D IR and Vis spectroscopies via pulse shaping. *Phys. Chem. Chem. Phys.* **2009**, *11*, 748-761.

(23) Hamm, P.; Zanni, M. T. *Concepts and methods of 2D infrared spectroscopy*; Cambridge University Press, **2011**.

(24) Fernández-Terán, R.; Ruf, J.; Hamm, P. Vibrational couplings in hydridocarbonyl complexes: A 2D-IR perspective. *Inorg Chem* **2020**, *59*, 7721-7726.

(25) *Gaussian 16, Revision A.03*; Frisch, M. J.; Trucks, G. W.; Schlegel, H. B.; Scuseria, G. E.; Robb, M. A.; Cheeseman, J. R.; Scalmani, G.; Barone, V.; Petersson, G. A.; Nakatsuji, H.; et al.; Gaussian, Inc: Wallingford CT, 2016.

(26) Andrae, D.; Häußermann, U.; Dolg, M.; Stoll, H.; Preuß, H. Energy-adjusted ab initio pseudopotentials for the second and third row transition elements. *Theor. Chim. Acta* **1990**, *77*, 123-141.

(27) Hariharan, P. C.; Pople, J. A. The influence of polarization functions on molecular orbital hydrogenation energies. *Theor. Chim. Acta* **1973**, *28*, 213-222.

(28) Hehre, W. J.; Ditchfield, R.; Pople, J. A. Self-consistent molecular orbital methods XII. Further extensions of Gaussian-type basis sets for use in molecular orbital studies of organic molecules. *J. Chem. Phys.* **1972**, *56*, 2257-2261.

(29) Becke, A. D. Density-functional exchange-energy approximation with correct asymptotic behavior. *Phys. Rev. A* **1988**, *38*, 3098-3100.

(30) Perdew, J. P. Density-functional approximation for the correlation energy of the inhomogeneous electron gas. *Phys. Rev. B* **1986**, *33*, 8822-8824.

(31) Grimme, S.; Antony, J.; Ehrlich, S.; Krieg, H. A consistent and accurate ab initio parametrization of density functional dispersion correction (DFT-D) for the 94 elements H-Pu. *J. Chem. Phys.* **2010**, *132*, 154104.

(32) Grimme, S.; Ehrlich, S.; Goerigk, L. Effect of the damping function in dispersion corrected density functional theory. *J. Comput. Chem.* **2011**, *32*, 1456-1465.

(33) Weigend, F. Accurate Coulomb-fitting basis sets for H to Rn. *Phys. Chem. Chem. Phys.* **2006**, *8*, 1057-1065.

(34) Weigend, F.; Köhn, A.; Hättig, C. Efficient use of the correlation consistent basis sets in resolution of the identity MP2 calculations. *J. Chem. Phys.* **2002**, *116*, 3175-3183.

(35) Tomasi, J.; Mennucci, B.; Cammi, R. Quantum mechanical continuum solvation models. *Chem. Rev.* **2005**, *105*, 2999-3094.

(36) Bader, R. F. W. *Atoms in Molecules: A Quantum Theory*; Clarendon Press, **1994**.

(37) AIMAll (Version 17.11.14); Keith, T. A.; T. K. Gristmill Software: Overland Park KS, USA, 2017.

(38) Chemcraft - graphical software for visualization of quantum chemistry computations. http://www.chemcraftprog.com.

(39) Perdew, J. P.; Burke, K.; Ernzerhof, M. Generalized gradient approximation made simple. *Phys. Rev. Lett.* **1996**, *77*, 3865-3868.

(40) Lee, C.; Yang, W.; Parr, R. G. Development of the Colle-Salvetti correlation-energy formula into a functional of the electron density. *Phys. Rev. B* **1988**, *37*, 785-789.

(41) Grimme, S. Semiempirical GGA-type density functional constructed with a long-range dispersion correction. *J. Comput. Chem.* **2006**, *27*, 1787-1799.

(42) Tao, J.; Perdew, J. P.; Staroverov, V. N.; Scuseria, G. E. Climbing the density functional ladder: Nonempirical meta-generalized gradient approximation designed for molecules and solids. *Phys. Rev. Lett.* **2003**, *91*, 146401.

(43) Adamo, C.; Barone, V. Toward reliable density functional methods without adjustable parameters: The PBE0 model. *J. Chem. Phys.* **1999**, *110*, 6158-6170.

(44) Becke, A. D. Density-functional thermochemistry. III. The role of exact exchange. *J. Chem. Phys.* **1993**, *98*, 5648-5652.

(45) Chai, J.-D.; Head-Gordon, M. Long-range corrected hybrid density functionals with damped atom–atom dispersion corrections. *Phys. Chem. Chem. Phys.* **2008**, *10*, 6615-6620.

(46) Perdew, J. P.; Burke, K.; Wang, Y. Generalized gradient approximation for the exchange-correlation hole of a many-electron system. *Phys. Rev. B* **1996**, *54*, 16533-16539.

(47) Zhao, Y.; Truhlar, D. G. The M06 suite of density functionals for main group thermochemistry, thermochemical kinetics, noncovalent interactions, excited states, and transition elements: two new functionals and systematic testing of four M06-class functionals and 12 other functionals. *Theor. Chem. Acc.* **2008**, *120*, 215-241.
